# CRL4^AMBRA1^ is a key mediator for AKT-dependent cell cycle control in neural progenitor cells

**DOI:** 10.1038/s44319-026-00768-7

**Published:** 2026-04-27

**Authors:** He Wang, Panmiao Liu, Runmin Wang, Hanwen Gu, Tingting Zhu, Guiquan Chen, Jian-Jun Yang

**Affiliations:** 1 Department of Anesthesiology, Pain and Perioperative Medicine, The First Affiliated Hospital of Zhengzhou University, Zhengzhou, China; 2 MOE Key Laboratory of Model Animal for Disease Study, Model Animal Research Center, Medical School, Nanjing University, Nanjing, China; 3 Department of Anesthesiology and Perioperative Medicine, The First Affiliated Hospital with Nanjing Medical University, Nanjing, China

**Keywords:** Cell Cycle, Neuroscience, Post-translational Modifications & Proteolysis

## Abstract

Neural progenitor cell (NPC) proliferation is fundamental for population expansion and brain development. G_1_ phase control determines the cell cycle duration of NPCs and thereby affects their proliferation efficiency. However, the molecular mechanisms governing G_1_ phase progression in NPCs remain unclear. Here, we show that AKT gain-of-function mutations and pharmacological inhibition exert opposing effects on NPC proliferation. Consistently, *Emx1-Cre*-mediated deletion of *Akt1/2/3* in mice impairs NPC proliferation and disrupts cortical development. We find that AKT deficiency induces G_1_ phase arrest and prolongs the cell cycle of NPCs. Mechanistically, we demonstrate that AKT-mediated phosphorylation inhibits the activity of CRL4^AMBRA1^ E3 ubiquitin ligase to safeguard cyclin D2 (CCND2) stability. Specifically, AKT phosphorylates DDB1, the adaptor of CRL4^AMBRA1^, which disrupts its interaction with CCND2 and reduces its degradation. These findings reveal a post-translational mechanism impacting NPC cell cycle and cortical morphogenesis, providing insight into the etiology of malformations of cortical development.

## Synopsis

This study reveals a post-translational mechanism impacting NPC cell cycle and cortical morphogenesis, showing that AKT maintains CCND2 stability through inhibition of CRL4^AMBRA1^ activity during NPC G_1_ phase progression.

- Genetic deletion of *Akt1/2/3* in mice impairs NPC proliferation and disrupts cortical development.
- AKT deficiency induces G_1_ phase arrest and prolongs the cell cycle of NPCs.
- AKT phosphorylates DDB1, the adaptor of CRL4^AMBRA1^, to reduce CCND2 degradation.

## Abstract (web summary)

This study reveals a post-translational mechanism impacting NPC cell cycle and cortical morphogenesis, showing that AKT maintains CCND2 stability through inhibition of CRL4^AMBRA1^ activity during NPC G_1_ phase progression.

## Graphical abstract

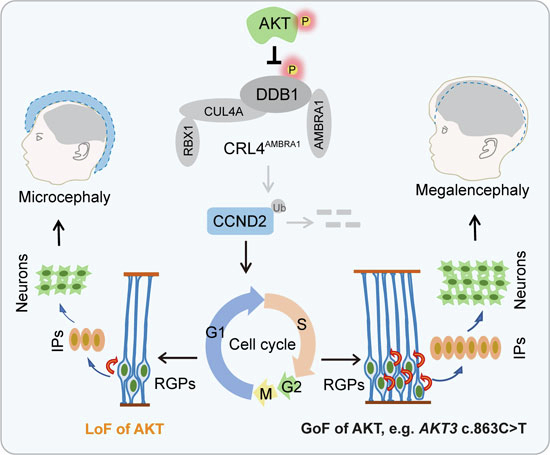

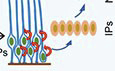

## Introduction

Protein kinase B (AKT) is a key serine/threonine kinase comprising three isoforms: AKT1, AKT2 and AKT3 (Pearce et al, 2010). AKT is well recognized for its critical roles in cell survival, metabolism, and oncogenesis (Manning and Toker, 2017). Clinical evidence indicates that AKT dysfunction is associated with malformations of cortical development (MCDs) (Boland et al, 2007; Poduri et al, 2012; Riviere et al, 2012; Thierry et al, 2012). Specifically, AKT loss-of-function is linked to microcephaly (Boland et al, 2007; Thierry et al, 2012), while AKT gain-of-function mutations are associated with megalencephaly (Poduri et al, 2012; Riviere et al, 2012). These findings underscore the essential role of AKT in brainmorphogenesis.

Recent studies have begun to uncover the functions of AKT in the central nervous system (CNS). It has been shown that *Akt3* knockout (KO) mice exhibit reduced brain size compared to wild-type (WT) controls (Tschopp et al, 2005), and AKT inhibition results in dendritic abnormalities (Jossin and Goffinet, 2007), highlighting the importance of AKT in brain development. Deletion of *Akt2* or *Akt3* has been shown to impair synaptic plasticity, learning and memory in mice (Howell et al, 2017; Levenga et al, 2017; Palumbo et al, 2021; Wang et al, 2017; Zhang et al, 2019), suggestive of the involvement of AKT in cognitive function. In addition, our previous work demonstrated that oligodendrocyte (OL) lineage-specific inactivation of *Pdk1* or *Akt1/2/3* blocks the differentiation of OL precursor cells (OPCs) in CNS (Wang et al, 2021a; Wang et al, 2021b), indicating a requirement for AKT in OPC development. Despite these insights, the role of AKT in regulating neural progenitor cell (NPC) fate during cortical morphogenesis remains poorly understood.

In rodents, NPCs are classified as radial glial progenitors (RGPs) and intermediate progenitors (IPs) (Mukhtar et al, 2022), and are primarily localized in the ventricular zone (VZ) and the subventricular zone (SVZ) (Gotz and Huttner, 2005; Zhang and Jiao, 2015). A fundamental characteristics of NPCs is their ability to proliferate (Greig et al, 2013; Xia and Jiao, 2017), a process critical for cortical development (Li et al, 2023; Taverna et al, 2014; Wang et al, 2016). NPC proliferation proceeds through four distinct cell cycle phases: G_1_, S, G_2_, and M (Liu et al, 2019; Molyneaux et al, 2007). The control of G_1_ phase progression is a critical determinant of cell cycle duration in NPCs (Lange et al, 2009; Pilaz et al, 2009; Salomoni and Calegari, 2010). While numerous regulatory molecules modulate the cell cycle progression of NPCs (Grison and Atanasoski, 2020; Lim and Kaldis, 2012; Mairet-Coello et al, 2012), D-type cyclins (CCNDs) are key drivers of G_1_ phase progression (Lukas et al, 1995). Notably, CCND2 is involved in brain development. *CCND2* mutations are linked to cortical malformations in human (Sameshima et al, 2020), and *Ccnd2* deletion causes microcephaly in mice (Glickstein et al, 2009). Recently, it has been reported that the CRL4^AMBRA1^ E3 ubiquitin ligase is the master regulator for CCND stability (Chaikovsky et al, 2021; Maiani et al, 2021; Simoneschi et al, 2021). Since the protein levels of CCNDs are dynamic throughout the cell cycle progression, we hypothesize that the activity of CRL4^AMBRA1^ should be regulated to control the expression of CCNDs.

To address these questions, we conducted a series of molecular and cellular investigations. We observed robust AKT expression in cultured human NPCs and in the developing mouse cortex. We found that AKT gain-of-function mutations and pharmacological inhibition exerted opposing effects on NPC proliferation. We showed that conditional triple knockout (cTKO) of *Akt1/2/3* in NPCs led to reduced progenitor populations and a thinner cortex in mice. Cell cycle analyses revealed G_1_ phase arrest in *Akt* cTKO NPCs, accompanied by downregulation of CCND2. We identified that DDB1, the adaptor of CRL4^AMBRA1^, is a direct AKT target. AKT phosphorylates DDB1 at threonine 1125 to maintain the stability of CCND2. Collectively, our findings reveal a novel post-translational mechanism that governs NPC cell cycle and cortical development.

## Results

### AKT is essential for NPC proliferation in vitro

**Figure 1:**
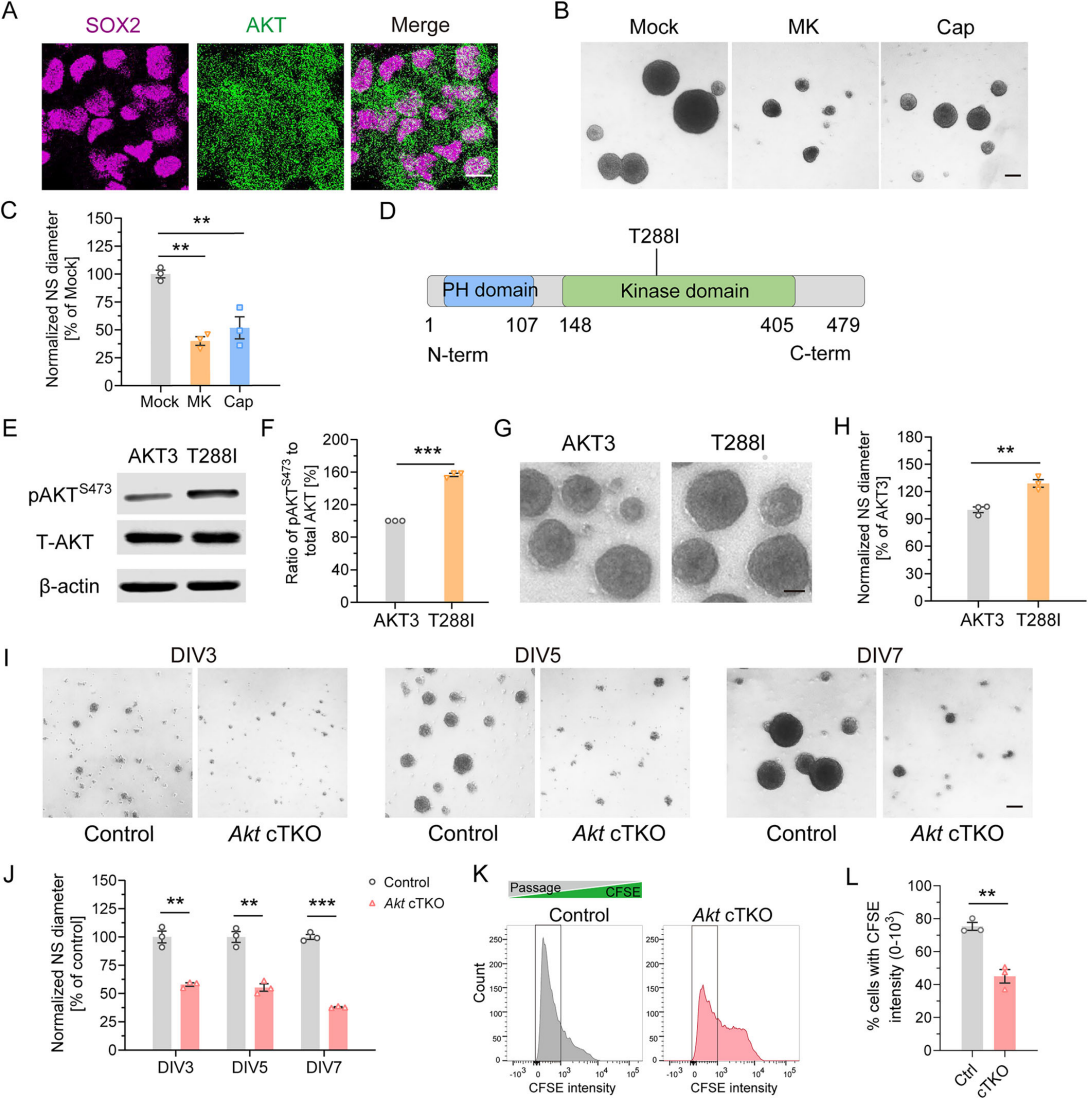
Loss of AKT causes decreased size of neurospheres. (**A**) Representative images of AKT and SOX2 co-staining in NPCs derived from the E15.5 mouse cortex at day 3 in vitro (DIV3). Strong Akt immunofluorescence was observed in SOX2+ cells. Scale bar: 10 μm. (**B**) Representative images of cultured neurospheres. Neurospheres derived from the mouse cortex were treated with 1 μM MK2206 (MK) or 1 μM capivasertib (Cap) for 6 days. Neurospheres treated with MK or Cap were smaller compared to untreated controls. Scale bar: 100 μm. (**C**) Normalization of neurosphere diameter. There were significant differences in the neurosphere diameter between Mock-, MK- and Cap-treated groups. Results are shown as mean ± SEM (*n* = 3 independent experiments, one-way ANOVA, **P = 0.0014 for MK, *P* = 0.0044 for Cap). (**D**) The diagram of *AKT3* mutation (c.863 C > T, p.T288I). (**E**, **F**) Western blot analysis for AKT and pAKT^S473^ in the 293 T cells. These cells were transfected with *AKT3* or *AKT3* mutation (c.863 C > T, p.T288I) for 24 h. The ratio of pAKT^S473^ to total AKT (T-AKT) was significantly increased in the cells transfected with *AKT3* mutation (c.863 C > T, p.T288I) compared to that in cells transfected with *AKT3*. Data are shown as mean ± SEM (*n* = 3 independent experiments, Student’s *t* test, ***P = 8.743 × 10^−6^). (**G**, **H**) Representative images and the average diameter for cultured neurospheres expressing WT and mutant AKT3. The neurospheres were infected with lentivirus expressing AKT3 or AKT3 mutation (c.863 C > T, p.T288I) and images were captured at day 7 during passage 2. Neurospheres infected with a lentivirus expressing the AKT3 mutation (c.863 C > T, p.T288I) showed a larger size than AKT3. Data are shown as mean ± SEM (*n* = 3 independent experiments, Student’s *t* test, **P = 0.0052). Scale bar: 50 μm. (**I**) Representative images of neurospheres cultured from mouse cortices at DIV3, DIV5 and DIV7. Neurospheres from *Akt* cTKO mice were smaller than those from controls. Scale bar: 100 μm. (**J**) Normalized neurosphere diameters. The average diameter of neurospheres from *Akt* cTKO mice was significantly reduced compared to controls. Data are shown as mean ± SEM (*n* = 3 independent experiments, Student’s *t* test, **P = 0.0014 for DIV3, *P* = 0.0016 for DIV5, ***P = 1.029 × 10^−5^). (**K**) CFSE-labeling assay in cultured neurospheres. NPCs were isolated from the cortices of control and *Akt* cTKO mice at E15.5. CFSE was added to the culture medium at DIV0, and fluorescence intensities were analyzed by FC at DIV5. (**L**) Percentage of cells with CFSE intensities between 0 and 10^3^. There was a significant decrease in neurospheres within this intensity range from *Akt* cTKO mice compared to controls. The results are shown as mean ± SEM (*n* = 3 independent experiments, Student’s *t* test, **P = 0.0031). Source data are available online for this figure.

To investigate the role of AKT in NPC fate regulation, we first examined its expression pattern in cultured human-derived NPCs (ENStem-A, ENSA) (Li et al, 2022). Immunostaining revealed strong AKT immunoreactivity in SOX2-positive (SOX2 + ) cells (Appendix Fig. S1A). Similarly, co-labeling of SOX2 and AKT in both cultured mouse cortical NPCs at embryonic day 15.5 (E15.5) and in brain sections at E13.5 confirmed robust AKT expression in SOX2+ NPCs (Fig. 1A; Appendix Fig. S1B). These results indicate that AKT is abundantly expressed in NPCs across species and developmental contexts.

To determine whether AKT is required for NPC proliferation, we treated mouse cortical neurospheres with two selective inhibitors: MK2206, an allosteric AKT inhibitor, (Hirai et al, 2010; Wang et al, 2021a) and capivasertib (Cap), an ATP-competitive AKT inhibitor (Smyth et al, 2020). Both inhibitors significantly reduced phosphorylated AKT substrate (PAS) levels as confirmed by Western blot (Appendix Fig. S1C), and caused a marked reduction in neurosphere size compared to mock-treated controls (Fig. 1B,C). Co-immunostaining for KI67 and SOX2 revealed that AKT inhibitors significantly decreased the ratio of KI67 + /SOX2+ cells to total SOX2+ cells (Appendix Fig. S1D,E), indicating suppressed NPC proliferation upon AKT inhibition. To assess whether AKT inhibition affects NPC survival, TUNEL assay was performed (Appendix Fig. S1F). The ratio of TUNEL+ cells to DAPI+ cells was comparable between MK2206-, Cap- and mock-treated NPCs (Appendix Fig. S1G), suggesting that AKT inhibition does not induce abnormal cell death.

We next examined the effect of a human *AKT3* gain-of-function mutation (c.863 C > T; p.T288I) associated with megalencephaly (Lai et al, 2022) (Fig. 1D). HEK293T cells expressing AKT3-p.T288I exhibited elevated pAKT^S473^ levels compared to WT AKT3, confirming its gain-of-function status (Fig. 1E,F). When expressed in cultured mouse neurospheres (Appendix Fig. S1H–J), the p.T288I mutant significantly increased neurosphere size compared to WT AKT3 controls (Fig. 1G,H). Co-immunostaining for KI67 and SOX2 showed that the p.T288I mutant caused significantly increased ratio of KI67 + /SOX2+ cells to total SOX2+ cells (Appendix Fig. S1K,L). These results suggest that AKT may promote NPC proliferation.

To determine the consequences of AKT loss in NPCs, we generated NPC-specific *Akt* cTKO mice (*Akt1*^*f/f*^*;Akt2*^*-/-*^*;Akt3*^*f/f*^ (Wang et al, 2021a) crossed with *Emx1-Cre* (Gorski et al, 2002; Xu et al, 2017; Ye et al, 2022)) (Appendix Fig. S2A). GFP reporter analysis in *mTmG*;*Emx1-Cre* mice confirmed dorsal telencephalon-specific Cre activity (Appendix Fig. S2B,C). Quantitative real-time PCR (qRT-PCR) and Western blot analyses confirmed substantial reductions in AKT1 and AKT3 transcripts and protein levels, including pAKT^S473^, in the dorsal telencephalon of *Akt* cTKO embryos (Appendix Fig. S2D–F). Immunohistochemistry (IHC) showed marked loss of AKT immunoreactivity in the mutant cortex at E13.5 (Appendix Fig. S2G) and E15.5 (Appendix Fig. S2H), confirming efficient inactivation. Western blot of cultured neurospheres from E15.5 cortices confirmed efficient deletion, with substantially reduced T-AKT and pAKT^S473^ levels in *Akt* cTKO samples (Appendix Fig. S2I,J). Correspondingly, AKT-deficient neurospheres were significantly smaller than controls at DIV3, DIV5, and DIV7 (Fig. 1I,J).

Proliferative capacity was further evaluated using carboxyfluorescein diacetate succinimidyl ester (CFSE) dilution and flow cytometry (FC) (Groszer et al, 2001). At DIV5, a high percentage of control NPCs exhibited low CFSE intensity, indicative of active proliferation. In contrast, *Akt* cTKO cultures showed a decreased proportion of low CFSE-intensity cells, suggesting reduced proliferation (Fig. 1K,L). Together, these results establish that AKT is essential for NPC proliferation.

### AKT is required for cortical growth and maintenance of NPC populations

**Figure 2:**
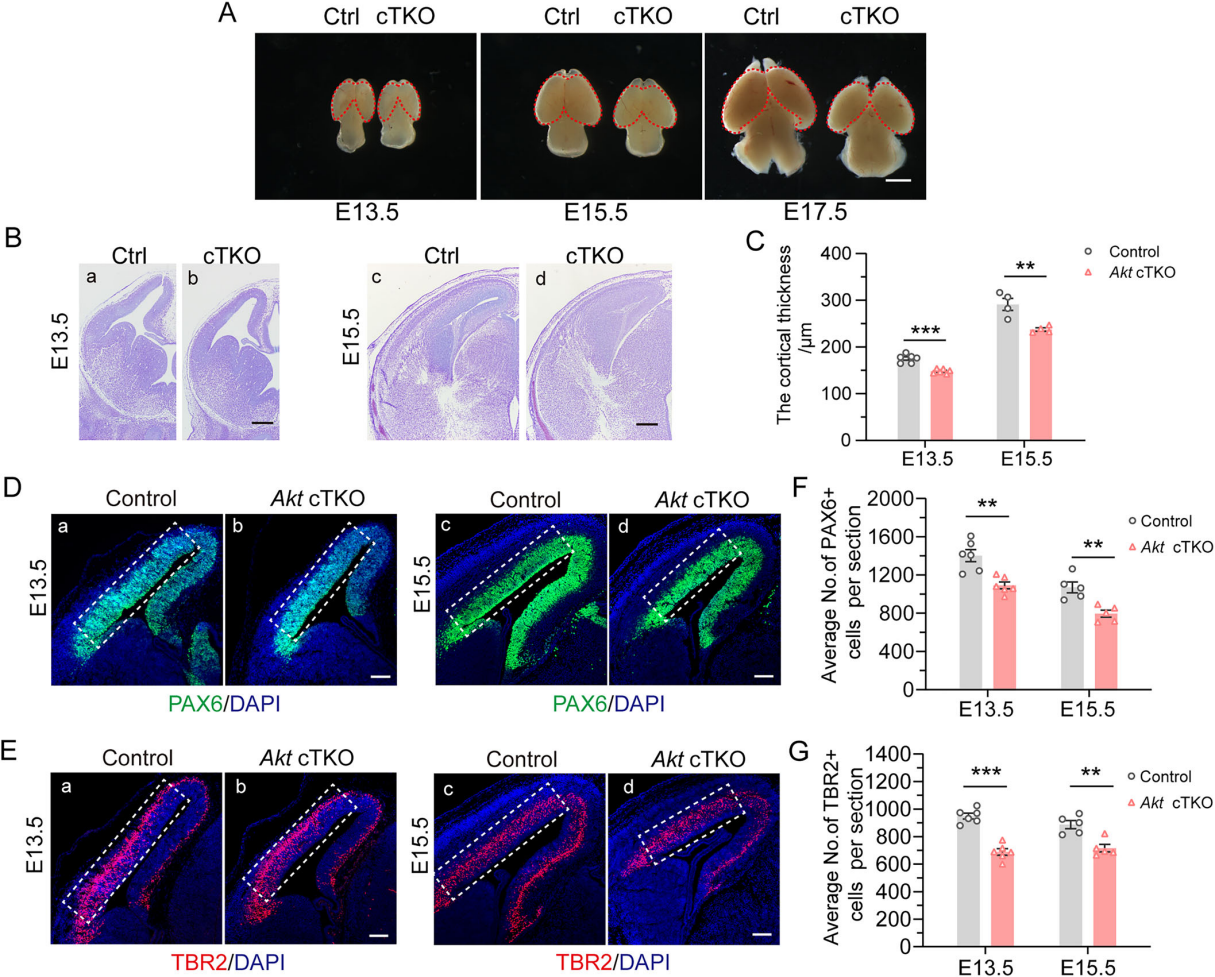
Decreased sub-populations of NPCs in *Akt* cTKO mice. (**A**) Brain images of control and *Akt* cTKO mice at E13.5, E15.5 and E17.5. The cortex of *Akt* cTKO mice was smaller compared to controls. The scale bar is 1 mm. (**B**) Nissl staining of mouse brains. Coronal sections from control and *Akt* cTKO mice at E13.5 and E15.5 were analyzed. Scale bar: 200 μm. (**C**) Quantification of the average cortical thickness. There was significant reduction in the cortical thickness in *Akt* cTKO mice compared to controls. Data are shown as mean ± SEM (*n* = 4–6 mice per genotype, Student’s *t* test, **P = 0.0071, ***P = 6.784  × 10^−5^). (**D**) Representative fluorescence images for PAX6. Cortices from control and *Akt* cTKO mice at E13.5 and E15.5 were examined. PAX6+ cells were counted within the dorsal cortical region adjacent to the ventricle, as delineated by the dashed borders. Scale bar: 100 μm. (**E**) Representative fluorescence images for TBR2. These TBR2+ cells within the dorsal cortex along the ventricle, marked by the dashed borders, were counted. Scale bar: 100 μm. (**F**) The average number of PAX6+ cells in the cortex across brain sections. There was significant reduction in *Akt* cTKO mice at E13.5 or E15.5 compared to littermate controls. Data are shown as mean ± SEM (*n* = 5–6 mice per group, Student’s *t* test, **P = 0.0016 for E13.5, *P* = 0.0036 for E15.5). (**G**) The average number of TBR2+ cells in the cortex across brain sections. There was significant reduction in *Akt* cTKO mice at E13.5 or E15.5. Data are shown as mean ± SEM (*n* = 5–6 mice per group, Student’s *t* test, ***P = 8.780 × 10^−6^, **P = 0.0028). Source data are available online for this figure.

To investigate the in vivo consequences of AKT loss, we examined *Akt* cTKO embryos at E13.5, E15.5, and E17.5. Visual examination indicated that *Akt* cTKO brains were notably smaller than littermate controls (Fig. 2A). To assess cortical morphology, brain sections at different ages were used for Nissl staining analysis (Fig. 2B). Quantification results revealed significant reductions in the thickness of the dorsal cortex in *Akt* cTKO mice at E13.5 or E15.5 compared to littermate controls (Fig. 2C). Overall, *Akt* cTKO mice exhibited microcephaly, suggesting a requirement of AKT in cortical morphogenesis.

To examine NPC subtypes, we performed immunostaining for PAX6, SOX2, and TBR2. All three populations, apical progenitors (PAX6 + , SOX2 +) and intermediate progenitors (TBR2 +) were significantly reduced in *Akt* cTKO cortices at E13.5 and E15.5 (Fig. 2D–G; Appendix Fig. S2K,L). These findings indicate that AKT is required to sustain NPC populations during cortical development.

### Conditional deletion of AKT1/2/3 impairs NPC proliferation without affecting cell survival

**Figure 3:**
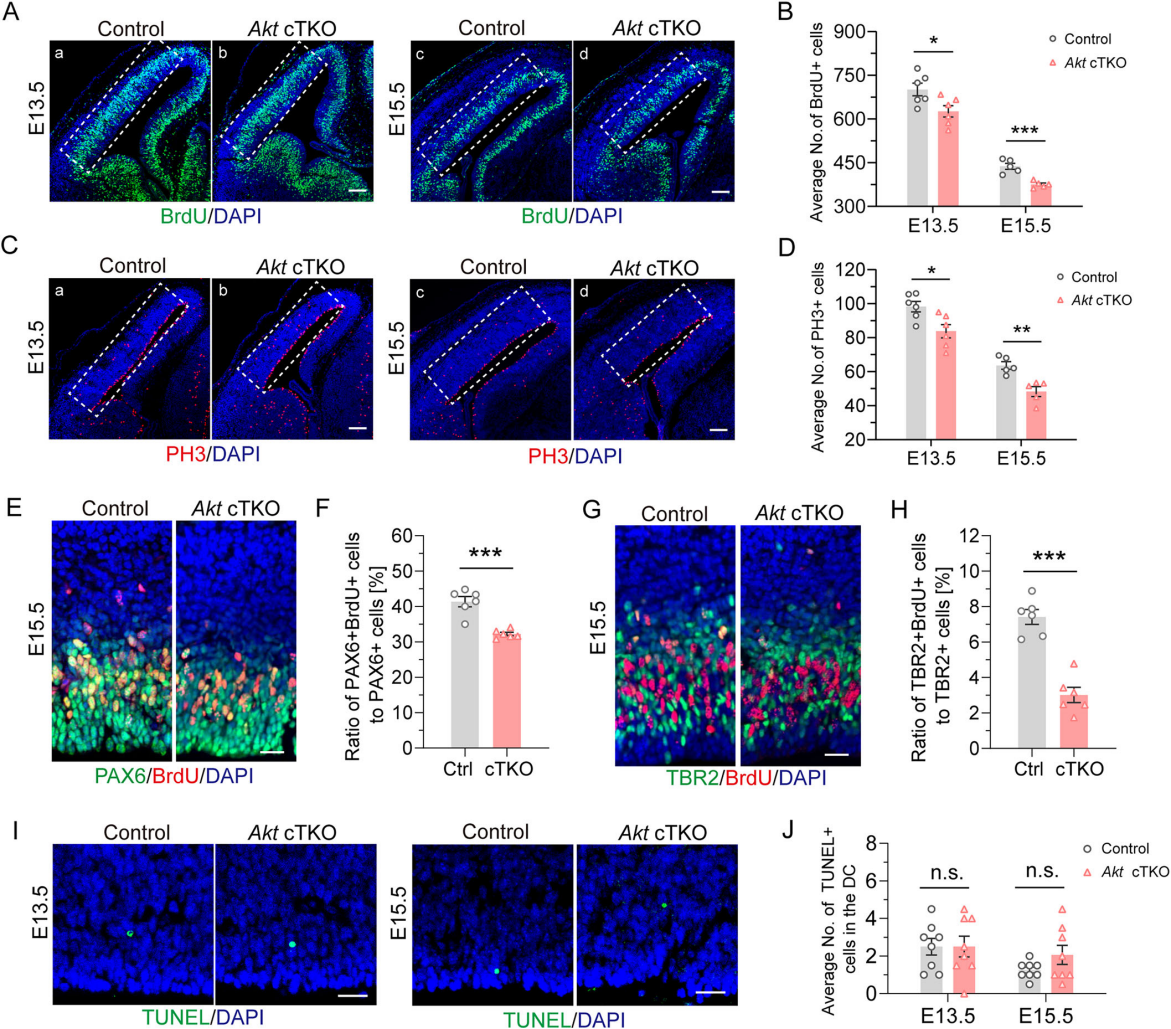
Impaired NPC proliferation in *Akt* cTKO mice. (**A**) Representative fluorescence images for BrdU staining. Pregnant mice at E13.5 and E15.5 were injected with BrdU and analyzed after half an hour. BrdU+ cells were counted within the dorsal cortical region adjacent to the ventricle, as marked by the dashed borders. Scale bar: 100 μm. (**B**) Quantification of BrdU+ cells in the cortex. The average number of BrdU+ cells across brain sections was significantly decreased in *Akt* cTKO mice at E13.5 or E15.5 compared to littermate controls. Data are shown as mean ± SEM (*n* = 5–6 mice per group, Student’s *t* test, *P = 0.0272, ***P = 0.0006). (**C**) Representative fluorescence images for PH3. Brain sections from control and *Akt* cTKO mice at E13.5 and E15.5 were analyzed. PH3+ cells within the dorsal cortex along the ventricle, delineated by the dashed borders, were counted. Scale bar: 100 μm. (**D**) Quantification of PH3+ cells in the cortex. A significant decrease in the number of PH3+ cells was observed in *Akt* cTKO mice at E13.5 or E15.5 compared to littermate controls. Data are shown as mean ± SEM (*n* = 5–6 mice per group, Student’s *t* test, *P = 0.0102, **P = 0.0030). (**E**) Representative fluorescence images of co-staining for PAX6/BrdU. Pregnant mice at E15.5 were injected with BrdU and analyzed after half an hour. Scale bar: 20 μm. (**F**) Quantification of PAX6 + /BrdU+ cells in the cortex. The ratio of PAX6 + /BrdU+ cells to PAX6+ cells was significantly reduced in *Akt* cTKO mice at E15.5 compared to littermate controls. Data are shown as mean ± SEM (*n* = 6 mice per group, Student’s *t* test, ***P = 0.0001). (**G**) Representative fluorescence images of co-staining for TBR2/BrdU in control and *Akt* cTKO cortices at E15.5, 30 min after BrdU injection. Scale bar: 20 μm. (**H**) Quantification of TBR2 + /BrdU+ cells in the cortex. The ratio of TBR2 + /BrdU+ cells to TBR2+ cells was significantly decreased in *Akt* cTKO mice at E15.5 compared to littermate controls. Data are shown as mean ± SEM (*n* = 6 mice per group, Student’s *t* test, ***P = 2.470 × 10^−5^). (**I**) Representative fluorescence images for TUNEL+ cells. Brain sections from control and *Akt* cTKO mice at E13.5 and E15.5 were used for TUNEL staining. Scale bar: 20  μm. (**J**) The average number of TUNEL+ cells in the dorsal cortex (DC). There was no significant difference between control and *Akt* cTKO mice at E13.5 or E15.5. Data are shown as mean ± SEM (*n* = 8 mice per group, Student’s *t* test, n.s. *P* > 0.9999 for E13.5, *P* = 0.2354 for E15.5). Source data are available online for this figure.

5-bromo-2’-deoxyuridine (BrdU) pulse-labeling revealed significantly fewer BrdU+ cells in *Akt* cTKO cortices at E13.5 and E15.5 (Fig. 3A,B), indicating reduced population of proliferating NPCs at the S-phase of the cell cycle. Similarly, mitotic activity assessed via phospho-histone H3 (PH3) immunostaining showed decreased numbers of PH3+ NPCs in cTKO cortices (Fig. 3C,D). To investigate the effect of AKT deletion on the proliferation of RGPs and IPs, IHC for PAX6/BrdU and TBR2/BrdU was performed using brain sections from control and *Akt* cTKO mice at E15.5 injected with BrdU for 30 min (Fig. 3E,G). While the proportion of BrdU + /PAX6+ cells to total PAX6+ cells was significantly reduced in *Akt* cTKO mice (Fig. 3F), the proportion of BrdU + /TBR2+ cells to total TBR2+ cells was also decreased (Fig. 3H), indicating that AKT loss impairs NPC proliferation. TUNEL staining revealed no significant difference in apoptotic cell numbers between controls and *Akt* cTKO cortices (Fig. 3I,J), suggesting that AKT loss does not affect NPC survival. Immunostaining for neuronal markers TBR1 and CTIP2 showed reduced numbers of TBR1+ and CTIP2+ cells in *Akt* cTKO cortices (Appendix Fig. S3A–D), suggesting decreased neurogenesis.

### Conditional deletion of AKT1/2/3 alters expression of cell cycle-related genes in NPCs

**Figure 4:**
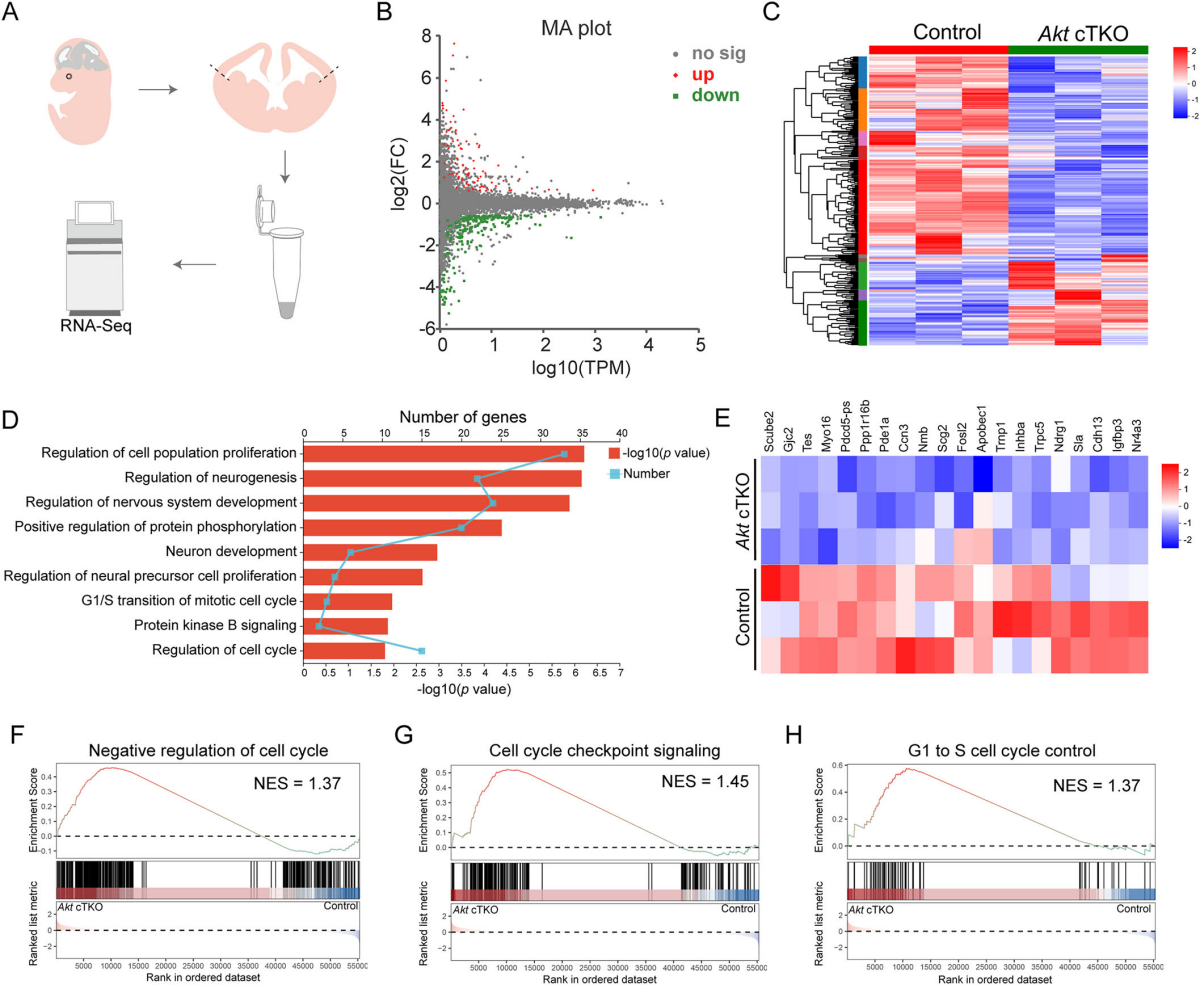
Transcriptomic analysis of *Akt* cTKO NPCs. (**A**) Workflow for RNA sequencing experiments. Mouse cortices were dissected at E13.5, and total RNA were extracted. RNA-seq libraries were then constructed and sequenced using the Illumina NovaSeq 6000 platform. (**B**) MA plot of cortical RNAs in control and *Akt* cTKO mice. Compared to controls 83 genes were upregulated and 197 genes were downregulated in *Akt* cTKO mice, with a fold change greater than 1.5 (*n* = 3 samples/group, each sample consisted of at least three embryonic cortices). (**C**) The heatmap of DEGs in *Akt* cTKO mice. A range of DEGs were shown for *Akt* cTKO mice at E13.5. (**D**) GO analysis of DEGs. DEGs were enriched in various signaling pathways, including cell proliferation, neurogenesis, nervous system development, PKB and cell cycle. (**E**) The heatmap showing a subset of downregulated genes involved in NPC proliferation in *Akt* cTKO mice at E13.5. (**F**–**H**) GSEA of cell cycle-related genes. There were significant changes in genes associated with negative regulation of cell cycle (**F**), cell cycle checkpoint signaling (**G**), and G_1_ to S cell cycle control (**H**) in *Akt* cTKO mice. (*n* = 3 samples/group, Permutation test, *P* < 0.001 (**F**), *P* = 0.0045 (**G**), *P* = 0.0333 (**H**), NES, normalized enrichment score). Source data are available online for this figure.

To elucidate molecular changes underlying reduced NPC proliferation, we performed RNA-seq on dorsal telencephalon tissue from control and *Akt* cTKO embryos at E13.5 (Fig. 4A). Differential gene expression analysis identified 197 downregulated and 83 upregulated genes in mutants (Fig. 4B,C). Gene ontology (GO) and gene set enrichment analysis (GSEA) analyses showed significant enrichment of differentially expressed genes (DEGs) in pathways related to cell proliferation, neurogenesis, PKB/AKT signaling, and the cell cycle (Fig. 4D–H).

Specifically, the expression of genes involved in cell cycle checkpoints, G_1_-to-S phase transition, and negative regulation of the cell cycle were altered in *Akt* cTKO samples (Fig. 4E–H). Neurogenesis-related gene sets, including those for cortical plate neurons and general neuronal function, were suppressed (Appendix Fig. S4A–D), in agreement with reduced TBR1+ and CTIP2+ cell numbers.

### Conditional deletion of AKT1/2/3 prolongs NPC cell cycle and impairs INM

**Figure 5:**
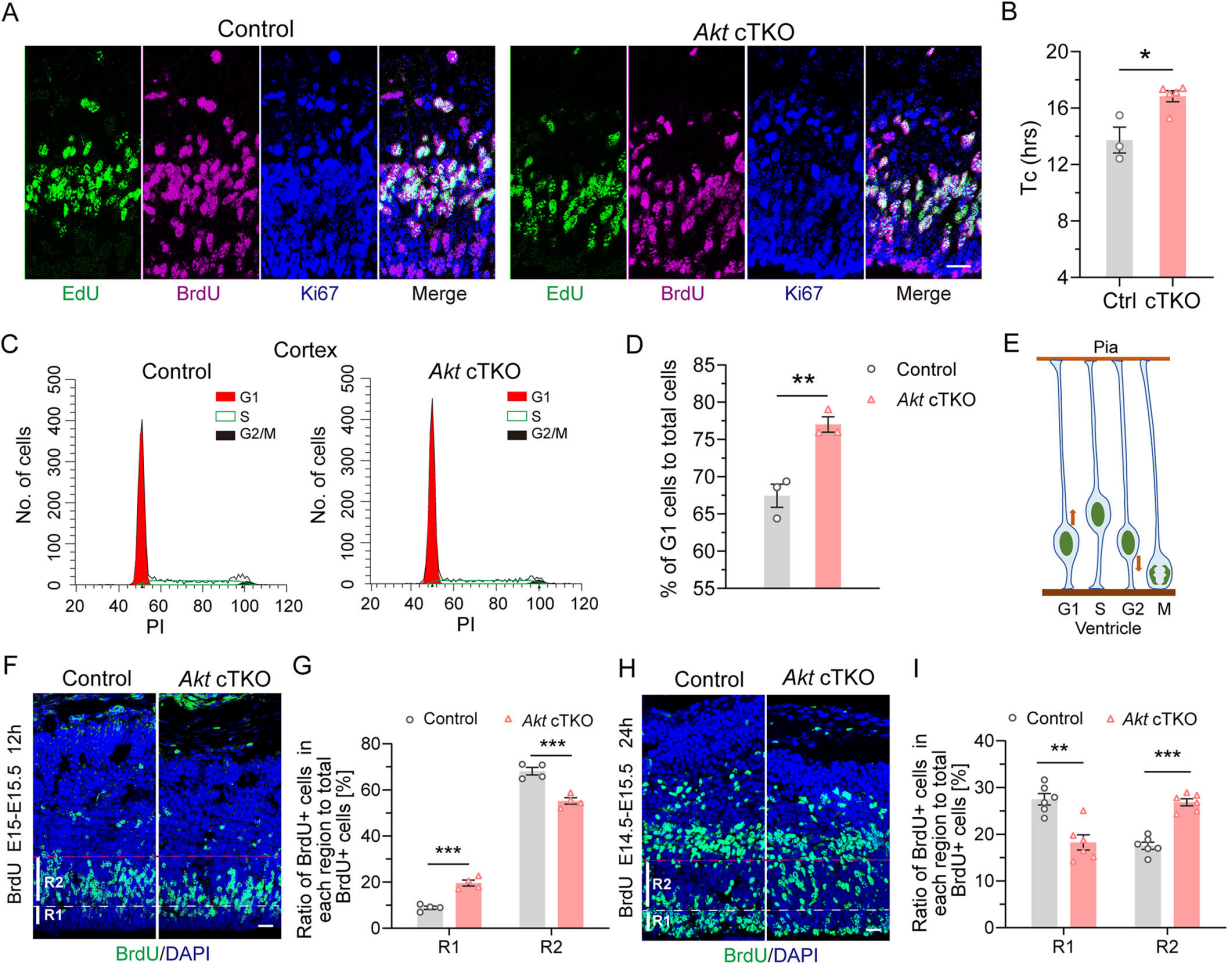
Prolonged cell cycle duration in *Akt* cTKO NPCs. (**A**) Representative fluorescence images of EdU/BrdU/Ki67 co-staining. Control and *Akt* cTKO mice at E15.5 received BrdU injection, followed by EdU injection 1.5 h later. Scale bar: 20  μm. (**B**) Quantification of Tc. There was a significant increase in Tc in *Akt* cTKO NPCs compared to controls. Data are shown as mean ± SEM (*n* = 3–5 mice per group, Student’s *t* test, *P = 0.0104). (**C**) The number of cells at distinct cell cycle phases. Cortical tissues from control and *Akt* cTKO mice at E13.5 were dissociated, stained with PI and analyzed with an FC system. (**D**) Percentage of cells in the G_1_ phase relative to the total cell population. There was a significant increase in G_1_ phase cells in the cortex of *Akt* cTKO mice compared to controls. Data are shown as mean ± SEM (*n* = 3 mice per group, Student’s *t* test, **P = 0.0067). (**E**) Schematic diagram illustrating the interkinetic nuclear migration of RGPs across different cell cycle phases. Red arrows indicate the direction of radial glial nuclear migration. (**F**) Representative fluorescence images for BrdU. Control and *Akt* cTKO mice received BrdU injection at E15, and cortical samples were collected 12 h later. Scale bar: 20 μm. (**G**) Ratio of BrdU+ cells in each region to total BrdU+ cells [%]. There was significant increase in R1 in *Akt* cTKO mice compared to controls. Data are shown as mean ± SEM (*n* = 4 mice per group, Student’s *t* test, ***P = 0.0004 for R1, *P* = 0.00099 for R2). (**H**) Representative fluorescence images for BrdU. Cortical samples were collected at E15.5, 24 h after the BrdU injection. Scale bar: 20 μm. (**I**) Ratio of BrdU+ cells in each region to total BrdU+ cells [%]. There was a significant decrease in R1 in *Akt* cTKO mice compared to controls. Data are shown as mean ± SEM (*n* = 6 mice per group, Student’s *t* test, **P = 0.0010, ***P = 6.080 × 10^−6^). Source data are available online for this figure.

To examine cell cycle dynamics, we employed BrdU/EdU dual-labeling and calculated the total cell cycle time (Tc) (Bertacchi et al, 2020; Martynoga et al, 2005). *Akt* cTKO NPCs exhibited significantly longer Tc than controls (Fig. 5A,B), suggesting delayed progression through the cell cycle. FC analysis of PI-stained cells revealed a higher proportion of NPCs arrested in G_1_ phase in *Akt* cTKO tissues and cultured neurospheres (Fig. 5C,D; Appendix Fig. S5A,B), indicating G_1_ phase arrest.

We next assessed interkinetic nuclear migration (INM) using BrdU pulse-chase labeling (Ochoa et al, 2023; Taverna et al, 2014). During INM, the nucleus of a RGP migrates basally during G1, remains in the basal VZ during S phase, returns apically during G2, and completes mitosis at the apical surface (Kosodo, 2012). At 12 h post-injection, significantly fewer BrdU+ cells were detected in the basal VZ (R2) of *Akt* cTKO cortices, suggesting altered cell cycle progression (Fig. 5E–G; Appendix Fig. S5C,D). A 24-hour BrdU labeling experiment further revealed that fewer *Akt* cTKO NPCs completed a second mitotic cycle compared to controls (Fig. 5H,I; Appendix Fig. S5E). These findings demonstrate that AKT is essential for timely cell cycle progression and efficient INM in NPCs.

### Conditional deletion of AKT1/2/3 enhances CCND2 degradation via the ubiquitin- proteasome pathway

**Figure 6:**
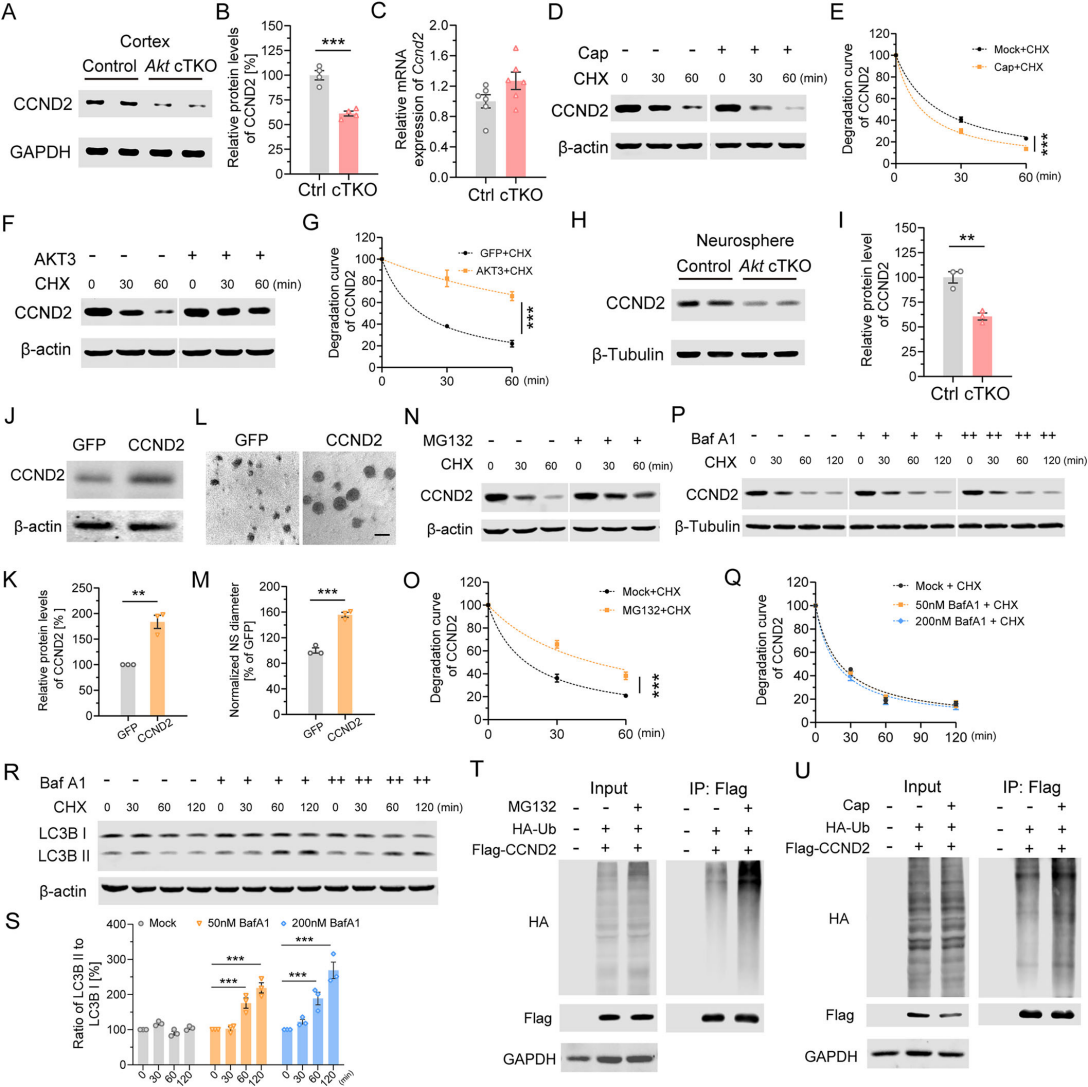
AKT inhibition promotes CCND2 degradation through UPS. (**A**, **B**) Western blot analysis of CCND2 in cortical lysates from control and *Akt* cTKO mice at E15.5. CCND2 protein levels were significantly reduced in the cortex of *Akt* cTKO mice compared to controls. GAPDH was used as a loading control. Data are shown as mean ± SEM (*n* = 4 mice per genotype, Student’s *t* test, ***P = 0.0003). (**C**) qRT-PCR analysis of *Ccnd2* mRNA levels. *Ccnd2* expression was comparable between control and *Akt* cTKO mice at E15.5. Data are shown as mean ± SEM (*n* = 6 mice per genotype, Student’s *t* test, *P* = 0.0903). (**D**) Time-course analysis of CCND2 stability. 293T cells were transfected with *Ccnd2* and treated with 5 μM Cap for 12 h. Protein was extracted at 0, 30 and 60 min after 50 μM cycloheximide (CHX) treatment and analyzed by Western blot using an anti- CCND2 antibody. (**E**) Quantification of CCND2 protein levels. Relative protein levels were normalized to 100% at time point 0. CCND2 degradation was significantly accelerated in Cap-treated group. Data are shown as mean ± SEM (*n* = 5 independent experiments, Two-way ANOVA, ***P = 7.970 × 10^−5^). (**F**) CCND2 stability analysis. 293T cells were transfected with *Ccnd2* and *Akt3* for 24 h, followed by treatment with 50 μM CHX. Cells were harvested at 0, 30 or 60 min after CHX addition and analyzed by Western blot with an anti- CCND2 antibody. (**G**) Quantification of CCND2 protein levels. CCND2 degradation was significantly inhibited in *Akt3*-transfected cells. Data are shown as mean ± SEM (*n* = 4 independent experiments, Two-way ANOVA, ***P = 1.471 × 10^−5^). (**H**, **I**) Western blot analysis of CCND2 in cultured neurospheres. CCND2 was significantly reduced in *Akt* cTKO neurospheres at DIV3 compared to controls. β-Tubulin served as a loading control. Data are shown as mean ± SEM (*n* = 3 independent experiments, Student’s *t* test, **P = 0.0043). (**J**, **K**) Western blot analysis for CCND2 in the *Akt* cTKO neurospheres overexpressed with CCND2. These cells were infected with lentivirus expressing CCND2 or GFP and BSD for 2 days. The infected cells were selected by 10 μg/ml blasticidin for 3 days during Passage 1 and then collected for Western blot. Levels of CCND2 were significantly increased in the neurospheres infected with lentivirus expressing CCND2. Data are shown as mean ± SEM (*n* = 3 independent experiments, Student’s *t* test, **P = 0.0028). (**L**, **M**) The neurosphere size. *Akt* cTKO neurospheres were infected with lentivirus expressing CCND2 or GFP and BSD for 2 days. These cells were then selected by blasticidin for 7 days during Passage 1. The diameter of *Akt* cTKO neurospheres infected with CCND2 was significantly increased compared to that infected with GFP. Data are shown as mean ± SEM (*n* = 3 independent experiments, Student’s *t* test, ***P = 0.0006). Scale bar: 100 μm. (**N**, **O**) Analysis of CCND2 protein degradation with proteasome inhibitor. 293 T cells were transfected with *Ccnd2* for 24 h and treated with 10 μM MG132 and 50 μM CHX. Cell lysates were prepared 0, 30 or 60 min after treatment, and CCND2 was detected by Western blot. β-actin served as a loading control. A protein degradation curve for CCND2 was plotted. CCND2 degradation was significantly inhibited in MG132/CHX-treated cells compared to CHX-treated cells. Data are shown as mean ± SEM (*n* = 3 independent experiments, Two-way ANOVA, ***P = 0.0002). (**P**, **Q**) Time-course analysis of CCND2 stability under autophagy inhibition. 293 T cells were transfected with *Ccnd2* for 24 h and treated with 50 nM or 200 nM Baf A1 and 50 μM CHX. Cell lysates were collected at 0, 30, 60 and 120 min after CHX/Baf A1 addition, and CCND2 levels was examined by Western blot. The CCND2 degradation curve was comparable between CHX-treated and CHX/Baf A1-treated cells. Data are shown as mean ± SEM (*n* = 3 independent experiments, Two-way ANOVA). (**R**, **S**) Western blot analysis for LC3B. 293 T cells were treated with Baf A1 and CHX, and cell lysates were prepared at 0, 30, 60 and 120 min after treatment. The ratio of LC3B II to LC3B I was higher in Baf A1-treated cells at 60 or 120 min compared to untreated cells. Data are shown as mean ± SEM (*n* = 3 independent experiments, One-way ANOVA, ***P = 6.126 × 10^−5^, 0 VS 60 min at 50 nM BafA1; *P* = 1.046 × 10^−7^, 0 VS 120 min at 50 nM BafA1; *P* = 7.220 × 10^−6^, 0 VS 60 min at 200 nM BafA1; *P* = 3.826 × 10^−10^, 0 VS 120 min at 200 nM BafA1). (**T**) CCND2 ubiquitination assay with proteasome inhibition. 293 T cells were transfected with Flag-tagged CCND2 and HA-tagged ubiquitin (Ub) and treated with 10 μM MG132 for 4 h. IP was performed using Flag antibody, and Flag-tagged CCND2 and HA-tagged Ub were detected by Western blot. HA-tagged Ub levels were higher in precipitated CCND2 in MG132-treated cells compared to untreated cells. (**U**) CCND2 ubiquitination assay with Akt inhibitor. 293 T cells were transfected with Flag-tagged CCND2 and HA-tagged Ub and treated with 5 μM Cap for 12 h. IP for Flag-tagged CCND2 was performed in the cell lysates. HA-tagged Ub and Flag-tagged CCND2 was detected by Western blot. HA-tagged Ub levels were higher in precipitated CCND2 from Cap-treated cells compared to untreated cells. Source data are available online for this figure.

Given the importance of CCND family proteins in cell cycle regulation and brain development (Herzinger and Reed, 1998; Lukas et al, 1995; Sicinska et al, 2003), we assessed the expression of CCND1, CCND2 and CCND3 in *Akt* cTKO mice. Western blot analysis of E15.5 cortical lysates revealed significantly reduced levels of CCND2 and CCND3 in *Akt* cTKO mice compared to controls (Fig. 6A,B; Appendix Fig. S6B,C), while CCND1 protein levels remained unchanged (Appendix Fig. S6A). We also performed qRT-PCR analysis to examine the transcripts of *Ccnd1/2/3*. Whereas mRNA levels of *Ccnd2* and *Ccnd3* were not altered in *Akt* cTKO mice compared to controls, those for *Ccnd1* were increased in *Akt* cTKO mice (Fig. 6C; Appendix Fig. S6D,E).

To investigate the mechanism underlying AKT-dependent regulation of the stability of CCND1/2/3, we performed a series of protein degradation assays. Treatment of 293 T cells with cycloheximide (CHX) to block protein synthesis revealed rapid degradation of CCND1/2/3, which was further accelerated by co-treatment with the AKT inhibitor Capivasertib (Cap) (Fig. 6D,E; Appendix Fig. S6F–I). Conversely, overexpression of AKT3 slowed CHX-induced degradation of CCND1/2/3 (Fig. 6F,G; Appendix Fig. S6J–M), indicating that AKT may stabilize CCND proteins. Since AKT deletion does not affect the transcription of *Ccnd2/3*, the reduction in protein levels of CCND2/3 may be due to accelerated degradation in *Akt* cTKO mice. It is quite likely that enhanced transcription of *Ccnd1* may compensate the accelerated degradation of CCND1 in *Akt* cTKO mice, leading to no significant change in CCND1 protein levels.

To determine whether CCND2 or CCND3 serves as the key mechanism for impaired proliferation in AKT-deficient NPCs, we performed rescue experiments using cultured *Akt* cTKO neurospheres. First, there was a significant reduction in CCND2/3 protein levels in *Akt* cTKO neurospheres compared to controls (Fig. 6H,I; Appendix Fig. S6N,O). Second, lentiviral-mediated of CCND2 effectively restored the size of *Akt* cTKO neurospheres (Fig. 6J–M). In contrast, CCND3 overexpression failed to increase the size of restore *Akt* cTKO neurospheres (Appendix Fig. S6P–S). These results suggest that CCND2 but not CCND3 may be the key mediator for AKT-dependent proliferation.

To delineate which degradation pathway may be involved, we treated CHX-exposed 293 T cells with either MG132, a proteasome inhibitor, or bafilomycin A1 (Baf A1), an autophagy-lysosome pathway inhibitor. MG132 treatment significantly attenuated CCND2 degradation (Fig. 6N,O), whereas Baf A1 had no detectable effect (Fig. 6P,Q), although it effectively blocked autophagic flux, as evidenced by increased LC3B II accumulation and LC3B II/I ratios (Fig. 6R,S). These results implicate the ubiquitin-proteasome pathway, rather than autophagy, in AKT-dependent CCND2 degradation.

We next examined whether AKT regulates CCND2 ubiquitination. Co-expression of HA-tagged ubiquitin and Flag-tagged CCND2 in 293T cells followed by MG132 treatment led to an accumulation of ubiquitinated CCND2 under both non-denaturing (Fig. 6T) and denaturing immunoprecipitation conditions (Appendix Fig. S6T). We did not detect ubiquitinated CDK4 in the immunoprecipitates of CCND2 (Appendix Fig. S6U). Notably, Cap treatment further increased CCND2 ubiquitination levels (Fig. 6U). Moreover, levels of ubiquitinated CCND2 was elevated in *Akt* cTKO neurospheres compared to controls (Appendix Fig. S6V). Therefore, AKT may suppresses CCND2 ubiquitination and subsequent proteasomal degradation.

### DDB1, a component of CRL4^AMBRA1^, is a direct substrate of AKT

**Figure 7:**
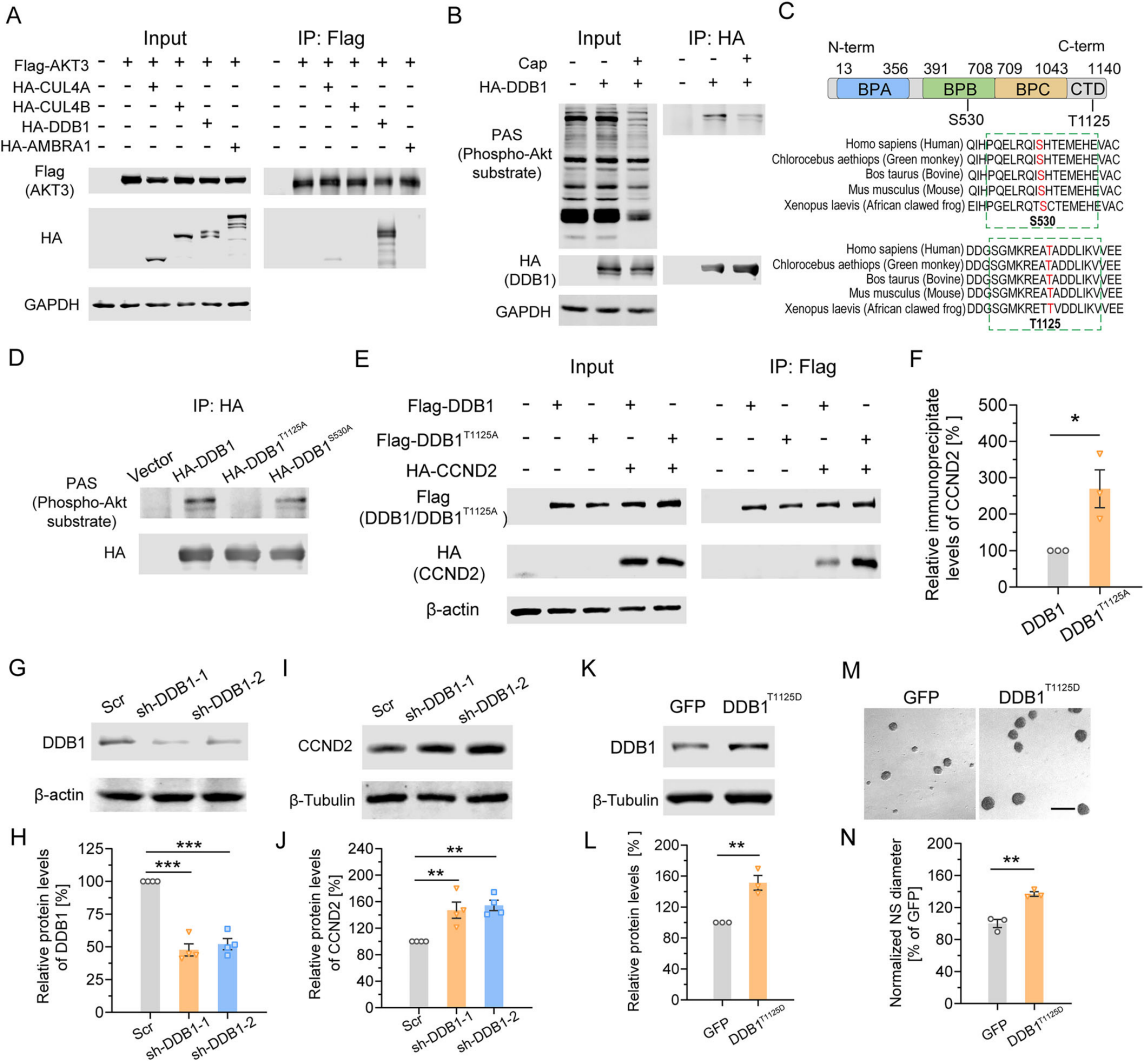
DDB1 is phosphorylated by AKT at the residue T1125. (**A**) Co-immunoprecipitation assay of AKT3 with CRL4^AMBRA1^ components. 293T cells were co-transfected with Flag-tagged AKT3 and HA-tagged versions of CUL4A, CUL4B, DDB1 and AMBRA1. Immunoprecipitation was performed using an anti-Flag antibody. Western blot exhibited that Flag-tagged AKT3 co-precipitated with HA-tagged CUL4A and DDB1, but not with CUL4B or AMBRA1. (**B**) IP assay using HA antibody in 293 T cells transfected with HA-tagged DDB1 and treated with 5 μM Cap for 12 h. The precipitated protein was detected by Western blot using PAS and HA antibodies. The results revealed that HA-tagged DDB1 was recognized by the PAS antibody, and the immune-reactivity of PAS was lower in Cap-treated samples compared to untreated samples. (**C**) The diagram of DDB1. S530 and T1125 of DDB1 were predicted as AKT-phosphorylated sites and these sites are conserved. BPA/BPB/BPC, β-propeller A-C, CTD C-terminal helical domain. (**D**) IP assay using HA antibody in the 293 T cells. These cells were transfected by HA-tagged DDB1, DDB1^T1125A^ or DDB1^S530A^. The precipitated proteins were analyzed by Western blot using PAS and HA antibodies. The results showed that HA-tagged DDB1 and DDB1^S530A^ were recognized by PAS antibody, whereas DDB1^T1125A^ was not. (**E**) Co-IP assay using Flag antibody in the 293 T cells. These cells were transfected by Flag-tagged DDB1 or DDB1^T1125A^ with HA-tagged CCND2. The precipitated protein was detected by Western blot using Flag and HA antibodies. More HA-tagged CCND2 was precipitated by Flag-tagged DDB1^T1125A^ than Flag-tagged DDB1. (**F**) Relative immunoprecipitate levels of CCND2 to precipitated DDB1/DDB1^T1125A^. Levels of CCND2 precipitated by DDB1^T1125A^ were significantly higher than those by DDB1. Data are shown as mean ± SEM (*n* = 3 independent experiments, Student’s *t* test, *P = 0.0312). (**G**) Knockdown of DDB1 by shRNAs. 293T cells were transfected by shRNA-1 (sh1) or shRNA-2 (sh2) targeting DDB1 for 72 h. Scramble (Scr) RNA served as a control. (**H**) Quantification of DDB1 protein expression. Levels of DDB1 were significantly reduced in the 293T cells transfected with sh1 or sh2. Data are shown as mean ± SEM (*n* = 4 independent experiments, One-way ANOVA, ***P = 8.193 × 10^−6^ for sh-DDB1-1, *P* = 1.709 × 10^−5^ for sh-DDB1-2). (**I**, **J**) Western blot analysis for CCND2. 293 T cells were transfected by sh1 or sh2 with CCND2 for 72 h. CCND2 expression was then examined by Western blot. Levels of CCND2 were significantly increased in the cells transfected with sh1 or sh2. Data are shown as mean ± SEM (*n* = 4 independent experiments, One-way ANOVA, **P = 0.0080 for sh-DDB1-1, *P* = 0.0033 for sh-DDB1-2). (**K**, **L**) Overexpression of the DDB1^T1125D^ in *Akt* cTKO neurospheres. These cells were infected with lentivirus expressing DDB1^T1125D^ or GFP, selected with 10 μg/ml blasticidin for 3 days and analyzed by Western blot. Data are shown as mean ± SEM (*n* = 3 independent experiments, Student’s *t* test, **P = 0.0056). (**M**, **N**) Rescue of neurosphere size by DDB1^T1125D^. *Akt* cTKO neurospheres infected with lentivirus expressing DDB1^T1125D^ or GFP were selected with blasticidin and imaged. The diameter of *Akt* cTKO neurospheres infected with DDB1^T1125D^ was significantly increased compared to controls. Scale bar: 100 μm. Data are shown as mean ± SEM (*n* = 3 independent experiments, Student’s *t* test, **P = 0.0032). Source data are available online for this figure.

CRL4^AMBRA1^, an E3 ubiquitin ligase complex composed of RBX1, CUL4A/4B, DDB1, and AMBRA1, has been implicated in CCND2 ubiquitination (Chaikovsky et al, 2021; Maiani et al, 2021; Simoneschi et al, 2021). Using SCANSITE4.0 (Obenauer et al, 2003), we identified putative AKT phosphorylation motifs within CUL4A/B, DDB1, and AMBRA1 (Appendix Fig. S7A). Co-immunoprecipitation (Co-IP) assays showed that AKT selectively interacted with CUL4A and DDB1, but not with CUL4B or AMBRA1 (Fig. 7A). To determine whether these proteins are phosphorylated by AKT, we immunoprecipitated CUL4A and DDB1 and probed for phosphorylated AKT substrates using a phospho-AKT substrate (PAS) antibody (Rogerson et al, 2021). While CUL4A showed no PAS signal (Appendix Fig. S7B), DDB1 displayed a prominent PAS-positive band (Fig. 7B), which was diminished following Cap treatment, confirming that DDB1 is a phosphorylation target of AKT.

To pinpoint the specific phosphorylation site, we generated alanine substitution mutants at two predicted sites on DDB1, S530A and T1125A (Fig. 7C). PAS immunoblotting revealed loss of signal in the DDB1^T1125A^ mutant, but not in DDB1^S530A^ (Fig. 7D), identifying T1125 as the AKT-targeted residue. To investigate the impact of AKT-mediated phosphorylation on DDB1, we first generated modeled structure of DDB1^T1125D^, a phospho-mimetic mutant of DDB1, using SWISS-MODEL (Waterhouse et al, 2018). Structural prediction analysis shows that T1125 may form ten hydrogen bonds with residues in the C-terminal α-helix, and that D1125 forms two (Appendix Fig. S7C), suggesting that the phosphorylation at T1125 may destabilize DDB1.

We next assessed whether the phosphorylation of DDB1 at T1125 affects its interaction with other CRL4^AMBRA1^ components and CCND2. Co-IP assays showed that DDB1^T1125A^, a non-phosphorylatable DDB1 mutant, was precipitated with significantly more CUL4A or AMBRA1 than WT DDB1 (Appendix Fig. S7D–G). Therefore, the phosphorylation at T1125 inhibited the assembly of CRL4^AMBRA1^. In addition, we found that the DDB1^T1125A^ mutant exhibited enhanced binding to CCND2 compared to the WT DDB1 (Fig. 7E,F), suggesting that AKT-mediated phosphorylation of DDB1 attenuates its interaction with CCND2, thereby limiting ubiquitination. Finally, we tested whether DDB1 regulates CCND2 protein levels. shRNA-mediated knockdown of DDB1 in 293 T cells led to a substantial reduction in DDB1 protein (Fig. 7G,H) and a concomitant increase in CCND2 levels (Fig. 7I,J), supporting a model in which DDB1 negatively regulates CCND2 stability downstream of AKT.

To verify the importance of the phosphorylation of DDB1 in AKT-dependent NPC proliferation, *Akt* cTKO neurospheres were infected with lentivirus expressing DDB1^T1125D^. Western blot showed significant expression of DDB1^T1125D^ in neurospheres (Fig. 7K,L). Moreover, we found that DDB1^T1125D^ overexpression caused significantly increased the size of *Akt* cTKO neurosphere (Fig. 7M,N). Given the role of DDB1 in the CRL4^AMBRA1^ complex, *Akt* cTKO neurospheres were treated with 0.1 µM MLN4924 (Simoneschi et al, 2021), a cullin-RING E3 ligase inhibitor that can target CRL4^AMBRA1^. Our results showed that MLN4924 resulted in increased CCND2 protein expression (Appendix Fig. S7H,I) and partially restored neurosphere size (Appendix Fig. S7J,K). We further examined the effect of MLN4924 on apoptosis. We found that MLN4924 at 1 µM and 10 µM but not 0.1 µM induced increased numbers of TUNEL+ cells in cultured neurospheres (Appendix Fig. S7L,M), excluding the possibility that the restoration of *Akt* cTKO neurosphere size by 0.1 µM MLN4924 is due to abnormal apoptosis. These findings indicate that AKT may promote NPC proliferation through inhibiting the activity of the CRL4^AMBRA1^ complex.

## Discussion

The proliferation of neural progenitor cells is the foundation for neurogenesis and brain expansion. Recent clinical studies have linked mutations in *AKT* to brain malformations (Boland et al, 2007; Thierry et al, 2012), yet the underlying cellular and molecular mechanisms have remained elusive. To elucidate how AKT regulates cortical development, we employed a comprehensive combination of in vitro and in vivo approaches. By generating conditional triple knockout (*Akt* cTKO) mice using *Emx1-Cre* and *Akt1*^*f/f*^*;Akt2*^*-/-*^*;Akt3*^*f/f*^, we selectively ablated all three AKT isoforms in NPCs of the dorsal telencephalon. Our findings demonstrate that AKT deficiency leads to a marked reduction in both RGPs and IPs, accompanied by a prolonged cell cycle and diminished NPC proliferation. We further identify the CRL4^AMBRA1^-CCND2 axis as a key downstream pathway through which AKT promotes cortical expansion.

Multiple lines of evidence support the essential role of AKT in NPC proliferation and cortical growth. First, we observed a significant decrease in the number of BrdU+ and PH3+ cells in the VZ of *Akt* cTKO embryos at E13.5 and E15.5. Second, flow cytometric analysis revealed an increased proportion of NPCs in G_1_ phase in *Akt* cTKO cortices. Third, BrdU/EdU double-labeling assays confirmed that NPCs in *Akt* cTKO mice exhibit a prolonged Tc, particularly in G_1_. Notably, we did not detect premature neurogenesis or increased apoptosis in *Akt* cTKO cortices, ruling out cell death as a major contributor to the observed microcephaly. Given that the size of the NPC pool during development is a critical determinant of brain size (Guerrini and Dobyns, 2014; Raybaud and Widjaja, 2011), the impaired proliferation in *Akt* cTKO mice likely underlies the reduction in cortical mass.

The extended G_1_ phase in *Akt* cTKO NPCs suggests that defective cell cycle progression contributes to reduced proliferative capacity. Supporting this interpretation, our RNA-seq data revealed that differentially expressed genes in *Akt* cTKO cortices are significantly enriched in pathways related to cell cycle control and G_1_-S transition. These findings are in line with previous work showing that insulin-like growth factor 1 (IGF-1), an upstream activator of AKT, shortens G_1_ (Mairet-Coello et al, 2009) and promotes NPC proliferation (Hodge et al, 2004). Together, our results position AKT as a critical regulator of cell cycle kinetics in neural progenitors.

To uncover the molecular mechanisms by which AKT modulates NPC proliferation, we investigated its regulation of CCND2, a G_1_-phase cyclin essential for cortical development. Several lines of evidence highlight CCND2 as a key AKT target: (1) human *CCND2* mutations are associated with brain malformations (Cappuccio et al, 2019; Mirzaa et al, 2014); (2) *Akt* cTKO cortices display reduced CCND2 protein levels, while *Ccnd2* mRNA remains unchanged; and (3) time-course degradation assays reveal that CCND2 protein turnover is accelerated upon AKT inhibition and stabilized by AKT3 overexpression. These findings indicate that AKT regulates CCND2 expression post-translationally by enhancing its stability.

Cyclin D proteins are induced by mitogens, accumulate during G_1_ phase, and are rapidly degraded thereafter (Gookin et al, 2017; Pagano et al, 1994; Sherr, 1995). This degradation is mediated by the E3 ubiquitin ligase CRL4^AMBRA1^ (Chaikovsky et al, 2021; Maiani et al, 2021; Simoneschi et al, 2021), but the regulatory mechanisms governing its activity have remained unclear. Since phosphorylation is a known mode of E3 ligase regulation, we explored whether AKT modulates CRL4^AMBRA1^ activity via phosphorylation of its subunits. Our data identify DDB1, a key scaffold of the CRL4^AMBRA1^ complex, as a direct substrate of AKT. Specifically, we show that AKT phosphorylates DDB1 at threonine 1125. Mutation of this residue to alanine (DDB1^T1125A^) increases DDB1’s affinity for CCND2 and enhances its ubiquitination, suggesting that phosphorylation of DDB1 by AKT inhibits CCND2 degradation. Importantly, knockdown of DDB1 elevates CCND2 protein levels, supporting a model in which DDB1 negatively regulates CCND2 stability. Notably, *DDB1* mutations have been implicated in human neurodevelopmental disease (White et al, 2021). Taken together, our findings suggest that AKT-mediated phosphorylation of DDB1 inhibits CCND2 ubiquitination, thereby promoting NPC proliferation and cortical expansion.

While the present study demonstrates that genetic or pharmacological inhibition of AKT suppresses NPC proliferation, recent evidence indicates that AKT inhibitors can synergize with temozolomide to inhibit glioblastoma growth (Cui et al, 2023). Glioblastoma is the most common primary brain tumor, and its malignancy is driven mainly by glioblastoma stem cells (GSCs) (Lathia et al, 2015). Notably, GSCs and NPCs share several fundamental cellular properties, including self-renewal capacity and the ability to differentiate into various lineages (Deng et al, 2025; Gotz and Huttner, 2005; Zhu et al, 2014). Although AKT inactivation markedly reduces the NPC population, a substantial number of NPCs retain proliferative capacity both in vivo and in vitro, suggesting that additional signaling pathways contribute to the maintenance of the self-renewal in AKT-deficient NPCs. Identifying these compensatory pathways in future studies may uncover novel therapeutic targets relevant to both neurodevelopmental disorders and brain tumors.

In conclusion, this study reveals a novel molecular mechanism by which AKT promotes NPC proliferation and cortical expansion. We propose that in NPCs, AKT phosphorylates DDB1, thereby impairing CRL4^AMBRA1^-mediated ubiquitination of CCND2. AKT deficiency leads to increased CRL4^AMBRA1^ activity, accelerated CCND2 degradation, prolonged G_1_ phase, and impaired NPC proliferation, leading to microcephaly. Our findings not only clarify how AKT regulates cortical development but also provide a unifying molecular framework for understanding a subset of brain malformations.

## Methods

**Table:** Reagents and tools table

Reagent/resource	Reference or source	Identifier or catalog number
**Experimental models**		
*Akt1* ^*f/f*^ *;Akt2* ^*-/-*^ *;Akt3* ^*f/f*^	Wang et al, 2021a	N/A
*Emx1-Cre*	Cheng et al, 2019	N/A
*mTmG*	Muzumdar et al, 2007	N/A
*Akt1* ^*f/f*^ *;Akt2* ^*-/-*^ *;Akt3* ^*f/f*^ *; Emx1-Cre*	This study	N/A
*Emx1-Cre; mTmG*	This study	N/A
HEK 293 T	ATCC	Cat# CRL-3216
ENSA	Li et al, 2022	N/A
**Recombinant DNA**		
pLVX-HA-CCND1-BSD	This study	N/A
pLVX-HA-CCND2-BSD	This study	N/A
pLVX-HA-CCND3-BSD	This study	N/A
pLVX-GFP-BSD	This study	N/A
pLVX-HA-AKT3 (human)-BSD	This study	N/A
pLVX-HA-AKT3^T288I^ (human)-BSD	This study	N/A
pLVX-Flag-CCND2-BSD	This study	N/A
pLVX-Flag-DDB1-BSD	This study	N/A
pLVX-Flag-DDB1^T1125A^ -BSD	This study	N/A
pLVX-Flag-DDB1^T1125D^ -BSD	This study	N/A
pLVX-Flag-Akt3 (mouse)- BSD	Wang et al, 2021a	N/A
pLKO.1-DDB1-shRNA-1	This study	N/A
pLKO.1-DDB1-shRNA-2	This study	N/A
pcDNA5-HA-CUL4A	This study	N/A
pcDNA5-HA-CUL4B	This study	N/A
pcDNA5-HA-DDB1	This study	N/A
pcDNA5-HA-DDB1 ^S530A^	This study	N/A
pcDNA5-HA-DDB1 ^T1125A^	This study	N/A
pcDNA5-HA-AMBRA1	This study	N/A
**Antibodies**		
Rabbit monoclonal anti-pan-AKT	Cell Signaling Technology	Cat# 4691, RRID:AB_915783
Rabbit monoclonal anti-phospho-AKT (S473)	Cell Signaling Technology	Cat# 4060, RRID:AB_2315049
Rat monoclonal anti-CTIP2	Abcam	Cat# ab18465, RRID:AB_2064130
Rabbit monoclonal anti-TBR1	Abcam	Cat# ab31940, RRID:AB_2200219
Mouse monoclonal anti-GAPDH	CW Biotech	Cat# cw0100M, RRID:AB_2801390
Rat monoclonal anti-BrdU	Abcam	Cat# ab6326, RRID:AB_305426
Rabbit polyclonal anti-Ki67	Abcam	Cat# ab15580, RRID:AB_443209
Rabbit polyclonal phospho-AKT substrates (PAS)	Cell Signaling Technology	Cat# 9611, RRID:AB_330302
Mouse monoclonal anti-β-actin	CW Biotech	Cat# CW0096, RRID:AB_2665433
Mouse monoclonal anti-SOX2	Santa Cruz	Cat# sc-365823, RRID:AB_10842165
Rabbit polyclonal anti-PAX6	Biolegend	Cat# 901301, RRID:AB_2565003
Rabbit polyclonal anti-TBR2	Abcam	Cat# ab23345, RRID:AB_778267
Mouse monoclonal phospho-Histone H3 (S10)	Cell Signaling Technology	Cat# 9706, RRID:AB_331748
Rabbit monoclonal anti-Cyclin D2	Cell Signaling Technology	Cat# 3741 T, RRID:AB_2070685
Mouse monoclonal anti-Cyclin D3	Abcam	Cat# ab289546, RRID:N/A
Rabbit monoclonal anti-CyclinD1	Abclonal	Cat# A19038, RRID:AB_2862530
Rabbit polyclonal anti-DDB1	Abclonal	Cat#A2896, RRID:AB_2764716
Mouse monoclonal anti-DYKDDDDK tag	Proteintech	Cat# 66008-4-Ig, RRID: AB_2918475
Mouse monoclonal anti-HA tag	Proteintech	Cat# 66006-2-Ig, RRID:AB_2881490
Rabbit polyclonal anti-HA tag	Abcam	Cat# ab9110, RRID: AB_307019
Rabbit monoclonal anti-LC3B	Abcam	Cat# ab9110, RRID: AB_307019
Mouse monoclonal anti-β-Tubulin	CW Biotech	Cat# CW0098, RRID:AB_2814800
Goat anti-Rabbit IgG, IRDye® 800CW Conjugated antibody	LI-COR Biosciences	Cat# 926-32211, RRID:AB_621843
Goat anti-Mouse IgG, IRDye® 800CW Conjugated antibody	LI-COR Biosciences	Cat# 926-32210, RRID:AB_621842
Goat anti-Mouse IgG,IRDye® 680RD Conjugated antibody	LI-COR Biosciences	Cat# 926-68070, RRID:AB_10956588
Goat Anti-Rabbit IgG,IRDye® 680RD Conjugated antibody	LI-COR Biosciences	Cat# 926-68071, RRID:AB_10956166
Cy3-Goat anti-Mouse IgG (H + L)	Jackson ImmunoResearch Labs	Cat# 115-165-166, RRID: AB_2338692
Alexa Fluor 647-Goat anti-Rat IgG (H + L)	Jackson ImmunoResearch Labs	Cat# 112-605-167, RRID: AB_2338404
Cy3-Donkey anti-Goat IgG (H + L)	Jackson ImmunoResearch Labs	Cat# 705-165-147, RRID:AB_2307351
Alexa Fluor 488-Goat anti-Rabbit IgG (H + L)	Jackson ImmunoResearch Labs	Cat# 111-545-003, RRID:AB_2338046
Alexa Fluor 488-Donkey anti-Mouse IgG (H + L)	Jackson ImmunoResearch Labs	Cat# 715-545-150, RRID: AB_2340846
**Oligonucleotides and other sequence-based reagents**		
Primer for qRT-PCR		
*Akt1-F:*CAGACTGTGGCAGATGGACTC	This study	N/A
*Akt1-R:*AAACTCGTTCATGGTCACACGG	This study	N/A
*Akt3-F:*AAACAGAACGACCAAAGCC	This study	N/A
*Akt3-R:*CGTCCACTCTTCTCTTTCCT	This study	N/A
*Ccnd1-F:* CGTGGCCTCTAAGATGAAGGA	This study	N/A
*Ccnd1-R:* AGTTCCATTTGCAGCAGCTC	This study	N/A
*Ccnd2-F:*GACCCATCTTCAGCTCCTGG	This study	N/A
*Ccnd2-R:*CTACCAGTTCCCACTCCAGC	This study	N/A
*Ccnd3-F:* CGAGCCTCCTACTTCCAGTG	This study	N/A
*Ccnd3-R:* GGACAGGTAGCGATCCAGGT	This study	N/A
*Gapdh-F:*GAGTGTTTCCTCGTCCCGT	This study	N/A
*Gapdh-R:*ACAATCTCCACTTTGCCACTG	This study	N/A
Primer for plasmid construction		
*Ccnd1-F:* ATGGAACACCAGCTCCTGTG	This study	N/A
*Ccnd1-R:* TCAGATGTCCACATCTCGCAC	This study	N/A
*Ccnd2-F:*ATGGAGCTGCTGTGCTGCGAG	This study	N/A
*Ccnd2-R:*TCACAGGTCAACATCCCGCACG	This study	N/A
*Ccnd3-F:* ATGGAGCTGCTGTGTTGCGAG	This study	N/A
*Ccnd3-F:* GGCCTGTCTCAAGCTACAGGTG	This study	N/A
*Cul4a-F:*ACAGCAGCAGGAGGAGGAG	This study	N/A
*Cul4a-R:*TGCCACGTAGTGGTACTGATTTG	This study	N/A
*Cul4b-F:*ATGTCACGTTCAACTAGGTCT	This study	N/A
*Cul4b-R:*TGCAATATAGTTGTACTGATTTGGATTTTC	This study	N/A
*Ddb1-F:*ATGTCGTACAACTACGTCGT	This study	N/A
*Ddb1-R:*ATGGATCCGAGTTAGCTCCTC	This study	N/A
*Ambra1-F:*ATGAAAGTTGTCCCAGAGAAGAATG	This study	N/A
*Ambra1-R:*TCGGTTCTGTGGCTCTCC	This study	N/A
*AKT3-F:*ATGAGCGATGTTACCATTGTG	This study	N/A
*AKT3-R:*TTATTCTCGTCCACTTGCAGAG	This study	N/A
*GFP-F:* ATGGTGAGCAAGGGCGAGGA	This study	N/A
*GFP-R:* TTACTTGTACAGCTCGTCCA	This study	N/A
*Ddb1-T1125A-F:* GGGAGGCAGCTGCAGATGACCTCATCAAAGTCGTG	This study	N/A
*Ddb1-T1125A-R:* ATCTGCAGCTGCCTCCCGCTTCATACCACTGC	This study	N/A
*Ddb1-T1125D-F:* CGGGAGGCAGATGCAGATGACCTCATCAAAGTCGTGG	This study	N/A
*Ddb1-T1125D-R:* TCATCTGCATCTGCCTCCCGCTTCATACCACTGCC	This study	N/A
*Ddb1-S530A-F:* GGCAAATCGCCCACACAGAGATGGAACATGAAGTGGC	This study	N/A
*Ddb1-S530A-R:* CTGTGTGGGCGATTTGCCGGAGCTCCTGAGGGTG	This study	N/A
*AKT3-T288I-F:* ATAAAAATTATAGATTTTGGACTTTGCAAAGAAGGGATC	This study	N/A
*AKT3-T288I-R:* TCCAAAATCTATAATTTTTATGTGGCCATCTTTGTCCAGC	This study	N/A
*DDB1-shRNA1-F:*CCGGTCCACTAGATCGCGATAATAACTCGAGTTATTATCGCGATCTAGTGGATTTTTG	This study	N/A
*DDB1-shRNA1-R:*AATTCAAAAATCCACTAGATCGCGATAATAACTCGAGTTATTATCGCGATCTAGTGGA	This study	N/A
*DDB1-shRNA2-F:*CCGGCGACTCAATAAAGTCATCAAACTCGAGTTTGATGACTTTATTGAGTCGTTTTTG	This study	N/A
*DDB1-shRNA2-R:*AATTCAAAAACGACTCAATAAAGTCATCAAACTCGAGTTTGATGACTTTATTGAGTCG	This study	N/A
**Chemicals, enzymes and other reagents**		
DAPI	Sigma-Aldrich	Cat# D9542
BrdU	Sigma-Aldrich	Cat# B5002
EdU	Sigma-Aldrich	Cat# 900584
Carboxyfluorescein diacetate succinimidyl ester (CFSE)	Invitrogen	Cat# 65-0850-85
Capivasertib	MedChemExpress	Cat# HY-U00434
MK 2206	MedChemExpress	Cat# HY-10358
Propidium iodide (PI)	Yeasen	Cat# 40711ES10
Cycloheximide (CHX)	Selleck	Cat# S7418
Basic FGF	Peprotech	Cat# 100-18B
EGF	R&D	Cat# 236-EG-01M
Penicillin-streptomycin	Gibco	Cat# 15140-122
Lipofectamine 2000	Invitrogen	Cat# 11668019
Lipofectamine 3000	Invitrogen	Cat# L3000015
Ribonuclease A (RNase A)	Yeasen	Cat# 10407ES60
B27	Gibco	Cat# 17504-044
PrimeScript RT Reagent Kit	Takara	Cat# RR047A
TUNEL assay	Vazyme	Cat# A112
BCA Protein Assay kit	GenStar	Cat# E162
**Software**		
GraphPad Prism 8.0.2	https://www.graphpad.com/	N/A
ImageJ	https://imagej.net/	N/A
PyMOL(TM) 3.1.4.1	https://www.pymol.org/	N/A
ModFit LT 3.1	https://www.vsh.com/	N/A
Image Studio Ver 5.2	https://www.licorbio.com/	N/A
StepOne Software v2.3	https://www.thermofisher.cn/	N/A
ZEISS ZEN 2.1 SP2	https://zeiss.com.cn/	N/A
Leica LAS AF Lite 2.6.0	https://www.leica-microsystems.com.cn/	N/A
cellSens Standard 1.4.1	https://www.olympuschina.com/	N/A
**Other**		
Illumina NovaSeq 6000	Illumina	N/A

### Animals

*Akt1*^*f/f*^*;Akt2*^*-/-*^*;Akt3*^*f/f*^ and *Emx1-Cre* mice were previously reported by us (Cheng et al, 2019; Wang et al, 2021a; Xu et al, 2017). To generate *Akt1*^*f/f*^*;Akt2*^*-/-*^*;Akt3*^*f/f*^*;Emx1-Cre* mice, *Akt1*^*f/f*^*;Akt2*^*-/-*^*;Akt3*^*f/f*^ were bred with *Emx1-Cre* to obtain *Akt1*^*f/+*^*;Akt2*^*+/-*^*;Akt3*^*f/+*^*;Emx1-Cre*. The *mTmG* reporter mouse was described previously (Muzumdar et al, 2007). All mice used in this study had a C57BL/6 genetic background and were housed in a specific pathogen-free (SPF) facility at the Model Animal Research Center of Nanjing University. The animal room was maintained at a temperature of 25 ± 1 °C with a 12-h light/dark cycle. Mice were provided with ad libitum access to food and water. Mouse breeding was conducted in strict compliance with an animal protocol approved by the Institutional Animal Care and Use Committee of Nanjing University. All the mouse experiments were performed in accordance with the Guide for the Care and Use of Laboratory Animals of Nanjing University.

### Neurosphere culture

1. The dorsal cortices of E15.5 mice were dissected, and the meninges were removed in cold DMEM medium.
2. The cortices were then mechanically triturated using pipette tips in DMEM/F12 medium supplemented with 2% B27, 20 ng/ml bFGF, 20 ng/ml EGF and 1% penicillin-streptomycin.
3. The cells were filtered through a 40-μm cell strainer to obtain a single-cell suspension, which was seeded in DMEM/F12 medium at a density of 4 × 10^4^/cm^2^.
4. After 1 day of culture, the cells were treated with either 1 μM MK2206, 1 μM capivasertib or 0.1 μM MLN4924 for 6 days.
5. Neurosphere images were captured to measure their diameters.

For NPC culture, cells were plated onto coverslips coated with 15 μg/ml poly-L-ornithine and 10 μg/ml laminin in DMEM/F12 medium for 48 h. All cells were maintained in an incubator at 37 °C with 5% CO_2_.

### Constructs of plasmids

Mouse *Ccnd1*, *Ccnd2*, *Ccnd3*, *Cul4a*, *Cul4b*, *Ddb1*, *Ambra1*, and human *AKT3* were amplified and cloned into pcDNA5-HA, pLVX-HA or pLVX-Flag vector. DDB1^S530A^, DDB1^T1125A^, DDB1^T1125D^ and AKT3^T288I^ mutants were generated following the manufacturer’s protocol (Vazyme, Cat#C214). *DDB1*-targeting shRNAs were designed and cloned into pLKO.1 vector. The primers used for constructing these plasmids are listed in the Reagents and Tools Table.

### Cell culture and transfection

HEK 293T cells (ATCC, Cat# CRL-3216) were cultured in DMEM supplemented with 10% fetal bovine serum (FBS) and 1% penicillin- streptomycin. After 24 h of seeding, the cells were transfected with a vector carrying *Akt3* (Wang et al, 2021a), *Ccnd1*, *Ccnd2*, *Ccnd3*, *AKT3*, *AKT3* mutants, *Cul4a*, *Cul4b*, *Ddb1*, *Ddb1* mutants or *Ambra1* using the Lipofectamine 2000 transfection reagent following the manufacturer’s protocol. Human NSC ENSA cells were maintained in neurobasal media supplemented with 2% B27, 20 ng/ml EGF and 20 ng/ml bFGF. These cells were maintained in an incubator at 37 °C with 5% CO_2_.

### Lentivirus generation

pLVX-HA-GFP-BSD, pLVX-HA-CCND2-BSD, pLVX-HA-CCND3-BSD, pLVX-DDB1^T1125D^-BSD, pLVX-HA-AKT3-BSD and pLVX-HA-AKT3^T288I^-BSD were constructed. These lentiviral expressing vectors and packaging vectors pMD2.G and psPAX2 were co-transfected into 293T cells by Lipofectamine 2000 to generate lentivirus. Viral supernatants were collected and filtered through 0.45 mm filter 48 h after transfection. The filtered supernatants were then concentrated by PEG-8000 solutions and centrifuged at 4000 rpm for 20 min at 4 °C. Neurospheres were infected at multiplicity of infection (MOI) of 5, and the infected neurospheres were selected by 10 μg/ml blasticidin.

### CSFE labeling

Cells were isolated from embryonic cortices. CFSE was added in the culture medium at a concentration of 5 μM, 3 h after cells seeding. Cells were then incubated at 37 °C for 15 min. Following this, they were washed and resuspended in fresh medium. After 5 days of culture, cells were digested using trypsin and triturated to obtain a single-cell suspension. Fluorescence intensity was measured using a BD LSRFortessa flow cytometer, with 10,000 events recorded by the FACSDiva system. Data analysis was performed using FlowJo software.

### Cell cycle analysis by flow cytometry

1. Neurospheres were cultured, trypsinized, triturated and filtered through a 40-μm cell strainer to obtain a single-cell suspension in PBS at DIV3. The dorsal cortices of E13.5 mice were dissected, and their meninges were carefully removed. The cortices were then digested with 0.25% trypsin at 37 °C for 5 min and triturated to form a single-cell suspension in DMEM medium supplemented with 10% FBS.
2. The cells were washed with PBS and filtered through a 40-μm cell strainer.
3. The resulting suspension was dropwise added to ice-cold 70% ethanol and fixed overnight at 4 °C.
4. Following fixation, the cells were washed with PBS, treated with 100 μg/ml RNase A for 15 min at 37 °C.
5. These cells then stained with 50 μg/ml propidium iodine (PI) for 15 min at room temperature (RT).
6. Throughout the process, cells were protected from light. Fluorescence intensity was measured using the BD LSRFortessa flow cytometer, and data were analyzed with ModFit LT software.

### Nissl staining

Paraffin-embedded embryonic brain sections were deparaffinized with xylene and rehydrated through a graded ethanol series. The sections were then stained with 0.1% cresyl violet for 1 min, air-dried and mounted with neutral resin. Images were acquired using a BX53 microscope (Olympus). Cortical thickness was quantified using ImageJ by measuring the distance from the pial surface to the ventricular surface at the top, middle, and bottom of the dorsal cortex. These three measurements were averaged for each section. A minimum of four embryos were analyzed per genotype.

### Immunohistochemistry (IHC)

Embryos at several given stages were dissected and fixed in 4% paraformaldehyde (PFA) in PBS at 4 °C for 2 h. The brains were then dehydrated in 30% sucrose in PBS at 4 °C overnight. Frozen sections, 10μm in thickness, were dried at 37 °C for 30 min and washed in PBS. For antigen retrieval, the sections were boiled in 0.01 M sodium citrate buffer solution (pH 6.0) for 20 min, and allowed to cool. After blocking with 5% BSA for 30 min, the sections were incubated with primary antibodies at 4 °C overnight, followed by incubation with the secondary antibodies at room temperature for 30 min. The primary antibody concentrations were as follows: anti-AKT (Cell Signaling Technology, Cat# 4691, 1:500), anti-SOX2 (Santa Cruz, Cat# sc-365823, 1:200), anti-PAX6 (Biolegend, Cat# 901301, 1:500), anti-TBR2 (Abcam, Cat# ab23345, 1:1000), anti-CTIP2 (Abcam, Cat# ab18465, 1:500), anti-TBR1 (Abcam, Cat# ab31940, 1:1000), anti-BrdU (Abcam, Cat# ab6326, 1:500), anti-PH3 (Cell Signaling Technology, Cat# 9706, 1:500) and anti-KI67 (Abcam, Cat# ab15580, 1:500). Nuclei were counterstained with 1 μg/ml DAPI. Images were captured using a ZEISS LSM880 laser confocal microscope or an Olympus BX53 fluorescence microscope.

For Immunocytochemistry, cells were fixed with 4% PFA for 15 min at RT, and then permeabilized with 0.1% Triton X-100 in PBS for 10 min. After blocking with 5% BSA, cells were incubated with primary antibodies at 4 °C overnight, followed by incubation with the secondary antibodies at RT for 30 min. The primary antibodies were used at the following concentrations: anti-AKT (Cell Signaling Technology, Cat# 4691, 1:500), anti-KI67 (Abcam, Cat# ab15580, 1:500) and anti-SOX2 (Santa Cruz, Cat# sc-365823, 1:200). Images were acquired using a Leica SP5 laser confocal microscope. Detailed information about the antibodies was listed in the Reagents and Tools Table.

### Western blot

To prepare protein samples, the dorsal cortices from embryos of various ages were dissected and the meninges were removed in cold PBS. The cortical tissues were homogenized in cold radio-immunoprecipitation assay (RIPA) lysis buffer supplemented with protease and phosphatase inhibitors. The lysates were then centrifuged at 12,000 × *g* for 20 min. Protein concentration was analyzed with a BCA Protein Assay kit (GenStar, Cat#E162). Normalized samples (20 μg of total proteins) were loaded in 10% SDS-PAGE gels and transferred to nitrocellulose membrane. After blocking with 5% (w/v) nonfat dry milk at RT for 1 h, the membranes were incubated with primary antibodies at 4 °C overnight, followed by incubation with secondary antibodies conjugated to infrared dye at RT for 45 min. The primary antibody concentrations were as follows: anti-AKT (Cell Signaling Technology, Cat# 4691, 1:1000), anti-PAS (Cell Signaling Technology, Cat# 9611, 1:1000), anti-pAKT^S473^ (Cell Signaling Technology, Cat# 4060, 1:1000), anti-CCND1 (Abclonal, Cat# A19038, 1:1000), anti-CCND2 (Cell Signaling Technology, Cat# 3741T, 1:1000), anti-CCND3 (Abcam, Cat# ab289546, 1:1000), anti-DDB1 (Abclonal, Cat#A2896, 1:1000), anti-LC3B (Abcam, Cat# ab9110, 1:1000), anti-HA (Abcam, Cat# ab9110, 1:2000), anti-Flag (Proteintech, Cat# 66008-4-Ig, 1:2000), anti-GAPDH (CW Biotech, Cat# cw0100M, 1:2000), anti-β-actin (CW Biotech, Cat# CW0096, 1:2000), and anti-β-Tubulin (CW Biotech, Cat# CW0098, 1:2000). The membranes were scanned and analyzed using the Odyssey Infrared Imaging System (Li-Cor). Detail information about the antibodies was listed in the Reagents and Tools Table.

### Immunoprecipitation (IP)

293T cells were transfected with the indicated plasmids (as shown in the figures) using Lipofectamine 3000. Cell lysates were prepared 48 h post-transfection using NP-40 lysis buffer (pH = 7.4) containing 50 mM Tris, 150 mM NaCl, 1 mM EDTA, and 1% NP-40. The lysates were incubated overnight at 4 °C with HA or Flag antibodies, followed by precipitation with the protein A/G magnetic beads (Vazyme, Cat#PB101) at 4 °C for 2 h. The precipitates were washed four times with lysis buffer and eluted in SDS-PAGE loading buffer (GeneStar, Cat#E153) at 95 °C for 5 min. The prepared samples were then analyzed by western blot. For denaturing ubiquitination assay, cell lysates were prepared in the lysis buffer containing 2% SDS and were boiled for 5 min at 95 °C. The high concentration of SDS was then diluted to 0.2% with lysis buffer before immunoprecipitation.

### BrdU labeling

To analyze NPCs at the S phase, BrdU (100 mg/kg) was intraperitoneally administered to pregnant mice at various developmental stages. Mice were sacrificed 30 min after BrdU injection for cortical sample collection. For experiments related to INM, BrdU (50 mg/kg) was intraperitoneally injected to pregnant mice at E15.5, and embryonic cortices were harvested 12 or 24 h post-injection.

### Calculation of Tc

BrdU/EdU (5-ethynyl-2’-deoxyuridine) double-labeling experiments were conducted using a recently reported method (Bertacchi et al, 2020; Martynoga et al, 2005). Pregnant mice at E15.5 were intraperitoneally injected with BrdU (100 mg/kg), followed by an EdU (25 mg/kg) injection 1.5 h later. They were sacrificed 0.5 h after the EdU injection. Consequently, the interval time (*T*_i_) between the two injections is 1.5 h, and the time between the first injection and sample collection is 2 h. The cell cycle time (*T*c) was calculated using the following formulas: *T*_s_ = *T*_i_/(*L*_cells_/*S*_cells_); *T*_c_ = *T*_s_/(*S*_cells_/*P*_cells_). Here, *T*_s_ represents the length of S phase. *L*_cells_ denotes the number of cells which left the S phase and were labeled by BrdU but not EdU. *S*_cells_ refers to the number of cells in the S phase that were stained by EdU. *P*_cells_ represents the number of proliferating cells stained by Ki67 in the VZ.

### Quantitative real-time PCR (qRT-PCR)

Total RNA were extracted from dorsal cortices using TRIzol reagent. 1 μg of total RNA were reverse-transcribed using the PrimeScript RT Reagent Kit (Takara Bio, Cat# RR037A) following the manufacturer’s protocol. Quantitative real-time PCR was conducted using SYBR Green Supermix Reagent (GenStar, Cat#A308) and the Applied Biosystems StepOnePlus System. Quantification was carried out using a comparative Ct method (Dai et al, 2023; Wang et al, 2017). *Gapdh* was used as the internal control. The primers were listed in the Reagents and Tools Table.

### TUNEL assay

We performed TUNEL assay using the BrightGreen Apoptosis Detection Kit (Vazyme, Cat# A112). Following the manufacturer’s instruction, brain sections or cell cultures were fixed, blocked and incubated in a reaction buffer containing terminal deoxynucleotidyl transferase (TdT) and FITC-labeled dUTP at 37 °C for 1 h. Nuclei were counterstained with 1 μg/ml DAPI. Images were acquired using a Leica SP5 confocal laser scanning microscope.

### RNA-Seq and bioinformatics analyses

Total RNA were extracted from the dorsal cortices of control and *Akt* cTKO mice at E13.5 using TRIzol reagent. RNA sequencing library was constructed and sequenced with the Illumina NovaSeq 6000 platform at Shanghai Majorbio Bio-pharm Biotechnology Company. Data analysis was performed using Majorbio Cloud Platform (Ren et al, 2022). Differentially expressed genes (DEGs) in *Akt* cTKO mice were identified using the DESeq2 with 1.5-fold change cutoff. Gene Ontology (GO) functional enrichment analysis was carried out using Goatools. Gene set enrichment analysis (GSEA) was performed using GSEA-related software.

### Cell counting

Cell counting experiments were performed using three to eight animals per genotype. For each embryo, two coronal sections spaced about 200 μm apart were analyzed. IHC images for PAX6, TBR2, BrdU, PH3 and TUNEL were acquired using a ×10 objective on an Olympus BX53 microscope. A rectangular region of interest (ROI) was drawn along the dorsal cortex, spanning from the ventricular to the pial surface. Positive cells for the aforementioned markers in this ROI were quantified using ImageJ.

### Statistical analysis

A range of three to eight mice per genotype were analyzed in this study. Sample sizes were determined based on prior literature using similar experimental paradigms. Two random coronal sections spaced about 200 μm per animal were quantified. To prevent observer bias, data acquisition and analysis were conducted in a blinded manner for both wild-type and knockout animals. One knockout sample and its corresponding littermate control were excluded because the knockout efficiency, as verified by Western blot, was lower than in the other samples. Although normality was not formally tested due to the small sample size, the use of parametric tests is justified by their known robustness for data without extreme outliers. Data are shown as mean ± SEM. A two-tailed unpaired Student’s *t* test was performed to examine the difference between control and *Akt* cTKO mice. For multiple comparisons, one-way ANOVA followed by Tukey-Kramer post hoc test or two-way ANOVA followed by Sidak’s multiple comparisons test was applied. *P* < 0.05 was considered statistically significant. Data analysis was performed using GraphPad Prism 8.

## Supplementary information

Appendix

Peer Review File

Source data Fig. 1

Source data Fig. 2

Source data Fig. 3

Source data Fig. 4

Source data Fig. 5

Source data Fig. 6

Source data Fig. 7

Appendix Figures Source Data

## Data Availability

The RNA-seq raw data reported in this study have been deposited in Sequence Read Achive (SRA) of NCBI under the accession number PRJNA1020756. The source data of this paper are collected in the following database record: biostudies:S-SCDT-10_1038-S44319-026-00768-7.

## References

[CR1] Bertacchi M, Romano AL, Loubat A, Tran Mau-Them F, Willems M, Faivre L, Khau van Kien P, Perrin L, Devillard F, Sorlin A et al (2020) NR2F1 regulates regional progenitor dynamics in the mouse neocortex and cortical gyrification in BBSOAS patients. EMBO J 39:e104163 32484994 10.15252/embj.2019104163 PMC7327499

[CR2] Boland E, Clayton-Smith J, Woo VG, McKee S, Manson FD, Medne L, Zackai E, Swanson EA, Fitzpatrick D, Millen KJ et al (2007) Mapping of deletion and translocation breakpoints in 1q44 implicates the serine/threonine kinase AKT3 in postnatal microcephaly and agenesis of the corpus callosum. Am J Hum Genet 81:292–303 17668379 10.1086/519999 PMC1950798

[CR3] Cappuccio G, Ugga L, Parrini E, D’Amico A, Brunetti-Pierri N (2019) Severe presentation and complex brain malformations in an individual carrying a CCND2 variant. Mol Genet Genom Med 7:e708 10.1002/mgg3.708 PMC6565554 31056854

[CR4] Chaikovsky AC, Li C, Jeng EE, Loebell S, Lee MC, Murray CW, Cheng R, Demeter J, Swaney DL, Chen S-H et al (2021) The AMBRA1 E3 ligase adaptor regulates the stability of cyclin D. Nature 592:794–798 33854239 10.1038/s41586-021-03474-7 PMC8246597

[CR5] Cheng S, Liu T, Hu Y, Xia Y, Hou J, Huang C, Zou X, Liang J, Shi YS, Zheng Y (2019) Conditional inactivation of Pen-2 in the developing neocortex leads to rapid switch of apical progenitors to basal progenitors. J Neurosci 39:2195–2207 30692224 10.1523/JNEUROSCI.2523-18.2019 PMC6433762

[CR6] Cui X, Zhao J, Li G, Yang C, Yang S, Zhan Q, Zhou J, Wang Y, Xiao M, Hong B et al (2023) Blockage of EGFR/AKT and mevalonate pathways synergize the antitumor effect of temozolomide by reprogramming energy metabolism in glioblastoma. Cancer Commun 43:1326–1353 10.1002/cac2.12502 PMC10693308 37920878

[CR7] Dai W, Wang H, Zhan Y, Li N, Li F, Wang J, Yan H, Zhang Y, Wang J, Wu L et al (2023) CCNK gene deficiency influences neural progenitor cells via Wnt5a signaling in CCNK-related syndrome. Ana Neurol. 10.1002/ana.26766 10.1002/ana.26766 37597256

[CR8] Deng Y, Yuan Z, Jin X, Wang Z, Gong R, Ren S, Park JB, Shi B, Yin J (2025) Synapsin III promotes neuronal-like transdifferentiation of glioblastoma stem cells by disrupting JAG1-Notch1 interaction. Neuro-Oncol 27:1686–1701 39994412 10.1093/neuonc/noaf056 PMC12417836

[CR9] Glickstein SB, Monaghan JA, Koeller HB, Jones TK, Ross ME (2009) Cyclin D2 is critical for intermediate progenitor cell proliferation in the embryonic cortex. J Neurosci 29:9614–9624 19641124 10.1523/JNEUROSCI.2284-09.2009 PMC2811167

[CR10] Gookin S, Min M, Phadke H, Chung M, Moser J, Miller I, Carter D, Spencer SL (2017) A map of protein dynamics during cell-cycle progression and cell-cycle exit. PLoS Biol 15:e2003268 28892491 10.1371/journal.pbio.2003268 PMC5608403

[CR11] Gorski JA, Talley T, Qiu M, Puelles L, Rubenstein JL, Jones KR (2002) Cortical excitatory neurons and glia, but not GABAergic neurons, are produced in the Emx1-expressing lineage. J Neurosci 22:6309–6314 12151506 10.1523/JNEUROSCI.22-15-06309.2002 PMC6758181

[CR12] Gotz M, Huttner WB (2005) The cell biology of neurogenesis. Nat Rev Mol Cell Biol 6:777–788 16314867 10.1038/nrm1739

[CR13] Greig LC, Woodworth MB, Galazo MJ, Padmanabhan H, Macklis JD (2013) Molecular logic of neocortical projection neuron specification, development and diversity. Nat Rev Neurosci 14:755–769 24105342 10.1038/nrn3586 PMC3876965

[CR14] Grison A, Atanasoski S (2020) Cyclins, cyclin-dependent kinases, and cyclin-dependent kinase inhibitors in the mouse nervous system. Mol Neurobiol 57:3206–3218 32506380 10.1007/s12035-020-01958-7

[CR15] Groszer M, Erickson R, Scripture-Adams DD, Lesche R, Trumpp A, Zack JA, Kornblum HI, Liu X, Wu H (2001) Negative regulation of neural stem/progenitor cell proliferation by the Pten tumor suppressor gene in vivo. Science 294:2186–2189 11691952 10.1126/science.1065518

[CR16] Guerrini R, Dobyns WB (2014) Malformations of cortical development: clinical features and genetic causes. Lancet Neurol 13:710–726 24932993 10.1016/S1474-4422(14)70040-7 PMC5548104

[CR17] Herzinger T, Reed SI (1998) Cyclin D3 is rate-limiting for the G1/S phase transition in fibroblasts. J Biol Chem 273:14958–14961 9614101 10.1074/jbc.273.24.14958

[CR18] Hirai H, Sootome H, Nakatsuru Y, Miyama K, Taguchi S, Tsujioka K, Ueno Y, Hatch H, Majumder PK, Pan B-S et al (2010) MK-2206, an allosteric Akt inhibitor, enhances antitumor efficacy by standard chemotherapeutic agents or molecular targeted drugs in vitro and in vivo. Mol Cancer Ther 9:1956–1967 20571069 10.1158/1535-7163.MCT-09-1012

[CR19] Hodge RD, D’ercole AJ, O’kusky JR (2004) Insulin-like growth factor-I accelerates the cell cycle by decreasing G1 phase length and increases cell cycle reentry in the embryonic cerebral cortex. J Neurosci 24:10201–10210 15537892 10.1523/JNEUROSCI.3246-04.2004 PMC6730172

[CR20] Howell KR, Floyd K, Law AJ (2017) PKBγ/AKT3 loss-of-function causes learning and memory deficits and deregulation of AKT/mTORC2 signaling: relevance for schizophrenia. PLoS ONE 12:e0175993 28467426 10.1371/journal.pone.0175993 PMC5414975

[CR21] Jossin Y, Goffinet AM (2007) Reelin signals through phosphatidylinositol 3-kinase and Akt to control cortical development and through mTOR to regulate dendritic growth. Mol Cell Biol 27:7113–7124 17698586 10.1128/MCB.00928-07 PMC2168915

[CR22] Kosodo Y (2012) Interkinetic nuclear migration: beyond a hallmark of neurogenesis. Cell Mol Life Sci 69:2727–2738 22415322 10.1007/s00018-012-0952-2 PMC11115108

[CR23] Lai D, Gade M, Yang E, Koh HY, Lu J, Walley NM, Buckley AF, Sands TT, Akman CI, Mikati MA et al (2022) Somatic variants in diverse genes leads to a spectrum of focal cortical malformations. Brain 145:2704–2720 35441233 10.1093/brain/awac117 PMC9612793

[CR24] Lange C, Huttner WB, Calegari F (2009) Cdk4/cyclinD1 overexpression in neural stem cells shortens G1, delays neurogenesis, and promotes the generation and expansion of basal progenitors. Cell Stem Cell 5:320–331 19733543 10.1016/j.stem.2009.05.026

[CR25] Lathia JD, Mack SC, Mulkearns-Hubert EE, Valentim CL, Rich JN (2015) Cancer stem cells in glioblastoma. Genes Dev 29:1203–1217 26109046 10.1101/gad.261982.115 PMC4495393

[CR26] Levenga J, Wong H, Milstead RA, Keller BN, LaPlante L, Hoeffer CA (2017) AKT isoforms have distinct hippocampal expression and roles in synaptic plasticity. eLife 6:e30640 29173281 10.7554/eLife.30640 PMC5722612

[CR27] Li D, Zhang Q, Li L, Chen K, Yang J, Dixit D, Gimple RC, Ci S, Lu C, Hu L et al (2022) β2-microglobulin maintains glioblastoma stem cells and induces M2-like polarization of tumor-associated macrophages. Cancer Res 82:3321–3334 35841593 10.1158/0008-5472.CAN-22-0507

[CR28] Li Y, Li Z, Wang C, Yang M, He Z, Wang F, Zhang Y, Li R, Gong Y, Wang B et al (2023) Spatiotemporal transcriptome atlas reveals the regional specification of the developing human brain. Cell 186:5892–5909.e5822 38091994 10.1016/j.cell.2023.11.016

[CR29] Lim S, Kaldis P (2012) Loss of Cdk2 and Cdk4 induces a switch from proliferation to differentiation in neural stem cells. Stem Cells 30:1509–1520 22532528 10.1002/stem.1114

[CR30] Liu L, Michowski W, Kolodziejczyk A, Sicinski P (2019) The cell cycle in stem cell proliferation, pluripotency and differentiation. Nat Cell Biol 21:1060–1067 31481793 10.1038/s41556-019-0384-4 PMC7065707

[CR31] Lukas J, Bartkova J, Welcker M, Petersen OW, Bartek J (1995) Cyclin D2 is a moderately oscillating nucleoprotein required for G1 phase progression in specific cell types. Oncogene 10:2125–2134 7784057

[CR32] Maiani E, Milletti G, Nazio F, Holdgaard SG, Bartkova J, Rizza S, Cianfanelli V, Lorente M, Simoneschi D, Di Marco M et al (2021) AMBRA1 regulates cyclin D to guard S-phase entry and genomic integrity. Nature 592:799–803 33854232 10.1038/s41586-021-03422-5 PMC8864551

[CR33] Mairet-Coello G, Tury A, DiCicco-Bloom E (2009) Insulin-like growth factor-1 promotes G1 cell cycle progression through bidirectional regulation of cyclins and cyclin-dependent kinase inhibitors via the phosphatidylinositol 3-kinase/Akt pathway in developing rat cerebral cortex. J Neurosci 29:775–788 19158303 10.1523/JNEUROSCI.1700-08.2009 PMC3256126

[CR34] Mairet-Coello G, Tury A, Van Buskirk E, Robinson K, Genestine M, DiCicco-Bloom E (2012) p57KIP2 regulates radial glia and intermediate precursor cell cycle dynamics and lower layer neurogenesis in developing cerebral cortex. Development 139:475–487 22223678 10.1242/dev.067314 PMC3252351

[CR35] Manning BD, Toker A (2017) AKT/PKB signaling: navigating the network. Cell 169:381–405 28431241 10.1016/j.cell.2017.04.001 PMC5546324

[CR36] Martynoga B, Morrison H, Price DJ, Mason JO (2005) Foxg1 is required for specification of ventral telencephalon and region-specific regulation of dorsal telencephalic precursor proliferation and apoptosis. Dev Biol 283:113–127 15893304 10.1016/j.ydbio.2005.04.005

[CR37] Mirzaa G, Parry DA, Fry AE, Giamanco KA, Schwartzentruber J, Vanstone M, Logan CV, Roberts N, Johnson CA, Singh S et al (2014) De novo CCND2 mutations leading to stabilization of cyclin D2 cause megalencephaly-polymicrogyria-polydactyly-hydrocephalus syndrome. Nat Genet 46:510–515 24705253 10.1038/ng.2948 PMC4004933

[CR38] Molyneaux BJ, Arlotta P, Menezes JR, Macklis JD (2007) Neuronal subtype specification in the cerebral cortex. Nat Rev Neurosci 8:427–437 17514196 10.1038/nrn2151

[CR39] Mukhtar T, Breda J, Adam MA, Boareto M, Grobecker P, Karimaddini Z, Grison A, Eschbach K, Chandrasekhar R, Vermeul S et al (2022) Temporal and sequential transcriptional dynamics define lineage shifts in corticogenesis. EMBO J 41:e111132 36345783 10.15252/embj.2022111132 PMC9753470

[CR40] Muzumdar MD, Tasic B, Miyamichi K, Li L, Luo L (2007) A global double-fluorescent Cre reporter mouse. Genesis 45:593–605 17868096 10.1002/dvg.20335

[CR41] Obenauer JC, Cantley LC, Yaffe MB (2003) Scansite 2.0: proteome-wide prediction of cell signaling interactions using short sequence motifs. Nucleic Acids Res 31:3635–3641 12824383 10.1093/nar/gkg584 PMC168990

[CR42] Ochoa A, Herrera A, Menendez A, Estefanell M, Ramos C, Pons S (2023) Vinculin is required for interkinetic nuclear migration (INM) and cell cycle progression. J Cell Biol 223:e202106169 37889294 10.1083/jcb.202106169 PMC10609122

[CR43] Pagano M, Theodoras AM, Tam SW, Draetta GF (1994) Cyclin D1-mediated inhibition of repair and replicative DNA synthesis in human fibroblasts. Genes Dev 8:1627–1639 7958844 10.1101/gad.8.14.1627

[CR44] Palumbo S, Paterson C, Yang F, Hood VL, Law AJ (2021) PKBβ/AKT2 deficiency impacts brain mTOR signaling, prefrontal cortical physiology, hippocampal plasticity and select murine behaviors. Mol Psychiatry 26:411–428 33328589 10.1038/s41380-020-00964-4 PMC7854513

[CR45] Pearce LR, Komander D, Alessi DR (2010) The nuts and bolts of AGC protein kinases. Nat Rev Mol Cell Biol 11:9–22 20027184 10.1038/nrm2822

[CR46] Pilaz L-J, Patti D, Marcy G, Ollier E, Pfister S, Douglas RJ, Betizeau M, Gautier E, Cortay V, Doerflinger N et al (2009) Forced G1-phase reduction alters mode of division, neuron number, and laminar phenotype in the cerebral cortex. Proc Natl Acad Sci USA 106:21924–21929 19959663 10.1073/pnas.0909894106 PMC2788480

[CR47] Poduri A, Evrony GD, Cai X, Elhosary PC, Beroukhim R, Lehtinen MK, Hills LB, Heinzen EL, Hill A, Hill RS et al (2012) Somatic activation of AKT3 causes hemispheric developmental brain malformations. Neuron 74:41–48 22500628 10.1016/j.neuron.2012.03.010 PMC3460551

[CR48] Raybaud C, Widjaja E (2011) Development and dysgenesis of the cerebral cortex: malformations of cortical development. Neuroimaging Clin 21:483–543 10.1016/j.nic.2011.05.014 21807310

[CR49] Ren Y, Yu G, Shi C, Liu L, Guo Q, Han C, Zhang D, Zhang L, Liu B, Gao H et al (2022) Majorbio cloud: a one-stop, comprehensive bioinformatic platform for multiomics analyses. iMeta 1:e12 38868573 10.1002/imt2.12 PMC10989754

[CR50] Riviere JB, Mirzaa GM, O’Roak BJ, Beddaoui M, Alcantara D, Conway RL, St-Onge J, Schwartzentruber JA, Gripp KW, Nikkel SM et al (2012) De novo germline and postzygotic mutations in AKT3, PIK3R2 and PIK3CA cause a spectrum of related megalencephaly syndromes. Nat Genet 44:934–940 22729224 10.1038/ng.2331 PMC3408813

[CR51] Rogerson C, Wotherspoon DJ, Tommasi C, Button RW, O’Shaughnessy RFL (2021) Akt1-associated actomyosin remodelling is required for nuclear lamina dispersal and nuclear shrinkage in epidermal terminal differentiation. Cell Death Differ 28:1849–1864 33462407 10.1038/s41418-020-00712-9 PMC8184862

[CR52] Salomoni P, Calegari F (2010) Cell cycle control of mammalian neural stem cells: putting a speed limit on G1. Trends Cell Biol 20:233–243 20153966 10.1016/j.tcb.2010.01.006

[CR53] Sameshima T, Morisada N, Egawa T, Kugo M, Iijima K (2020) MPPH syndrome with aortic coarctation and macrosomia due to CCND2 mutations. Pediatrics Int 62:115–117 10.1111/ped.14068 31957131

[CR54] Sherr CJ (1995) D-type cyclins. Trends Biochem Sci 20:187–190 7610482 10.1016/s0968-0004(00)89005-2

[CR55] Sicinska E, Aifantis I, Le Cam L, Swat W, Borowski C, Yu Q, Ferrando AA, Levin SD, Geng Y, von Boehmer H et al (2003) Requirement for cyclin D3 in lymphocyte development and T cell leukemias. Cancer Cell 4:451–461 14706337 10.1016/s1535-6108(03)00301-5

[CR56] Simoneschi D, Rona G, Zhou N, Jeong Y-T, Jiang S, Milletti G, Arbini AA, O’Sullivan A, Wang AA, Nithikasem S et al (2021) CRL4AMBRA1 is a master regulator of D-type cyclins. Nature 592:789–793 33854235 10.1038/s41586-021-03445-y PMC8875297

[CR57] Smyth LM, Tamura K, Oliveira M, Ciruelos EM, Mayer IA, Sablin M-P, Biganzoli L, Ambrose HJ, Ashton J, Barnicle A et al (2020) Capivasertib, an AKT kinase inhibitor, as monotherapy or in combination with fulvestrant in patients with AKT1 E17K mutant, ER-positive metastatic breast cancer. Clin Cancer Res 26:3947–3957 32312891 10.1158/1078-0432.CCR-19-3953 PMC7415507

[CR58] Taverna E, Gotz M, Huttner WB (2014) The cell biology of neurogenesis: toward an understanding of the development and evolution of the neocortex. Annu Rev Cell Dev Biol 30:465–502 25000993 10.1146/annurev-cellbio-101011-155801

[CR59] Thierry G, Bénéteau C, Pichon O, Flori E, Isidor B, Popelard F, Delrue M-A, Duboscq-Bidot L, Thuresson A-C, van Bon BWM et al (2012) Molecular characterization of 1q44 microdeletion in 11 patients reveals three candidate genes for intellectual disability and seizures. Am J Med Genet 158A:1633–1640 22678713 10.1002/ajmg.a.35423

[CR60] Tschopp O, Yang ZZ, Brodbeck D, Dummler BA, Hemmings-Mieszczak M, Watanabe T, Michaelis T, Frahm J, Hemmings BA (2005) Essential role of protein kinase B gamma (PKB gamma/Akt3) in postnatal brain development but not in glucose homeostasis. Development 132:2943–2954 15930105 10.1242/dev.01864

[CR61] Wang H, Liu M, Ye Z, Zhou C, Bi H, Wang L, Zhang C, Fu H, Shen Y, Yang JJ et al (2021a) Akt regulates Sox10 expression to control oligodendrocyte differentiation via phosphorylating FoxO1. J Neurosci 41:8163–8180 34385359 10.1523/JNEUROSCI.2432-20.2021 PMC8482862

[CR62] Wang H, Liu M, Zou G, Wang L, Duan W, He X, Ji M, Zou X, Hu Y, Yang J et al (2021b) Deletion of PDK1 in oligodendrocyte lineage cells causes white matter abnormality and myelination defect in the central nervous system. Neurobiol Dis 148:105212 33276084 10.1016/j.nbd.2020.105212

[CR63] Wang H, Zhang B, Zhang T, Wang L, Zou X, Xu Y, Chen L, Chen G (2017) Impaired spatial learning is associated with disrupted integrity of the white matter in Akt3 knockout mice. CNS Neurosci Ther 23:99–102 27671373 10.1111/cns.12647 PMC6492745

[CR64] Wang Y, Wu Q, Yang P, Wang C, Liu J, Ding W, Liu W, Bai Y, Yang Y, Wang H et al (2016) LSD1 co-repressor Rcor2 orchestrates neurogenesis in the developing mouse brain. Nat Commun 7:10481 26795843 10.1038/ncomms10481 PMC4736047

[CR65] Waterhouse A, Bertoni M, Bienert S, Studer G, Tauriello G, Gumienny R, Heer FT, de Beer TAP, Rempfer C, Bordoli L et al (2018) SWISS-MODEL: homology modelling of protein structures and complexes. Nucleic Acids Res 46:W296–W303 29788355 10.1093/nar/gky427 PMC6030848

[CR66] White SM, Bhoj E, Nellåker C, Lachmeijer AMA, Marshall AE, Boycott KM, Li D, Smith W, Hartley T, McBride A et al (2021) A DNA repair disorder caused by de novo monoallelic DDB1 variants is associated with a neurodevelopmental syndrome. Am J Hum Genet 108:749–756 33743206 10.1016/j.ajhg.2021.03.007 PMC8059373

[CR67] Xia WL, Jiao JW (2017) Histone variant H3.3 orchestrates neural stem cell differentiation in the developing brain. Cell Death Differ 24:1548–1563 28524856 10.1038/cdd.2017.77 PMC5563987

[CR68] Xu C, Yu L, Hou J, Jackson R, Wang H, Huang C, Liu T, Wang Q, Zou X, Morris R et al (2017) Conditional deletion of PDK1 in the forebrain causes neuron loss and increased apoptosis during cortical development. Front Cell Neurosci 11:330 29104535 10.3389/fncel.2017.00330 PMC5655024

[CR69] Ye X, Yao L, Chen W, Shao W, Hu Y, Zhang B, Chen G (2022) Emx1-Cre-mediated inactivation of PDK1 prevents plaque deposition in an Alzheimer’s disease-like mouse model. Adv Neurol 1:153

[CR70] Zhang J, Jiao JW (2015) Molecular biomarkers for embryonic and adult neural stem cell and neurogenesis. Biomed Res Int 1:727542 10.1155/2015/727542 PMC4569757 26421301

[CR71] Zhang T, Shi Z, Wang Y, Wang L, Zhang B, Chen G, Wan Q, Chen L (2019) Akt3 deletion in mice impairs spatial cognition and hippocampal CA1 long long-term potentiation through downregulation of mTOR. Acta Physiol 225:e13167 10.1111/apha.13167 30053339

[CR72] Zhu T-Z, Li X-M, Luo L-H, Song Z-Q, Gao X, Li Z-Q, Su J-Y, Liang G-B (2014) β-elemene inhibits stemness, promotes differentiation and impairs chemoresistance to temozolomide in glioblastoma stem-like cells. Int J Oncol 45:699–709 24841897 10.3892/ijo.2014.2448

