# Supplementary material for: CRL4AMBRA1 is a key mediator for AKT-dependent cell cycle control in neural progenitor cells
Source: EMBO Rep. 2026 Apr 27;27(11):3099–119. doi: 10.1038/s44319-026-00768-7 (PMC13260899; doi:10.1038/s44319-026-00768-7)
Supplement: Supplementary file 1 — Appendix [file 44319_2026_768_MOESM1_ESM.pdf]

APPENDIX

CRL4^{AMBRA1} Is a Key Mediator for AKT-dependent Cell Cycle Control in Neural Progenitor Cells

He Wang*, Panmiao Liu, Runmin Wang, Hanwen Gu, Tingting Zhu, Guiquan Chen*, Jian-Jun Yang*

*Correspondence: He Wang, wangheriver@zzu.edu.cn; Guiquan Chen, chenguiquan@nju.edu.cn; Jian-Jun Yang, yjyangjj@zzu.edu.cn

TABLE OF CONTENTS

Appendix Figures

Appendix Figure S1. Abundant expression of AKT in human and mouse NPCs……………..2
Appendix Figure S2. Molecular characterization of NPC-specific *Akt* cTKO mice…………..4
Appendix Figure S3. Reduced neurogenesis in *Akt* cTKO mice……………………..…..……6
Appendix Figure S4. Down-regulation of neurogenic genes in *Akt* cTKO mice……….…….7
Appendix Figure S5. Delineation of R1 in the VZ………………………………….……………8
Appendix Figure S6. CCND1/3 expression is regulated by AKT…………………………....10
Appendix Figure S7. Phosphorylation of DDB1 changes its interaction with components of CRL4^{AMBRA1}…………………………………………………………………….....…13

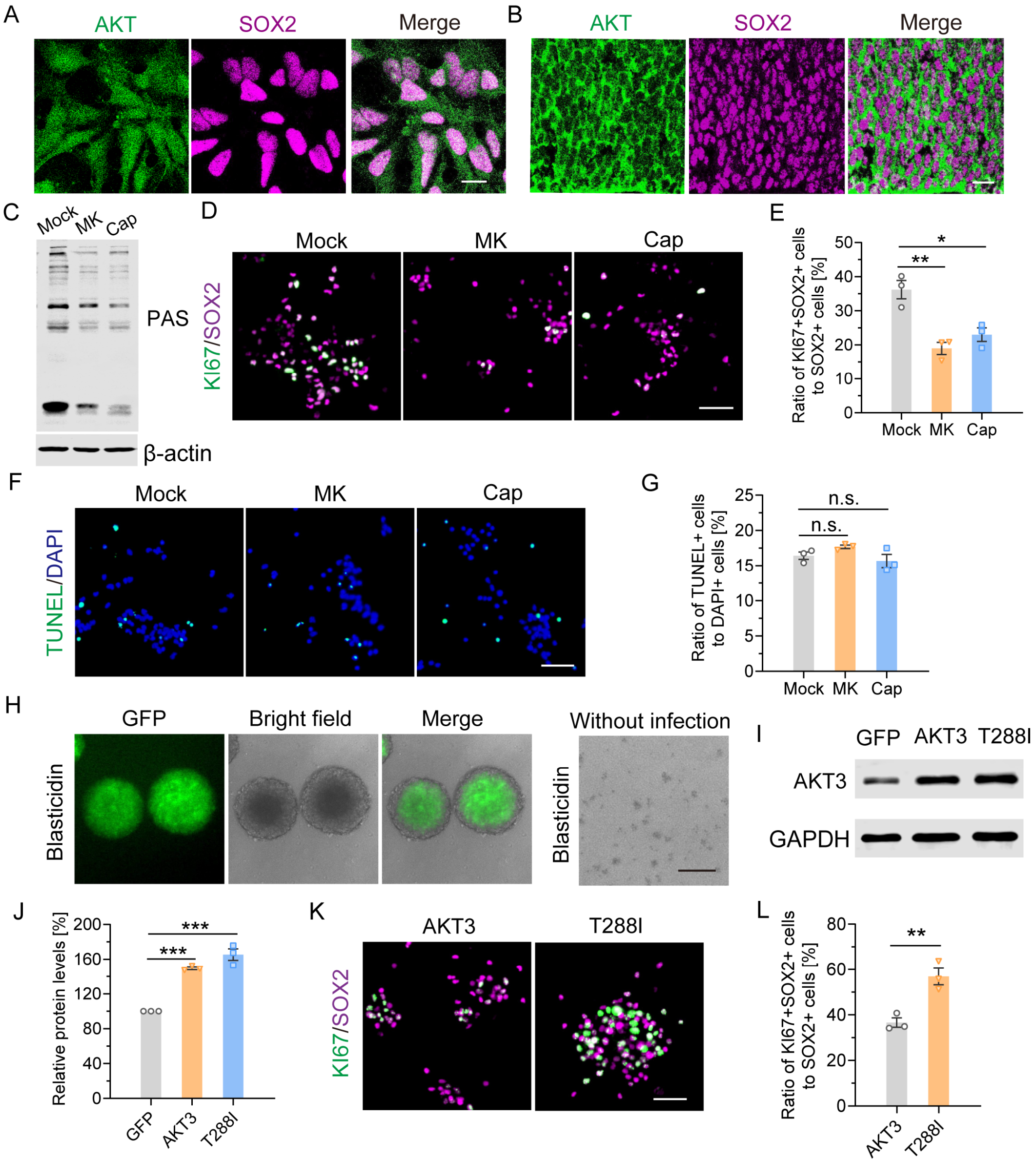

Appendix Figure S1. Abundant expression of AKT in human and mouse NPCs.

(A) Co-staining of AKT/SOX2 in human ENSA cells. There was strong immuno-reactivity of AKT in the ENSA cells positive for SOX2. The scale bar is 20μm.

(B) Co-staining for AKT/SOX2 in the mouse cortex at E13.5. AKT was abundantly expressed in the SOX2+ cells in the cortex. The scale bar is 20μm.

(C) Western blot for phospho-AKT substrates (PAS). Neurospheres were cultured using mouse cortical tissues at E15.5. Cells were treated with 1μM MK2206 (MK) or 1μM capivasertib (Cap) for 24 hrs. Cell lysates were collected at DIV2 for Western blot. Levels of PAS were robustly decreased in the MK- or Cap-treated neurospheres compared with mock-treated neurospheres. β-actin served as the loading control.

(D) Co-staining of KI67/SOX2 in mouse NPCs. Neurospheres derived from E15.5 cortical tissues were treated with 1μM MK or 1μM Cap for 2 days. These cells were transferred to poly-L-ornithine/laminin-coated coverslips for 24 hrs and examined by immunocytochemistry. The scale bar is 50 μm.

(E) Quantification of KI67+/SOX2+ cells. The ratio of KI67+/SOX2+ cells to SOX2+ cells was significantly reduced in MK- or Cap- treated cells compared to mock-treated controls. Data are shown as mean ± SEM (n=3 independent experiments, One-way ANOVA, **, p = 0.0036, *, p = 0.0130).

(F, G) TUNEL assay in mouse NPCs. Neurospheres were treated with 1μM MK or 1μM Cap for 2 days and were transferred to poly-L-ornithine/laminin-coated coverslips for 24 hrs, followed by TUNEL examination. No significant difference was observed between MK- or Cap- treated cells and controls. The scale bar is 50 μm. Data are shown as mean ± SEM (n=3 independent experiments, One-way ANOVA, n.s., p = 0.3999 for MK, p = 0.6953 for Cap).

(H) Neurosphere images. Neurospheres were infected with the lentivirus carrying GFP and blasticidin S deaminase (BSD). The blasticidin (10 μg/ml) was added 2 days after infection to select infected cells. Pictures were captured at day 7 during Passage 2. The scale bar is 50 μm.

(I, J) AKT3 and $AKT3^{T288I}$ overexpression in neurospheres. These cells were infected with lentivirus expressing GFP, AKT3 or $AKT3^{T288I}$ for 2 days, selected with blasticidin for 3 days and then analyzed by Western blot. Data are shown as mean ± SEM (n=3 independent experiments, One-way ANOVA, ***, p = 0.0002 for AKT3, p < 0.0001 for T288I).

(K) Co-staining of KI67/SOX2 in mouse NPCs overexpressed with AKT3 or $AKT3^{T288I}$. These cells were infected with lentivirus expressing AKT3 or $AKT3^{T288I}$, transferred to poly-L-ornithine/ laminin-coated coverslips for 24 hrs and examined by immunocytochemistry at day 3 during Passage 2. The scale bar is 50 μm.

(L) Quantification of KI67+/SOX2+ cells. The ratio of KI67+/SOX2+ cells to SOX2+ cells was significantly increased in cells overexpressing $AKT3^{T288I}$ compared to that overexpressing AKT3. Data are shown as mean ± SEM (n=3 independent experiments, Student's t test, **, p = 0.0085).

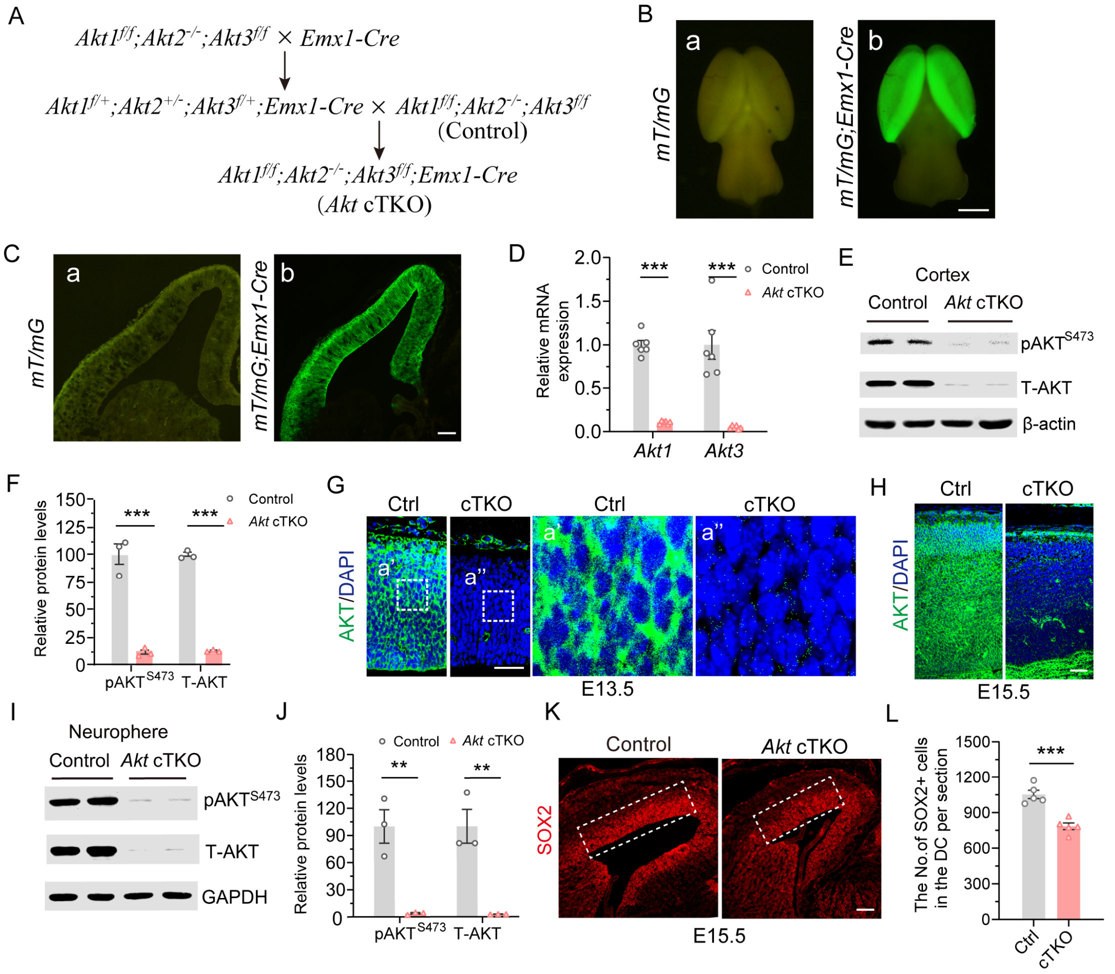

Appendix Figure S2. Molecular characterization of NPC-specific *Akt* cTKO mice.

(A) Breeding strategy for the generation of *Akt* cTKO mice. *Akt1*$^{f/f}$*;Akt2*$^{-/-}$*;Akt3*$^{f/f}$ mice were crossed to *Emx1-Cre* to obtain *Akt1*$^{f/+}$*;Akt2*$^{+/-}$*;Akt3*$^{f/+}$*;Emx1-Cre*. The latter were backcrossed to *Akt1*$^{f/f}$*;Akt2*$^{-/-}$*;Akt3*$^{f/f}$ to generate *Akt1*$^{f/f}$*;Akt2*$^{-/-}$*;Akt3*$^{f/f}$*; Emx1-Cre* mice (*Akt* cTKO).

(B, C) Photos for brains expressing GFP. The brain of *mTmG;Emx1-Cre* mice at E13.5 showed green fluorescence (B). The cortex of *mTmG;Emx1-Cre* mice exhibited abundant GFP+ signals (C). The scale bar is 500μm in (B) or 100μm in (C).

(D) Relative levels of *Akt1* and *Akt3* in the cortex of *Akt* cTKO mice at E15.5. Quantitative RT-PCR analysis showed highly significant reductions on *Akt1* and *Akt3* mRNA levels in *Akt* cTKO mice. Data are shown as mean ± SEM (n=6 mice per genotype, Student's *t* test, ***, $p < 0.0001$ for *Akt1*, $p = 0.0002$ for *Akt3*).

(E, F) Western blot analysis for pAKT^{S473} and T-AKT in *Akt* cTKO mice at E15.5. There were significant decreases on levels of pAKT^{S473} and T-AKT in the cortex of *Akt* cTKO mice. β-actin was the loading control. Data are shown as mean ± SEM (n=3 mice per genotype,

Student's *t* test, ***, $p = 0.0009$ for $pAKT^{S473}$, $p < 0.0001$ for T-AKT).

(G) Representative fluorescence images of IHC for AKT in the cortex. Brain sections were prepared from control and *Akt* cTKO mice at E13.5. The scale bar is 50 µm. There was abundant fluorescence of AKT in the cortex of control mice but not in *Akt* cTKO mice. Boxed areas of a' and a'' were enlarged.

(H) Representative fluorescence images for total AKT in the cortex. Mice at E15.5 were used. AKT+ signals were barely detected in the cortex of *Akt* cTKO mice compared with controls. The scale bar is 50µm.

(I) Western blot for $pAKT^{S473}$ and T-AKT in neurospheres derived from control and *Akt* cTKO mice. Cell lysates were prepared from neurospheres cultured to DIV7. GAPDH served as the internal control.

(J) Quantification of $pAKT^{S473}$ and T-AKT levels. Both $pAKT^{S473}$ and T-AKT levels were significantly decreased in *Akt* cTKO neurospheres compared to controls. The results represent mean ± SEM (n=3 independent experiments, Student's *t* test, **, $p = 0.0065$ for $pAKT^{S473}$, $p = 0.0065$ for T-AKT).

(K) Representative fluorescence images for SOX2. Brain sections of mice at E15.5 were used for IHC. The scale bar is 100µm.

(L) Average number of SOX2+ cells. SOX2+ cells in the dorsal cortex were counted across sections. There was a significant reduction on the average number of SOX2+ cells in *Akt* cTKO mice. Data are shown as mean ± SEM (n=5 mice per genotype, Student's *t* test, ***, $p = 0.0003$).

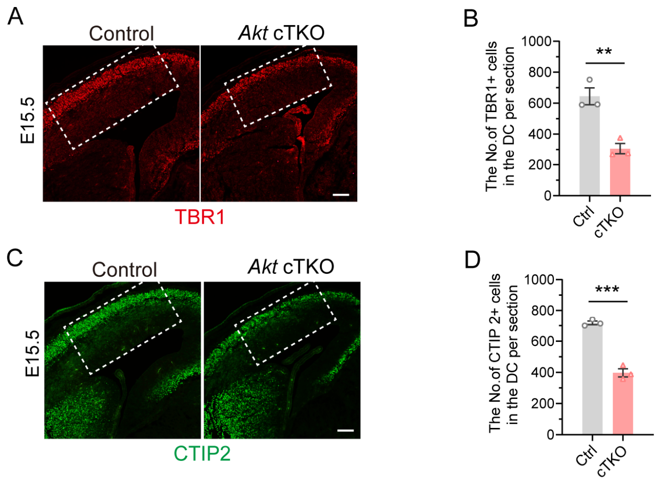

Appendix Figure S3. Reduced neurogenesis in *Akt* cTKO mice.

(A) Representative fluorescence images for TBR1 in the E15.5 dorsal cortex. The scale bar is 100μm.

(B) Average number of TBR1+ cells in the dorsal cortex (DC). There were significant decreases in *Akt* cTKO mice compared to controls. Data are shown as mean ± SEM (n=3 mice per group, Student's *t* test, **, $p = 0.0063$).

(C) Representative fluorescence images for CTIP2 in the dorsal cortex. Brain sections of mice at E15.5 were used. The scale bar is 100μm.

(D) Quantification of the CTIP2+ cell number. There were significant decreases in the dorsal cortex of *Akt* cTKO mice compared to controls. Data are shown as mean ± SEM (n=3 mice per group, Student's *t* test, ***, $p = 0.0004$).

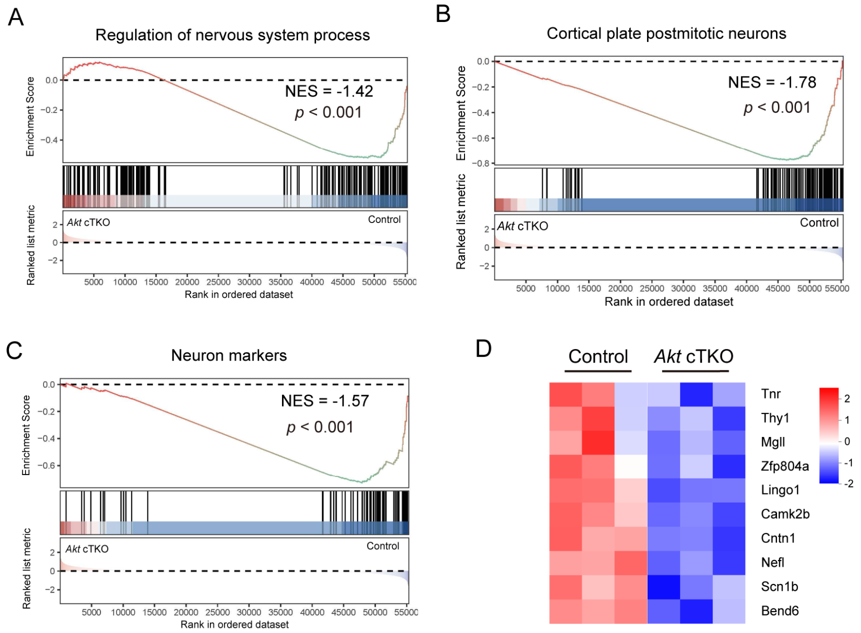

Appendix Figure S4. Down-regulation of neurogenic genes in *Akt* cTKO mice.

(A-C) GSEA on neurogenic genes in *Akt* cTKO mice at E13.5. *Akt* cTKO mice exhibited significantly reduced expression of genes involved in the following neurogenesis-related pathways, nervous system process (A), cortical plate postmitotic neurons (B) and neuron markers (C). NES stands for normalized enrichment score.

(D) Heatmap for a number of neurogenic genes with down-regulation in *Akt* cTKO mice. Genes such as *Thy1*, *Camk2b* and many others were shown.

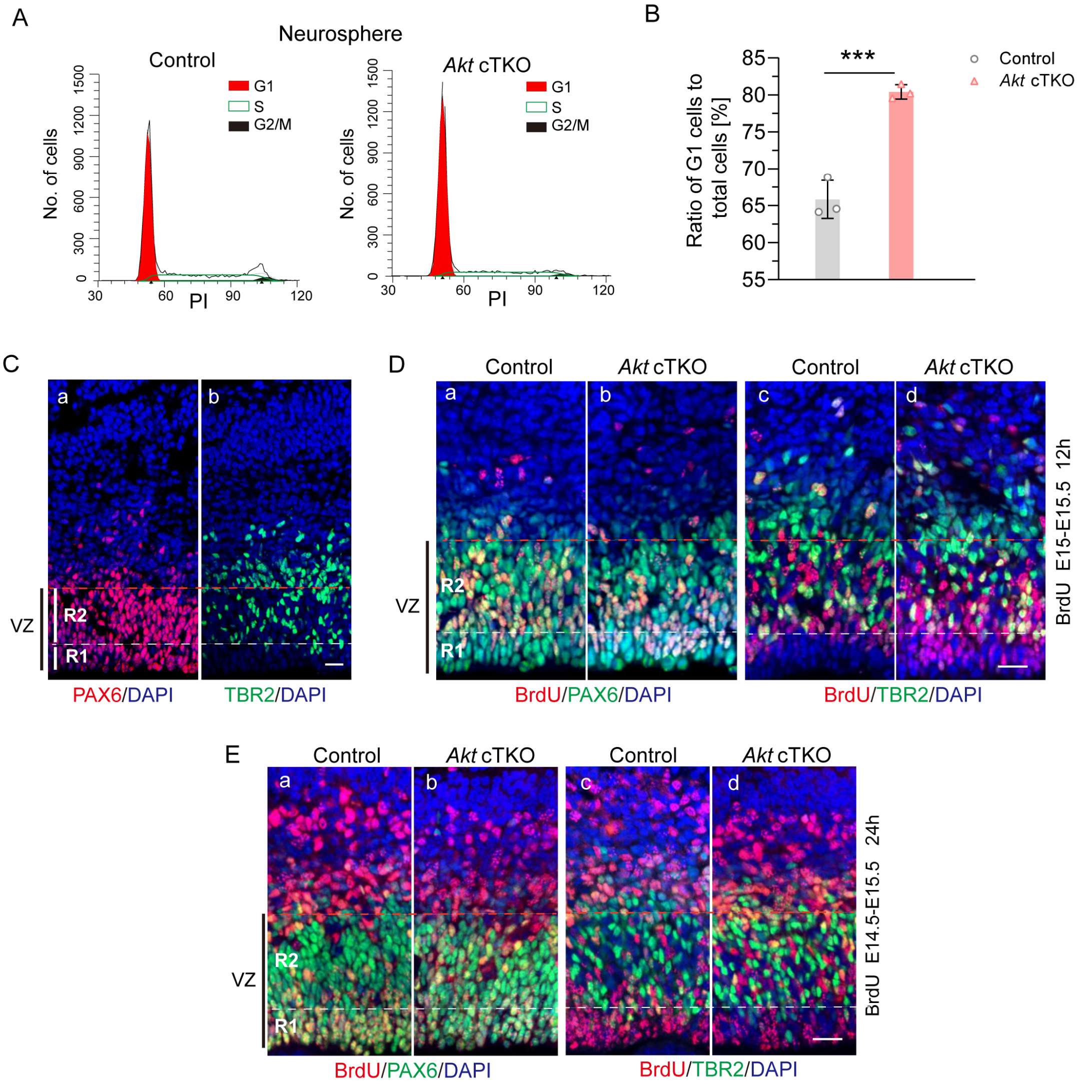

Appendix Figure S5. Delineation of R1 in the VZ.

(A, B) Cell cycle analysis by the FC system. Neurospheres from control and *Akt* cTKO mice were digested, stained with PI and analyzed at DIV3. The number of cells at the G1 phase was significantly increased in *Akt* cTKO neurospheres compared with controls. Data are shown as mean ± SEM (n=3 independent experiments, Student's *t* test, ***, $p = 0.0008$).

(C) Representative fluorescence images for PAX6 and TBR2. Brain sections of a control mouse at E15.5 were used for fluorescence IHC for PAX6 (a) or TBR2 (b). Apical VZ (ventricular zone) was defined as Region1 (R1), in which cells were positive for PAX6 and negative for TBR2 (PAX6+/TBR2-). The remaining part of VZ was defined as Region 2 (R2) in the cortex. The scale bar is 20μm.

(D) Representative fluorescence images for co-staining of PAX6/BrdU (a, b) or TBR2/BrdU (c, d). Control and *Akt* cTKO mice at E15 were injected with BrdU, and brain tissues were

collected at E15.5. The scale bar is 20μm.

(E) Representative fluorescence images for co-staining of PAX6/BrdU (a, b) or TBR2/BrdU (c, d). Control and *Akt* cTKO mice at E14.5 were injected with BrdU, and brain tissues were collected at E15.5. The scale bar is 20μm.

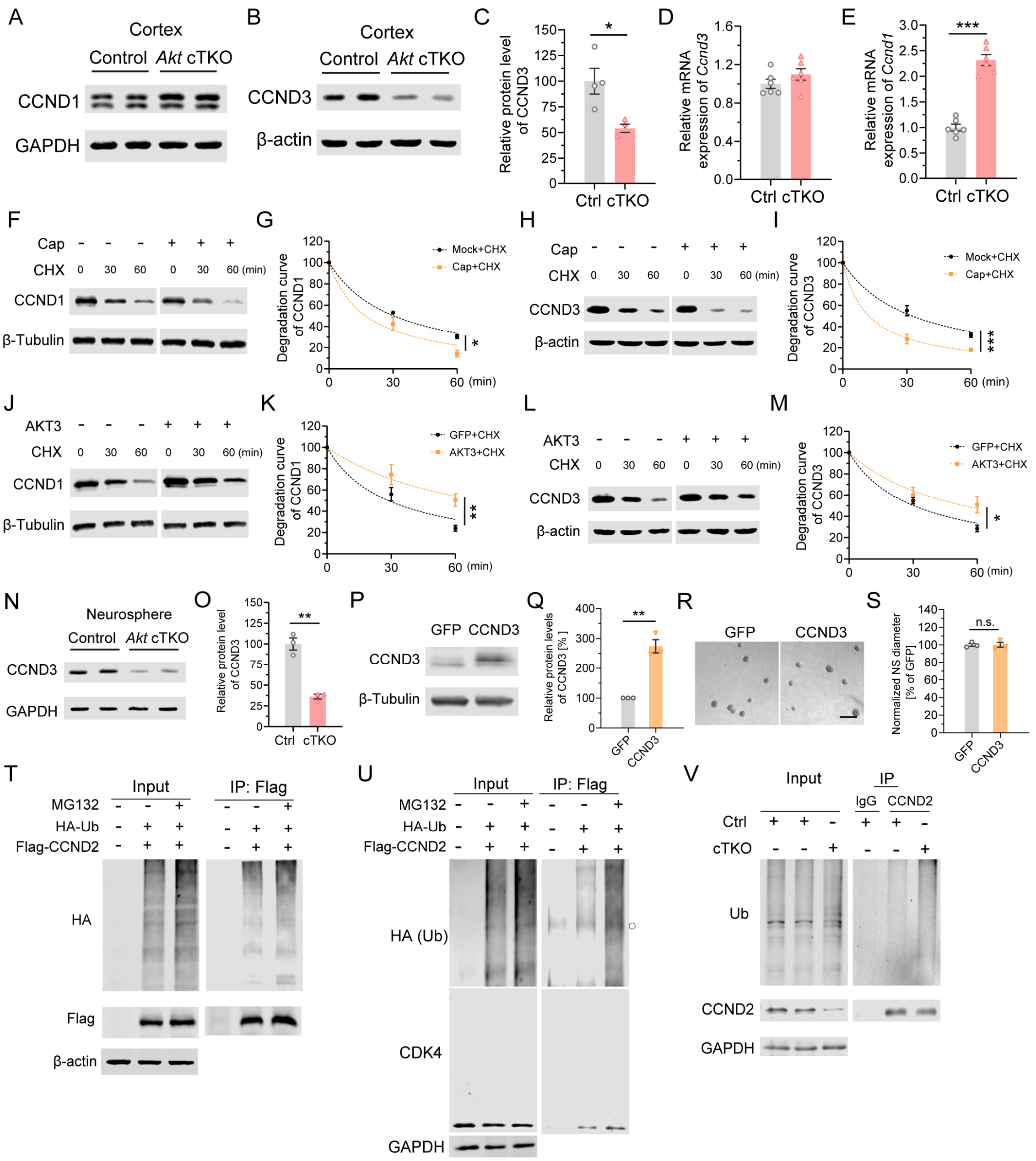

Appendix Figure S6. CCND1/3 expression is regulated by AKT.

(A) Western blot for CCND1. There was no reduction on CCND1 levels in the cortex of *Akt* cTKO mice compared with controls. Cortical lysates were prepared from mice at E15.5. GAPDH served as the loading control.

(B, C) Western blot analysis for CCND3 in cortical lysates from control and *Akt* cTKO mice at E15.5. CCND3 protein levels were significantly reduced in the cortex of *Akt* cTKO mice compared to controls. β-actin was used as a loading control. Data are shown as mean ± SEM (n=4 mice per genotype, Student's *t* test, *, $p = 0.0131$).

(D) qRT-PCR analysis of *Ccnd3* mRNA levels. *Ccnd3* expression was not changed in the

cortex of *Akt* cTKO mice at E15.5 compared to controls. Data are shown as mean ± SEM (n=6 mice per genotype, Student's *t* test, $p = 0.2313$).

(E) qRT-PCR analysis of *Ccnd1* mRNA levels. *Ccnd1* expression was significantly increased in *Akt* cTKO cortices at E15.5 compared to controls. Data are shown as mean ± SEM (n=6 mice per genotype, Student's *t* test, ***, $p < 0.0001$).

(F, G) CCND1 protein stability assay. 293T cells transfected with *Ccnd1* were treated with 5 μM Cap for 12 hrs. Protein was extracted at 0, 30 and 60 min after 50 μM CHX treatment and analyzed by Western blot (F). CCND1 degradation was significantly accelerated in Cap-treated group (G). Data are shown as mean ± SEM (n=3 independent experiments, Two-way ANOVA, *, $p = 0.0129$).

(H, I) CCND3 protein stability assay. A parallel assay showed AKT inhibition accelerated CCND3 degradation. Data are shown as mean ± SEM (n=3 independent experiments, Two-way ANOVA, ***, $p = 0.0001$).

(J, K) CCND1 stability analysis. 293T cells were transfected with *Ccnd1* and *Akt3* for 24h, followed by treatment with 50 μM CHX. Cells were harvested at 0, 30 or 60 min after CHX addition and analyzed by Western blot (J). CCND1 degradation was significantly inhibited in *Akt3*-transfected cells (K). Data are shown as mean ± SEM (n=3 independent experiments, Two-way ANOVA, **, $p = 0.0050$).

(L, M) CCND3 stability analysis. A parallel assay showed that CCND3 degradation was repressed in *Akt3*-transfected cells. Data are shown as mean ± SEM (n=3 independent experiments, Two-way ANOVA, *, $p = 0.0271$).

(N, O) Western blot analysis of CCND3 in cultured neurospheres. CCND3 was significantly reduced in *Akt* cTKO neurospheres at DIV3 compared to controls. GAPDH served as a loading control. Data are shown as mean ± SEM (n=3 independent experiments, Student's *t* test, **, $p = 0.0013$).

(P, Q) CCND3 overexpression in *Akt* cTKO neurospheres. These cells infected with lentivirus expressing CCND3 or GFP for 2 days were selected by blasticidin and collected at day3 for Western blot (P). CCND3 expression was significantly increased in neurospheres overexpressing CCND3 compared to controls (Q). Data are shown as mean ± SEM (n=3 independent experiments, Student's *t* test, **, $p = 0.0014$).

(R, S) The neurosphere size. *Akt* cTKO neurospheres were infected with lentivirus expressing CCND3 and then selected by blasticitin. The diameter of *Akt* cTKO neurospheres overexpressed with CCND3 was comparable compared to that infected with GFP (S). Scale bar: 100 μm. Data are shown as mean ± SEM (n=3 independent experiments, Student's *t* test, n.s., p = 0.9761).

(T) CCND2 ubiquitination assay under denaturing immunoprecipitation conditions. 293T cells were transfected with Flag-tagged CCND2 and HA-tagged Ub and treated with 10 μM MG132 for 4 hrs. IP was performed using Flag antibody. Higher levels of HA-tagged Ub were detected in the precipitated CCND2 from MG132-treated cells compared to untreated cells.

(U) The examination of CDK4 ubiquitination. 293T cells were co-transfected with Flag-tagged CCND2 and HA-tagged Ub and treated with 10 μM MG132 for 4 hrs. IP was preformed using Flag antibody. While CDK4 and HA-tagged Ub were co-precipitated with CCND2, no higher molecular weight ubiquitinated form of CDK4 was detected. The circle indicates non-specific bands.

(V) CCND2 ubiquitination assay in neurospheres. The cell lysates were prepared form control and *Akt* cTKO neurospheres at DIV3. IP was preformed using anti-CCND2 antibody. The levels of Ub were higher in the precipitated CCND2 from *Akt* cTKO neurospheres than controls. GAPDH served as a loading control.

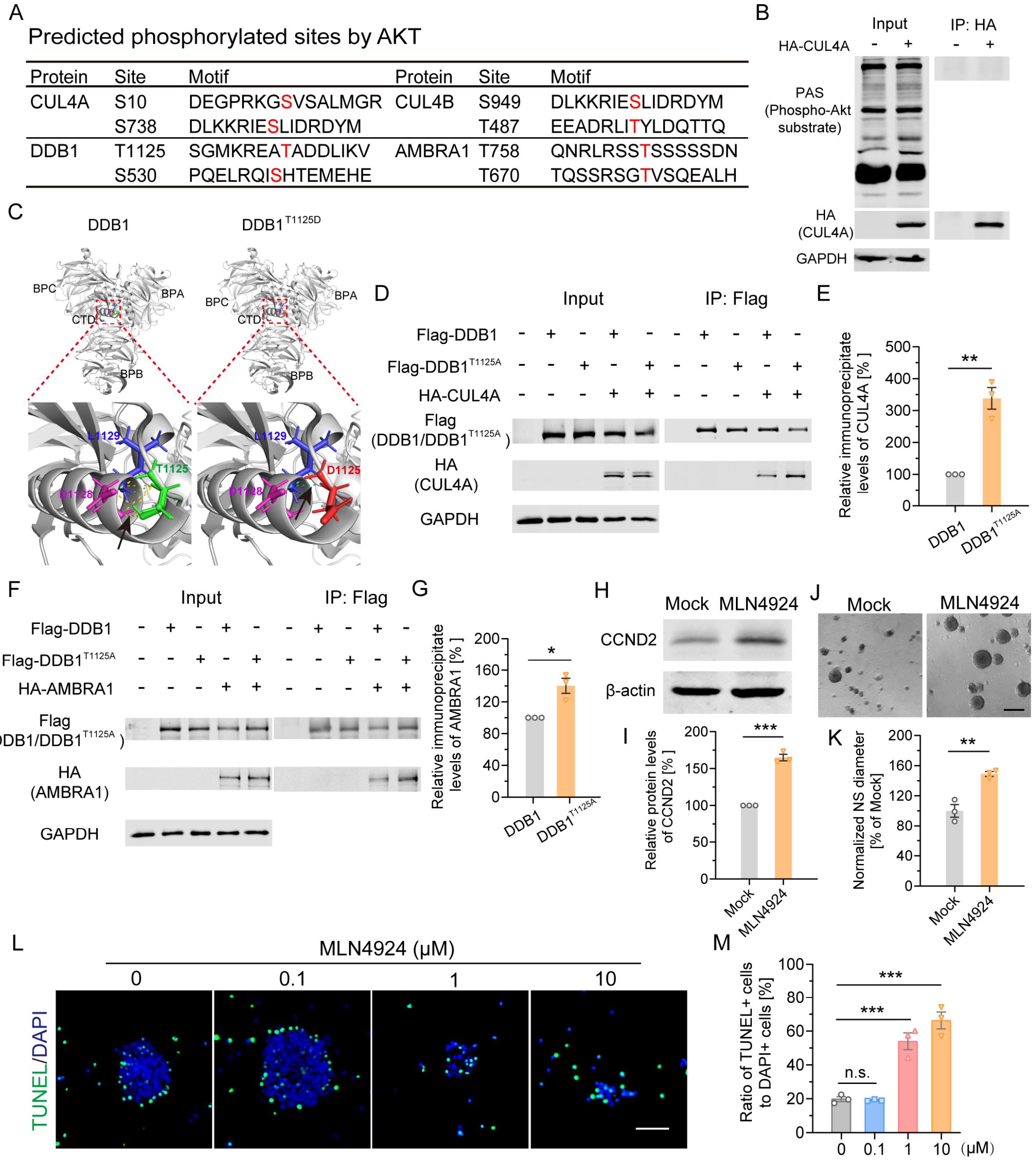

Appendix Figure S7. Phosphorylation of DDB1 changes its interaction with components of CRL4^{AMBRA1}.

(A) The predicted AKT-phosphorylated sites and motifs by SCANSITE4.0. Two sites on these proteins were listed in the table.

(B) IP assay using HA antibody in 293 cells transfected with HA-tagged CUL4A. Cell lysates were immunoprecipitated by HA antibody, and the precipitated samples were analyzed by Western blot using PAS and HA antibodies. The results showed that the precipitated protein was recognized by HA antibody but not by PAS antibody.

(C) Structural prediction of DDB1 and DDB1^{T1125D}. The structure of DDB1 was molded using

AlphaFold, and DDB1^{T1125D} was generated based on this structure using SWISS-MODEL. The contacts of T1125 or D1125 with other amino acids were analyzed by PyMOL. T1125 forms more hydrogen bonds (indicated by black arrow) with neighboring amino acids than D1125. BPA/BPB/BPC, β-propeller A-C; CTD, C-terminal helical domain.

(D, E) The interaction analysis between DDB1^{T1125A} and CUL4A by Co-IP assay. 293T cells were co-transfected with HA-tagged CUL4A and Flag-tagged DDB1 or DDB1^{T1125A}. IP was performed with anti-Flag antibody. The precipitated protein was detected by Western blot using Flag and HA antibodies (D). The DDB1^{T1125A} mutant bound significantly more CUL4A than WT DDB1 (E). Data are shown as mean ± SEM (n=3 independent experiments, Student *t* test, **, $p = 0.0022$).

(F, G) Co-IP analysis of DDB1^{T1125A} with AMBRA1. A parallel assay was performed with HA-tagged AMBRA1. The protein levels of AMBRA1 was precipitated by DDB1^{T1125A} were significant higher than that by DDB1 (G). Data are shown as mean ± SEM (n=3 independent experiments, Student *t* test, *, $p = 0.0132$).

(H, I) Western blot analysis for CCND2. Neurospheres were cultured from the cortices of E15.5 *Akt* cTKO mice. MLN4924 was added into medium 1 day after cell seeding and neurospheres were collected for Western blot 6 days after treatment. The protein levels of CCND2 were significantly increased in the MLN4924-treated *Akt* cTKO neurospheres. Data are shown as mean ± SEM (n=3 independent experiments, Student *t* test, ***, $p = 0.0001$).

(J, K) The neurosphere size. *Akt* cTKO neurospheres were captured 6 days after 0.1μM MLN4924 treatment. The size of *Akt* cTKO neurospheres treated with MLN4924 was significantly increased compared to non-treated cells. The scale bar is 100μm. Data are shown as mean ± SEM (n=3 independent experiments, Student *t* test, **, $p = 0.0054$).

(L, M) TUNEL assay in neurospheses treated with MLN4924. Neurospheres derived from E15.5 mouse cortex were treated with 0.1, 1 or 10 μM MLN4924 for 6 days. These cells were transferred to poly-L-ornithine/laminin-coated coverslips for 24 hrs prior to analysis (L). The number of TUNEL+ cells was significantly increased at 1 μM and 10 μM MLN4924 compared to mock- treated controls, but not at 0.1 μM (M). The scale bar is 50 μm. Data are shown as mean ± SEM (n=3 independent experiments, One-way ANOVA, n.s., $p = 9989$, ***, $p = 0.0007$ for 1 μM, $p < 0.0001$ for 10 μM).
