# Supplementary figures and images for: CRL4AMBRA1 is a key mediator for AKT-dependent cell cycle control in neural progenitor cells (part 1 of 2)
Source: EMBO Rep. 2026 Apr 27;27(11):3099–119. doi: 10.1038/s44319-026-00768-7 (PMC13260899; doi:10.1038/s44319-026-00768-7)

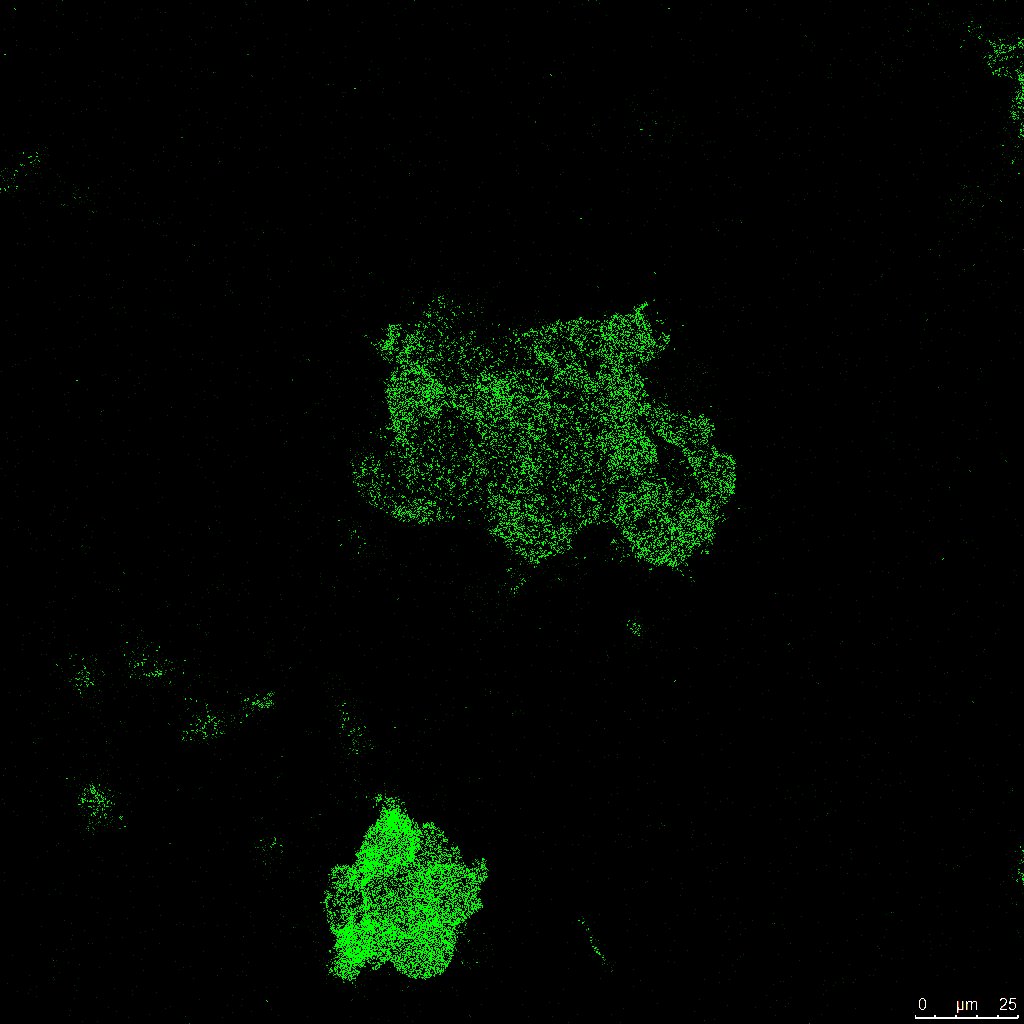

Supplement: Supplementary file 3 — Source data Fig. 1 [file 44319_2026_768_MOESM3_ESM.zip › Figure 1/1A/AKT.jpg]

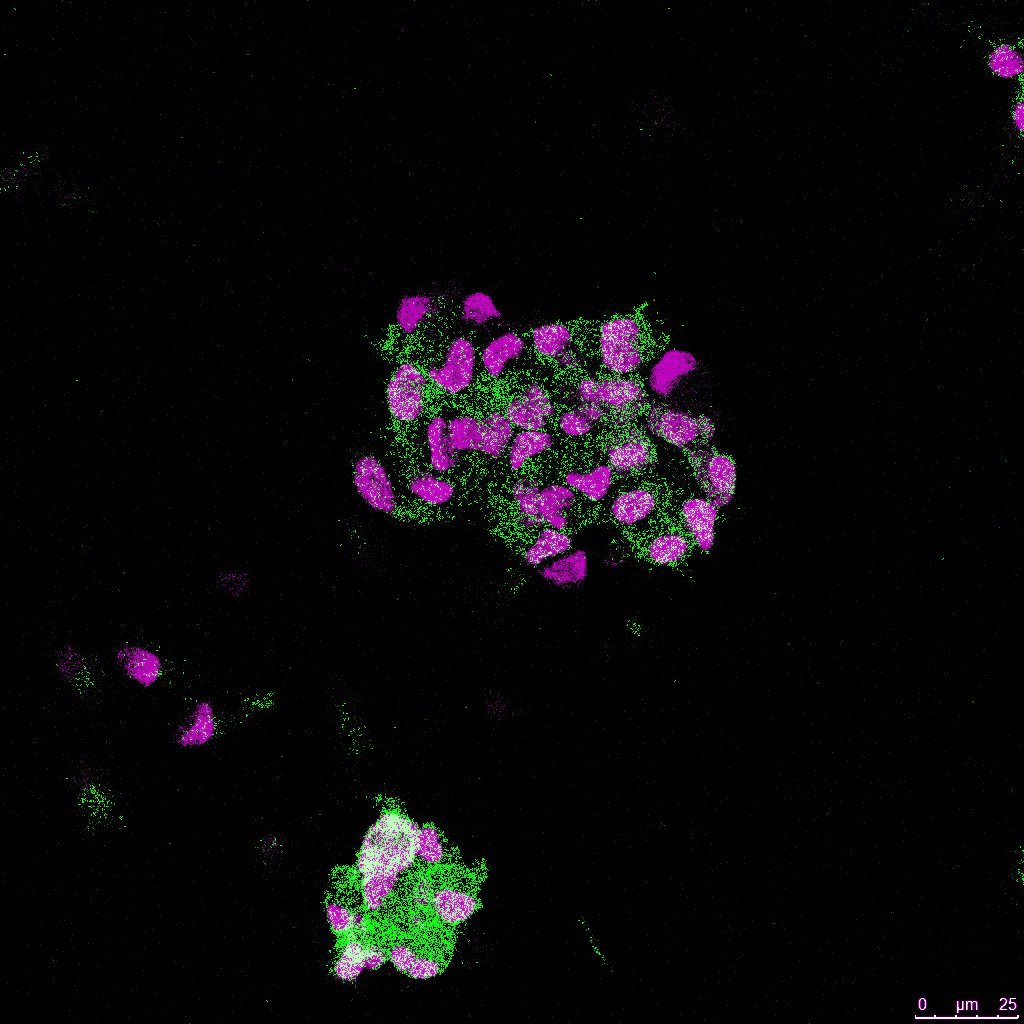

Supplement: Supplementary file 3 — Source data Fig. 1 [file 44319_2026_768_MOESM3_ESM.zip › Figure 1/1A/merge.jpg]

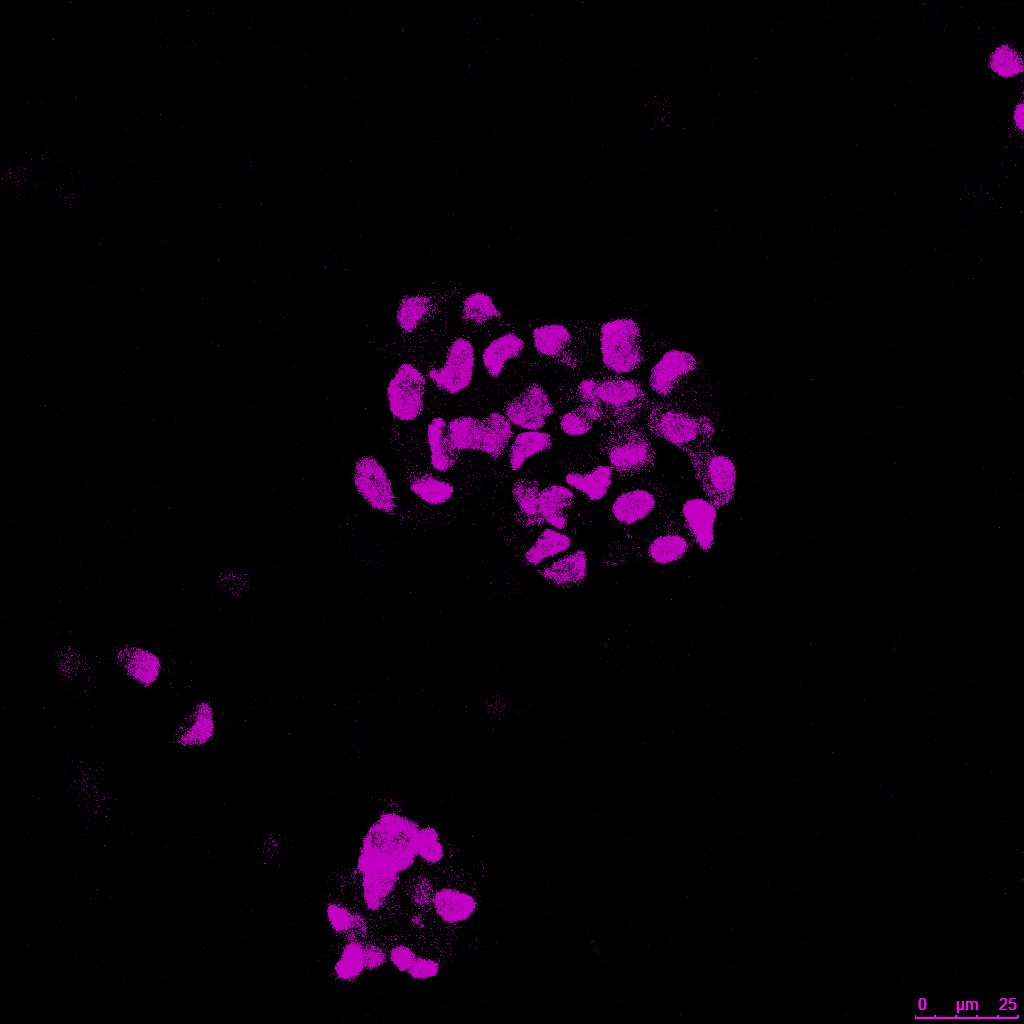

Supplement: Supplementary file 3 — Source data Fig. 1 [file 44319_2026_768_MOESM3_ESM.zip › Figure 1/1A/SOX2.jpg]

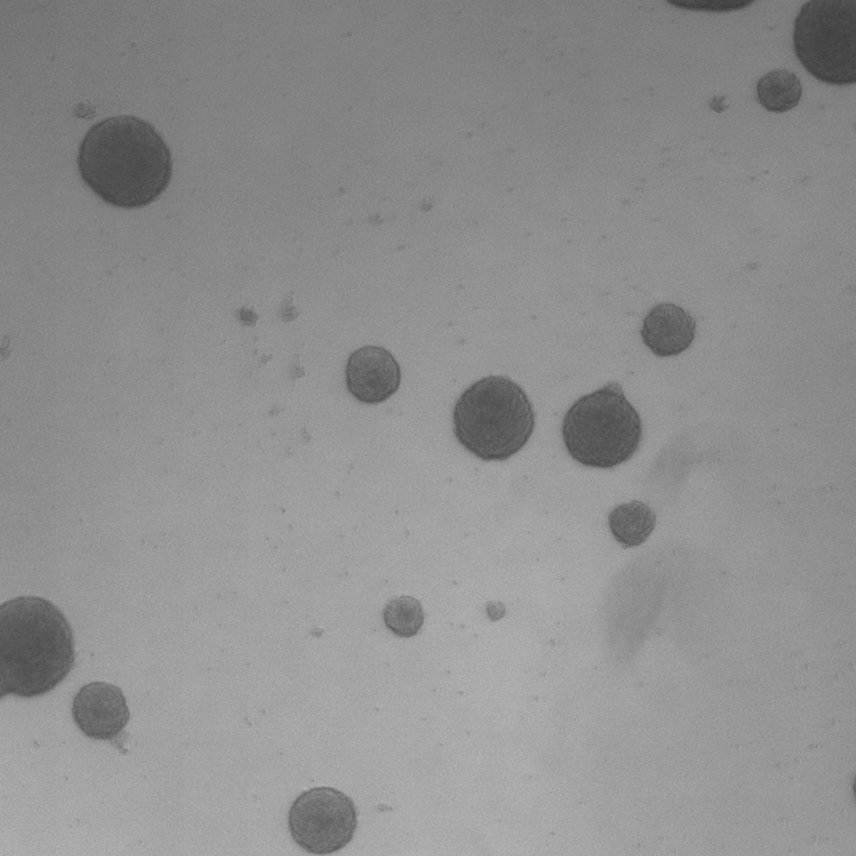

Supplement: Supplementary file 3 — Source data Fig. 1 [file 44319_2026_768_MOESM3_ESM.zip › Figure 1/1B/B-Cap.jpg]

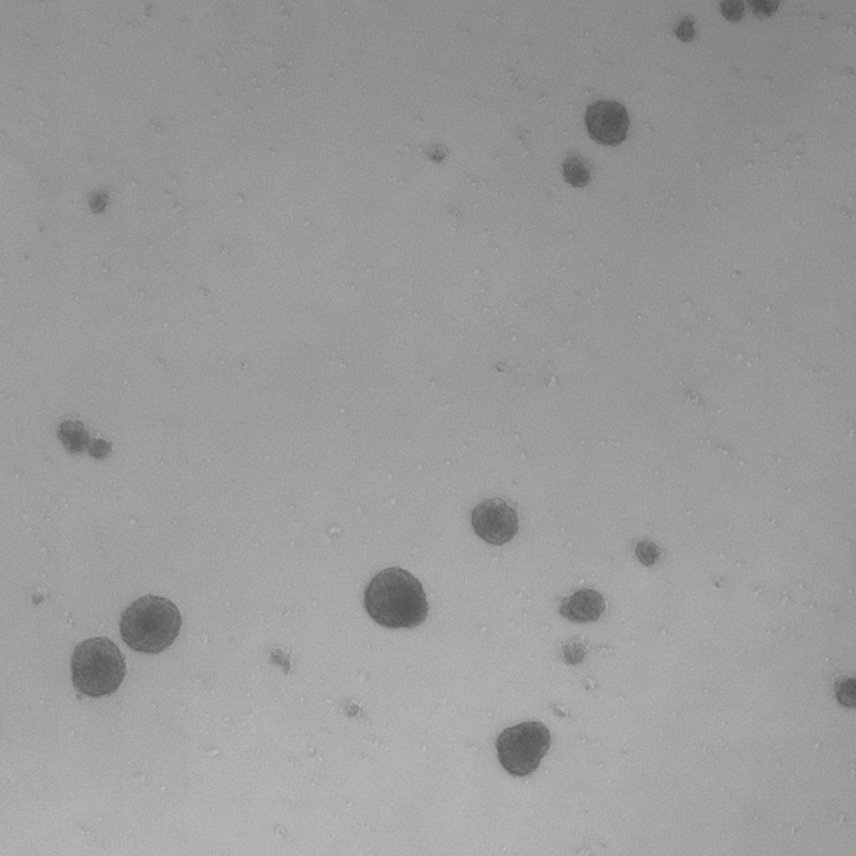

Supplement: Supplementary file 3 — Source data Fig. 1 [file 44319_2026_768_MOESM3_ESM.zip › Figure 1/1B/B-MK.jpg]

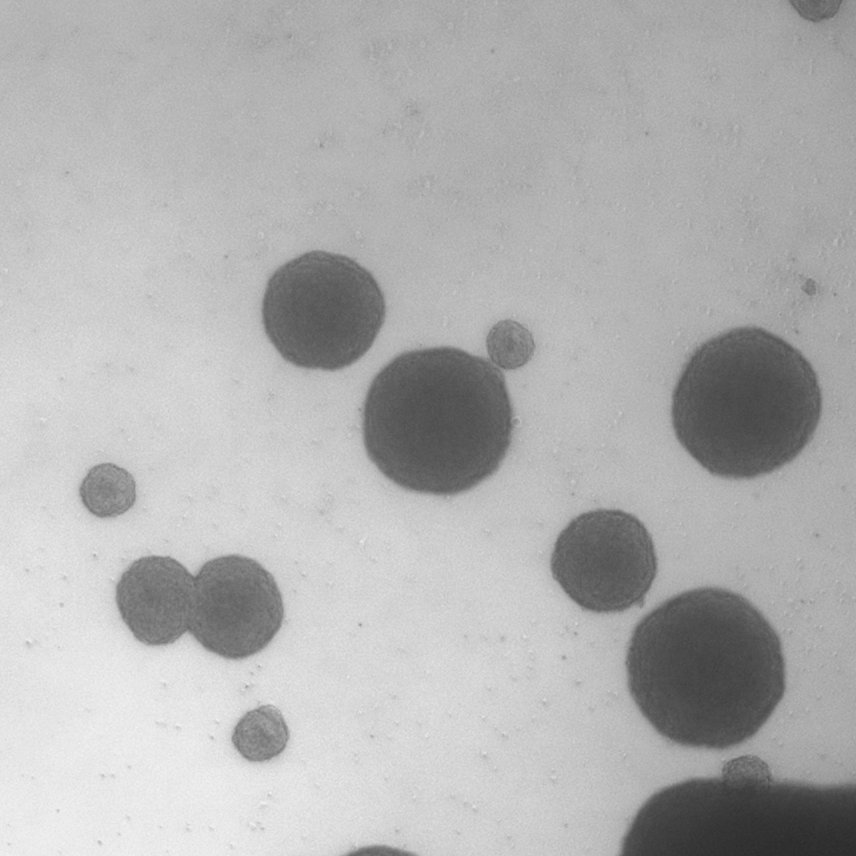

Supplement: Supplementary file 3 — Source data Fig. 1 [file 44319_2026_768_MOESM3_ESM.zip › Figure 1/1B/B-Mock.jpg]

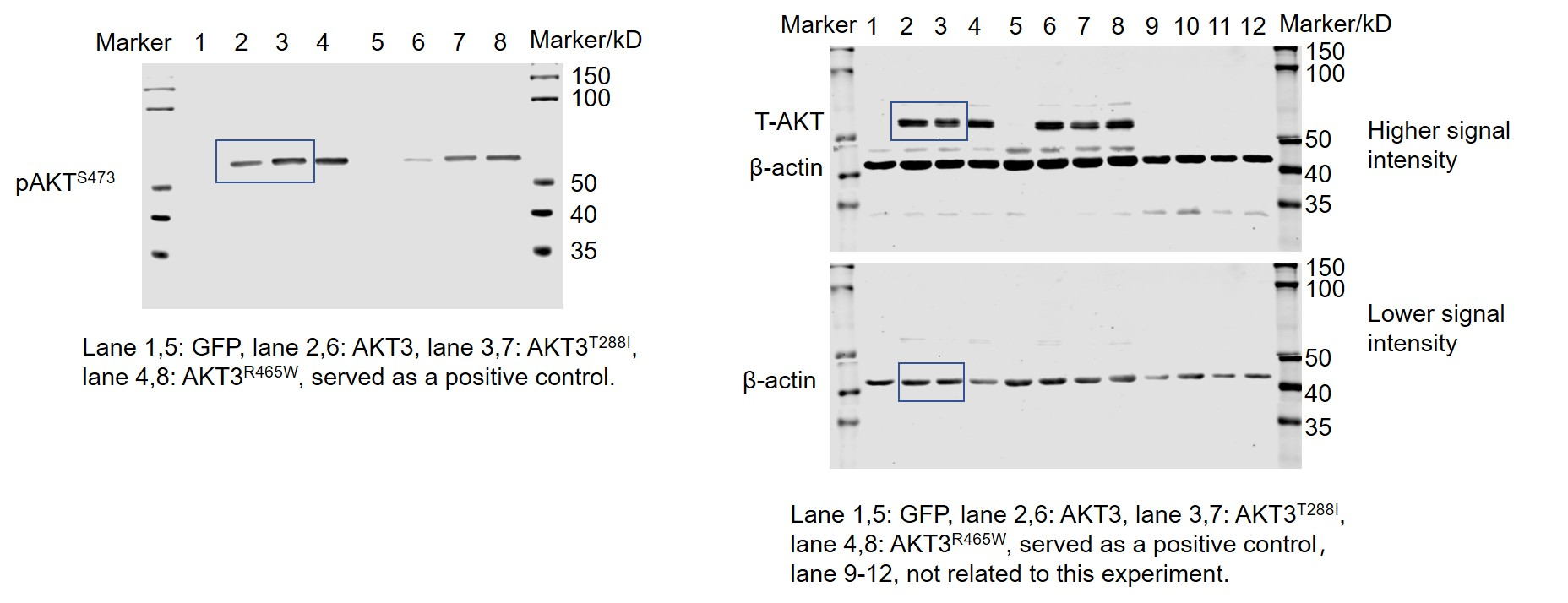

Supplement: Supplementary file 3 — Source data Fig. 1 [file 44319_2026_768_MOESM3_ESM.zip › Figure 1/1E/1E.tif]

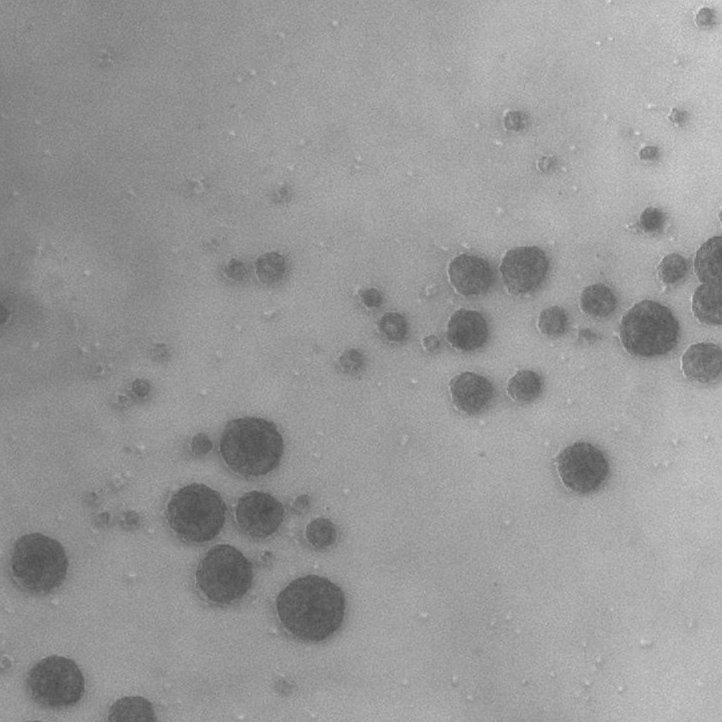

Supplement: Supplementary file 3 — Source data Fig. 1 [file 44319_2026_768_MOESM3_ESM.zip › Figure 1/1G/G-AKT3.jpg]

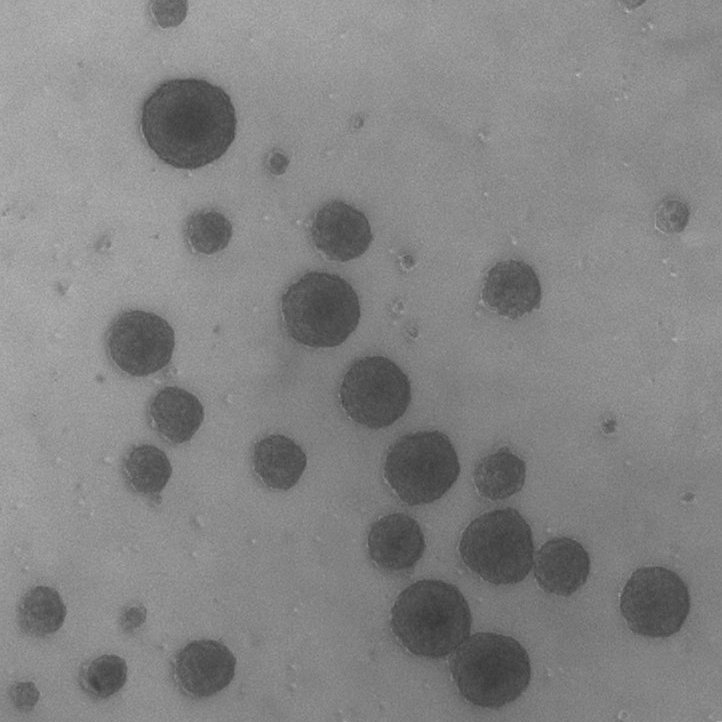

Supplement: Supplementary file 3 — Source data Fig. 1 [file 44319_2026_768_MOESM3_ESM.zip › Figure 1/1G/G-T288I.jpg]

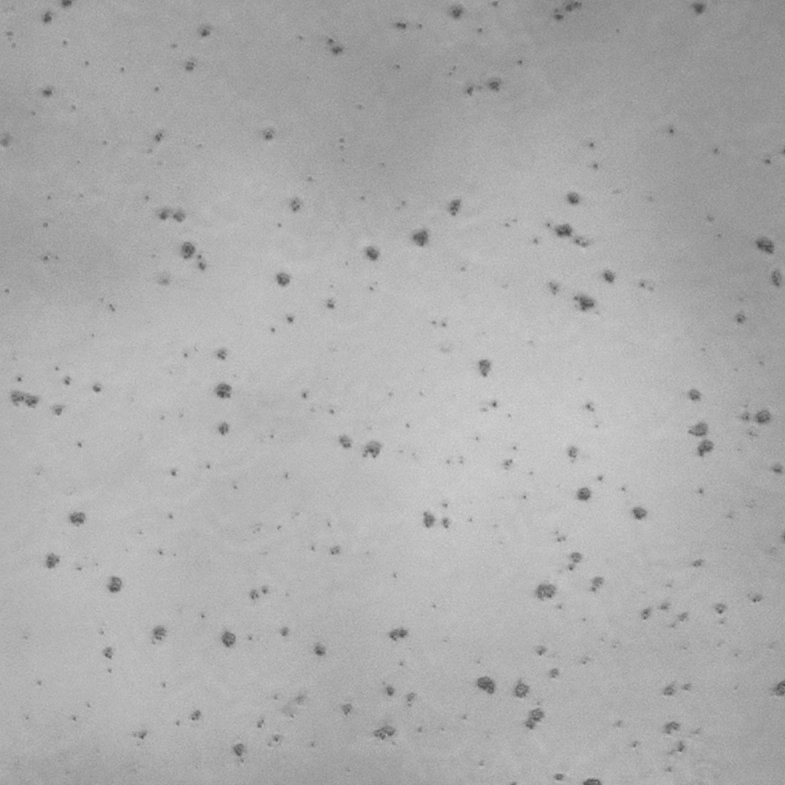

Supplement: Supplementary file 3 — Source data Fig. 1 [file 44319_2026_768_MOESM3_ESM.zip › Figure 1/1I/cTKO-DIV3.jpg]

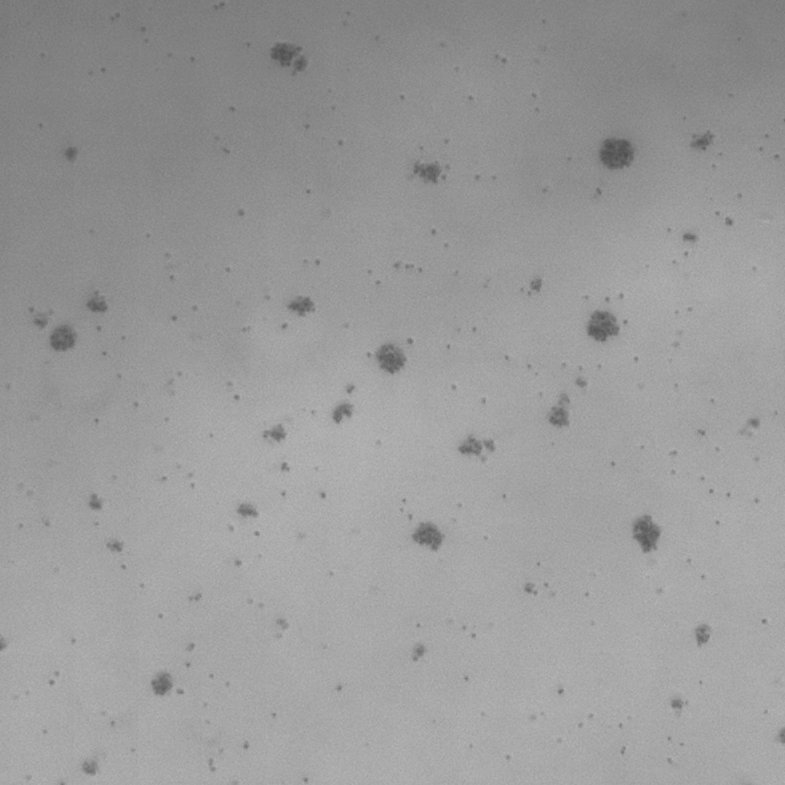

Supplement: Supplementary file 3 — Source data Fig. 1 [file 44319_2026_768_MOESM3_ESM.zip › Figure 1/1I/cTKO-DIV5.jpg]

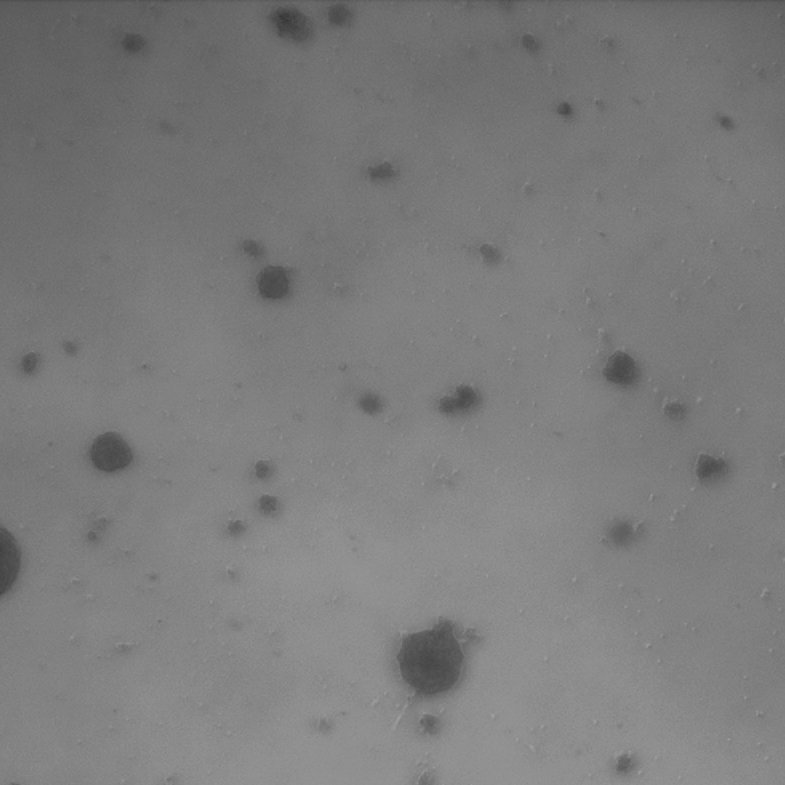

Supplement: Supplementary file 3 — Source data Fig. 1 [file 44319_2026_768_MOESM3_ESM.zip › Figure 1/1I/cTKO-DIV7.jpg]

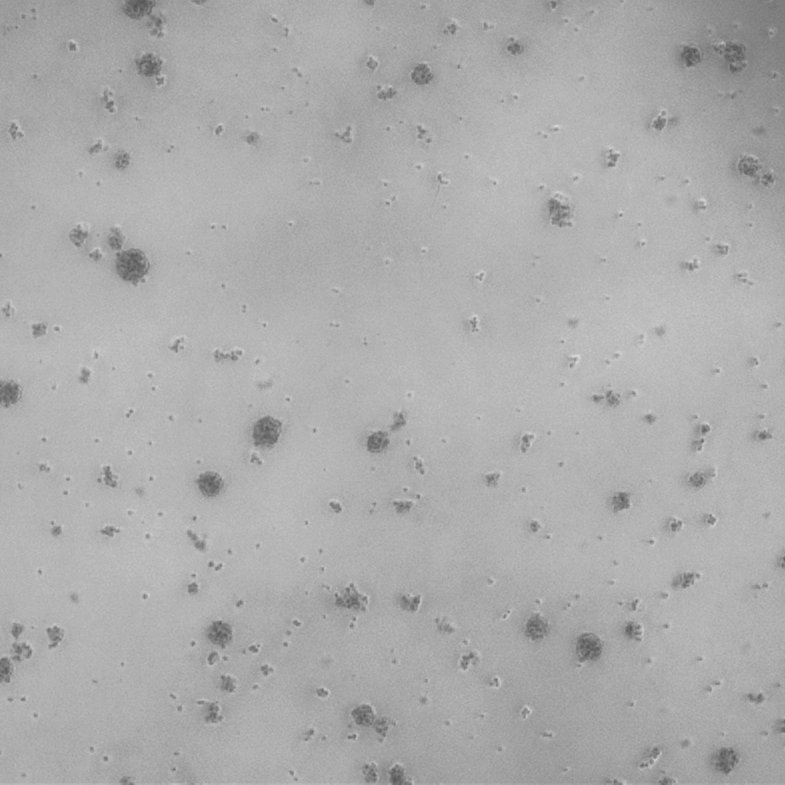

Supplement: Supplementary file 3 — Source data Fig. 1 [file 44319_2026_768_MOESM3_ESM.zip › Figure 1/1I/ctrl-DIV3.jpg]

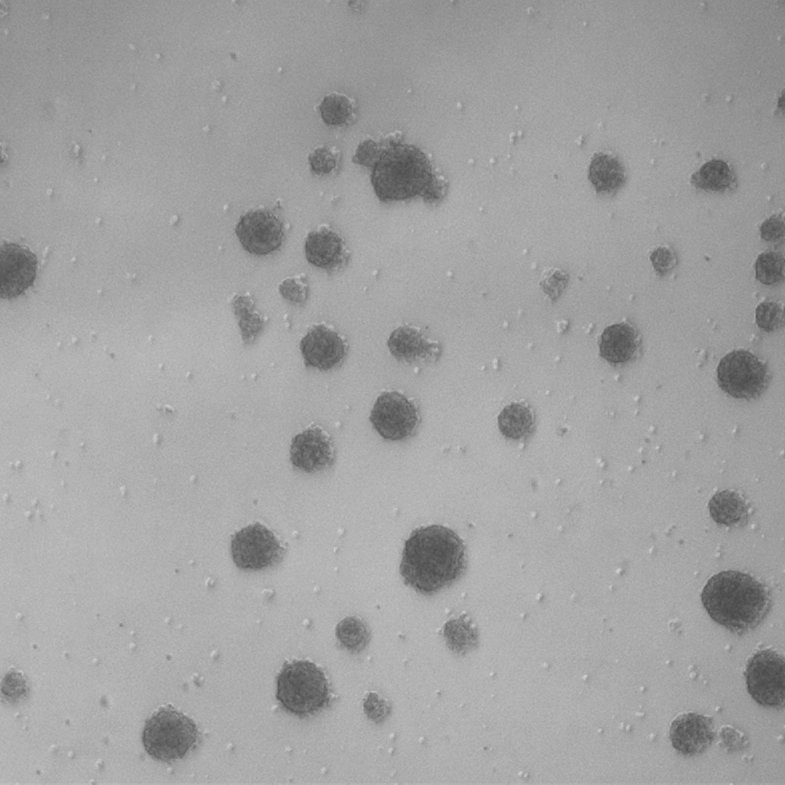

Supplement: Supplementary file 3 — Source data Fig. 1 [file 44319_2026_768_MOESM3_ESM.zip › Figure 1/1I/ctrl-DIV5.jpg]

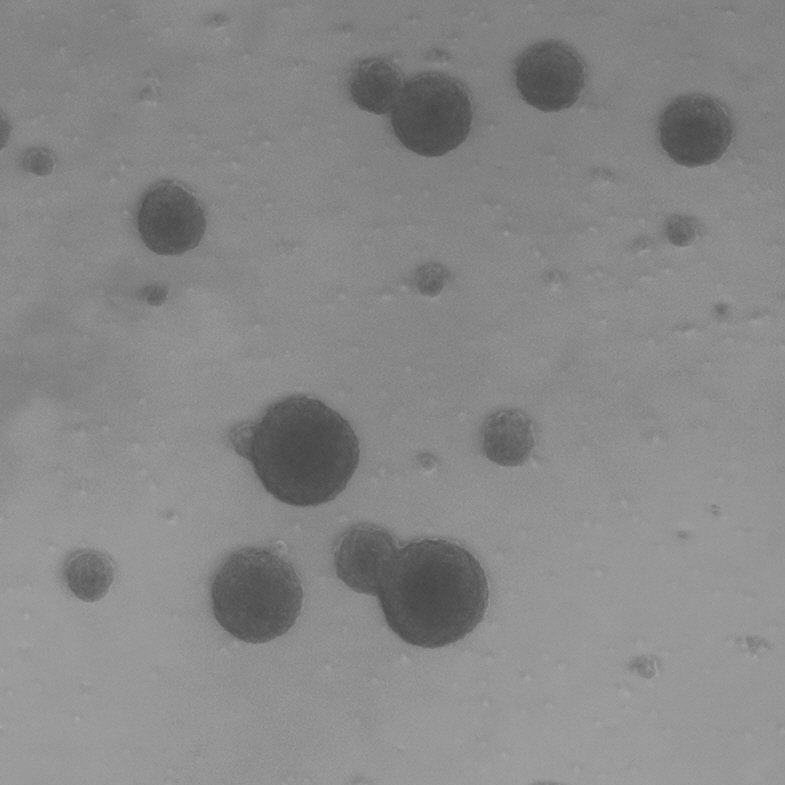

Supplement: Supplementary file 3 — Source data Fig. 1 [file 44319_2026_768_MOESM3_ESM.zip › Figure 1/1I/ctrl-DIV7.jpg]

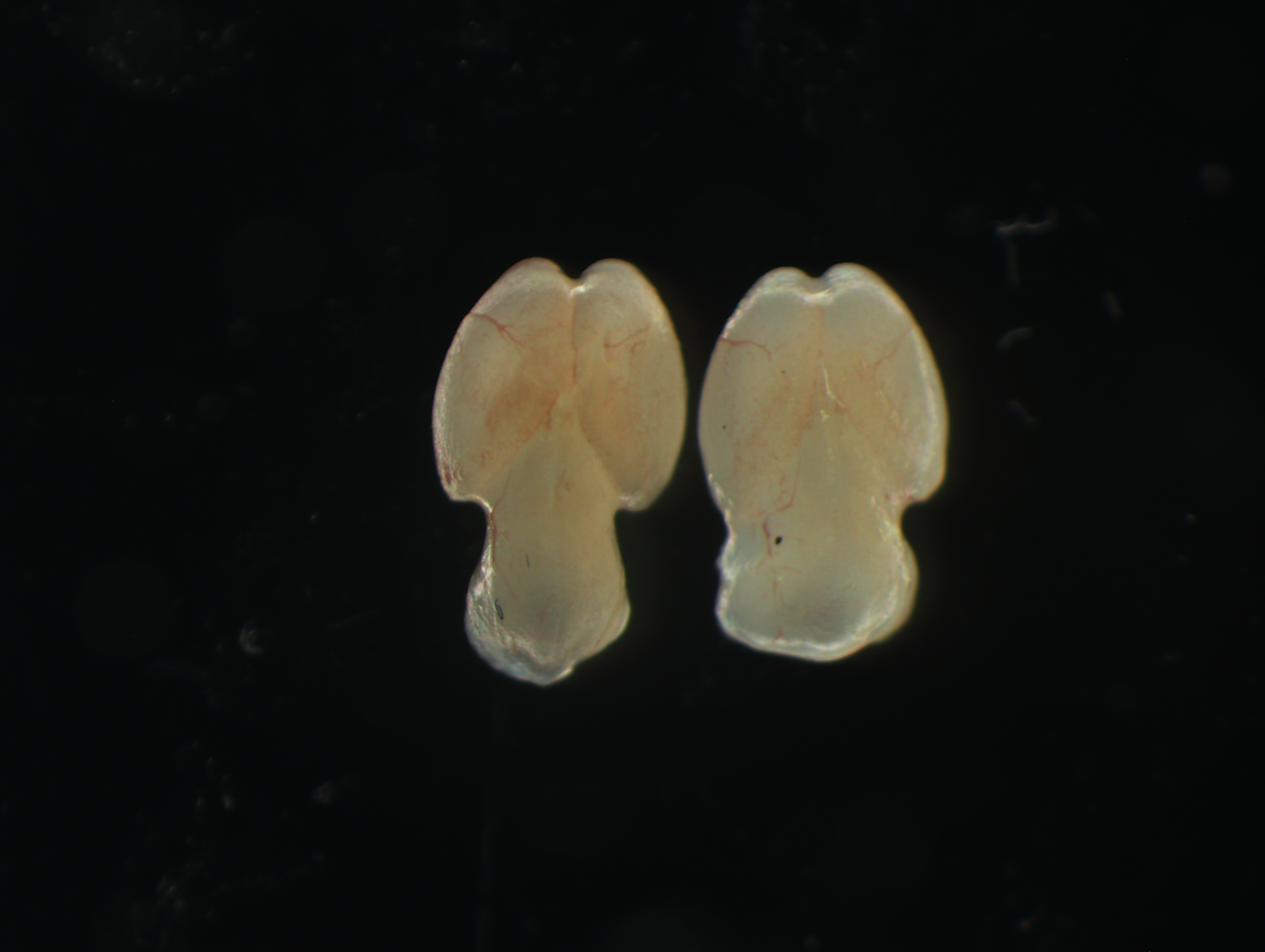

Supplement: Supplementary file 4 — Source data Fig. 2 [file 44319_2026_768_MOESM4_ESM.zip › Figure 2/2A/e13.5.tif]

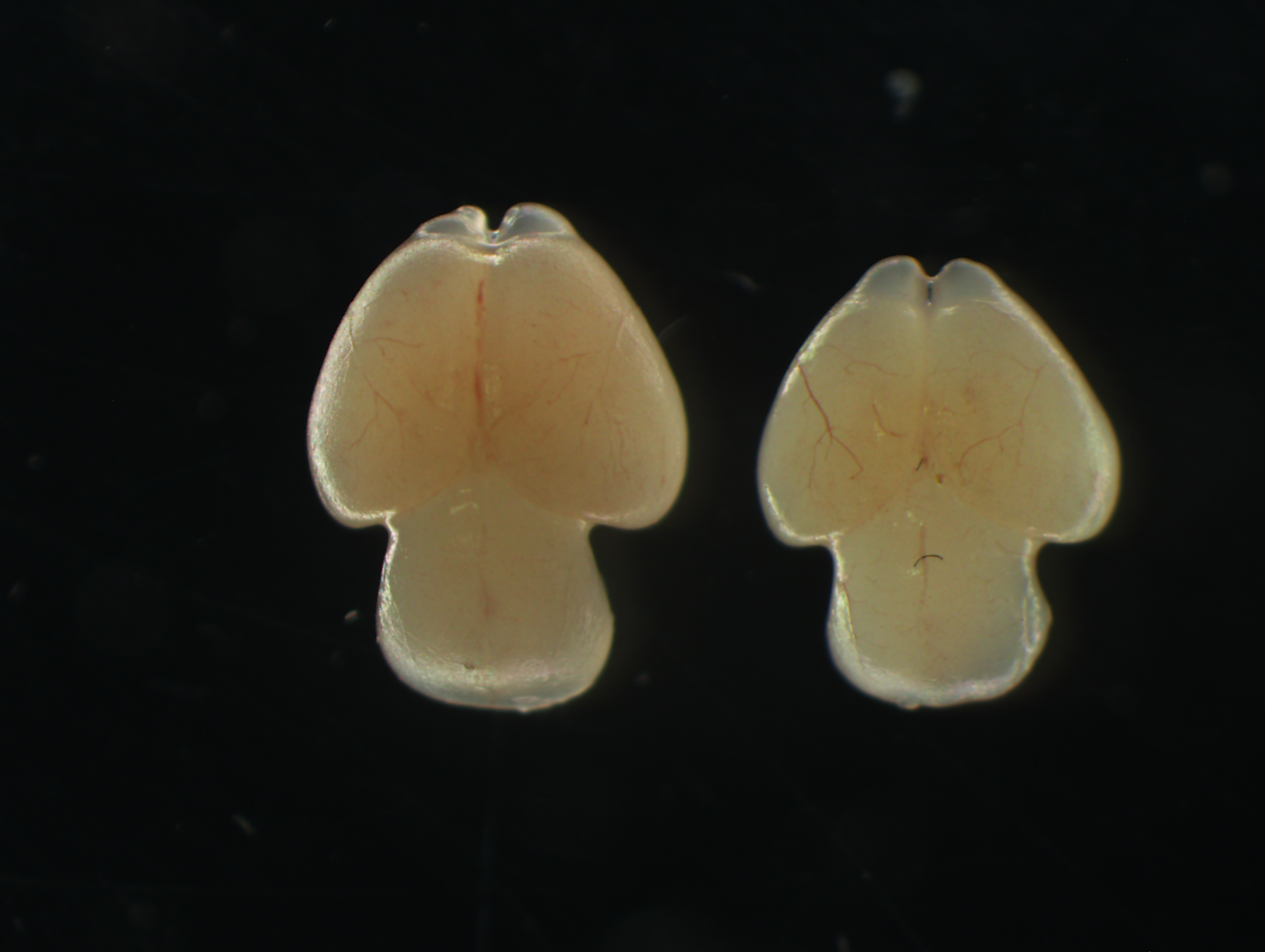

Supplement: Supplementary file 4 — Source data Fig. 2 [file 44319_2026_768_MOESM4_ESM.zip › Figure 2/2A/e15.5.tif]

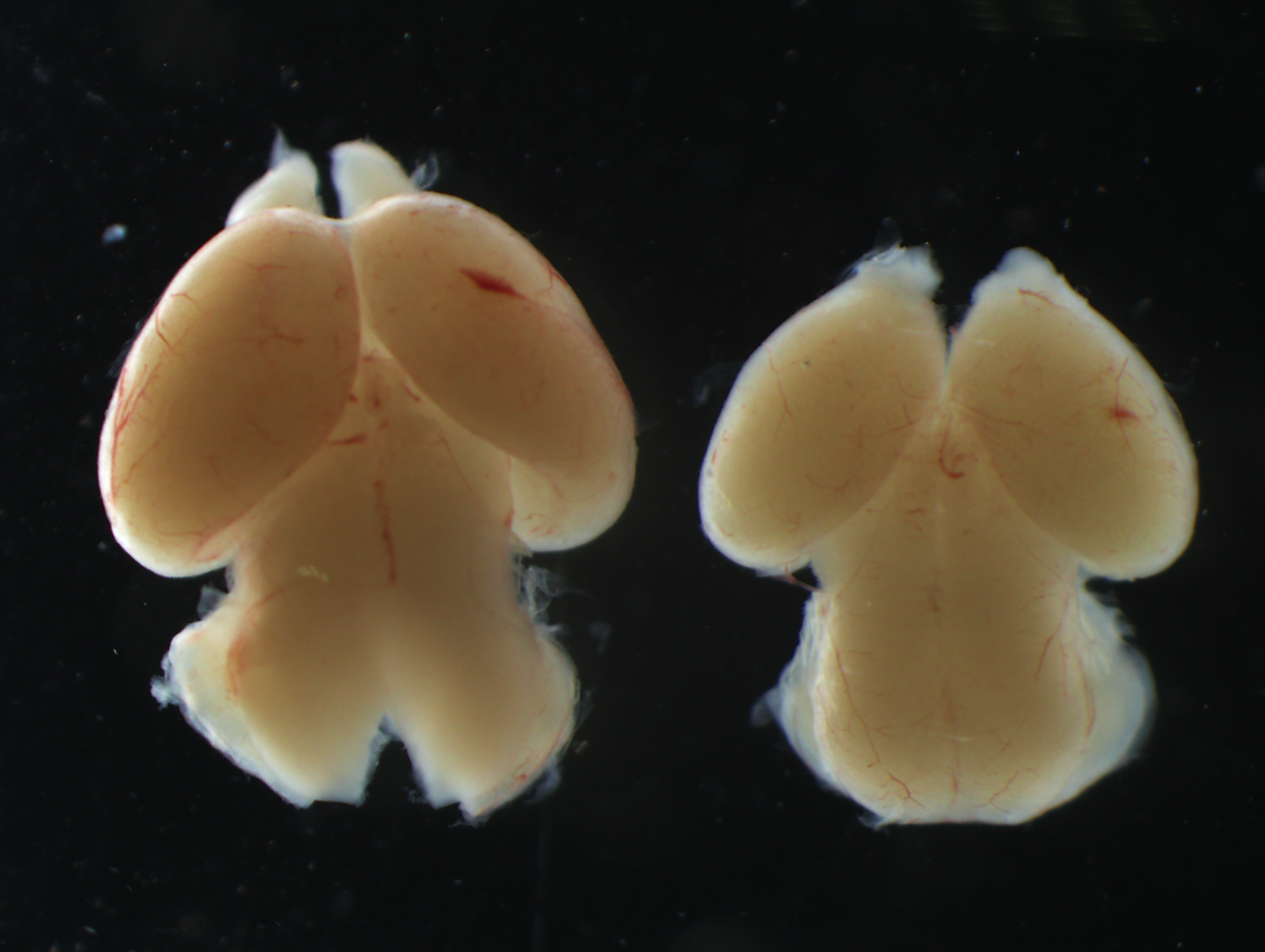

Supplement: Supplementary file 4 — Source data Fig. 2 [file 44319_2026_768_MOESM4_ESM.zip › Figure 2/2A/e17.5.tif]

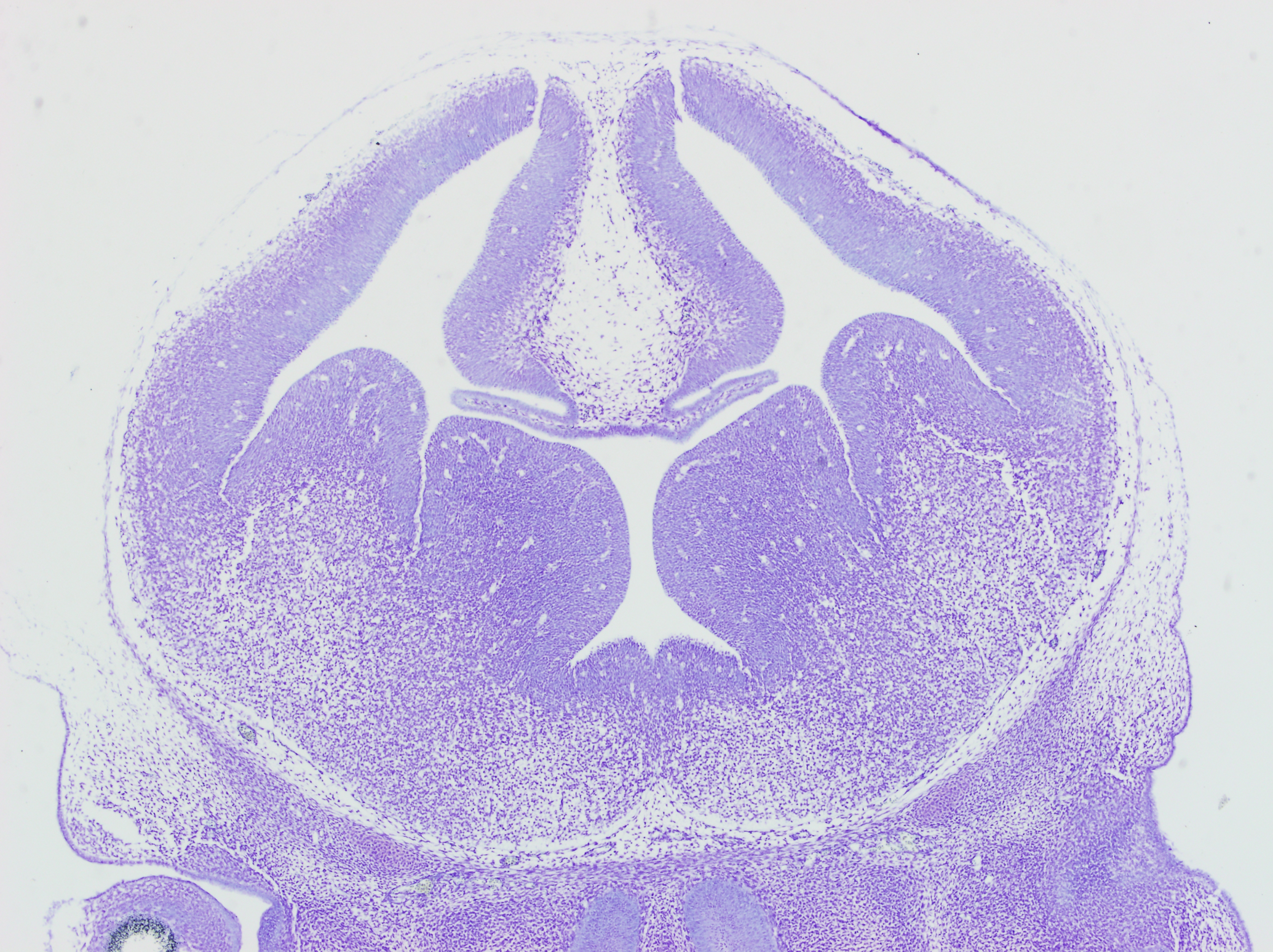

Supplement: Supplementary file 4 — Source data Fig. 2 [file 44319_2026_768_MOESM4_ESM.zip › Figure 2/2B/a.tif]

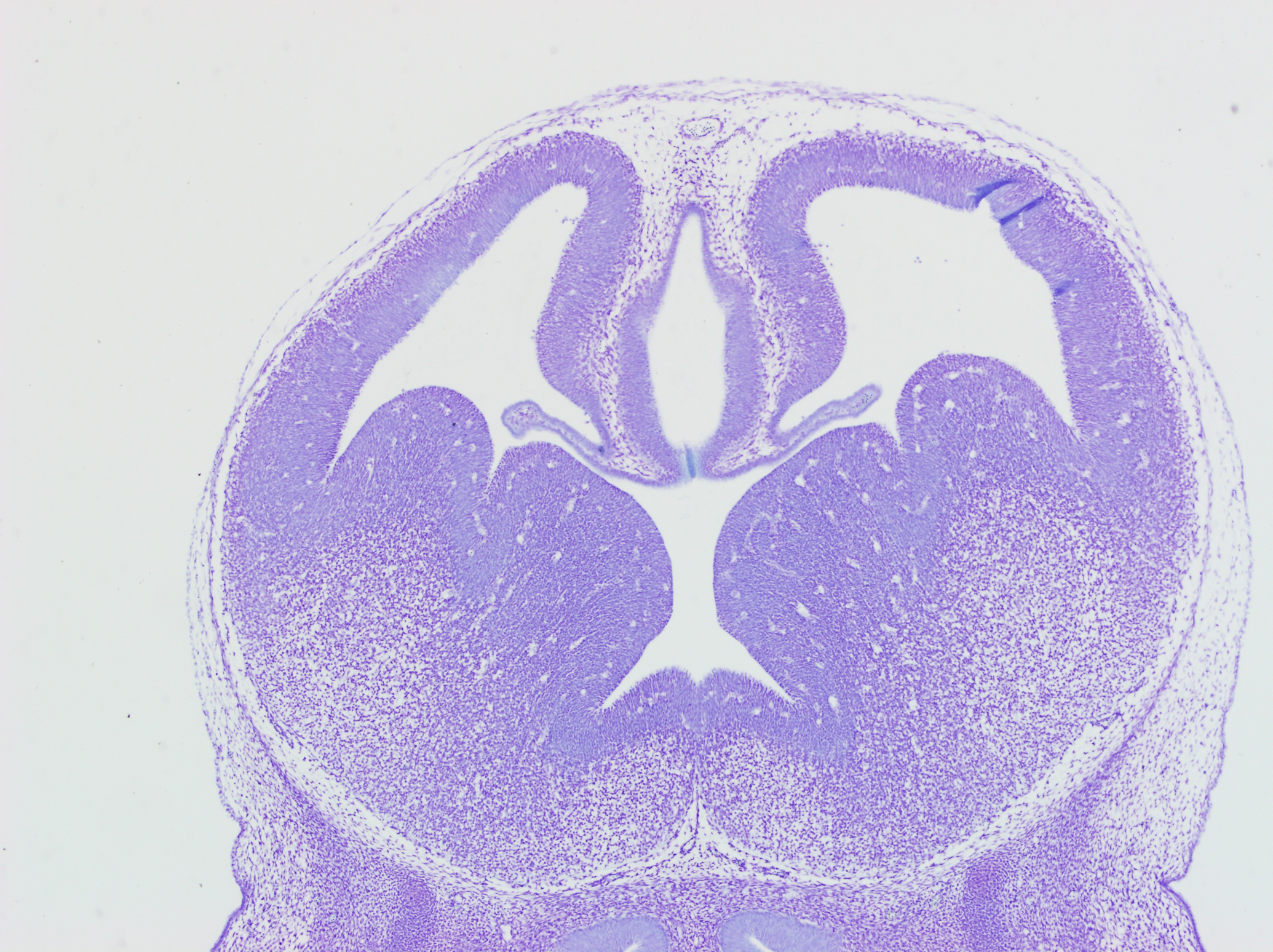

Supplement: Supplementary file 4 — Source data Fig. 2 [file 44319_2026_768_MOESM4_ESM.zip › Figure 2/2B/b.tif]

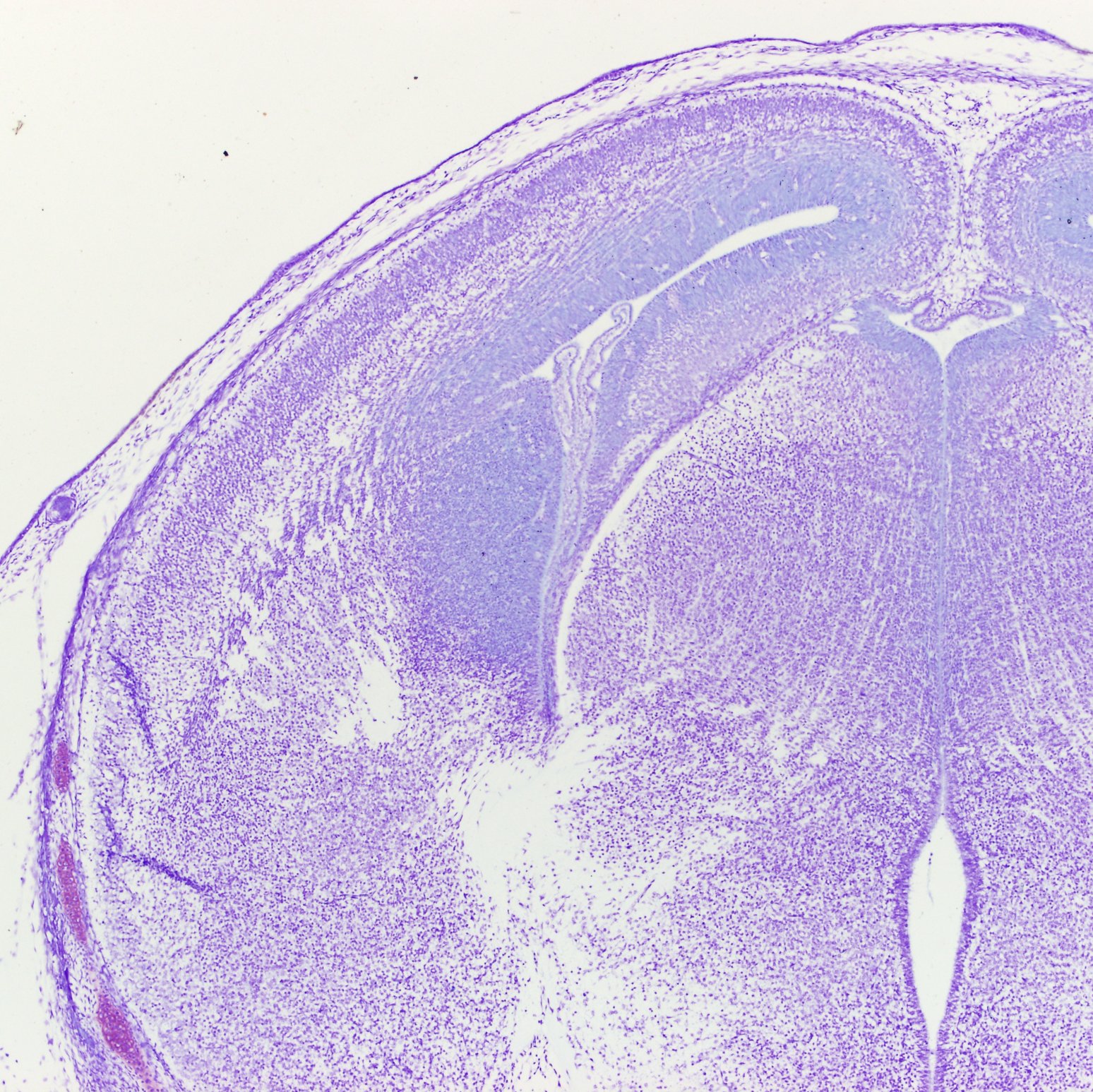

Supplement: Supplementary file 4 — Source data Fig. 2 [file 44319_2026_768_MOESM4_ESM.zip › Figure 2/2B/c.jpg]

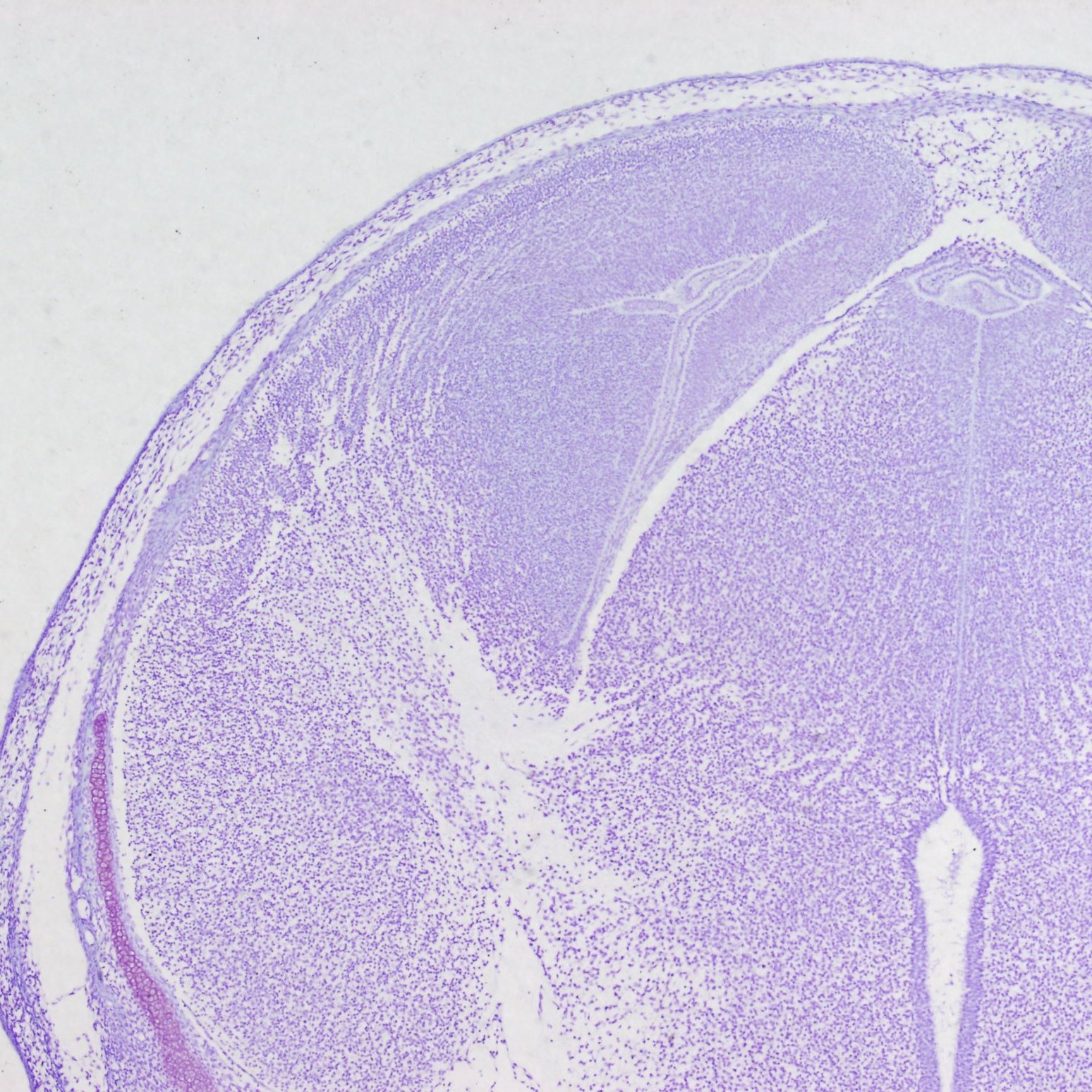

Supplement: Supplementary file 4 — Source data Fig. 2 [file 44319_2026_768_MOESM4_ESM.zip › Figure 2/2B/d.jpg]

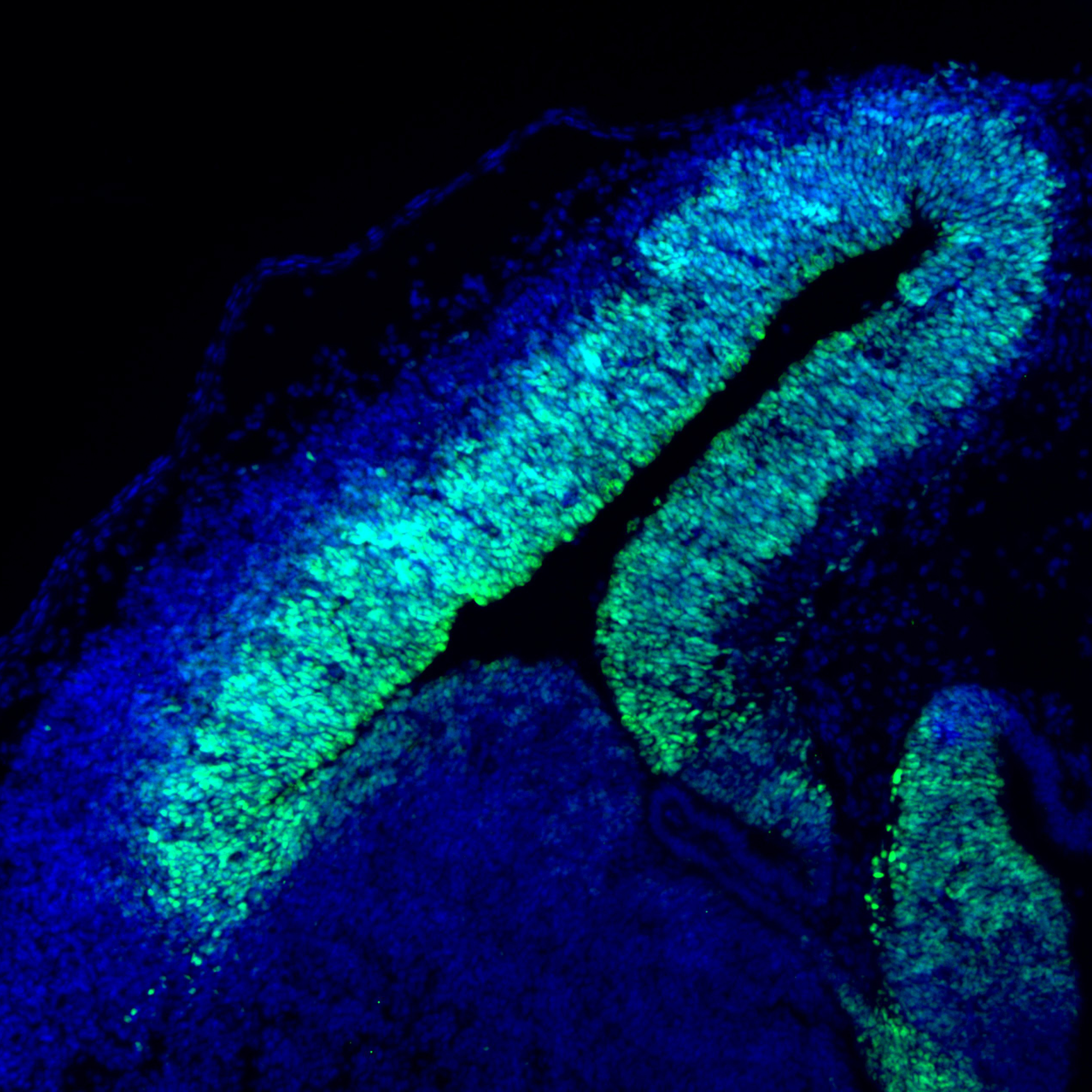

Supplement: Supplementary file 4 — Source data Fig. 2 [file 44319_2026_768_MOESM4_ESM.zip › Figure 2/2D/a.jpg]

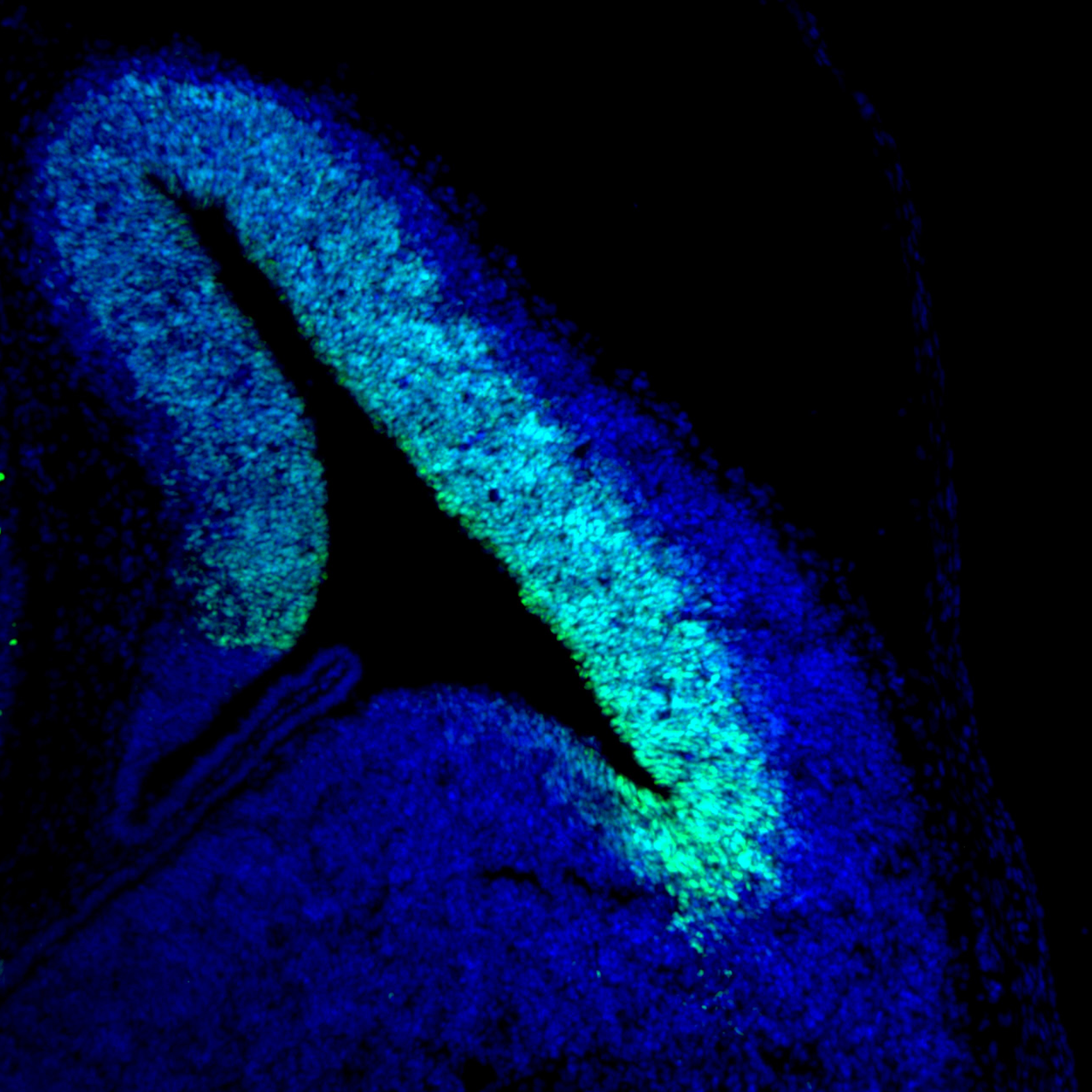

Supplement: Supplementary file 4 — Source data Fig. 2 [file 44319_2026_768_MOESM4_ESM.zip › Figure 2/2D/b.jpg]

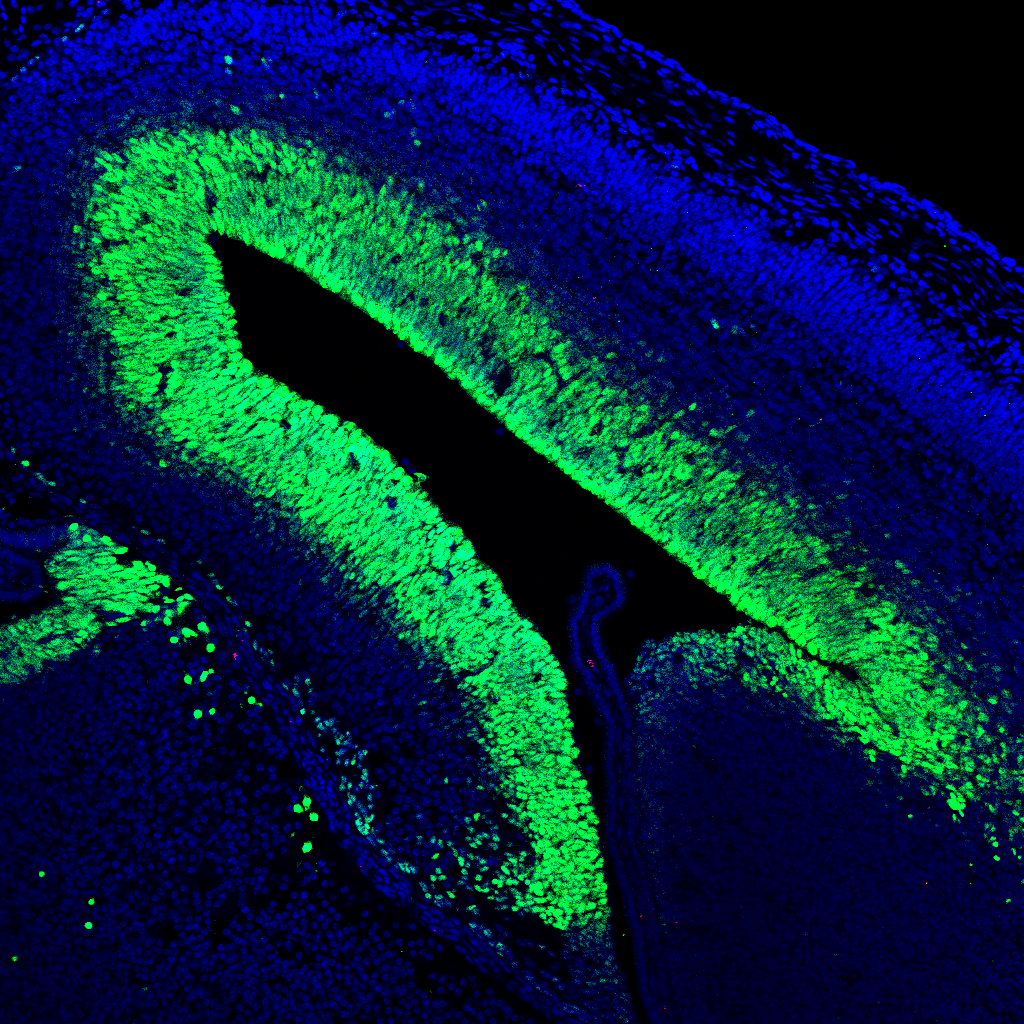

Supplement: Supplementary file 4 — Source data Fig. 2 [file 44319_2026_768_MOESM4_ESM.zip › Figure 2/2D/c.tif]

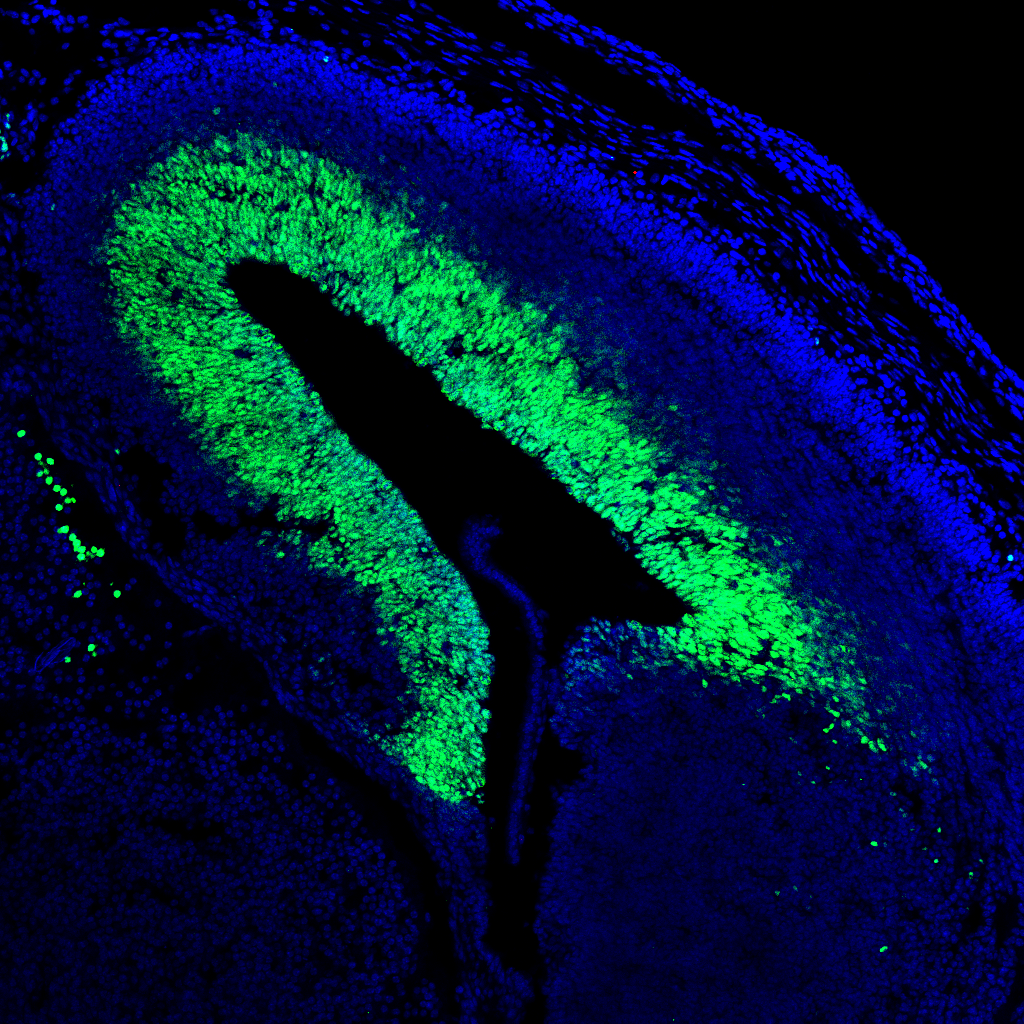

Supplement: Supplementary file 4 — Source data Fig. 2 [file 44319_2026_768_MOESM4_ESM.zip › Figure 2/2D/d.tif]

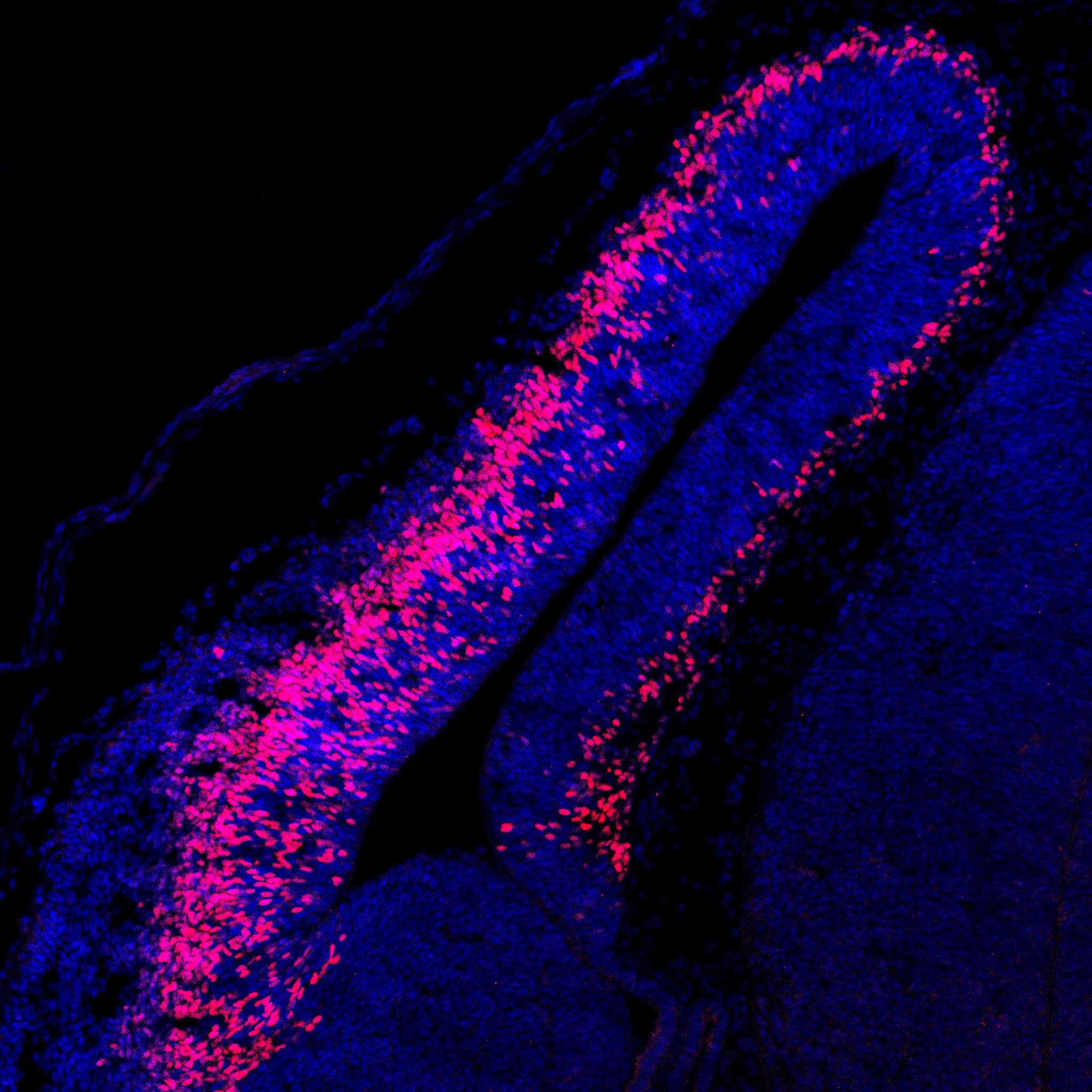

Supplement: Supplementary file 4 — Source data Fig. 2 [file 44319_2026_768_MOESM4_ESM.zip › Figure 2/2E/a.jpg]

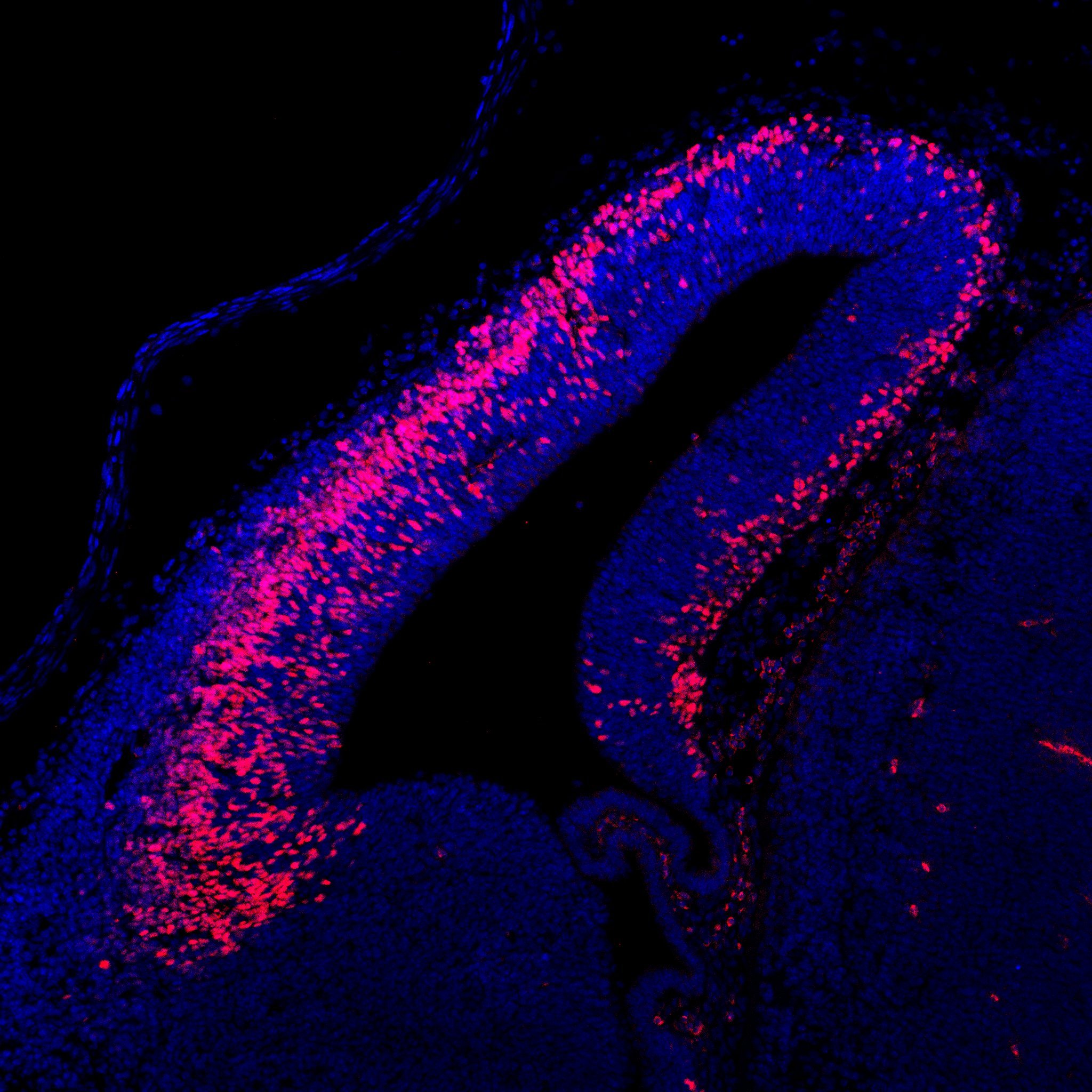

Supplement: Supplementary file 4 — Source data Fig. 2 [file 44319_2026_768_MOESM4_ESM.zip › Figure 2/2E/b.jpg]

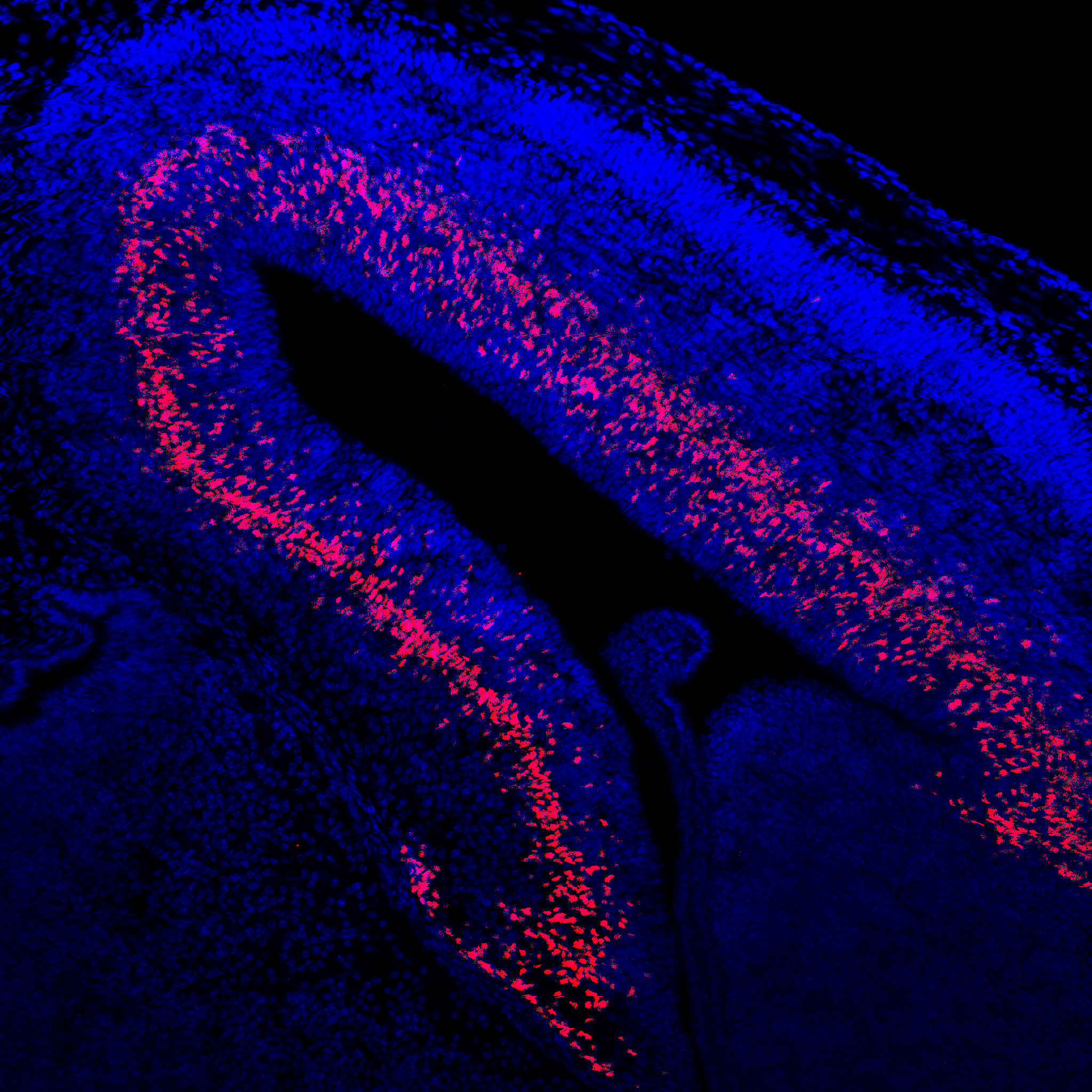

Supplement: Supplementary file 4 — Source data Fig. 2 [file 44319_2026_768_MOESM4_ESM.zip › Figure 2/2E/c.tif]

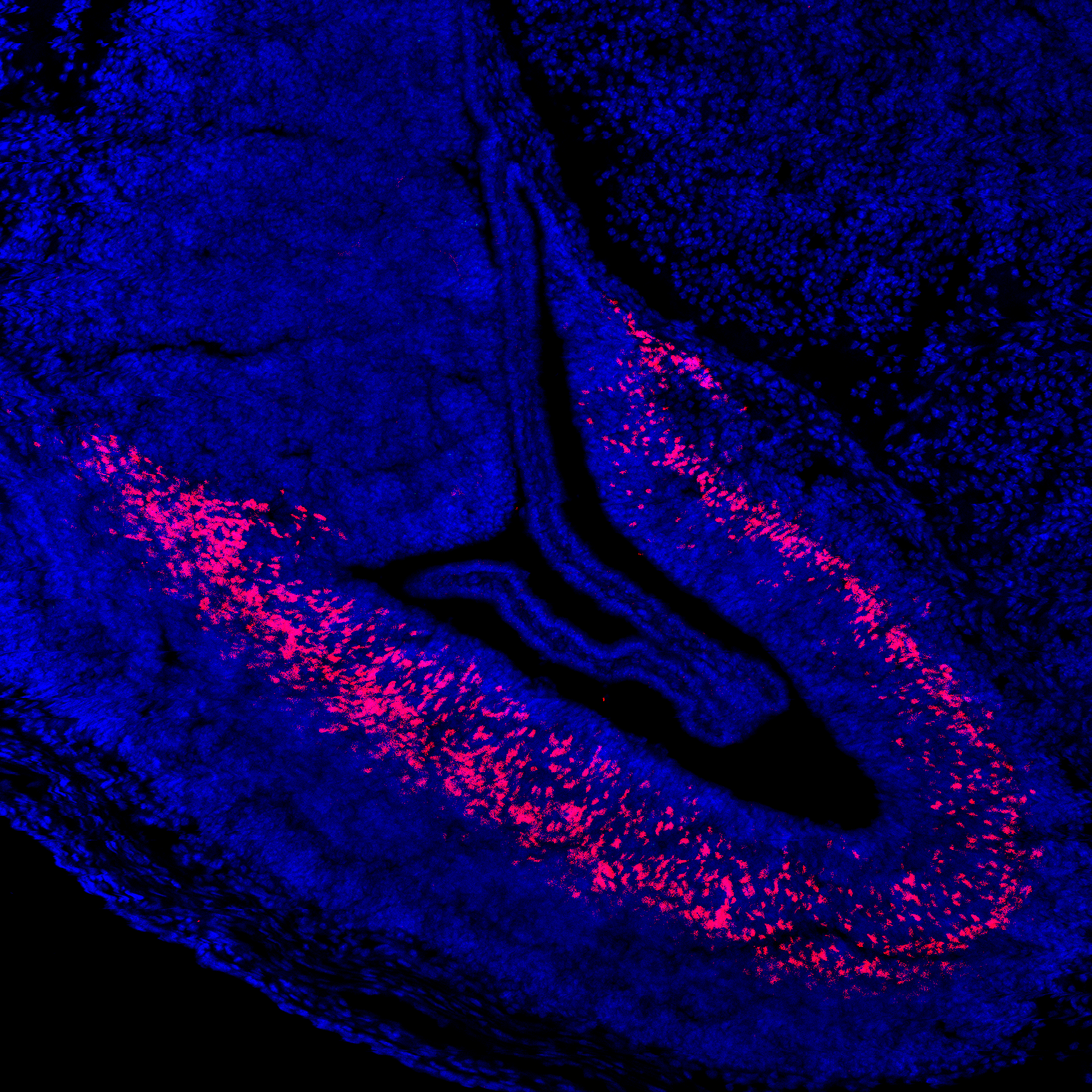

Supplement: Supplementary file 4 — Source data Fig. 2 [file 44319_2026_768_MOESM4_ESM.zip › Figure 2/2E/d.tif]

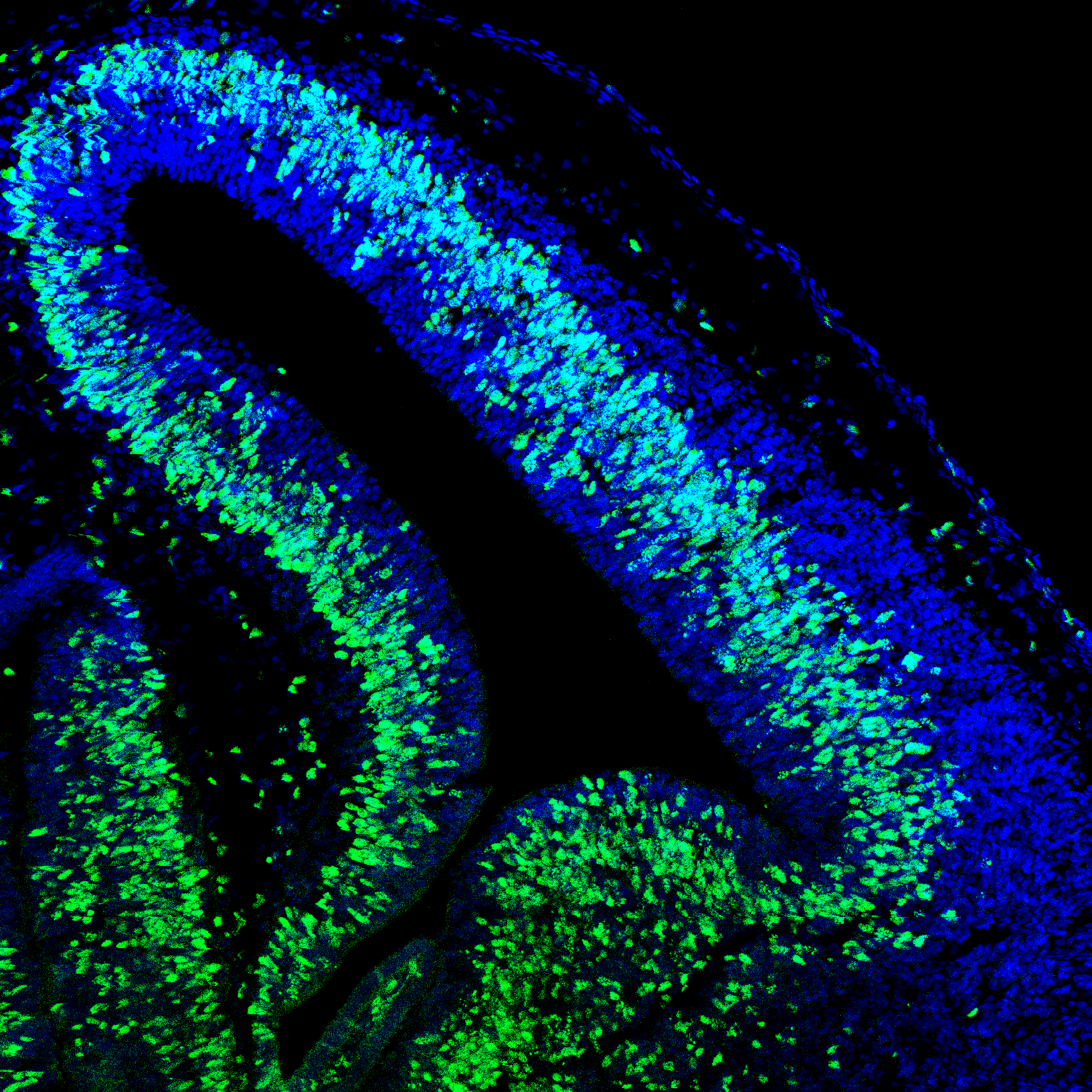

Supplement: Supplementary file 5 — Source data Fig. 3 [file 44319_2026_768_MOESM5_ESM.zip › Figure 3/3A/a.tif]

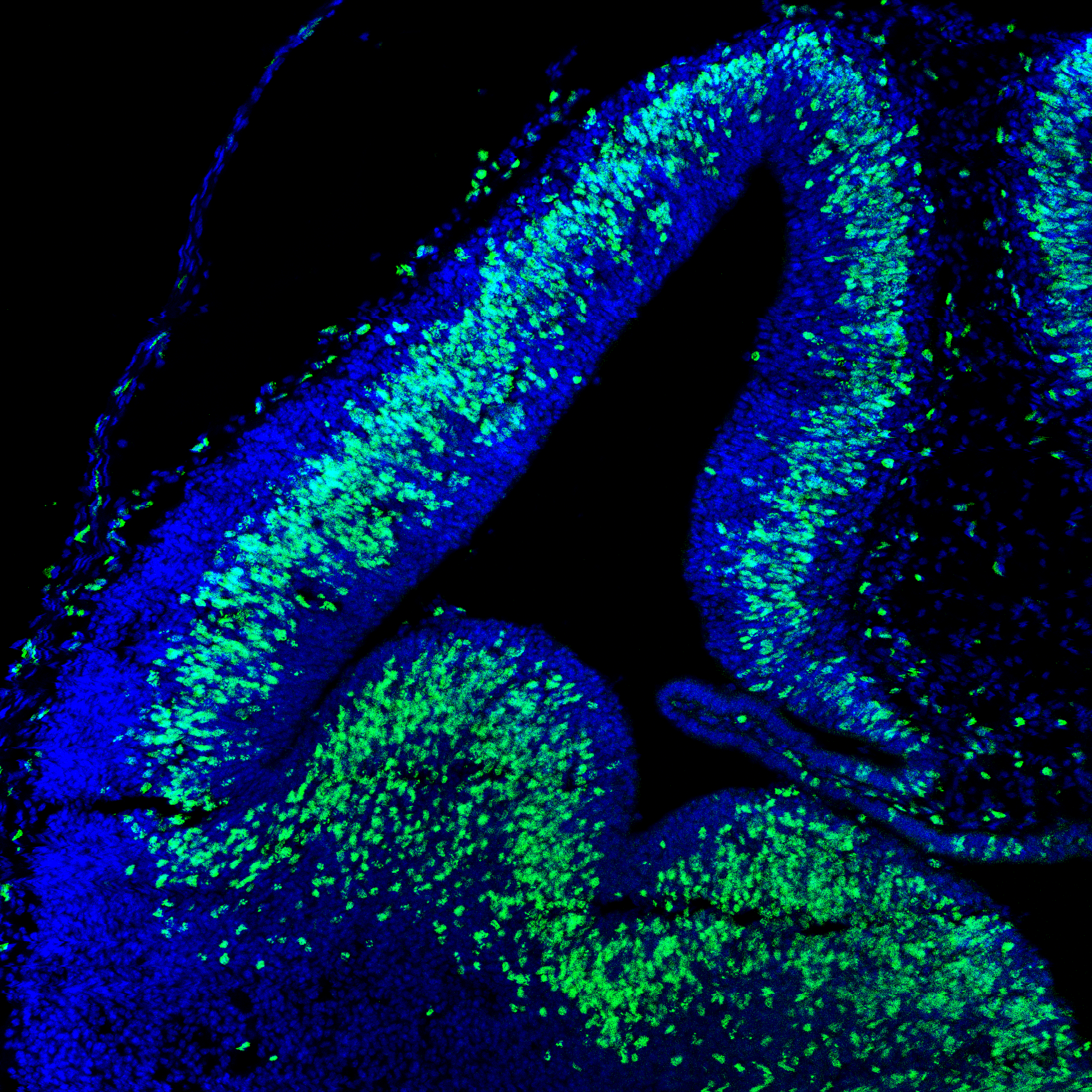

Supplement: Supplementary file 5 — Source data Fig. 3 [file 44319_2026_768_MOESM5_ESM.zip › Figure 3/3A/b.tif]

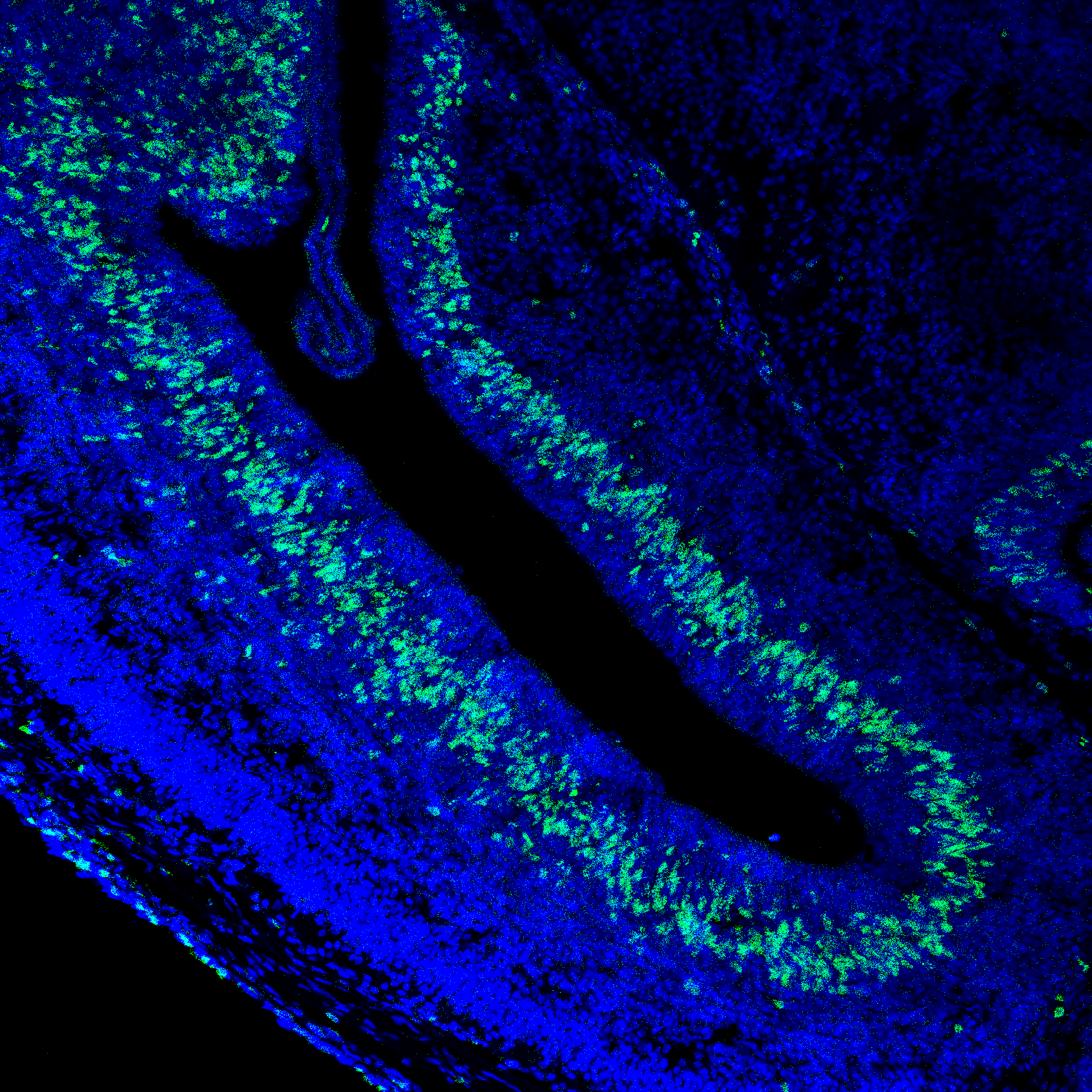

Supplement: Supplementary file 5 — Source data Fig. 3 [file 44319_2026_768_MOESM5_ESM.zip › Figure 3/3A/c.tif]

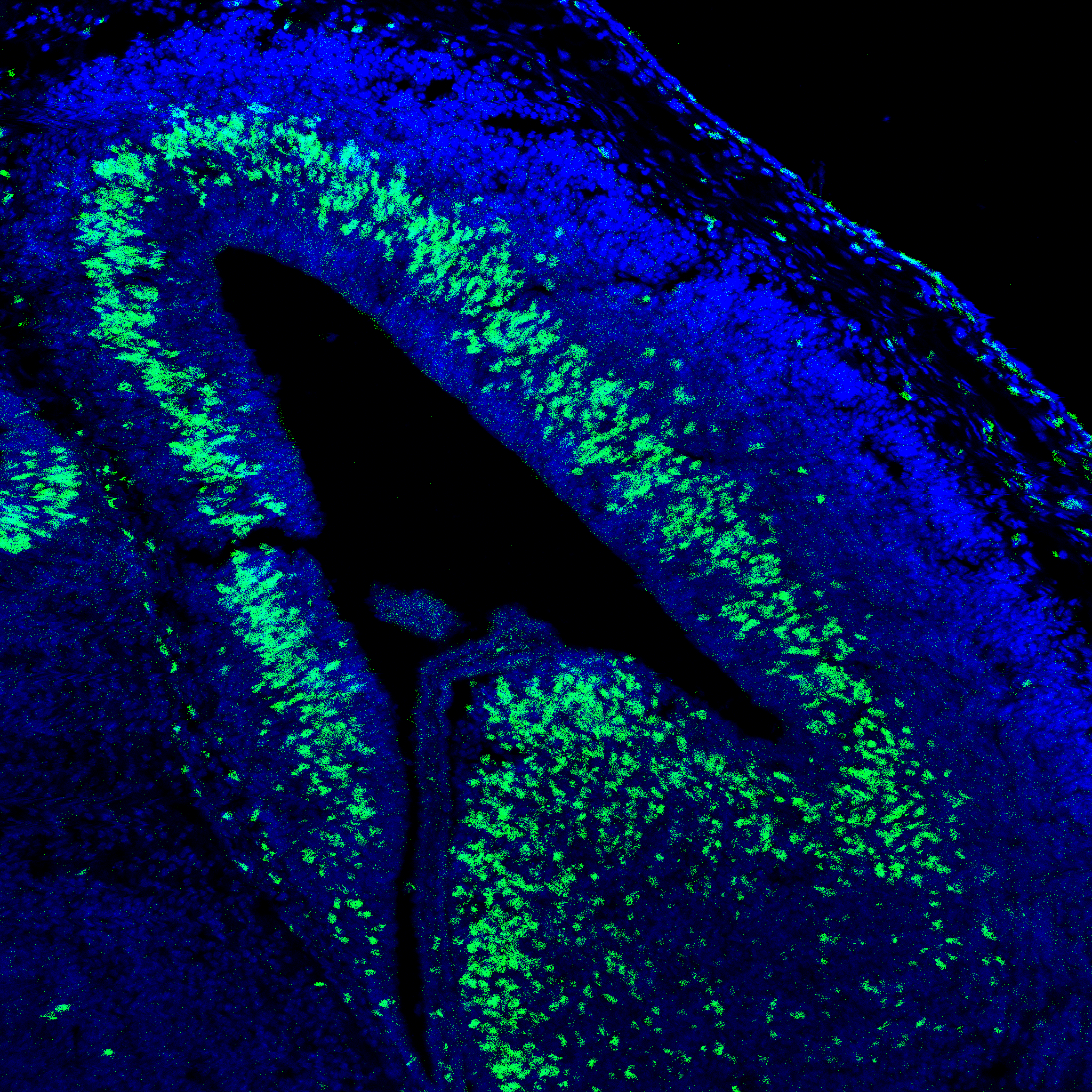

Supplement: Supplementary file 5 — Source data Fig. 3 [file 44319_2026_768_MOESM5_ESM.zip › Figure 3/3A/d.tif]

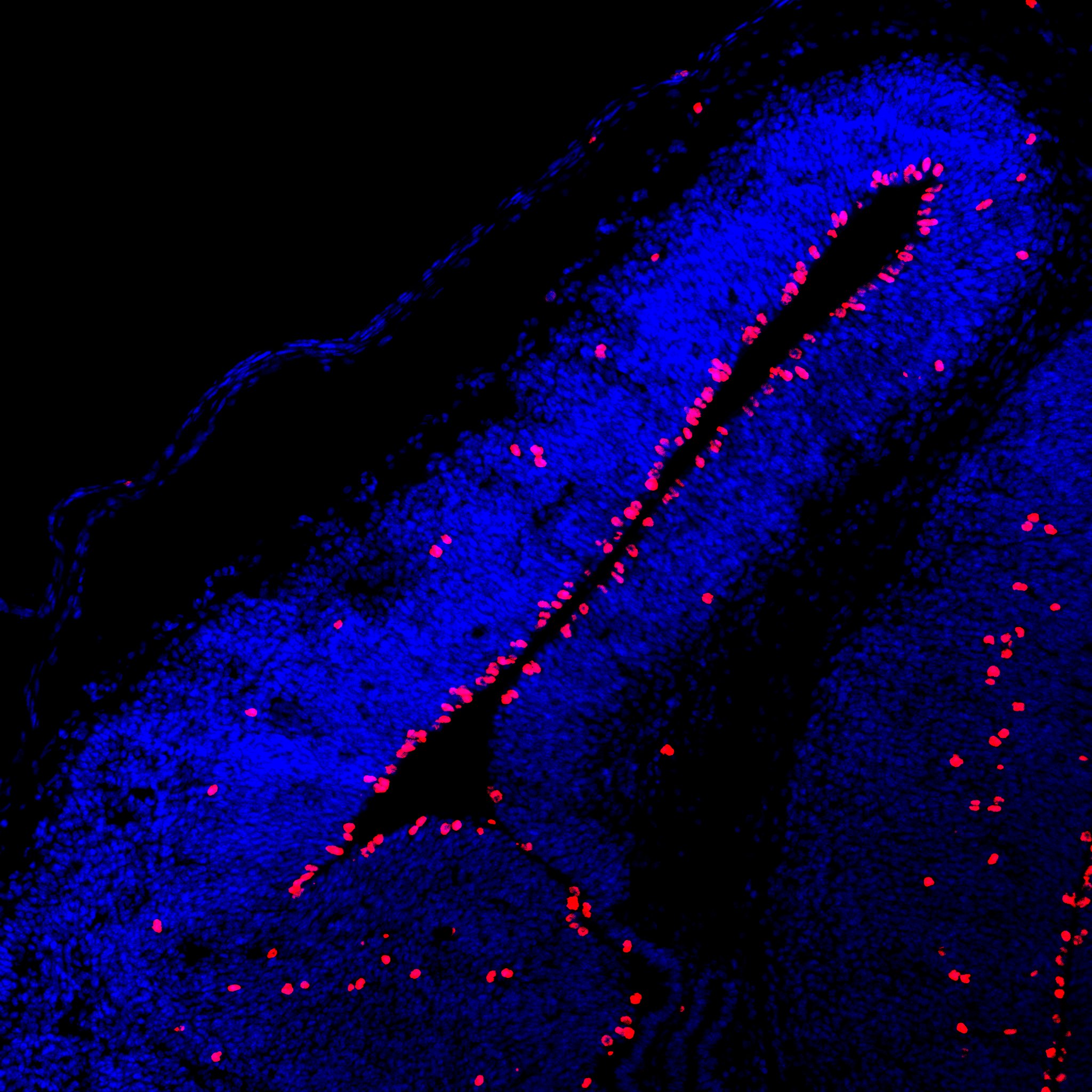

Supplement: Supplementary file 5 — Source data Fig. 3 [file 44319_2026_768_MOESM5_ESM.zip › Figure 3/3C/a.jpg]

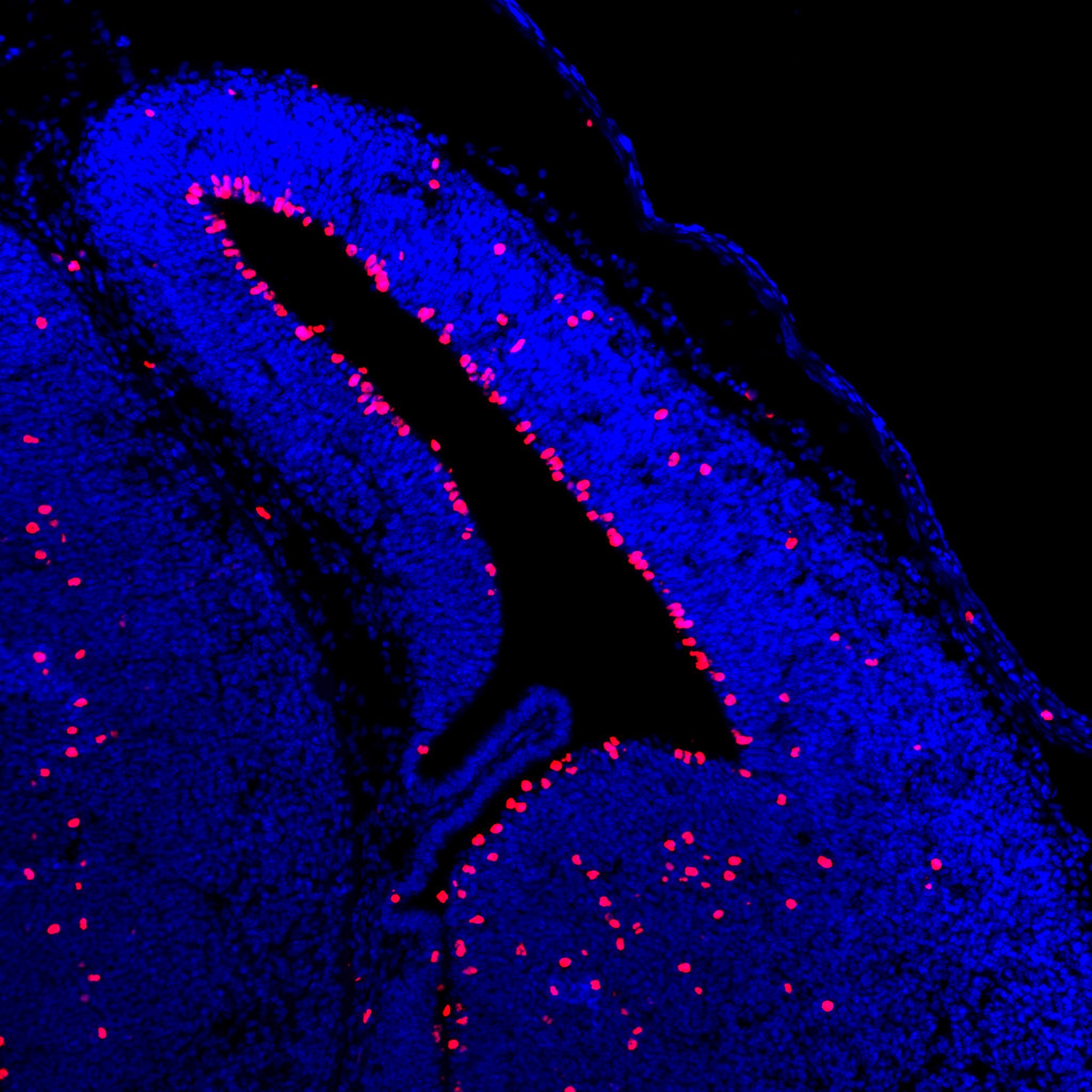

Supplement: Supplementary file 5 — Source data Fig. 3 [file 44319_2026_768_MOESM5_ESM.zip › Figure 3/3C/b.jpg]

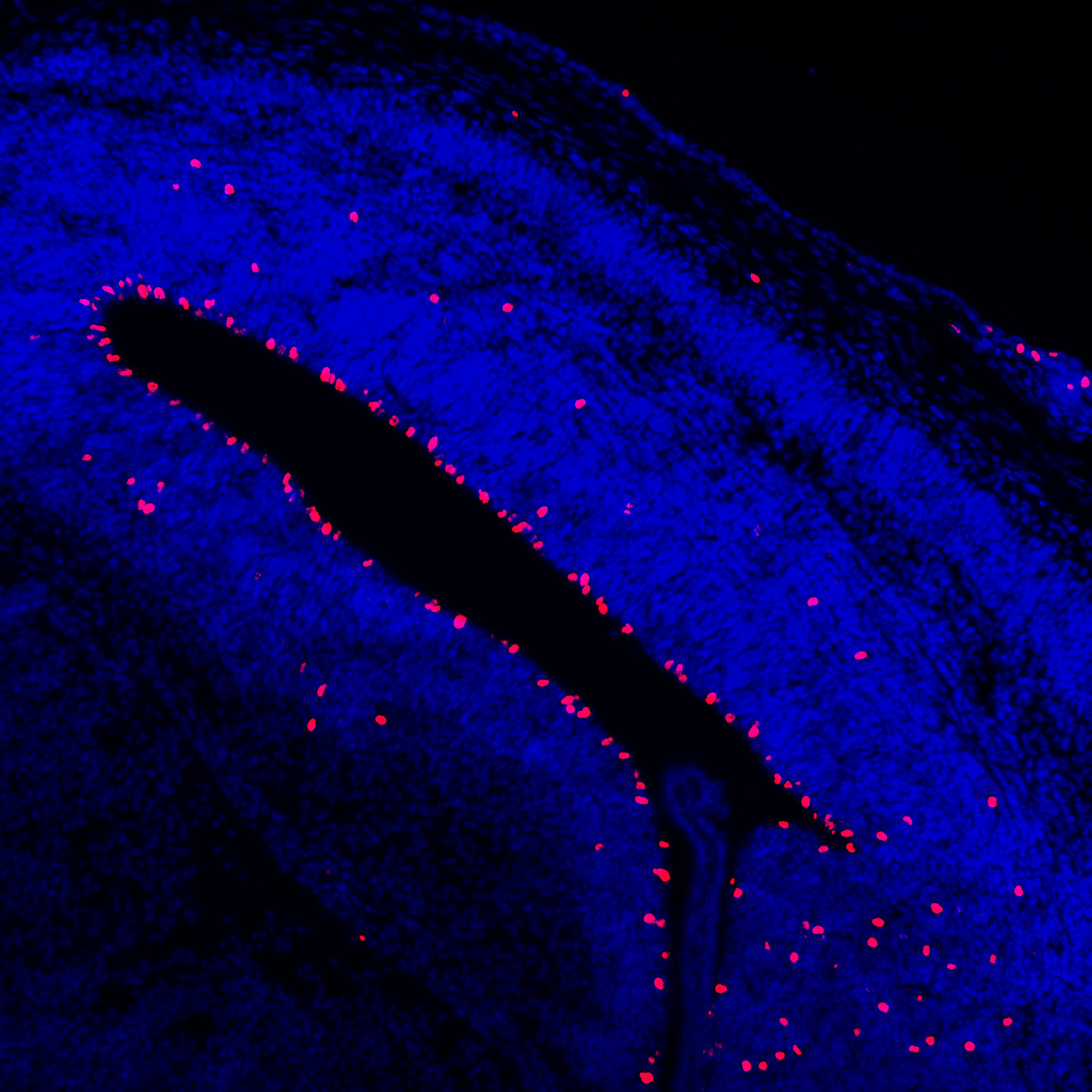

Supplement: Supplementary file 5 — Source data Fig. 3 [file 44319_2026_768_MOESM5_ESM.zip › Figure 3/3C/c.tif]

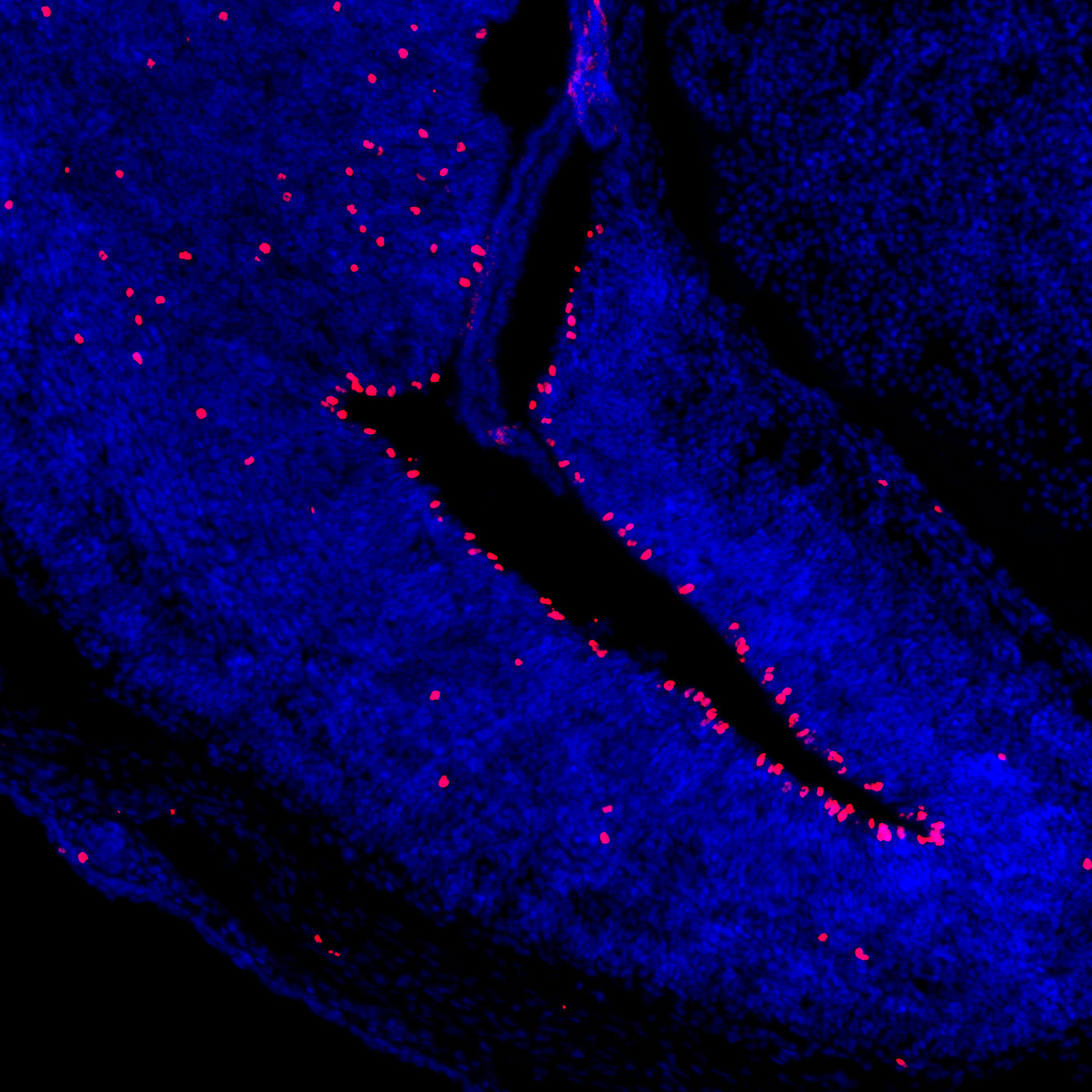

Supplement: Supplementary file 5 — Source data Fig. 3 [file 44319_2026_768_MOESM5_ESM.zip › Figure 3/3C/d.tif]

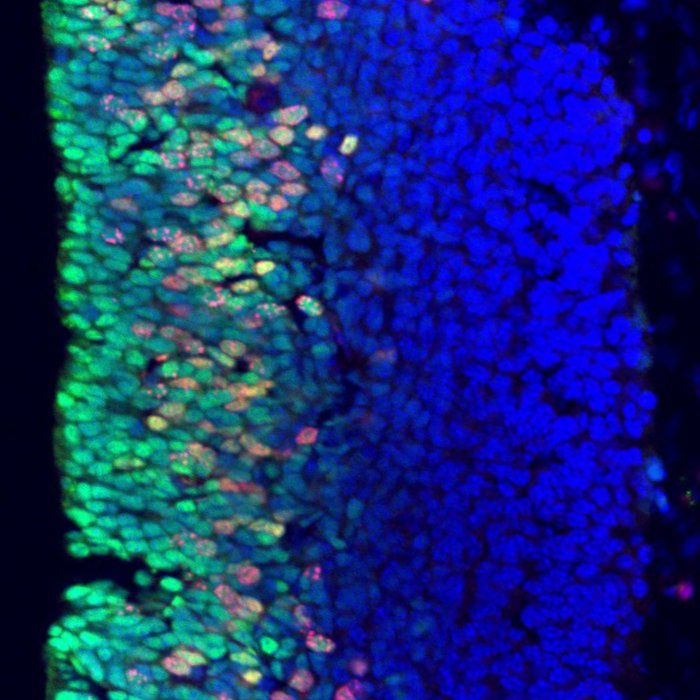

Supplement: Supplementary file 5 — Source data Fig. 3 [file 44319_2026_768_MOESM5_ESM.zip › Figure 3/3E/ctko.jpg]

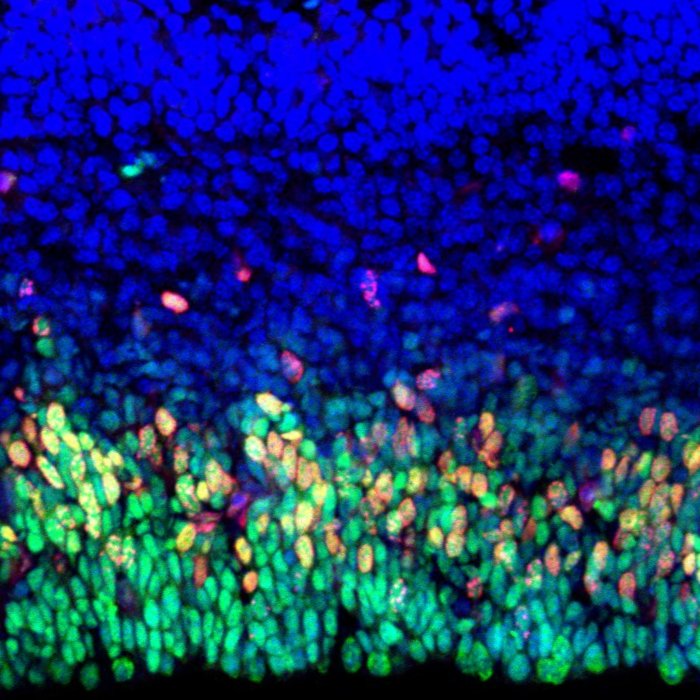

Supplement: Supplementary file 5 — Source data Fig. 3 [file 44319_2026_768_MOESM5_ESM.zip › Figure 3/3E/ctrl.jpg]

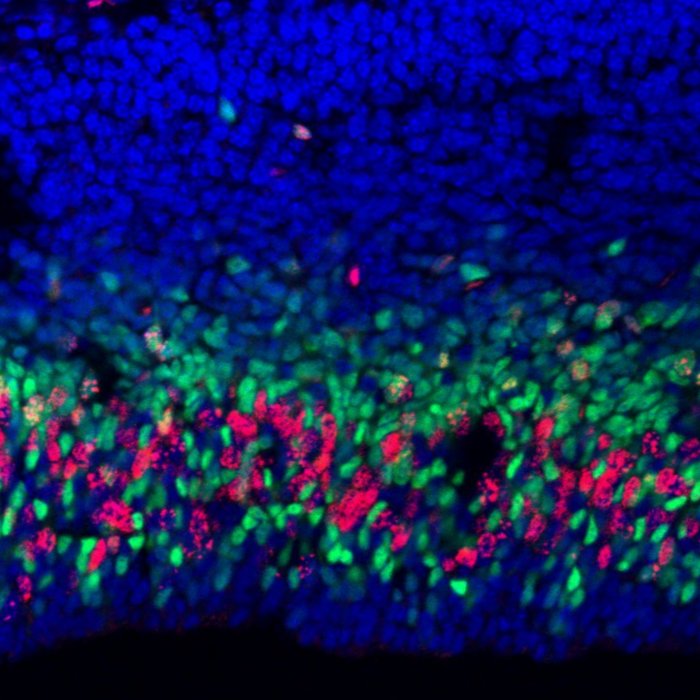

Supplement: Supplementary file 5 — Source data Fig. 3 [file 44319_2026_768_MOESM5_ESM.zip › Figure 3/3G/ctko.jpg]

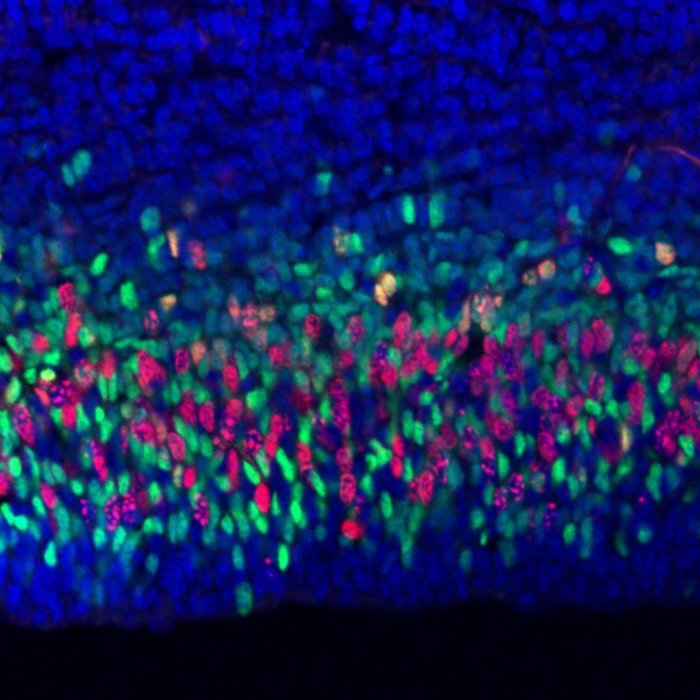

Supplement: Supplementary file 5 — Source data Fig. 3 [file 44319_2026_768_MOESM5_ESM.zip › Figure 3/3G/ctrl.jpg]

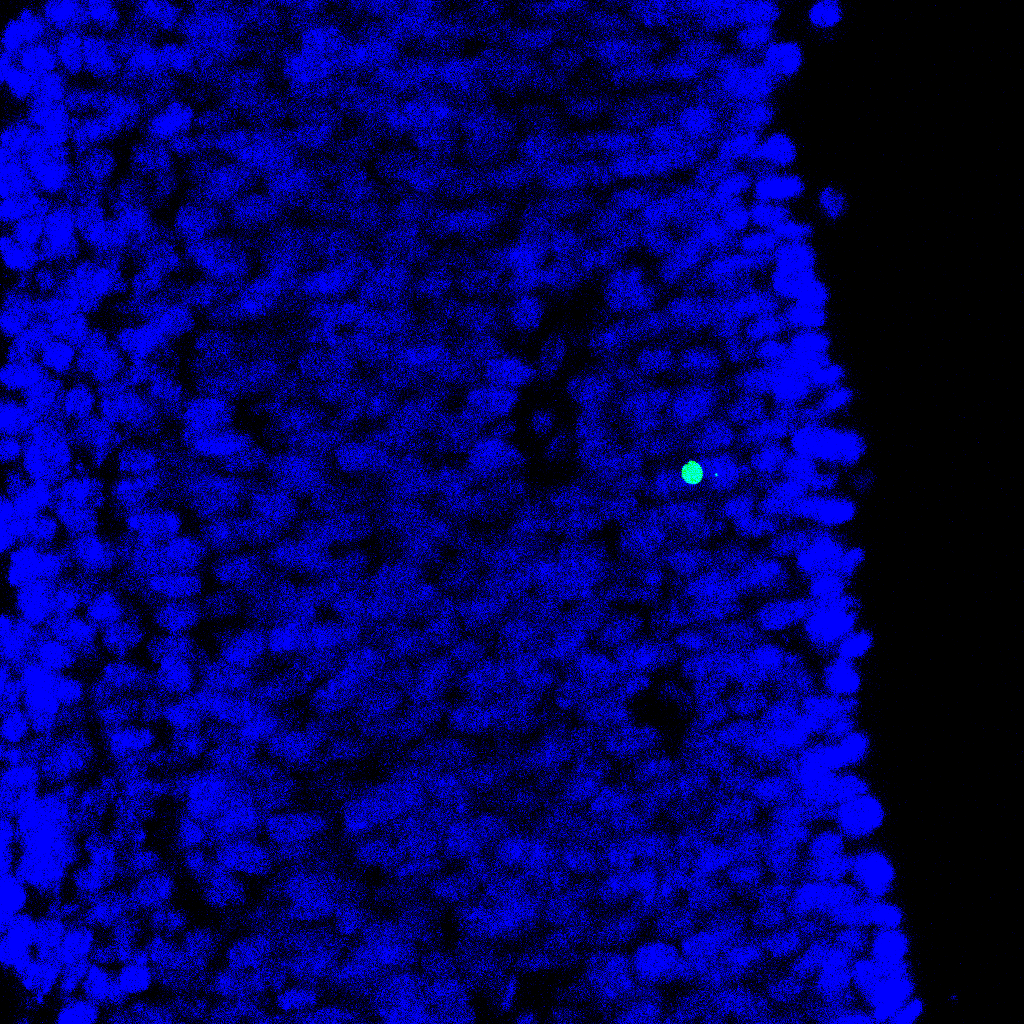

Supplement: Supplementary file 5 — Source data Fig. 3 [file 44319_2026_768_MOESM5_ESM.zip › Figure 3/3I/e13.5-ctko.tif]

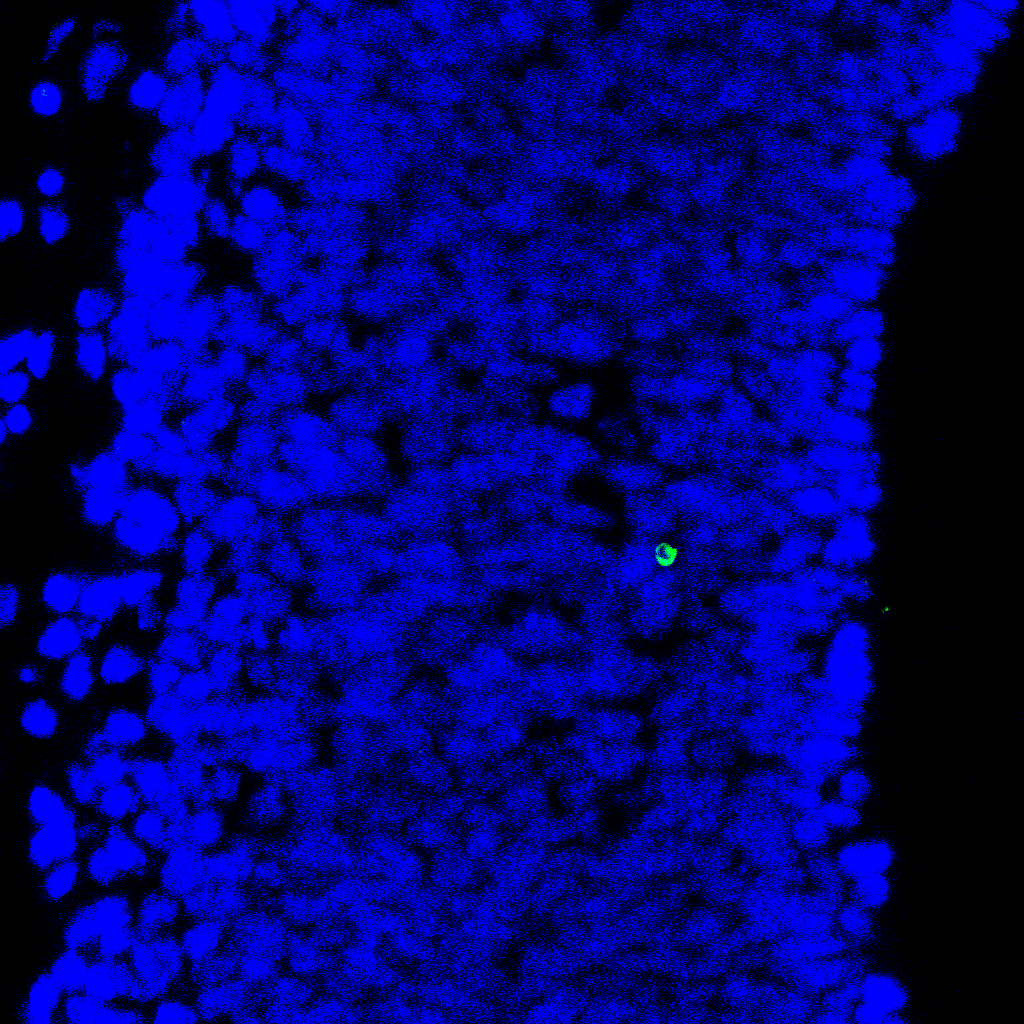

Supplement: Supplementary file 5 — Source data Fig. 3 [file 44319_2026_768_MOESM5_ESM.zip › Figure 3/3I/e13.5-ctrl.tif]

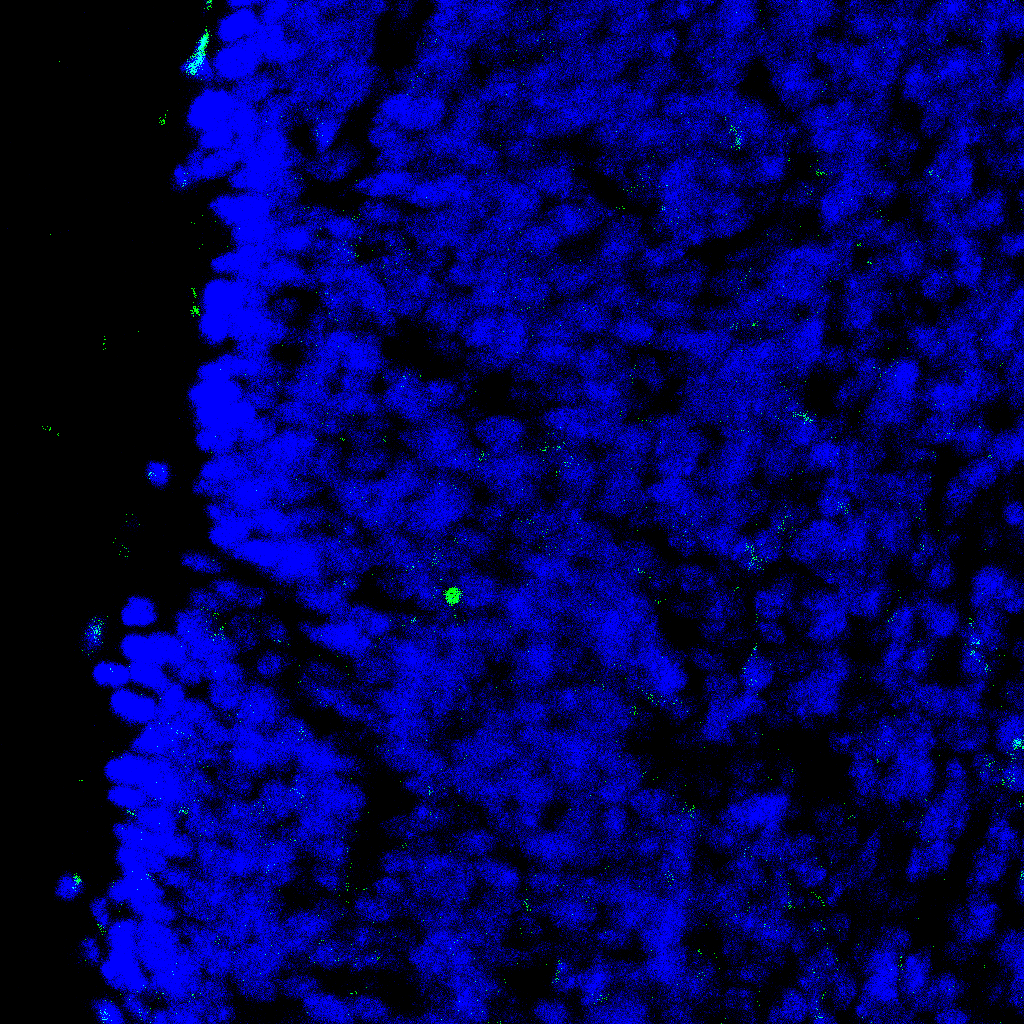

Supplement: Supplementary file 5 — Source data Fig. 3 [file 44319_2026_768_MOESM5_ESM.zip › Figure 3/3I/e15.5-ctko.tif]

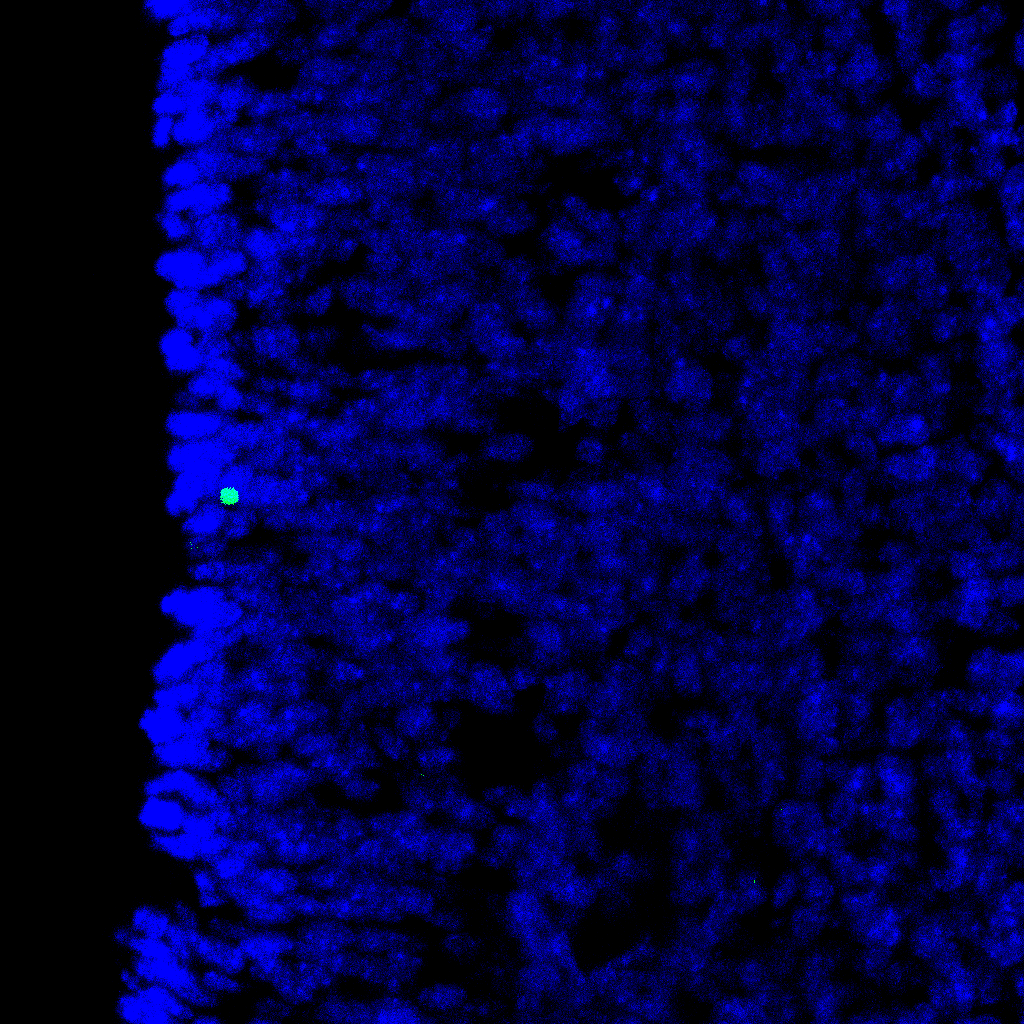

Supplement: Supplementary file 5 — Source data Fig. 3 [file 44319_2026_768_MOESM5_ESM.zip › Figure 3/3I/e15.5-ctrl.tif]

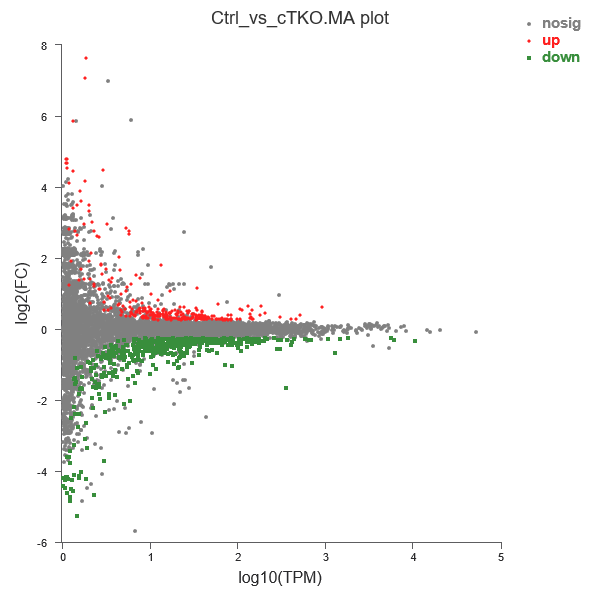

Supplement: Supplementary file 6 — Source data Fig. 4 [file 44319_2026_768_MOESM6_ESM.zip › Figure 4/4B/4b.png]

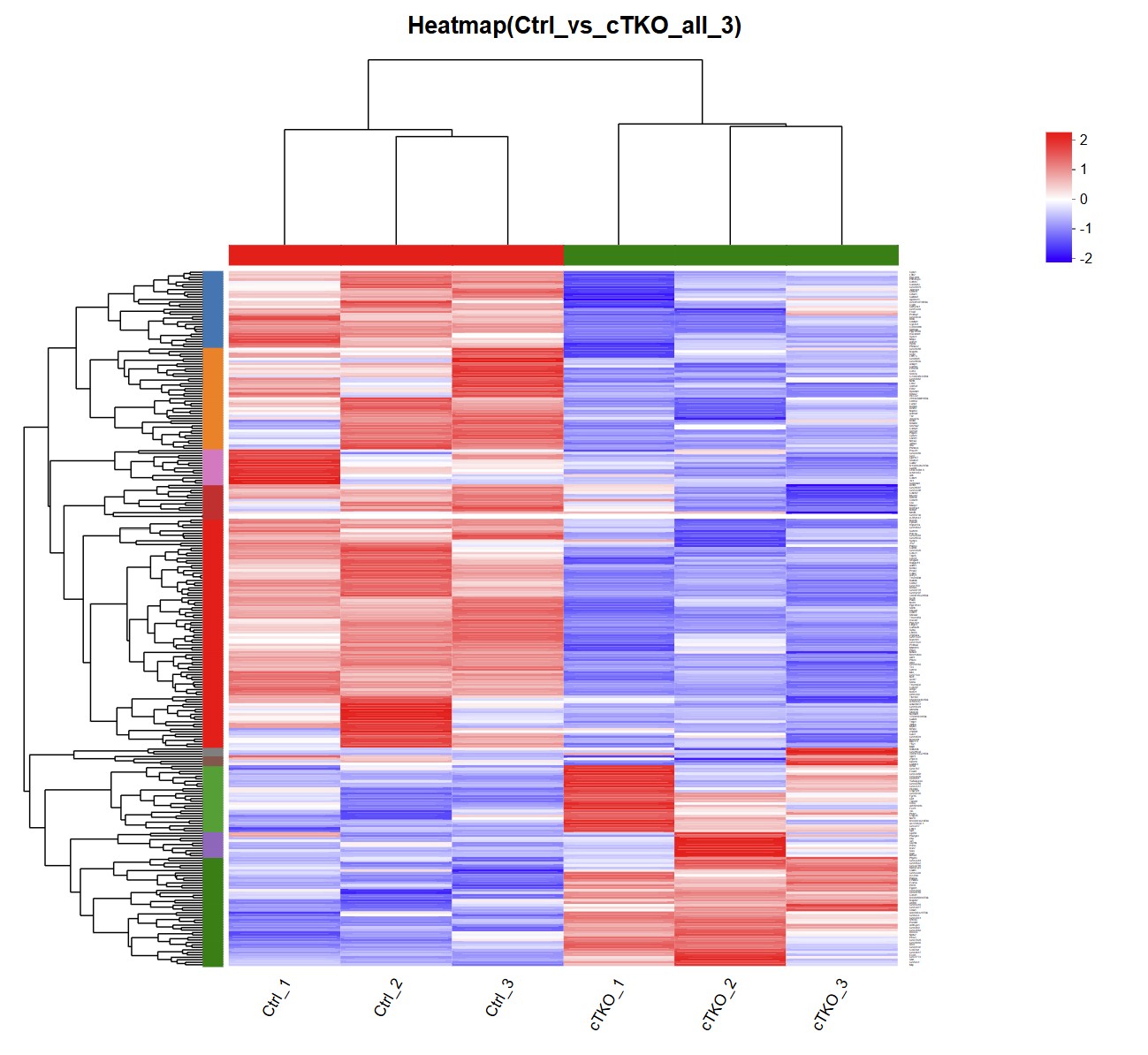

Supplement: Supplementary file 6 — Source data Fig. 4 [file 44319_2026_768_MOESM6_ESM.zip › Figure 4/4C/heatmap all deg.jpg]

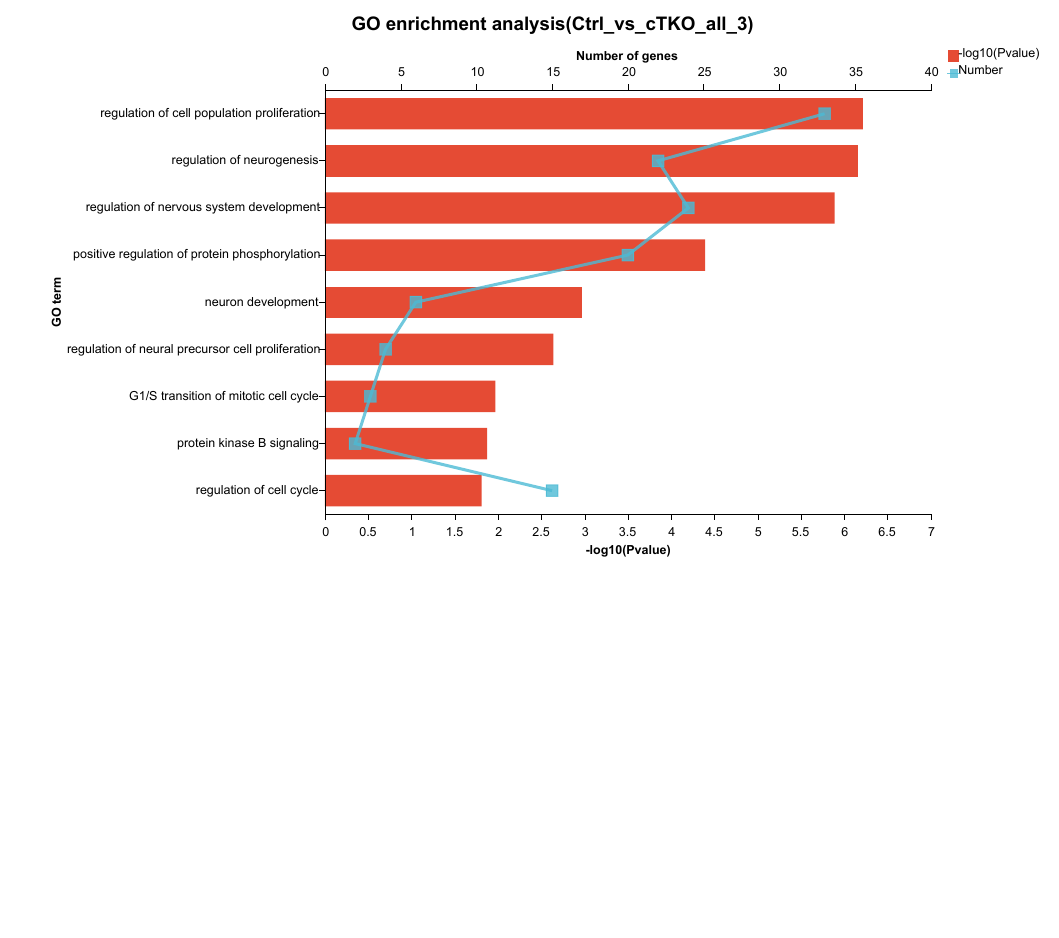

Supplement: Supplementary file 6 — Source data Fig. 4 [file 44319_2026_768_MOESM6_ESM.zip › Figure 4/4D/4D.png]

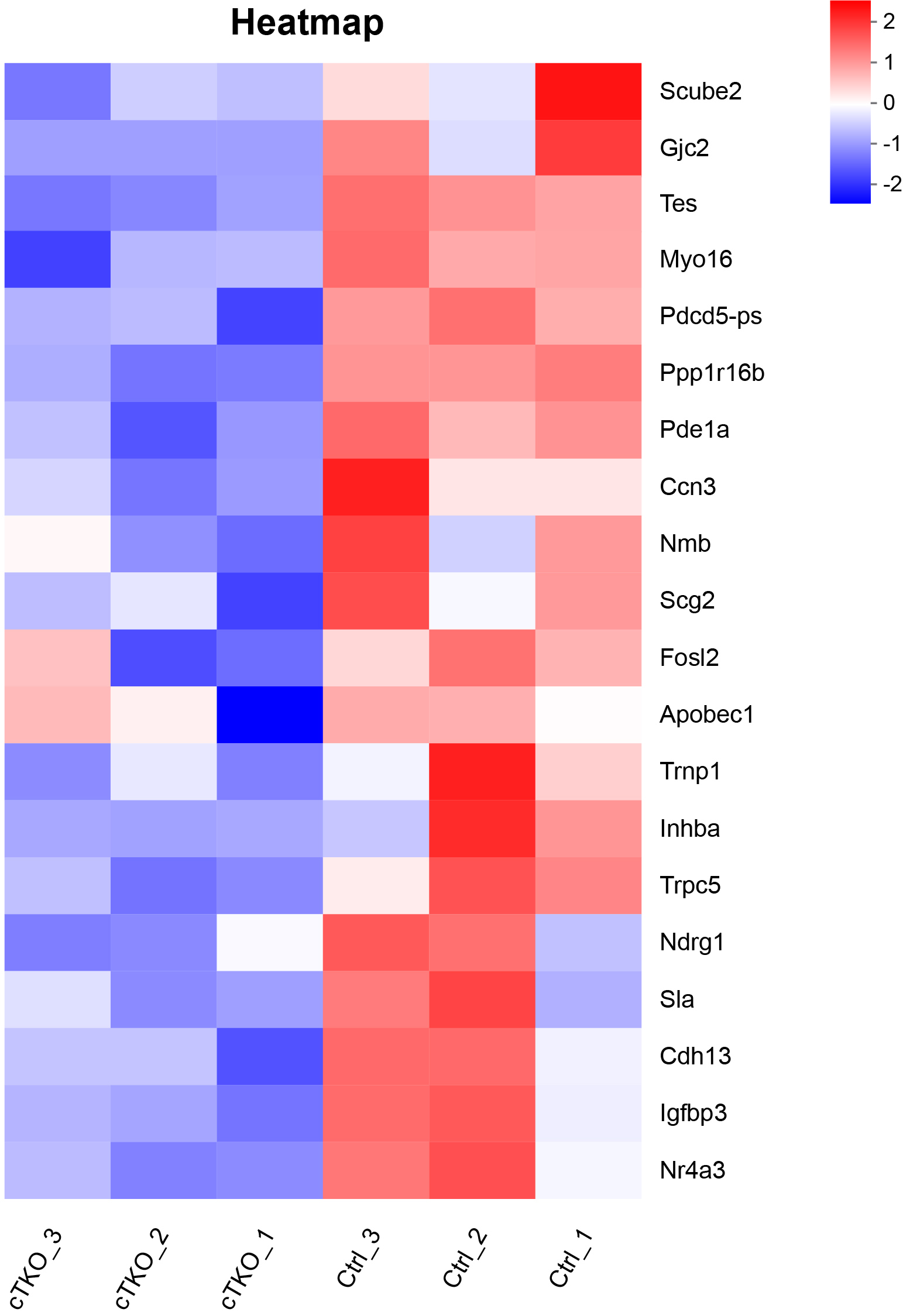

Supplement: Supplementary file 6 — Source data Fig. 4 [file 44319_2026_768_MOESM6_ESM.zip › Figure 4/4E/4e.jpg]

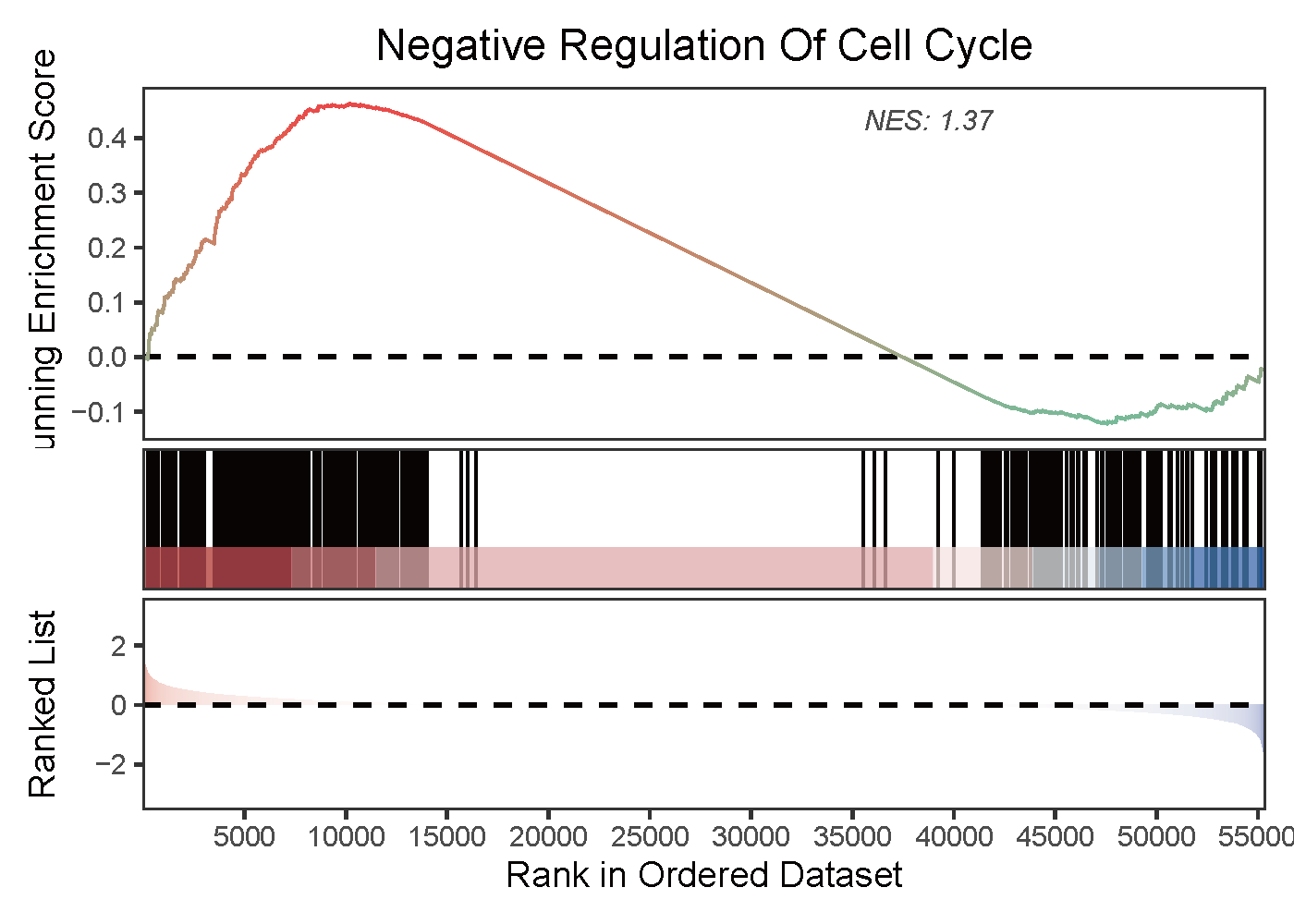

Supplement: Supplementary file 6 — Source data Fig. 4 [file 44319_2026_768_MOESM6_ESM.zip › Figure 4/4F/GOBP_NEGATIVE_REGULATION_OF_CELL_CYCLE.tif]

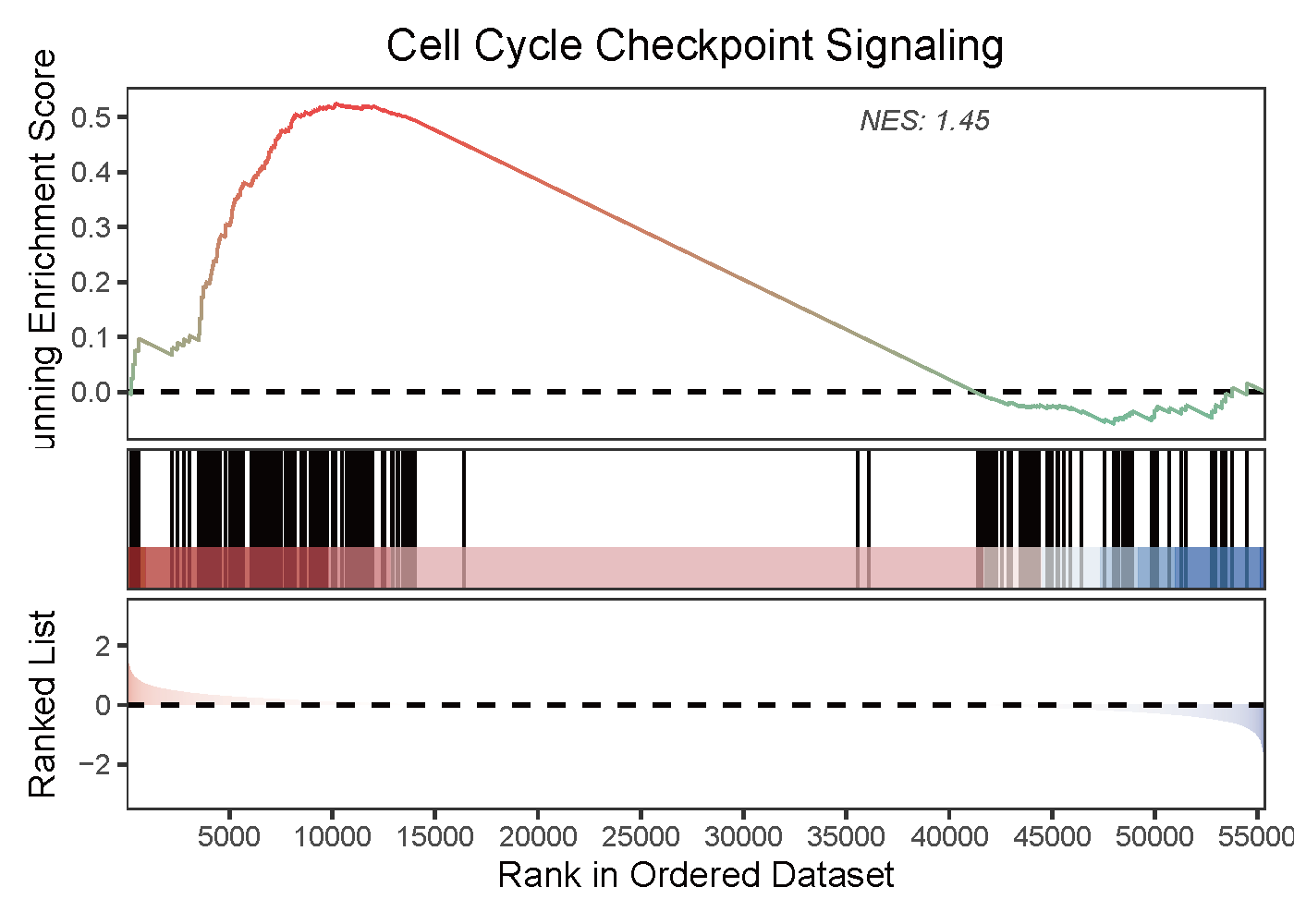

Supplement: Supplementary file 6 — Source data Fig. 4 [file 44319_2026_768_MOESM6_ESM.zip › Figure 4/4G/GOBP_CELL_CYCLE_CHECKPOINT_SIGNALING.tif]

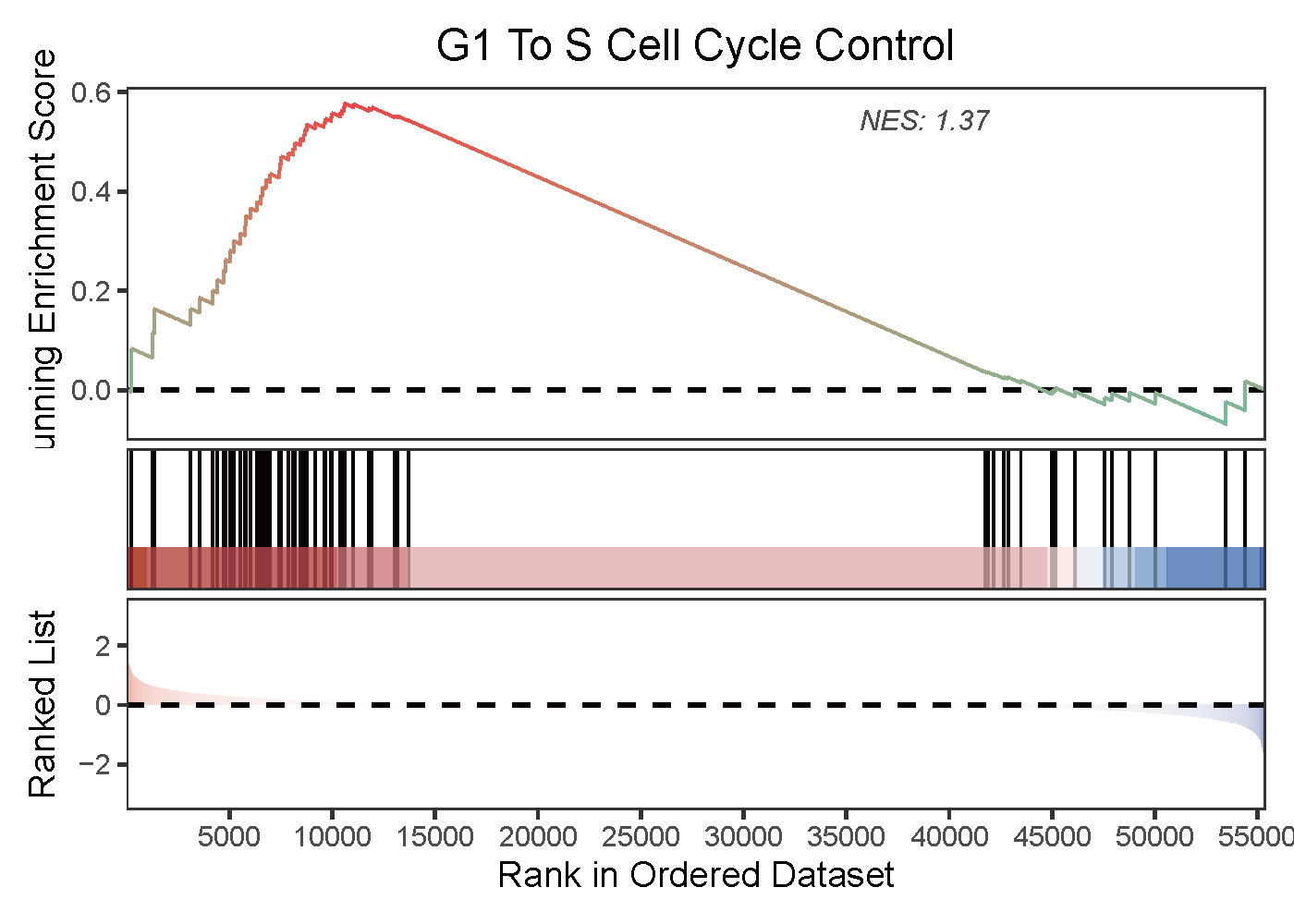

Supplement: Supplementary file 6 — Source data Fig. 4 [file 44319_2026_768_MOESM6_ESM.zip › Figure 4/4H/WP_G1_TO_S_CELL_CYCLE_CONTROL.tiff]

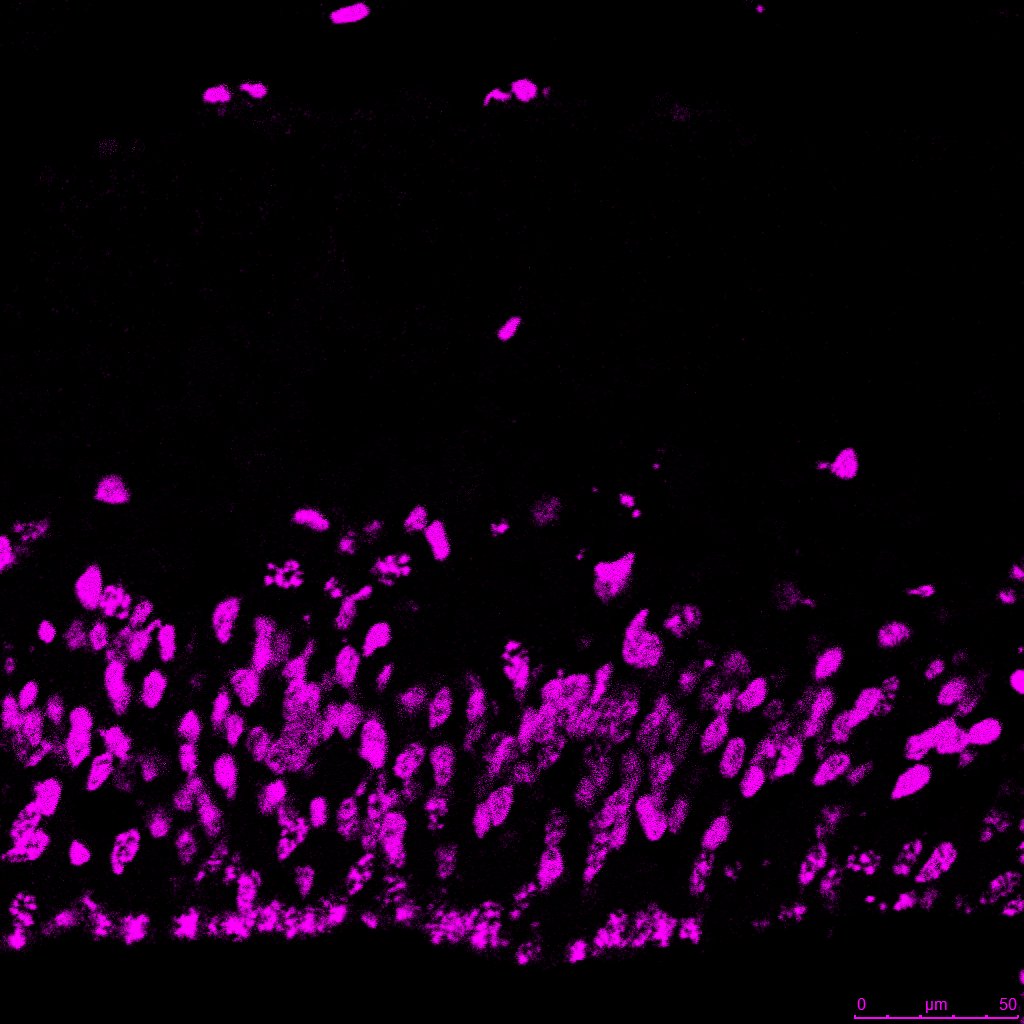

Supplement: Supplementary file 7 — Source data Fig. 5 [file 44319_2026_768_MOESM7_ESM.zip › Figure 5/5A/ctko-brdu.jpg]

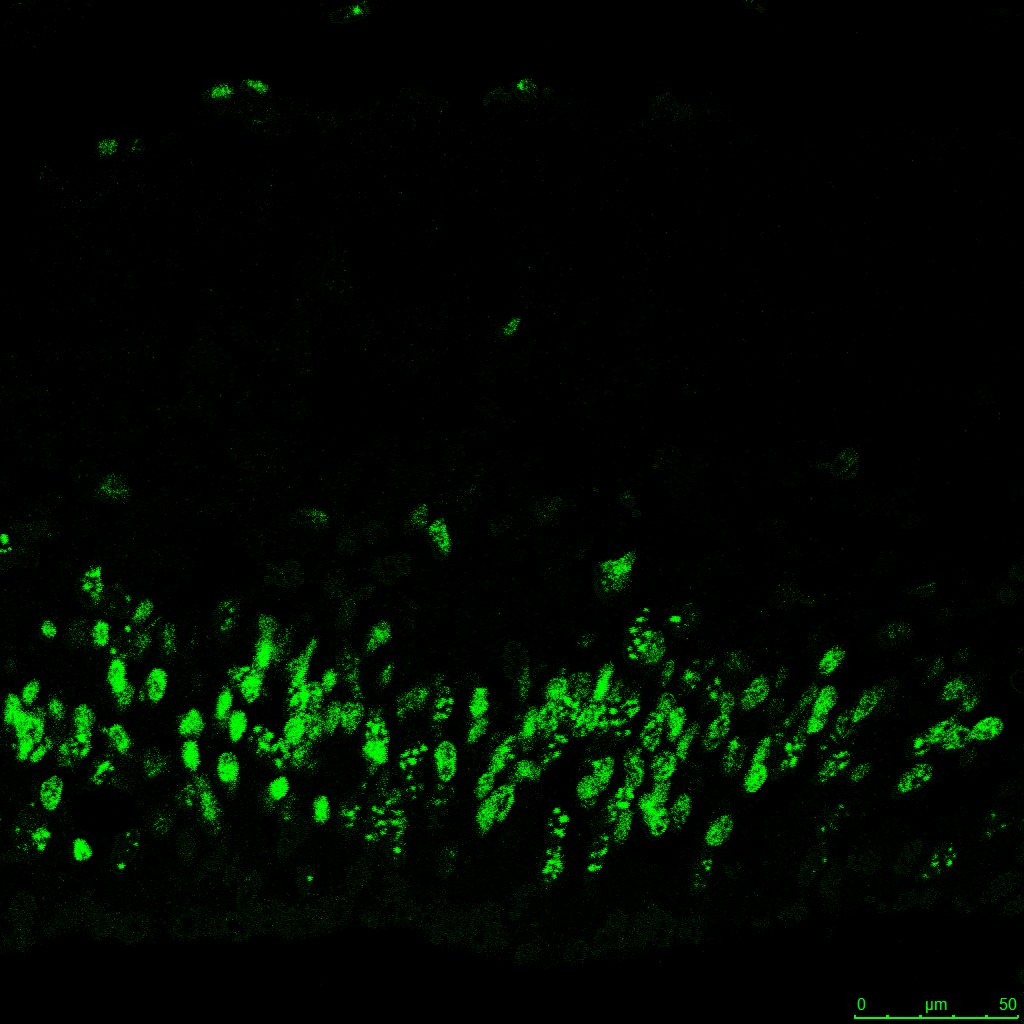

Supplement: Supplementary file 7 — Source data Fig. 5 [file 44319_2026_768_MOESM7_ESM.zip › Figure 5/5A/ctko-edu.jpg]

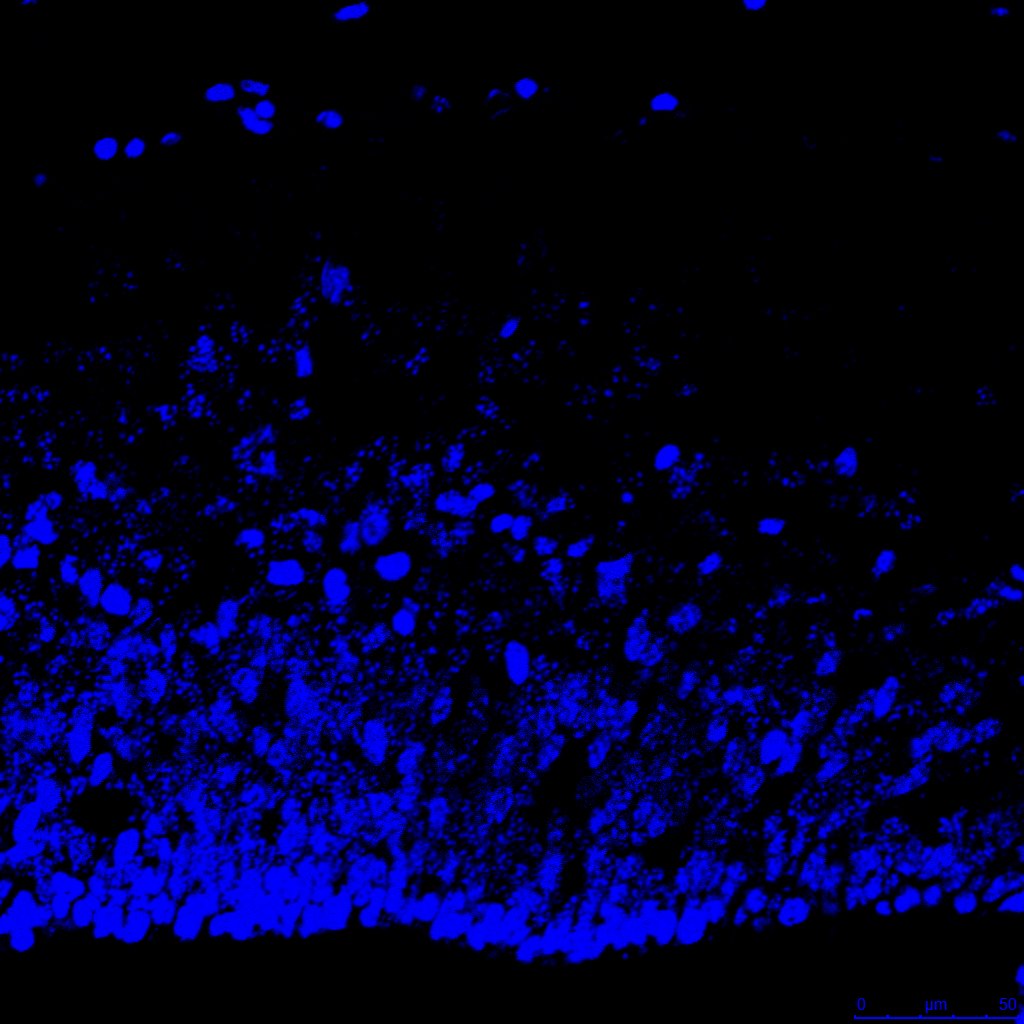

Supplement: Supplementary file 7 — Source data Fig. 5 [file 44319_2026_768_MOESM7_ESM.zip › Figure 5/5A/ctko-ki67.jpg]

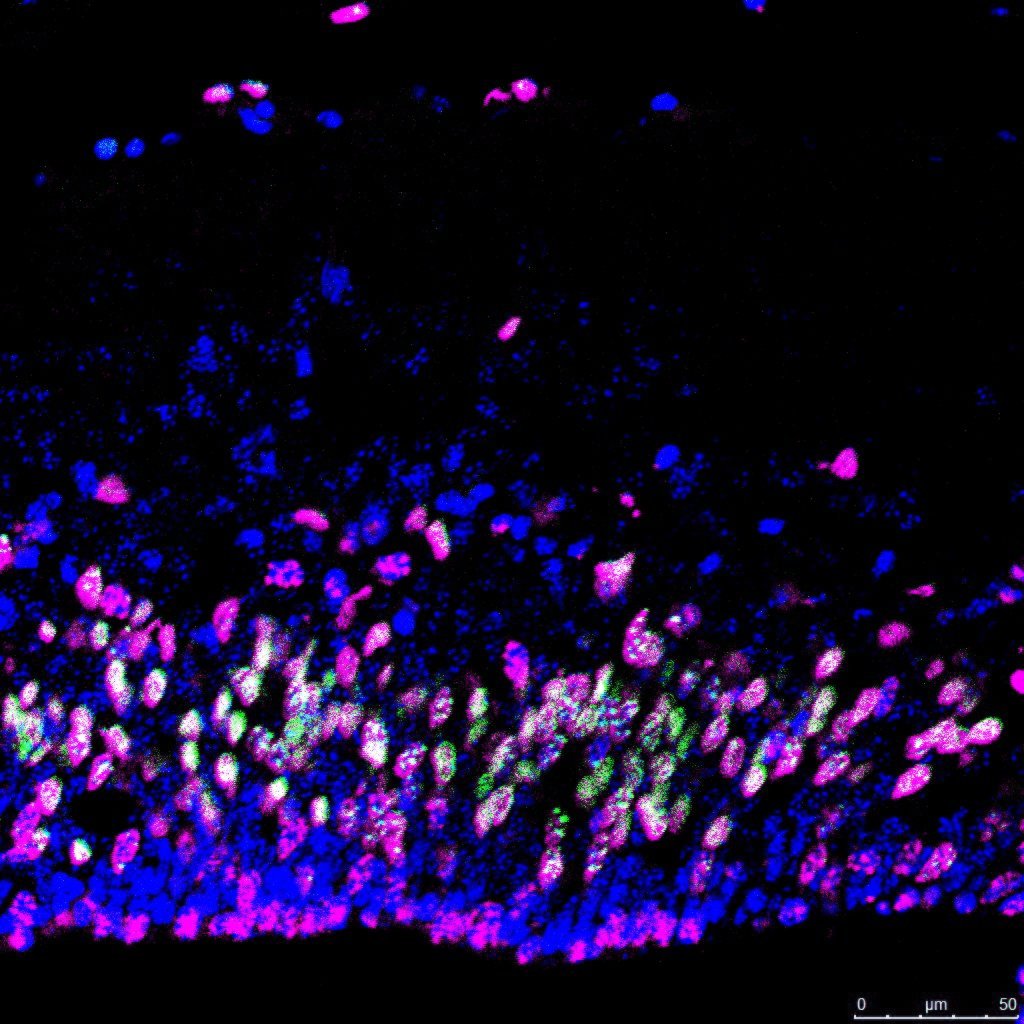

Supplement: Supplementary file 7 — Source data Fig. 5 [file 44319_2026_768_MOESM7_ESM.zip › Figure 5/5A/ctko-merge.jpg]

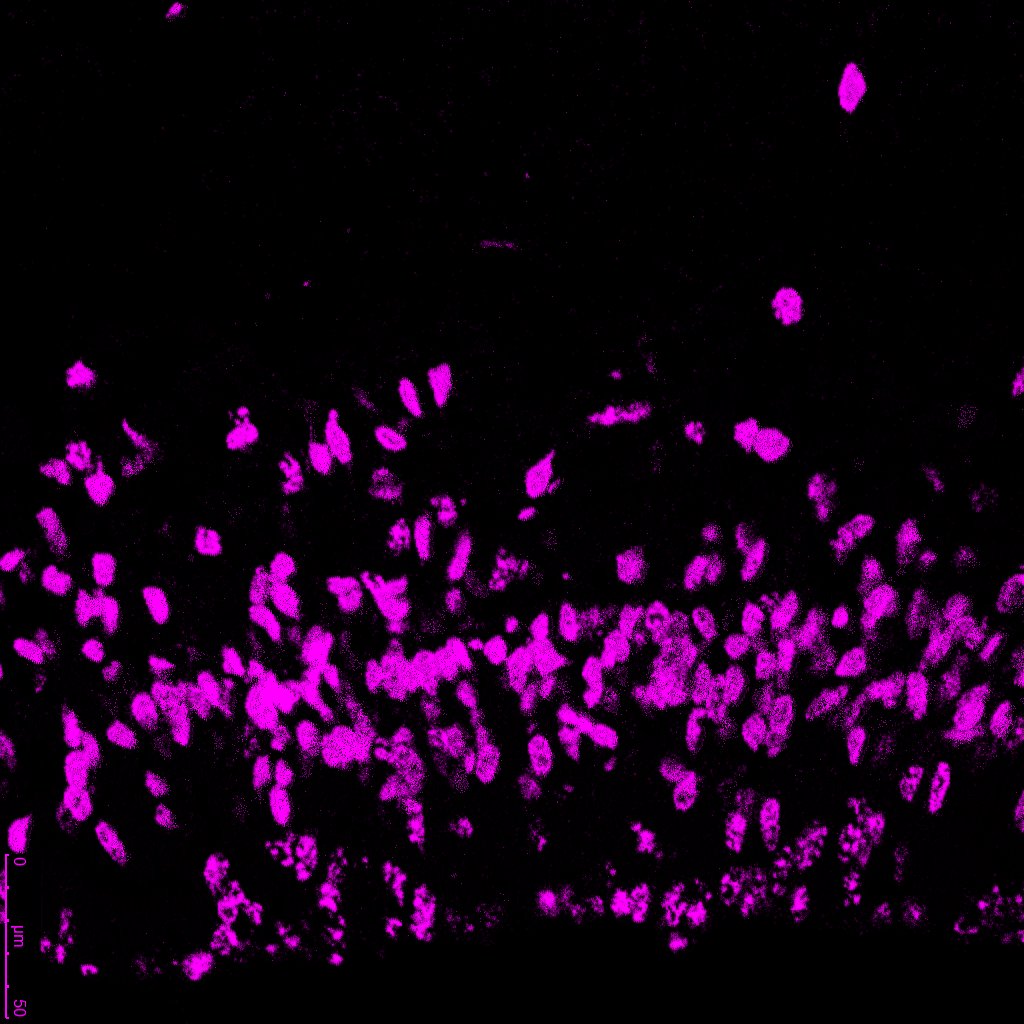

Supplement: Supplementary file 7 — Source data Fig. 5 [file 44319_2026_768_MOESM7_ESM.zip › Figure 5/5A/ctrl-brdu.jpg]

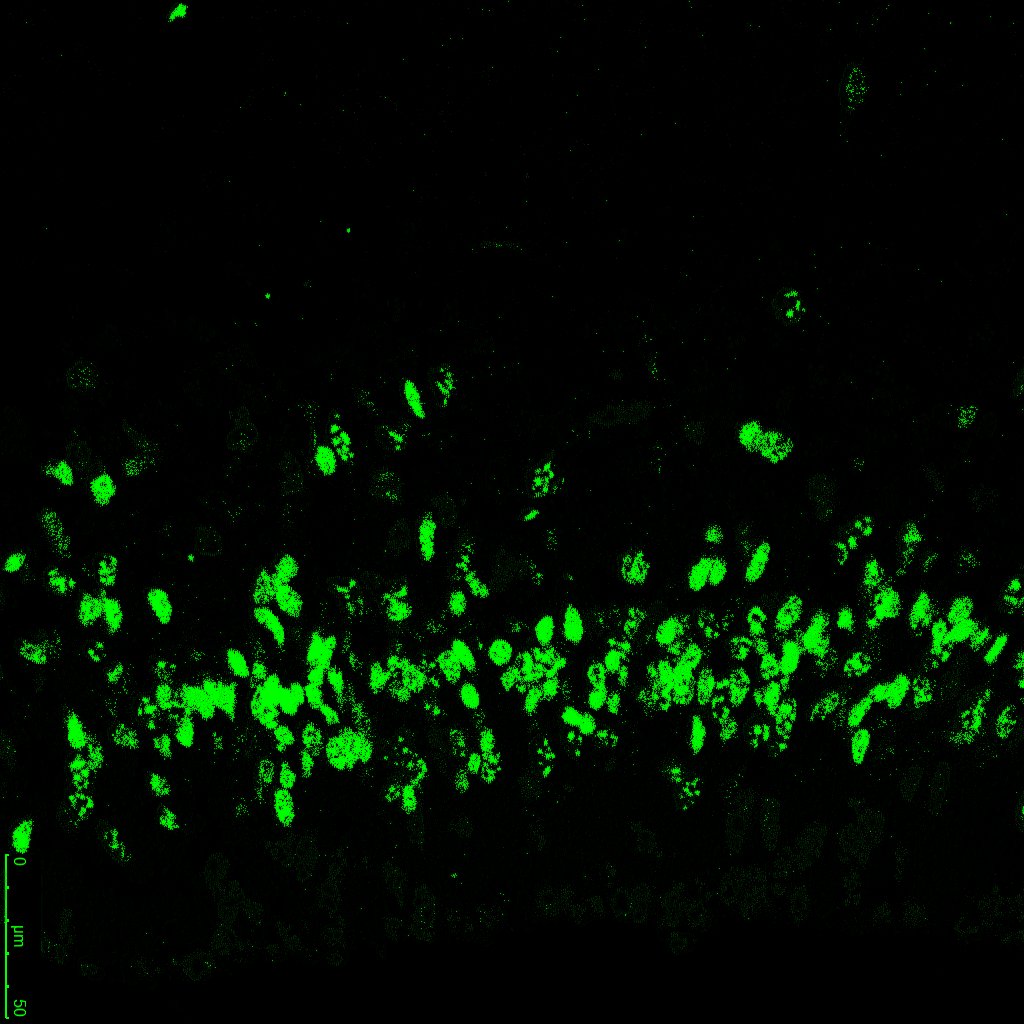

Supplement: Supplementary file 7 — Source data Fig. 5 [file 44319_2026_768_MOESM7_ESM.zip › Figure 5/5A/ctrl-edu.jpg]

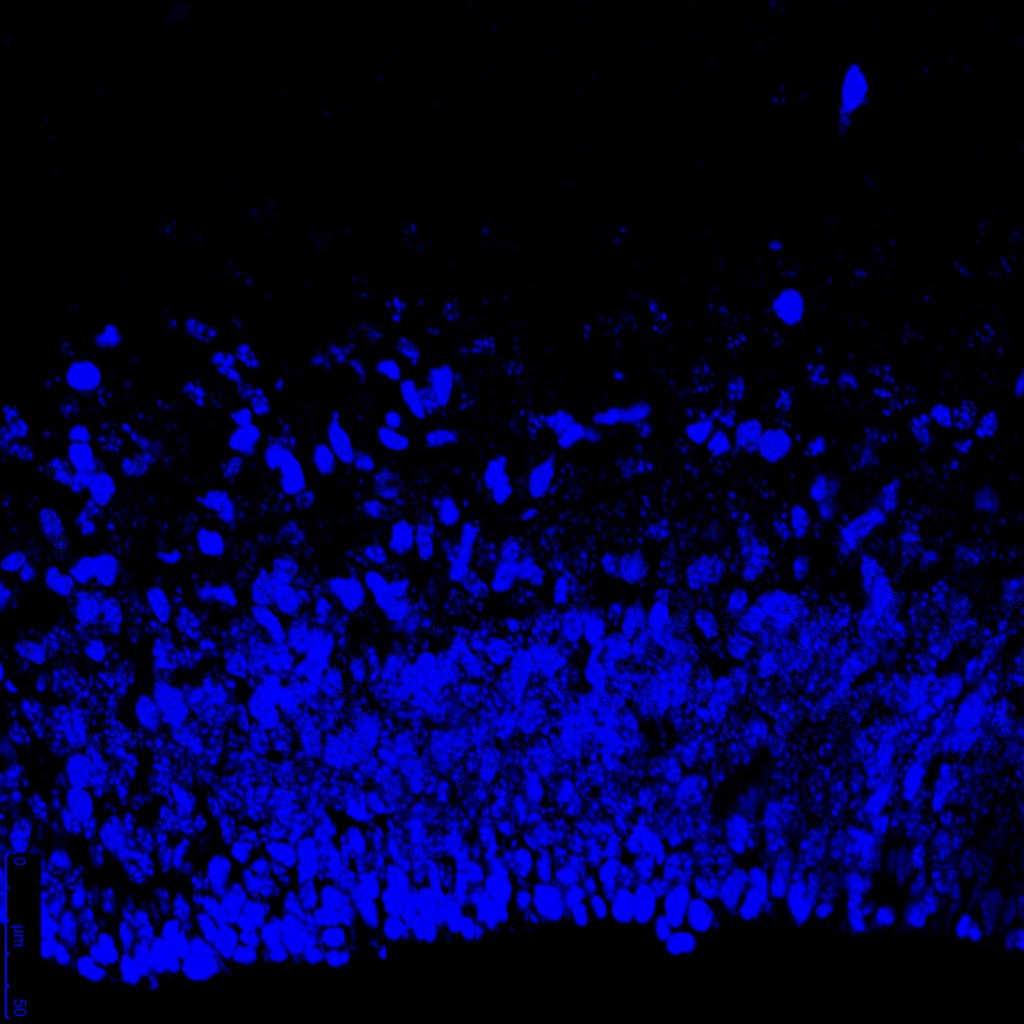

Supplement: Supplementary file 7 — Source data Fig. 5 [file 44319_2026_768_MOESM7_ESM.zip › Figure 5/5A/ctrl-ki67.jpg]

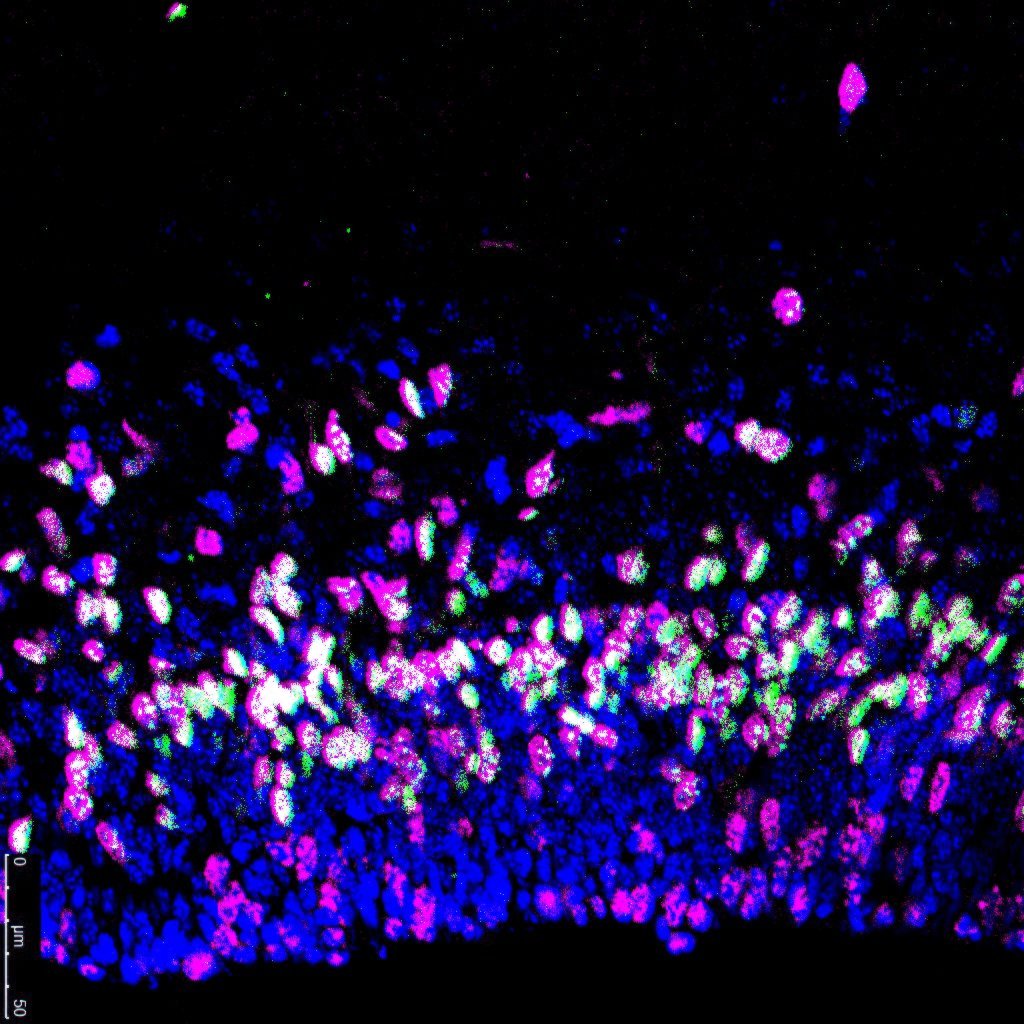

Supplement: Supplementary file 7 — Source data Fig. 5 [file 44319_2026_768_MOESM7_ESM.zip › Figure 5/5A/ctrl-merge.jpg]

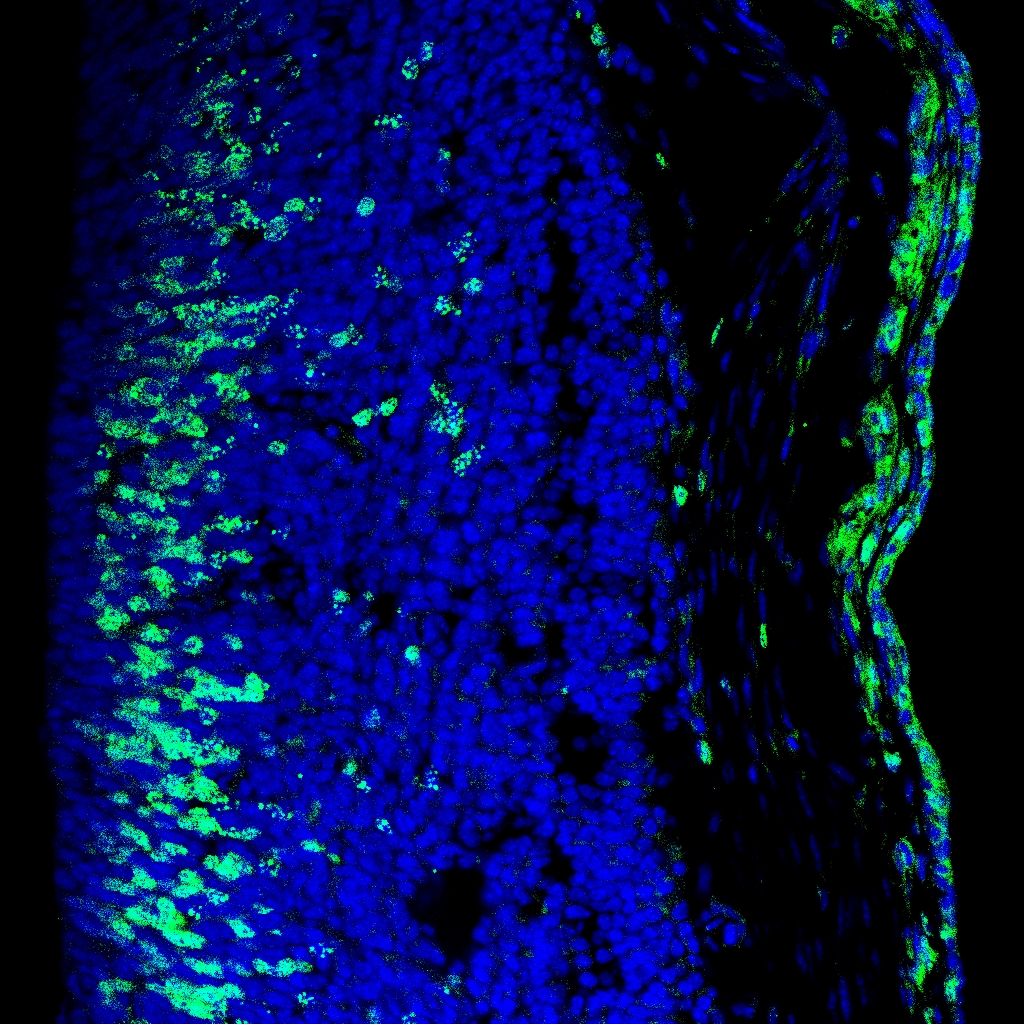

Supplement: Supplementary file 7 — Source data Fig. 5 [file 44319_2026_768_MOESM7_ESM.zip › Figure 5/5F/ctko.jpg]

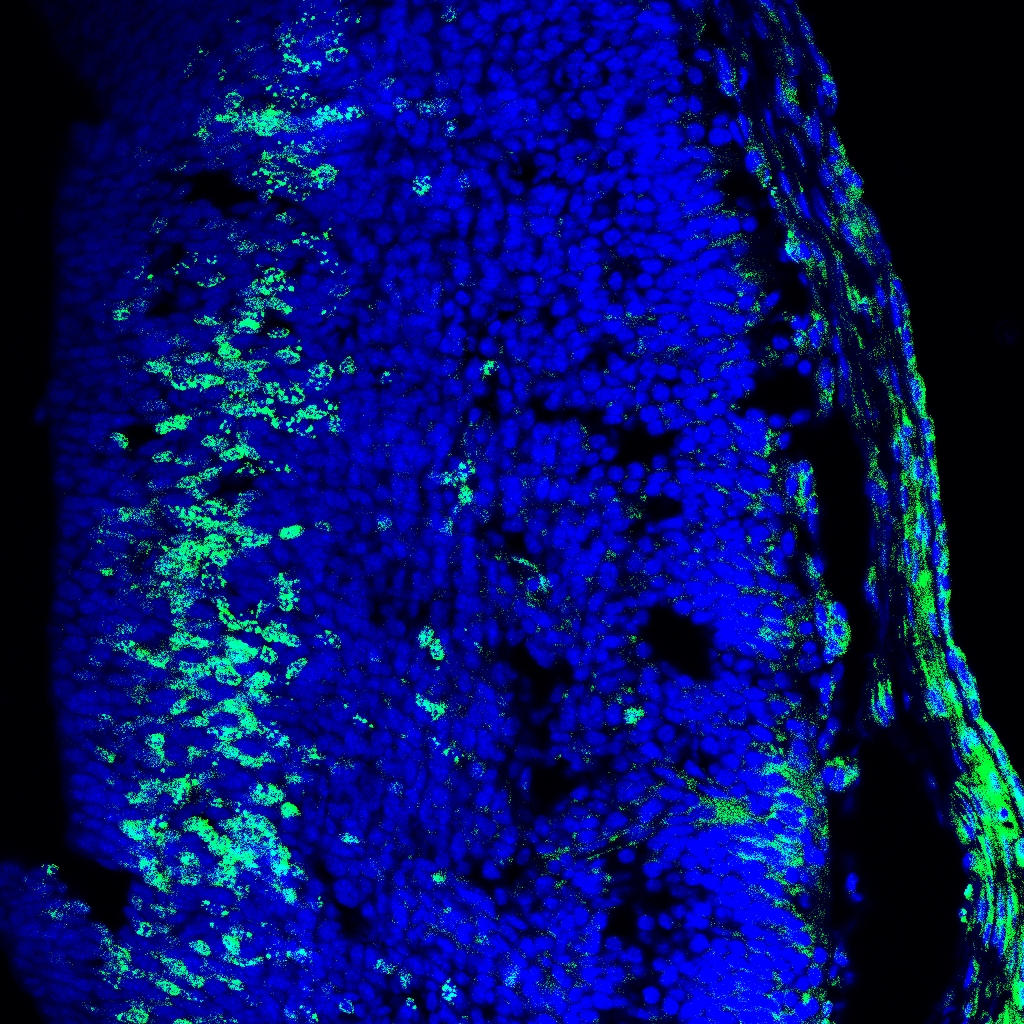

Supplement: Supplementary file 7 — Source data Fig. 5 [file 44319_2026_768_MOESM7_ESM.zip › Figure 5/5F/ctrl.jpg]

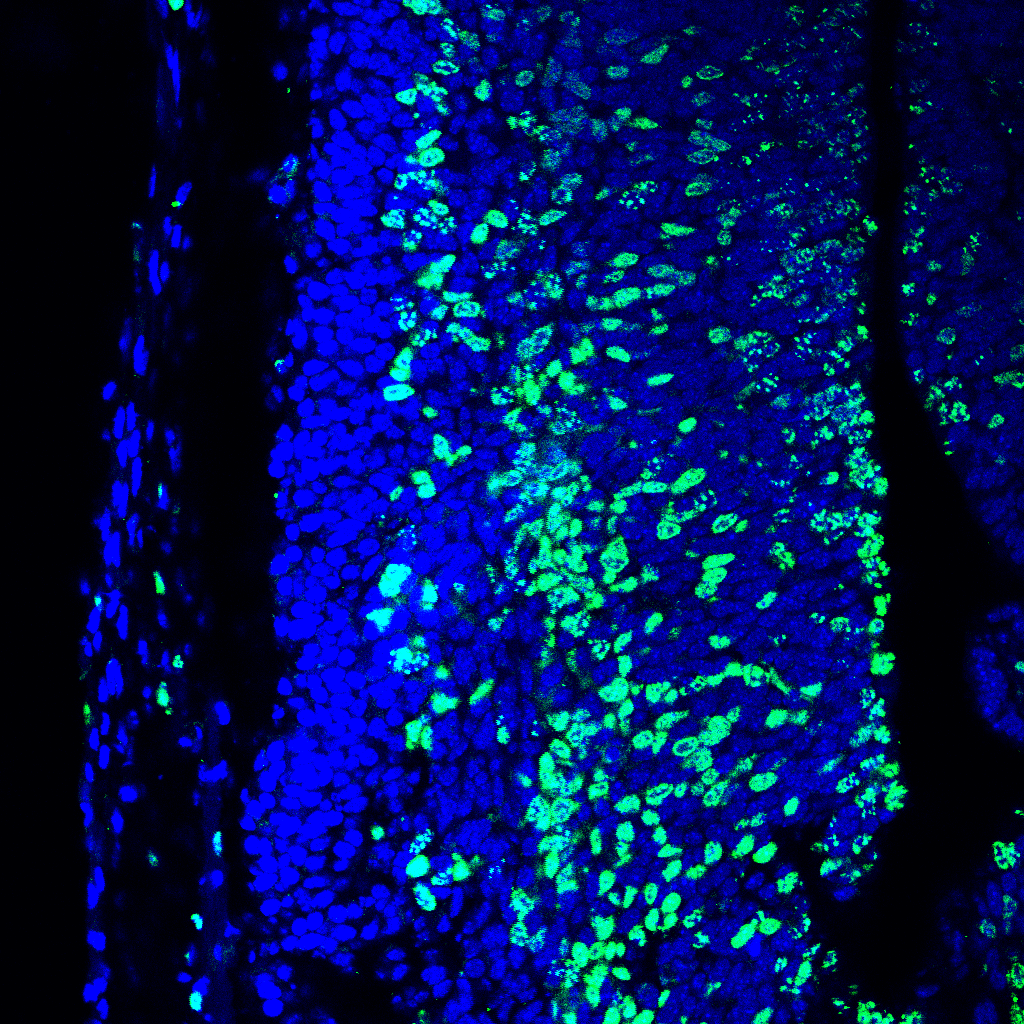

Supplement: Supplementary file 7 — Source data Fig. 5 [file 44319_2026_768_MOESM7_ESM.zip › Figure 5/5H/ctko.tif]

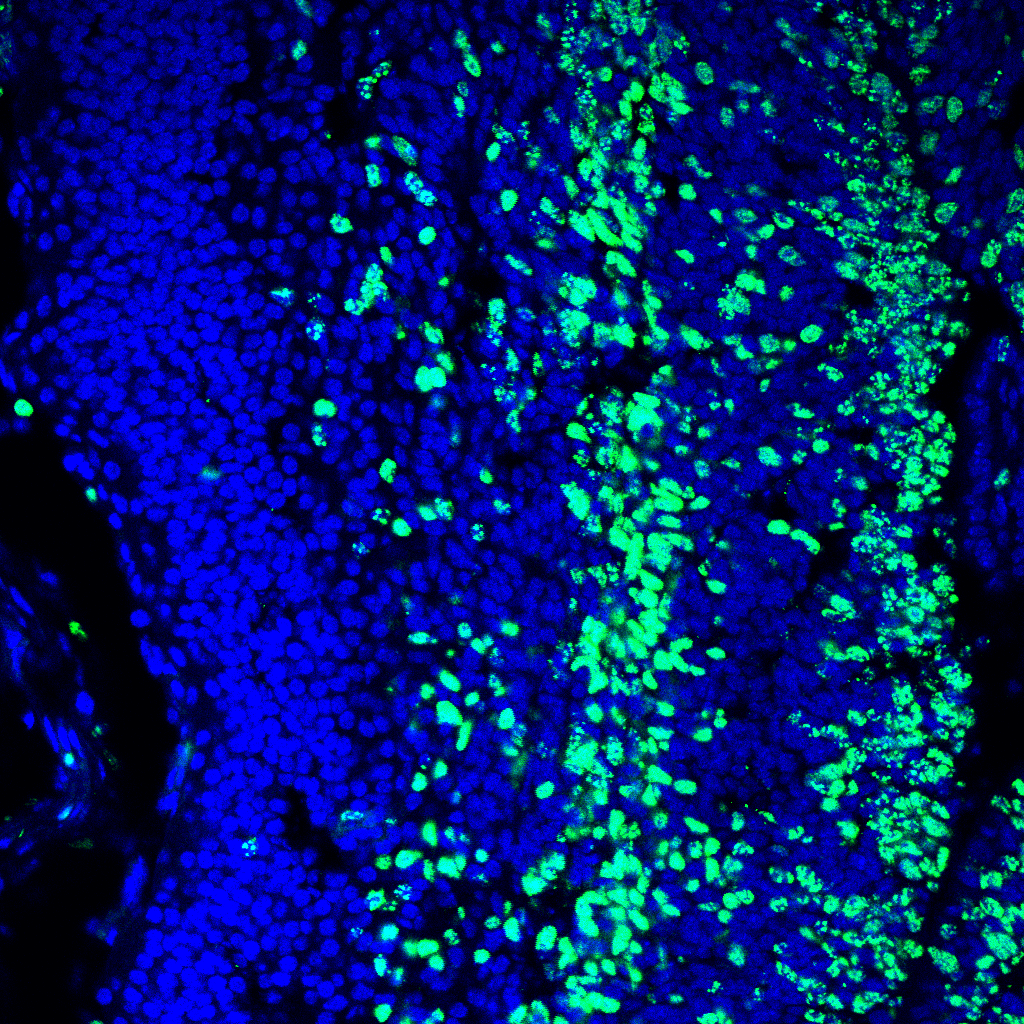

Supplement: Supplementary file 7 — Source data Fig. 5 [file 44319_2026_768_MOESM7_ESM.zip › Figure 5/5H/ctrl.tif]

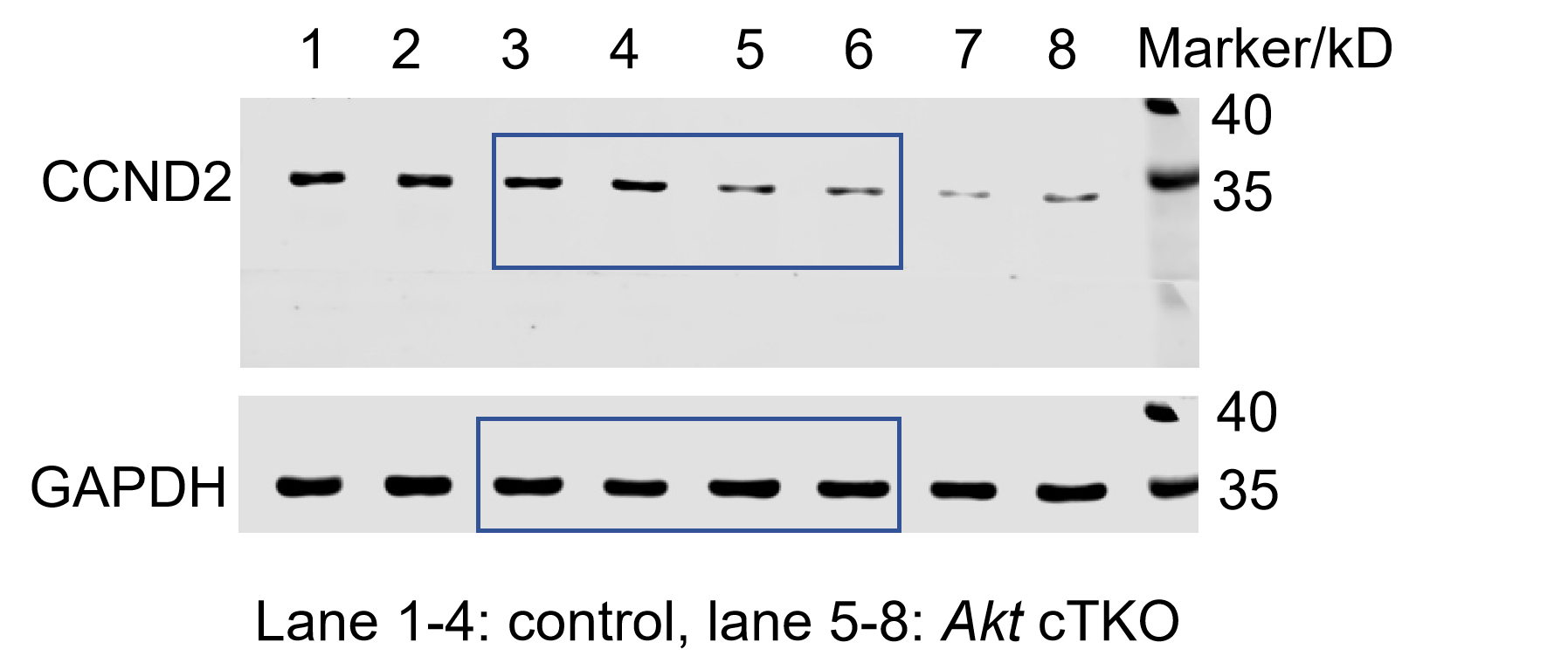

Supplement: Supplementary file 8 — Source data Fig. 6 [file 44319_2026_768_MOESM8_ESM.zip › Figure 6/6A/6A.tif]

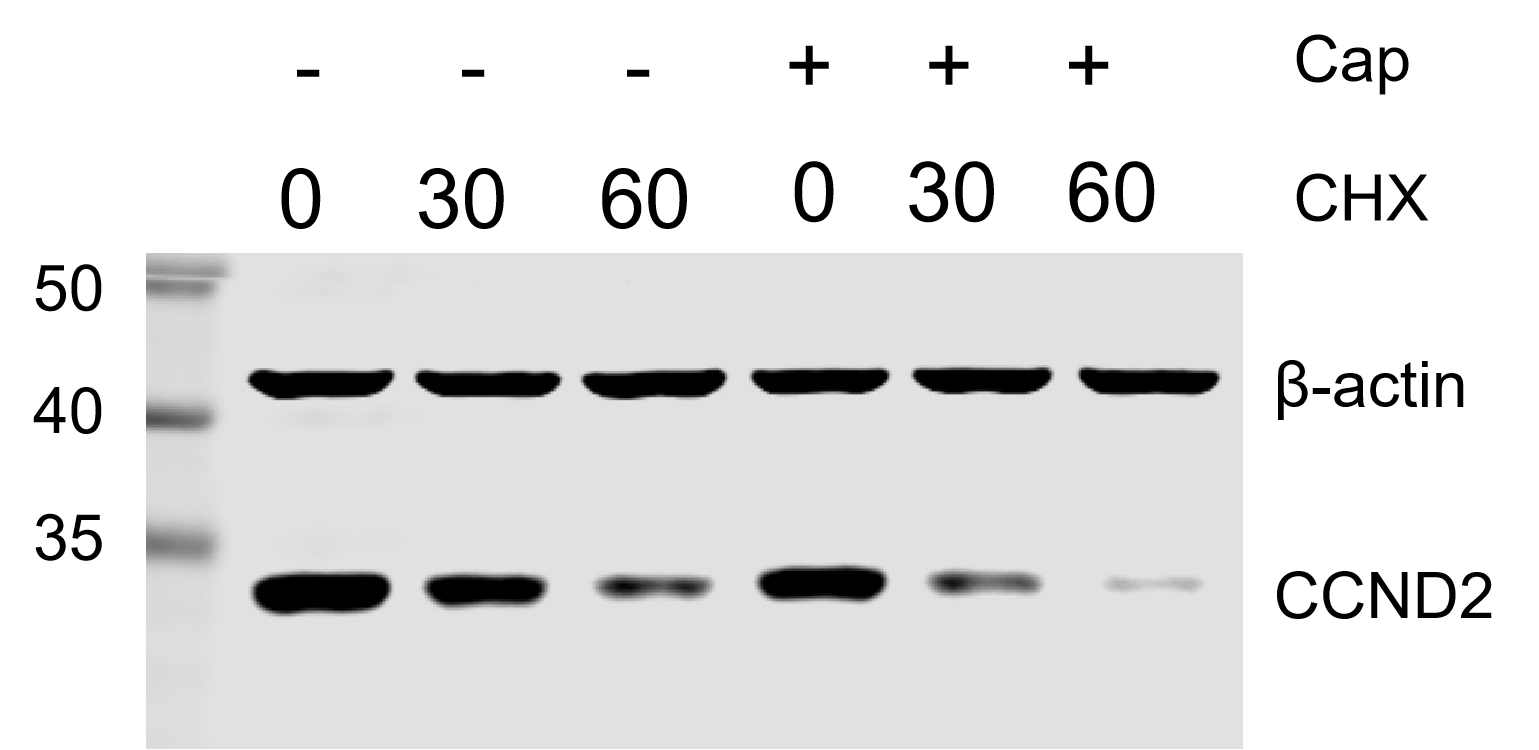

Supplement: Supplementary file 8 — Source data Fig. 6 [file 44319_2026_768_MOESM8_ESM.zip › Figure 6/6D/6D.tif]

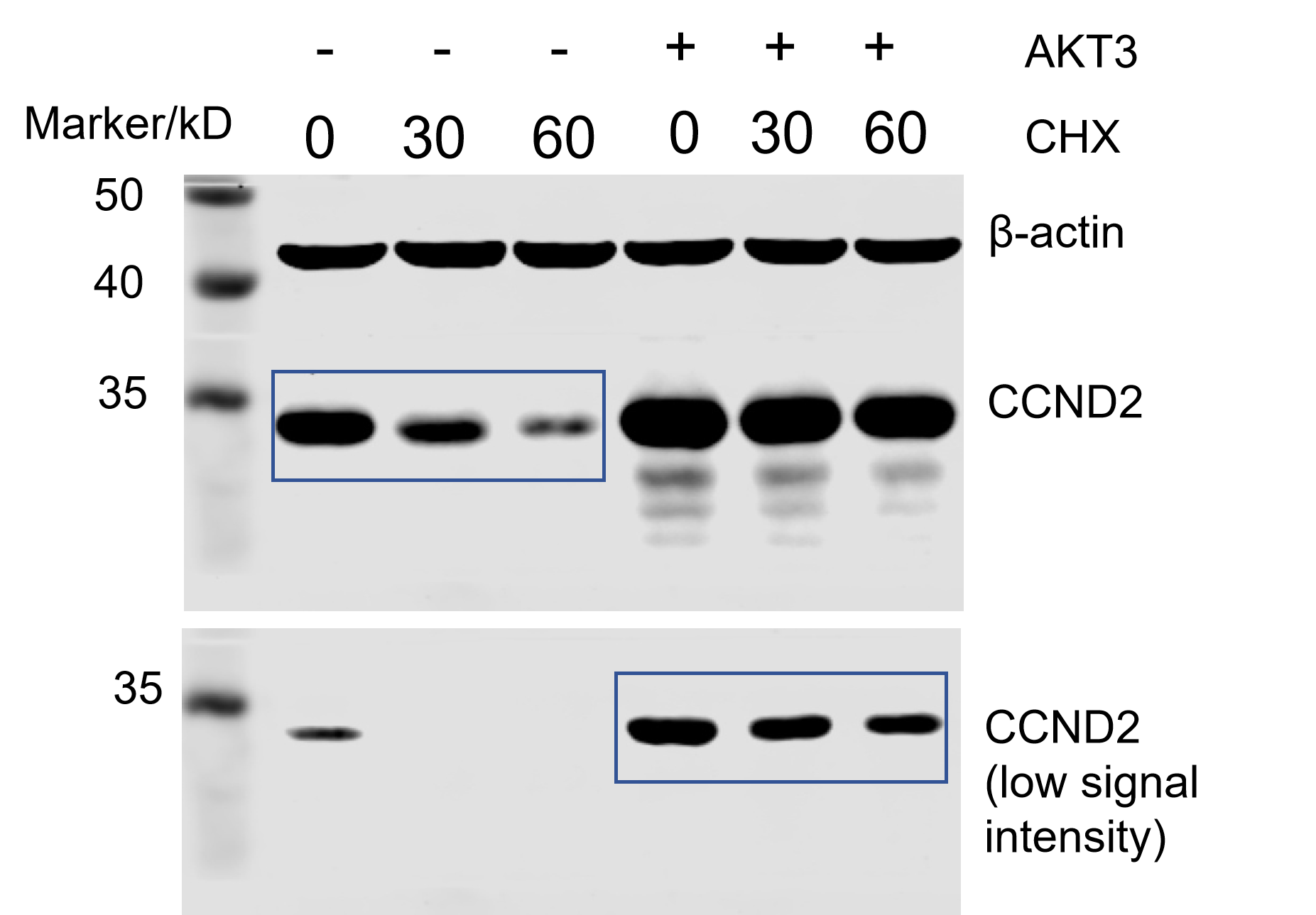

Supplement: Supplementary file 8 — Source data Fig. 6 [file 44319_2026_768_MOESM8_ESM.zip › Figure 6/6F/6F.tif]

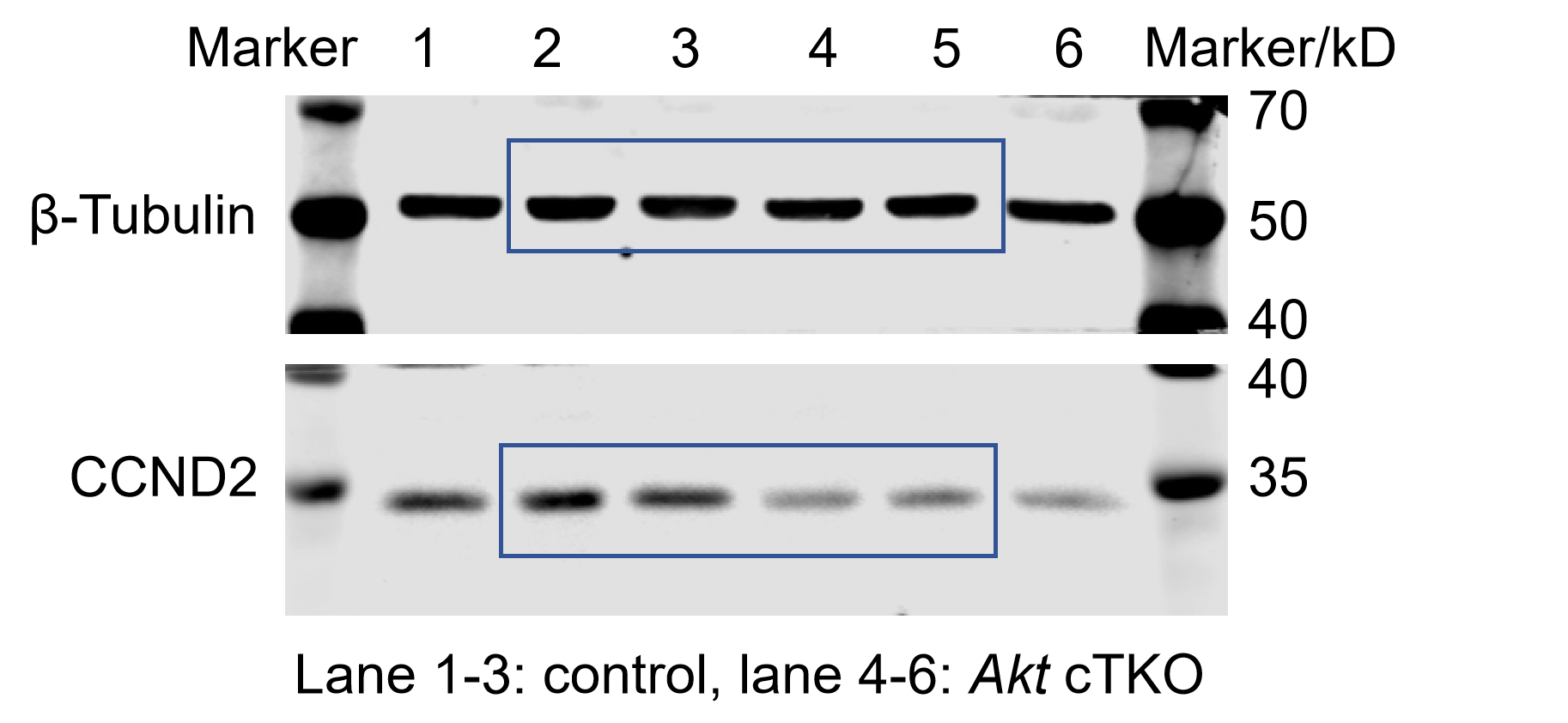

Supplement: Supplementary file 8 — Source data Fig. 6 [file 44319_2026_768_MOESM8_ESM.zip › Figure 6/6H/6H.tif]

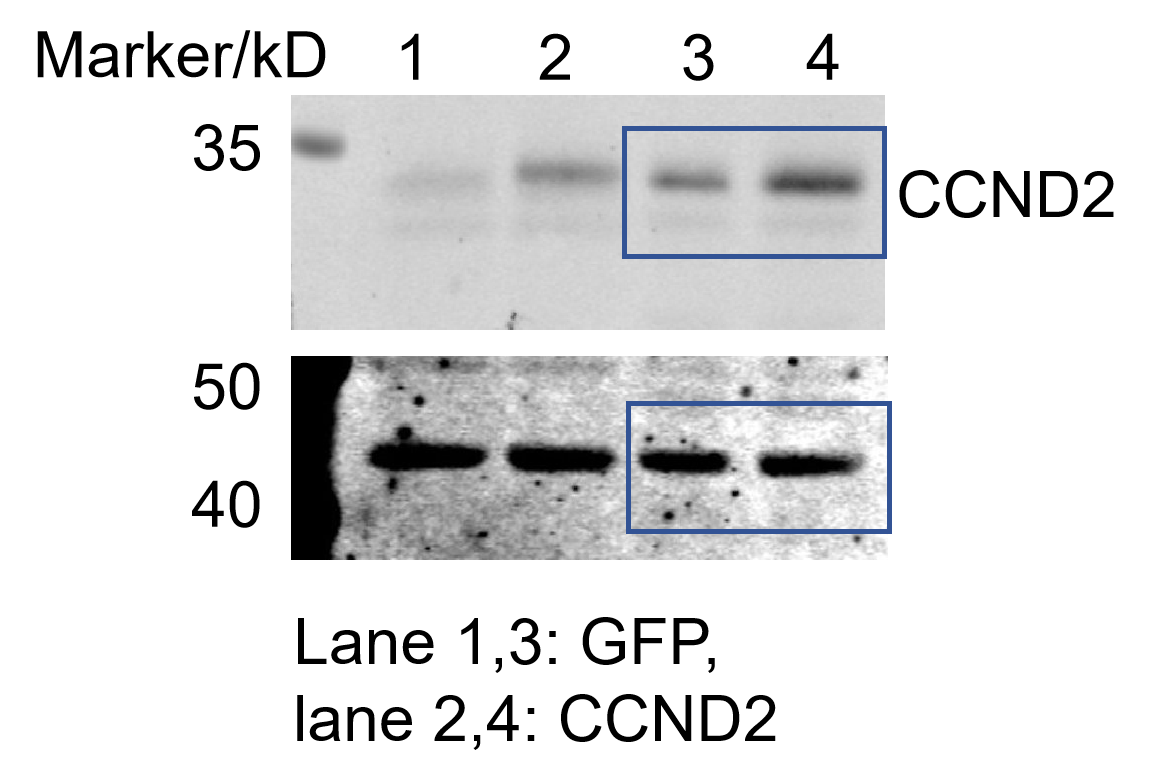

Supplement: Supplementary file 8 — Source data Fig. 6 [file 44319_2026_768_MOESM8_ESM.zip › Figure 6/6J/6J.tif]

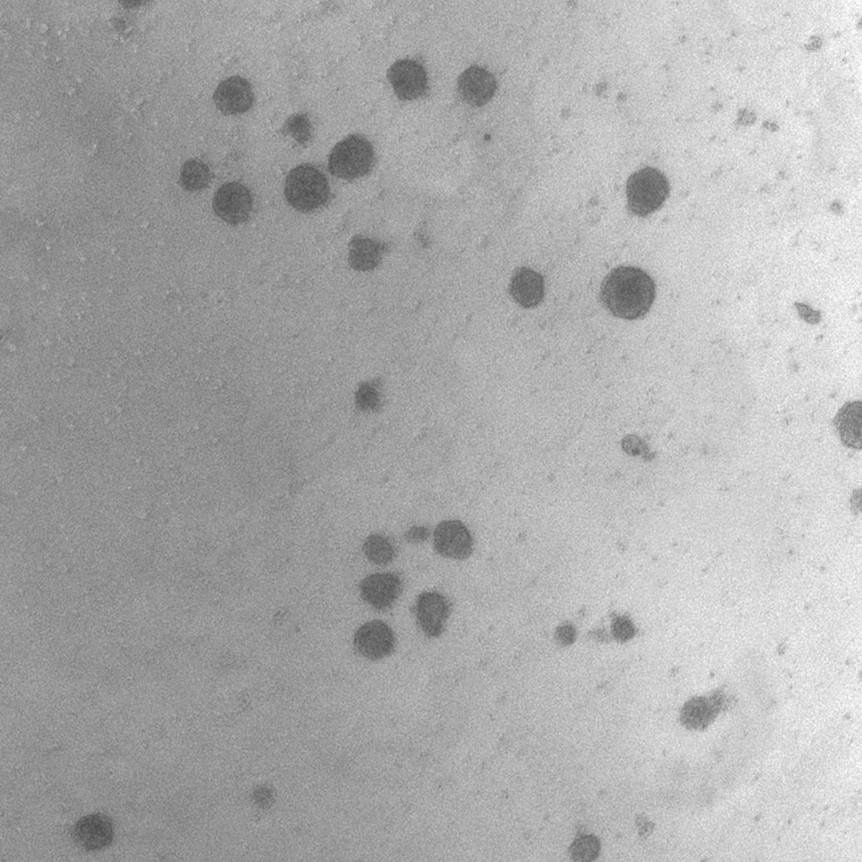

Supplement: Supplementary file 8 — Source data Fig. 6 [file 44319_2026_768_MOESM8_ESM.zip › Figure 6/6L/CCND2.jpg]

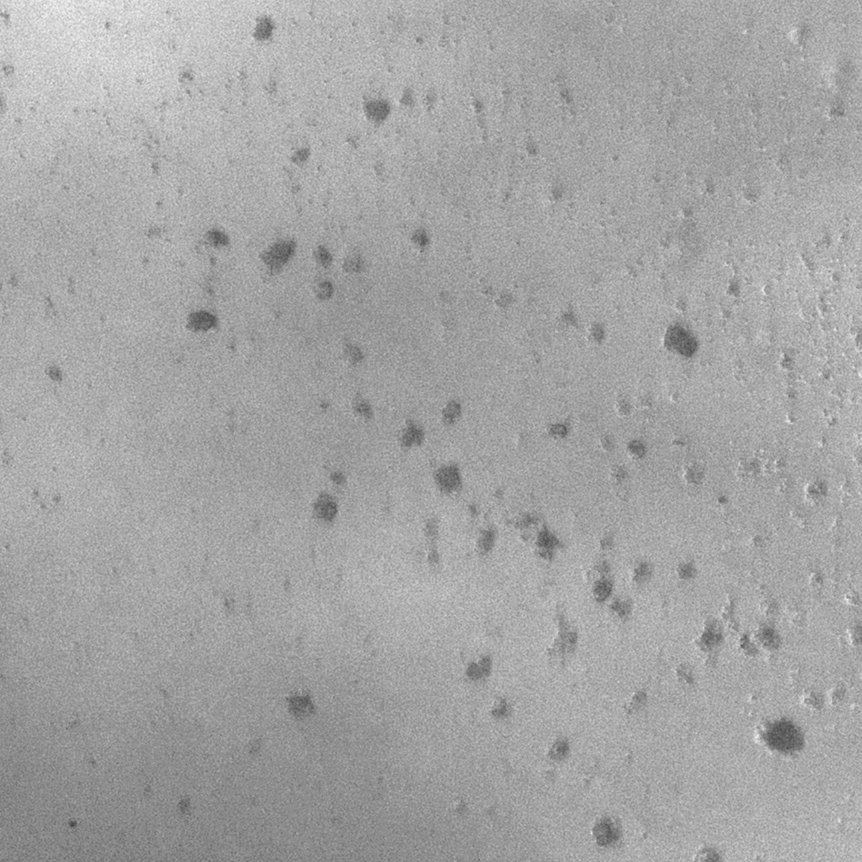

Supplement: Supplementary file 8 — Source data Fig. 6 [file 44319_2026_768_MOESM8_ESM.zip › Figure 6/6L/GFP.jpg]

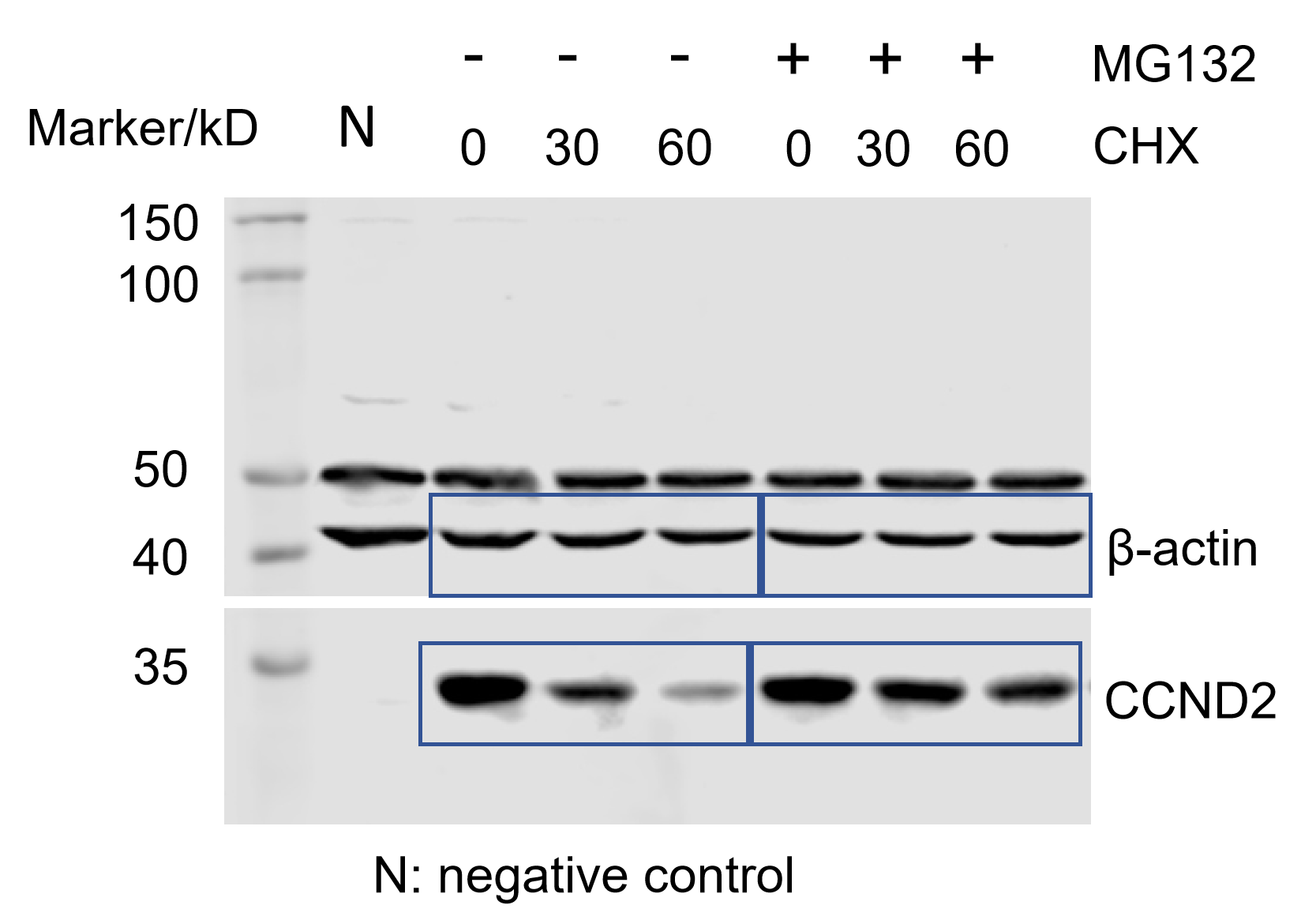

Supplement: Supplementary file 8 — Source data Fig. 6 [file 44319_2026_768_MOESM8_ESM.zip › Figure 6/6N/6N.tif]

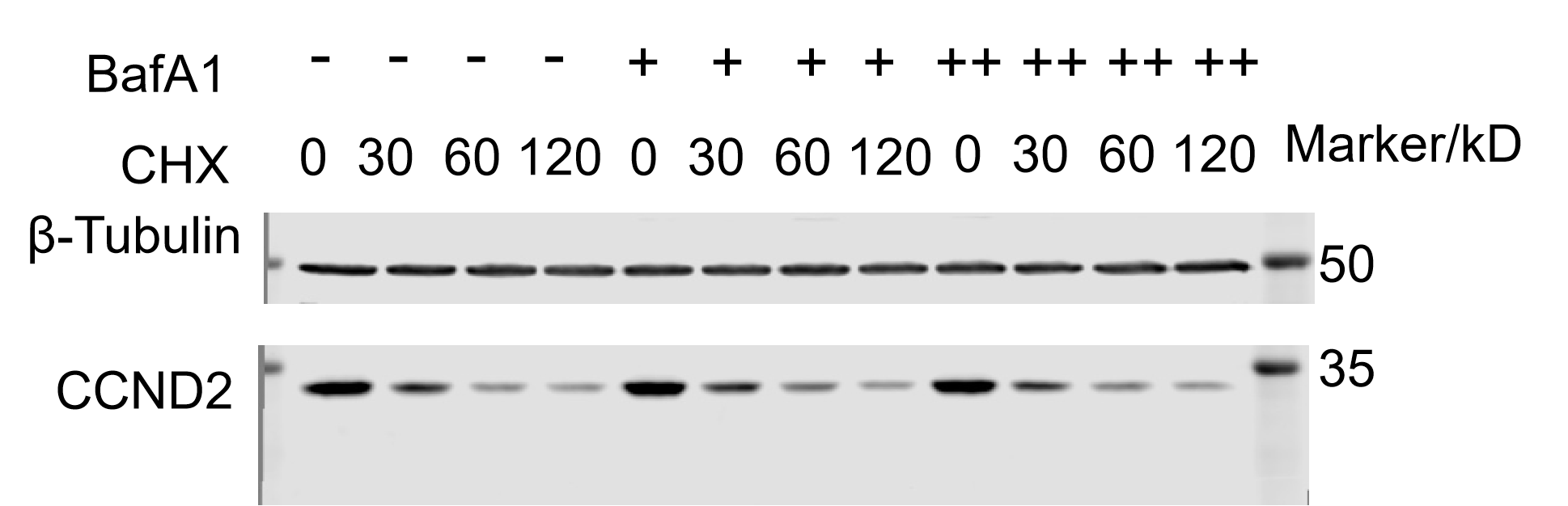

Supplement: Supplementary file 8 — Source data Fig. 6 [file 44319_2026_768_MOESM8_ESM.zip › Figure 6/6P/6P.tif]

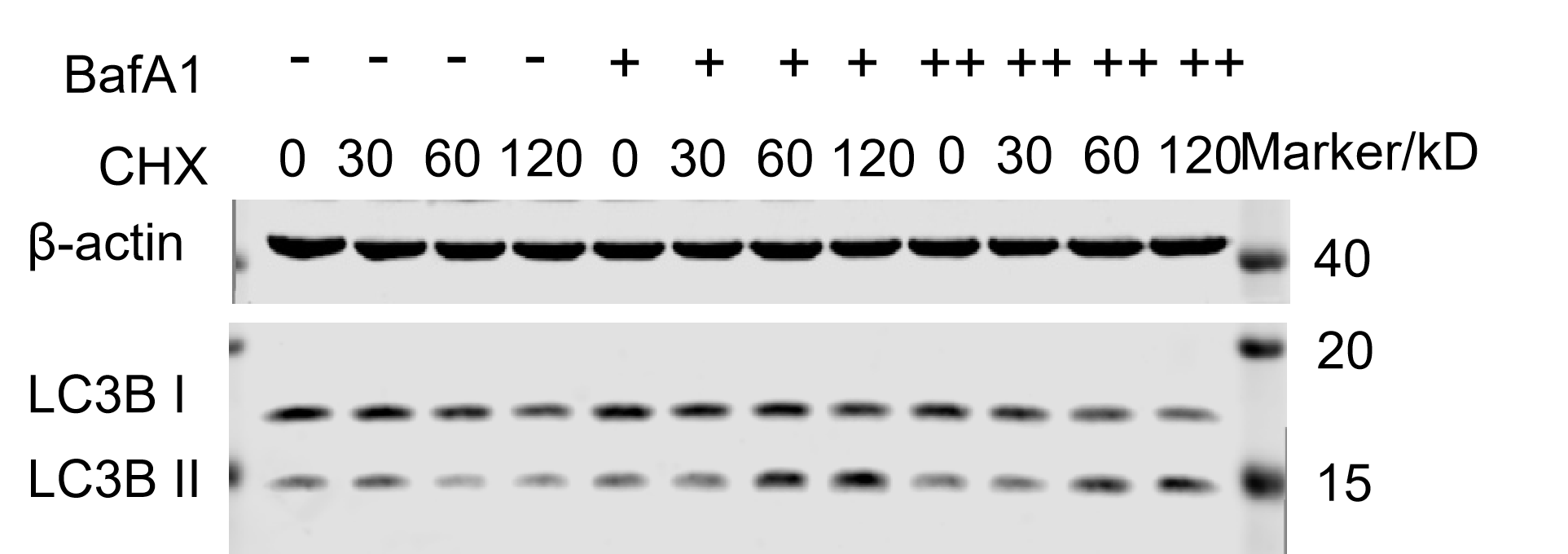

Supplement: Supplementary file 8 — Source data Fig. 6 [file 44319_2026_768_MOESM8_ESM.zip › Figure 6/6R/6R.tif]

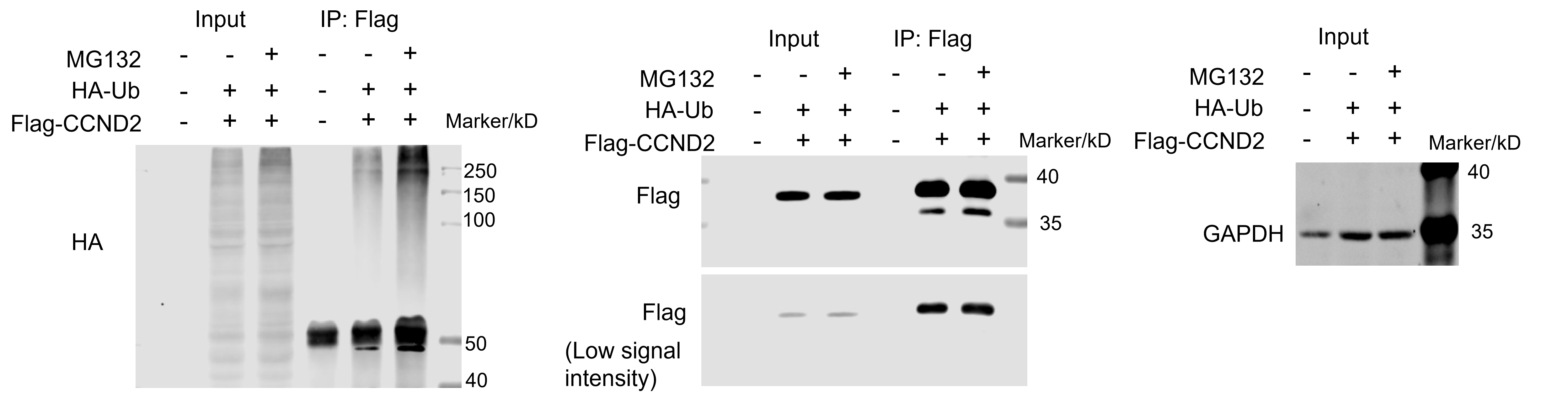

Supplement: Supplementary file 8 — Source data Fig. 6 [file 44319_2026_768_MOESM8_ESM.zip › Figure 6/6T/6T.tif]

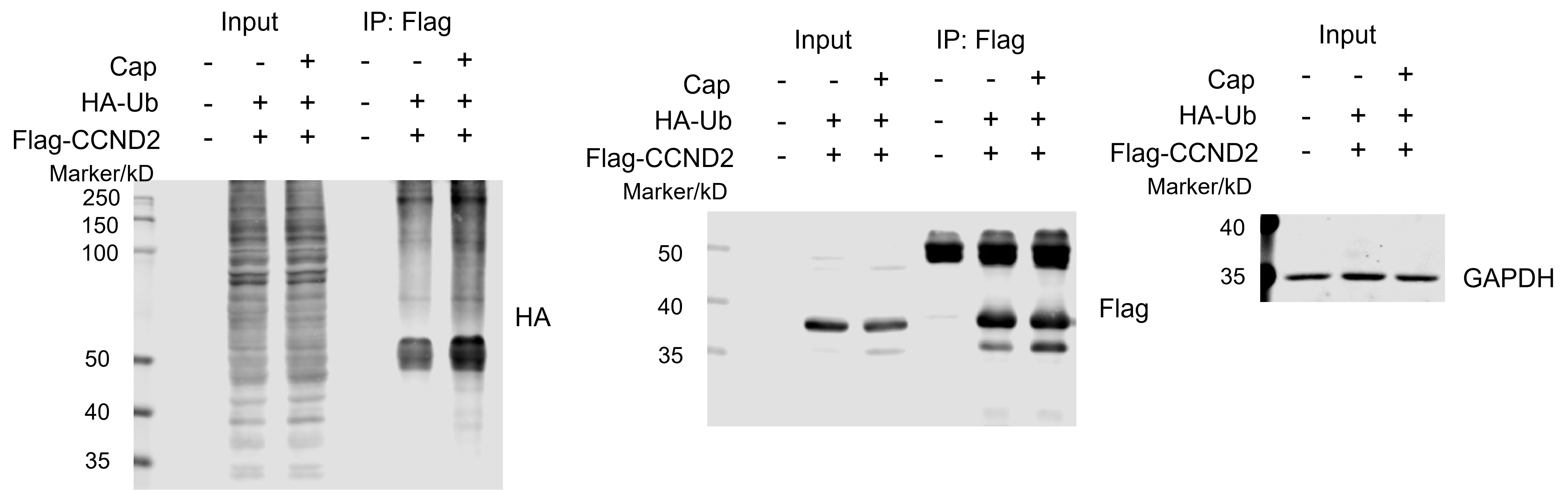

Supplement: Supplementary file 8 — Source data Fig. 6 [file 44319_2026_768_MOESM8_ESM.zip › Figure 6/6U/6U.tif]

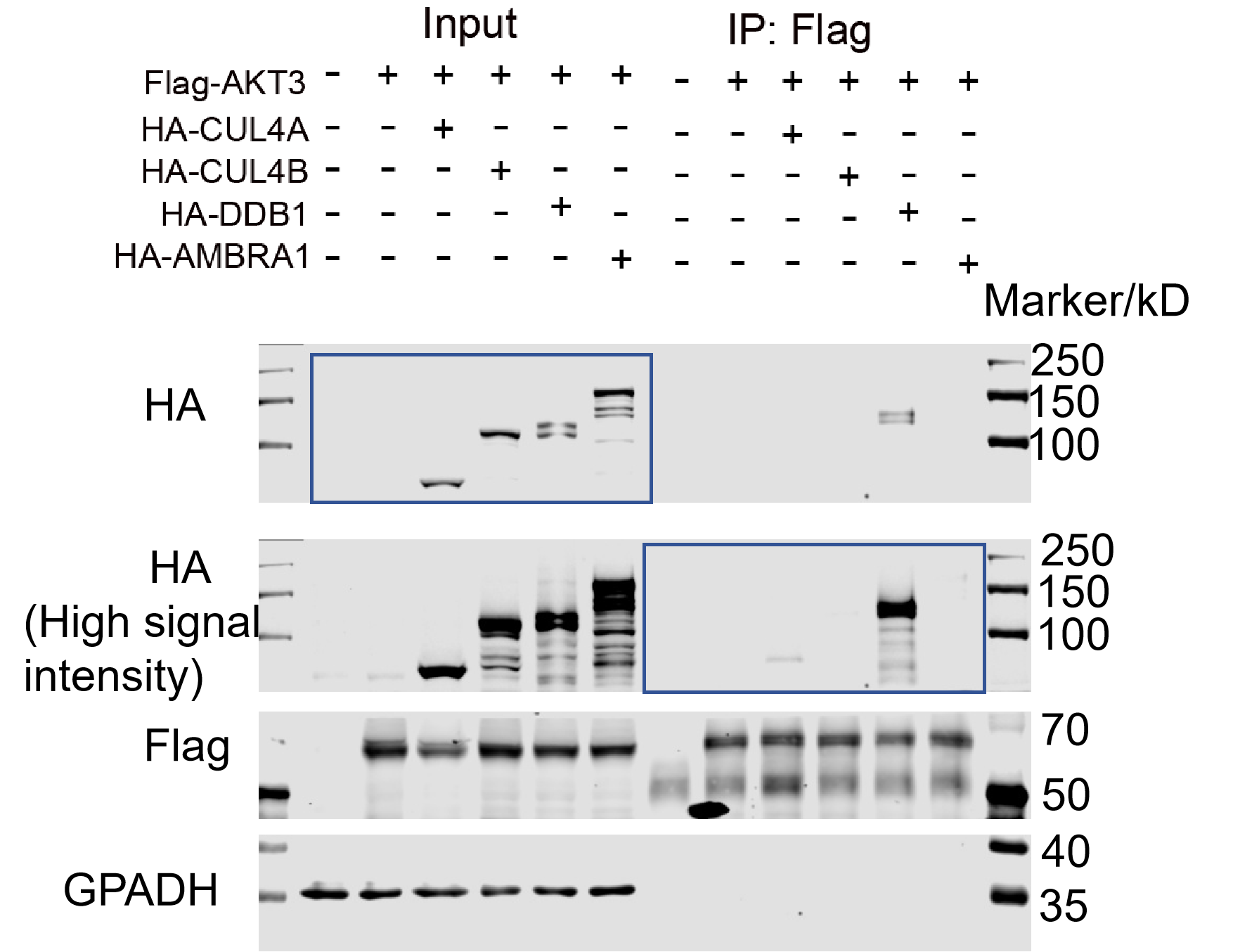

Supplement: Supplementary file 9 — Source data Fig. 7 [file 44319_2026_768_MOESM9_ESM.zip › Figure 7/7A/7A.tif]

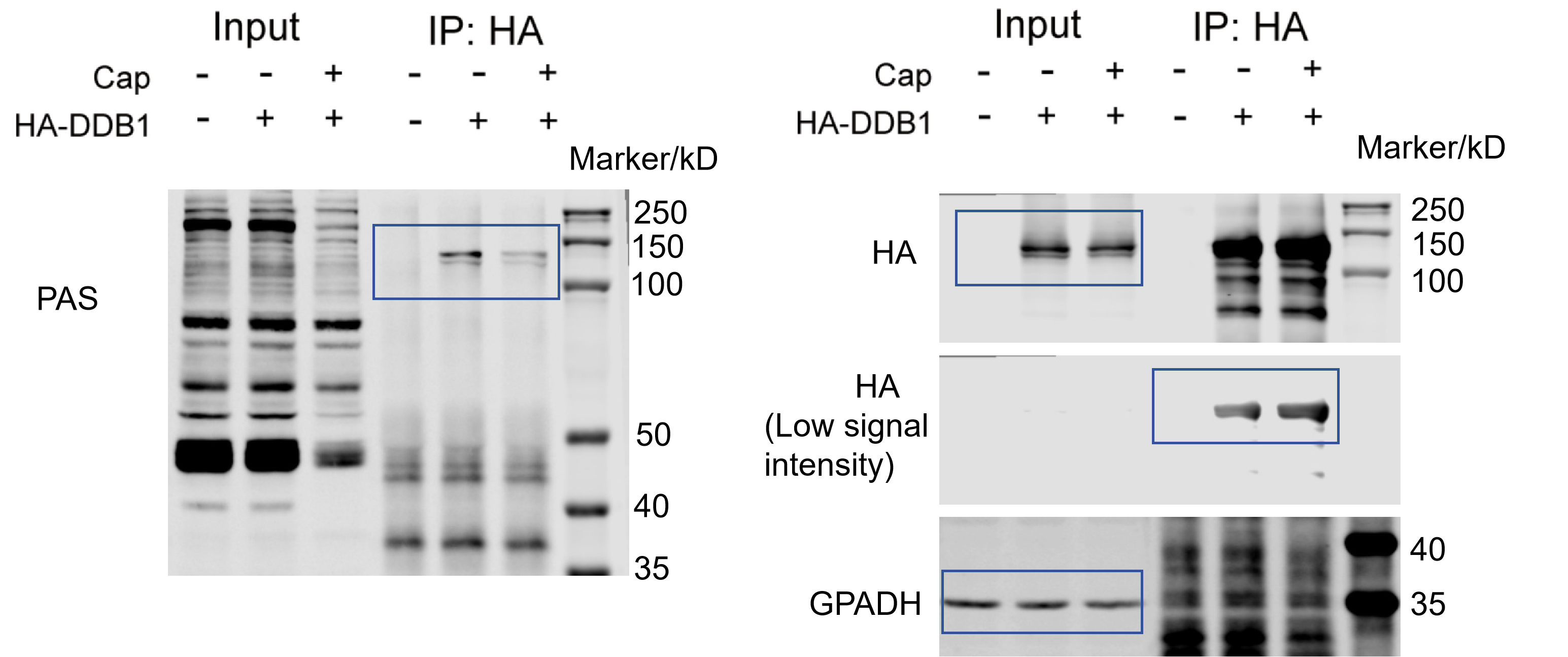

Supplement: Supplementary file 9 — Source data Fig. 7 [file 44319_2026_768_MOESM9_ESM.zip › Figure 7/7B/7B.tif]

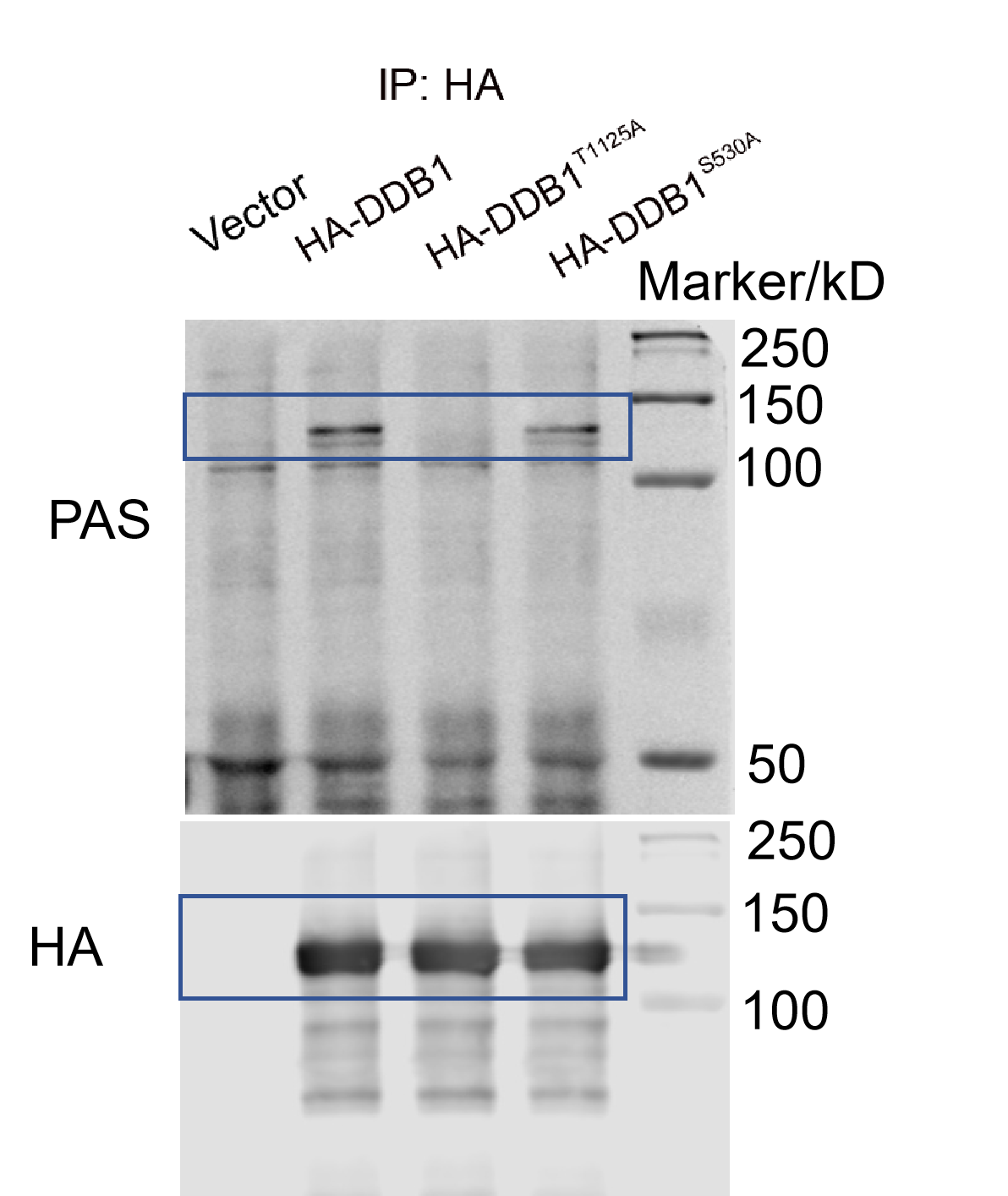

Supplement: Supplementary file 9 — Source data Fig. 7 [file 44319_2026_768_MOESM9_ESM.zip › Figure 7/7D/7D.tif]

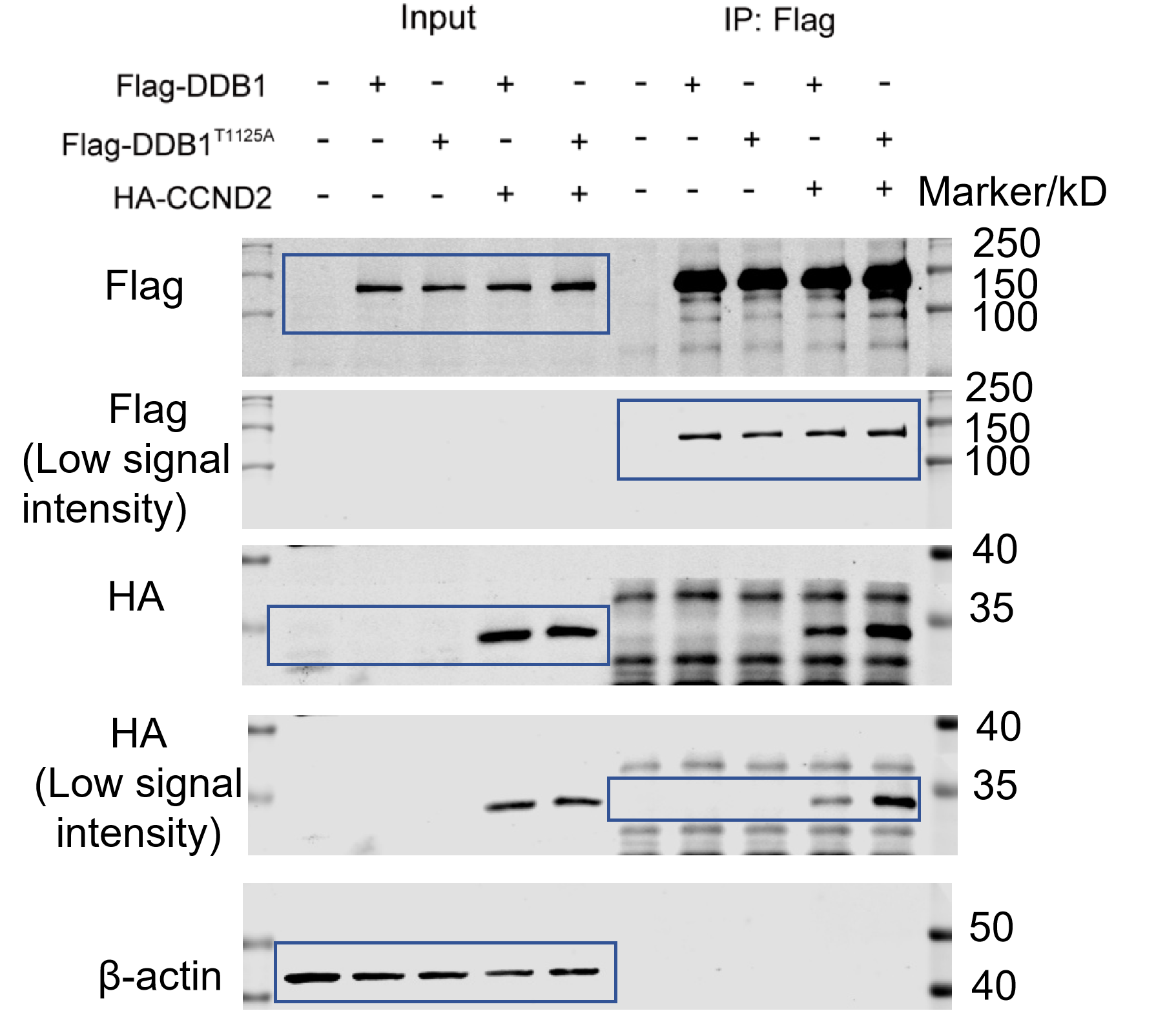

Supplement: Supplementary file 9 — Source data Fig. 7 [file 44319_2026_768_MOESM9_ESM.zip › Figure 7/7E/7E.tif]

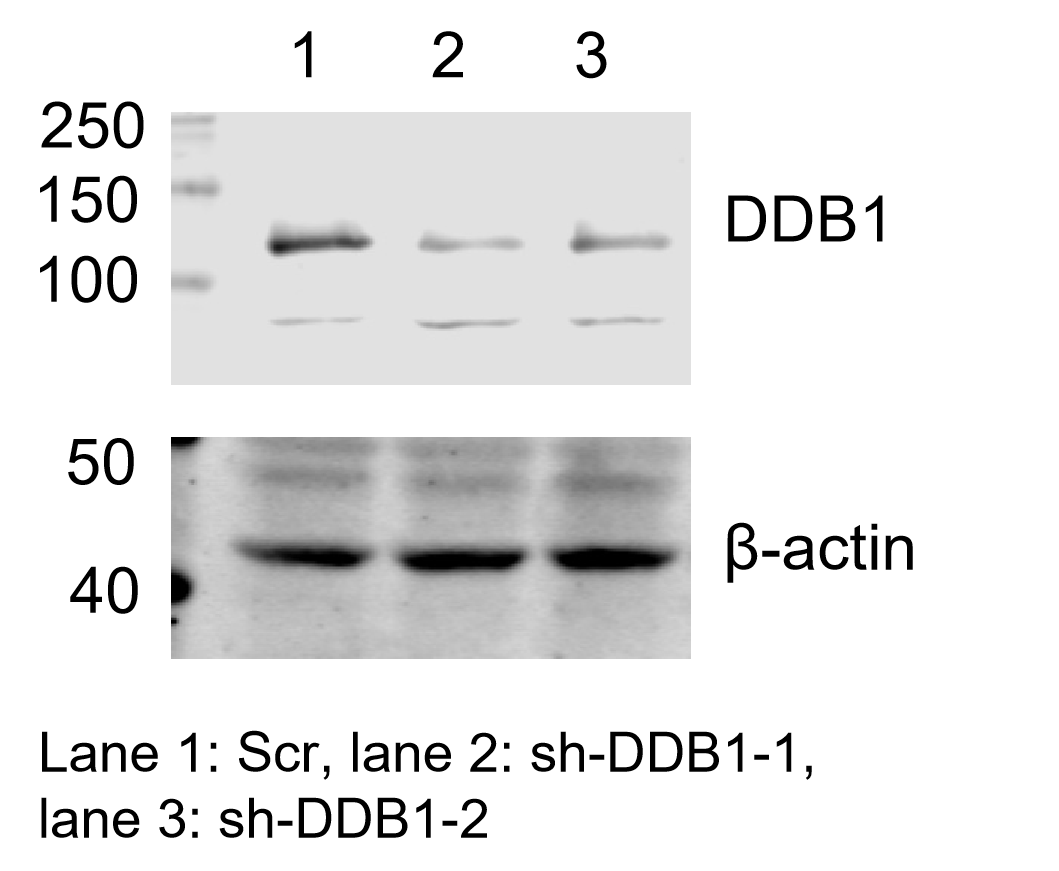

Supplement: Supplementary file 9 — Source data Fig. 7 [file 44319_2026_768_MOESM9_ESM.zip › Figure 7/7G/7G.tif]

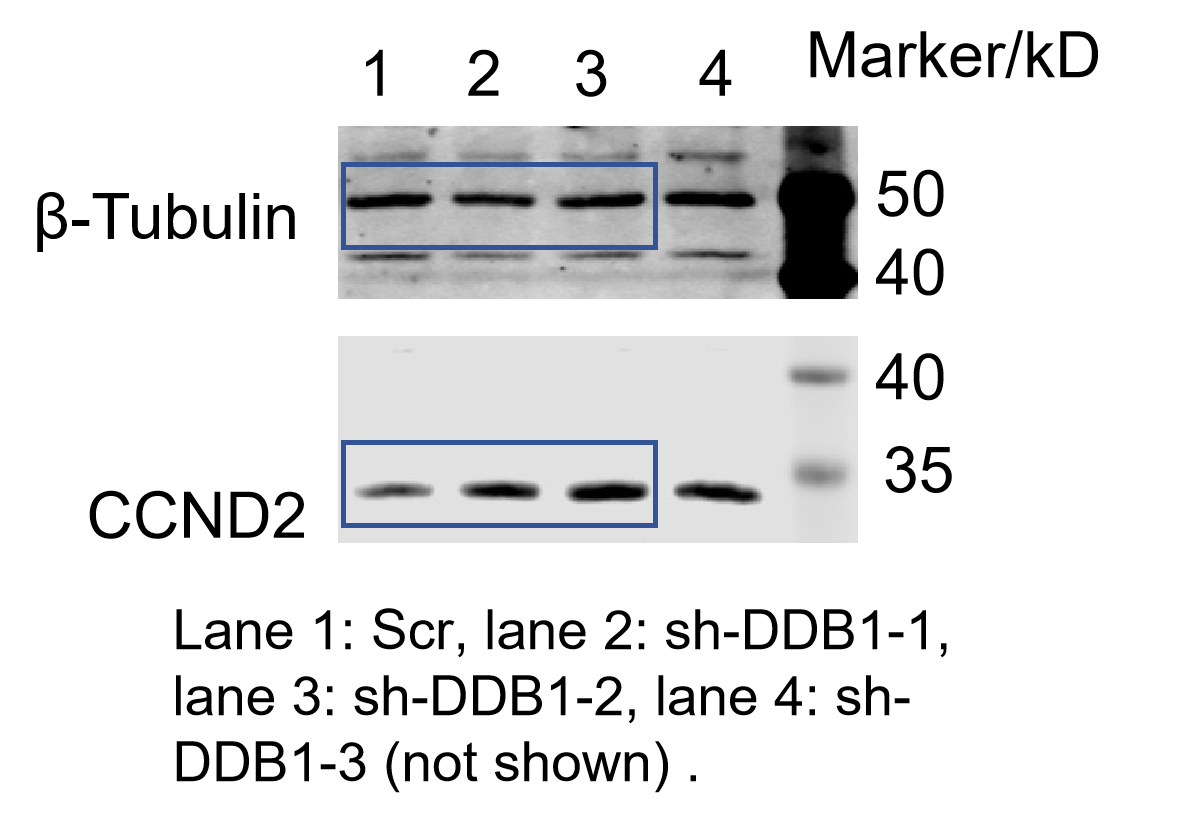

Supplement: Supplementary file 9 — Source data Fig. 7 [file 44319_2026_768_MOESM9_ESM.zip › Figure 7/7I/7I.tif]

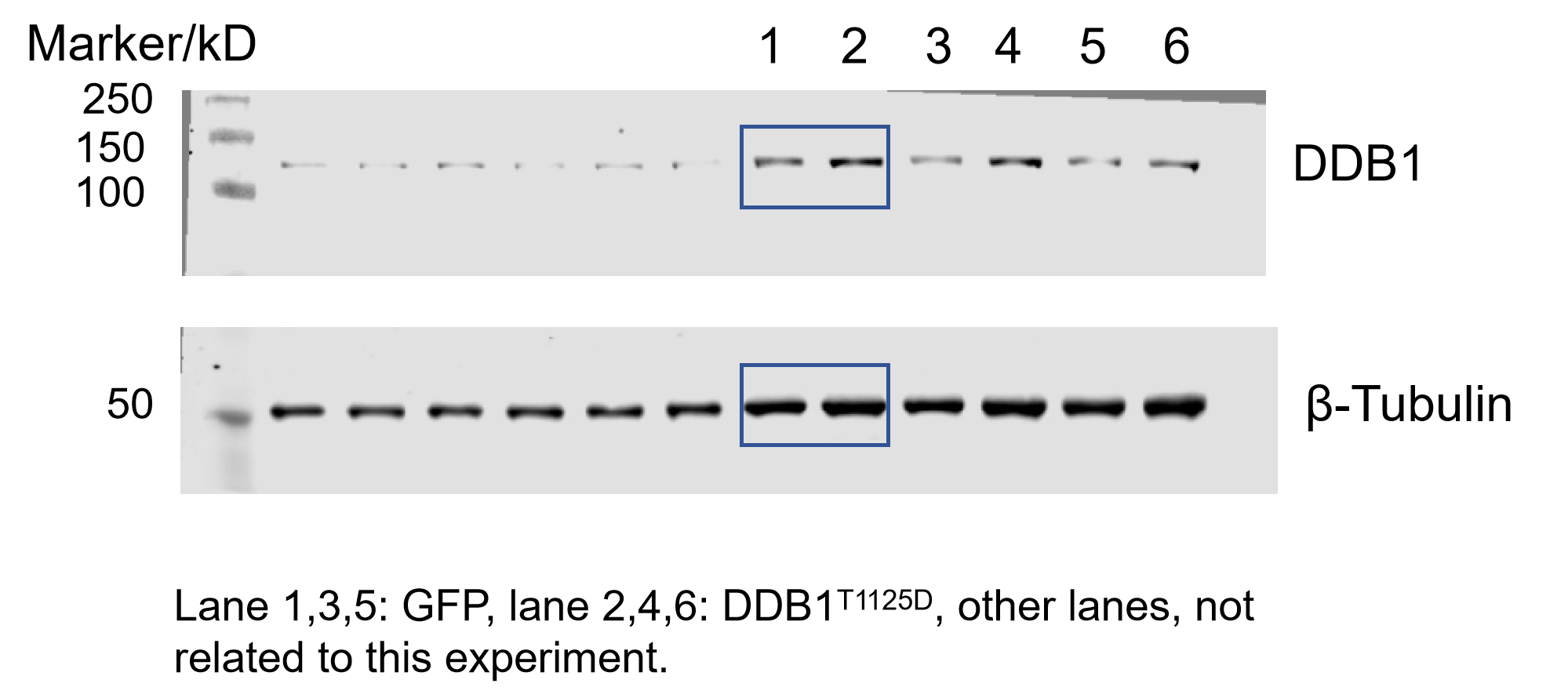

Supplement: Supplementary file 9 — Source data Fig. 7 [file 44319_2026_768_MOESM9_ESM.zip › Figure 7/7K/7K.tif]

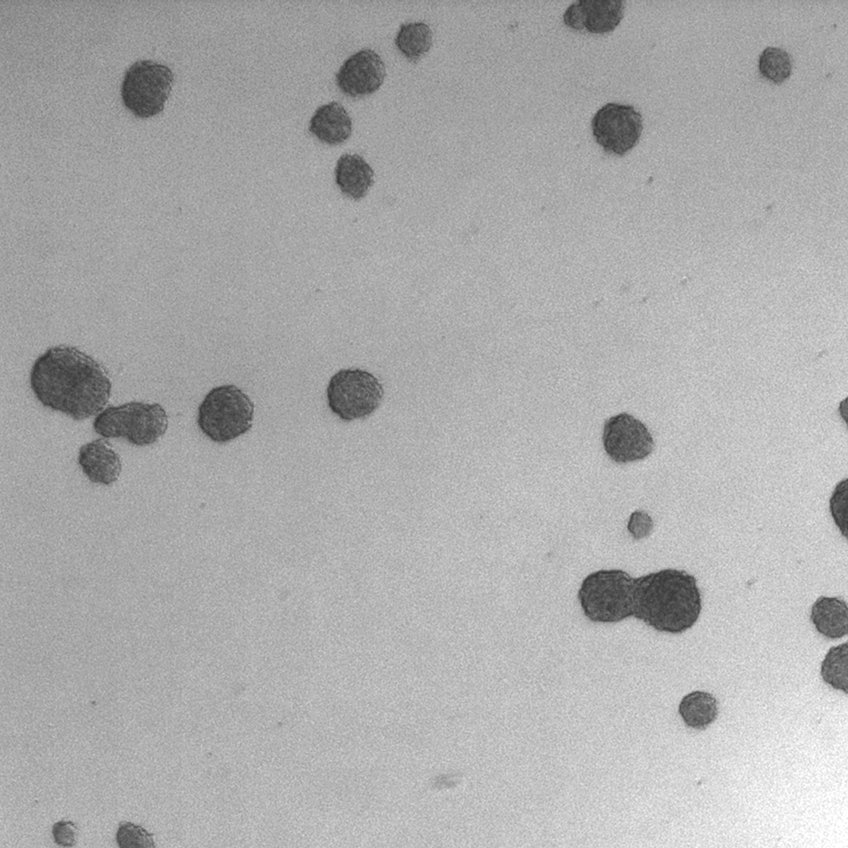

Supplement: Supplementary file 9 — Source data Fig. 7 [file 44319_2026_768_MOESM9_ESM.zip › Figure 7/7M/DDB1T1125D.jpg]

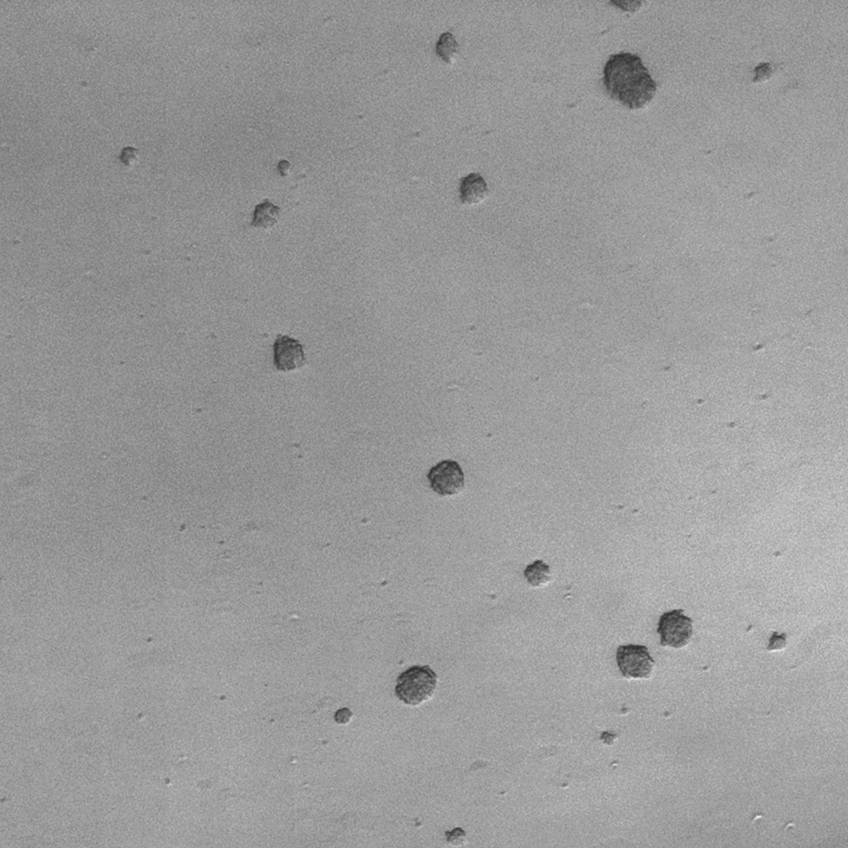

Supplement: Supplementary file 9 — Source data Fig. 7 [file 44319_2026_768_MOESM9_ESM.zip › Figure 7/7M/GFP.jpg]

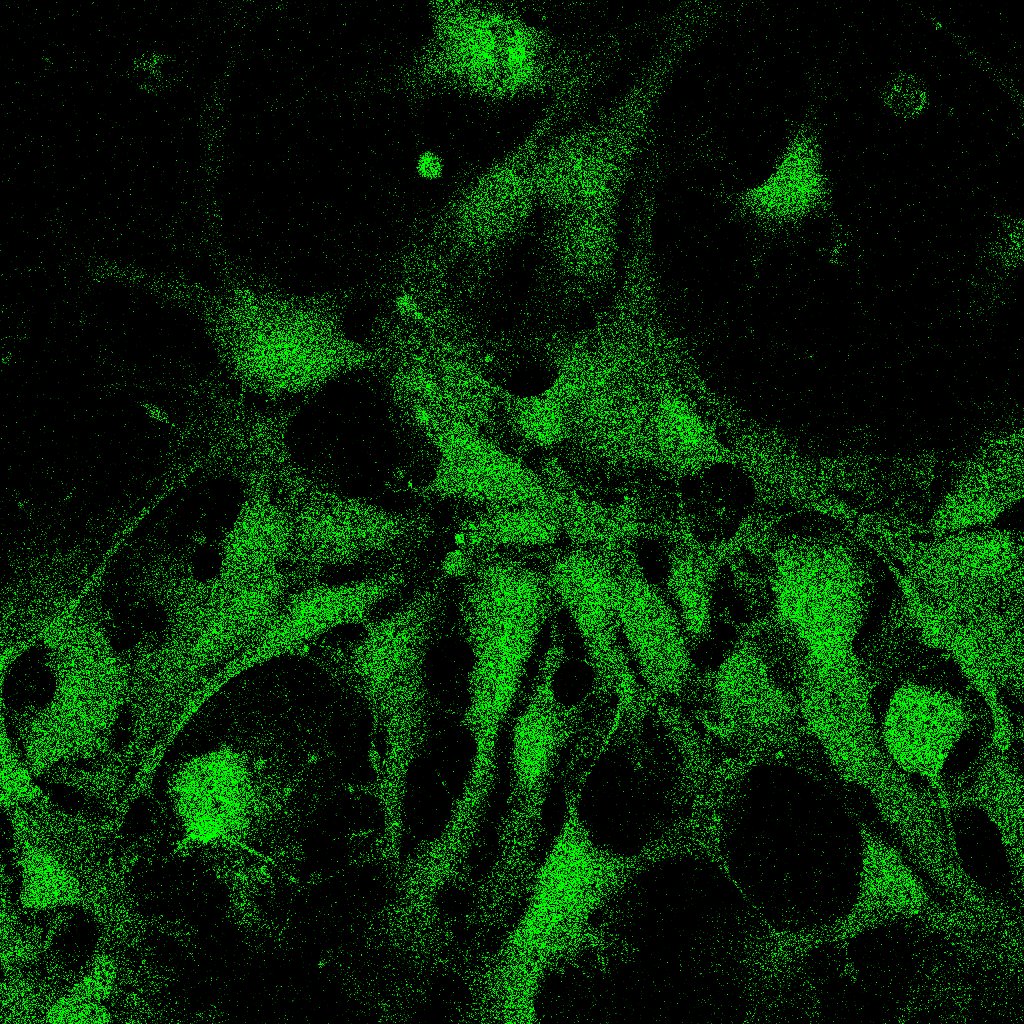

Supplement: Supplementary file 10 — Appendix Figures Source Data [file 44319_2026_768_MOESM10_ESM.zip › Appendix Figures/Appendix Figure S1/S1A/AKT.jpg]

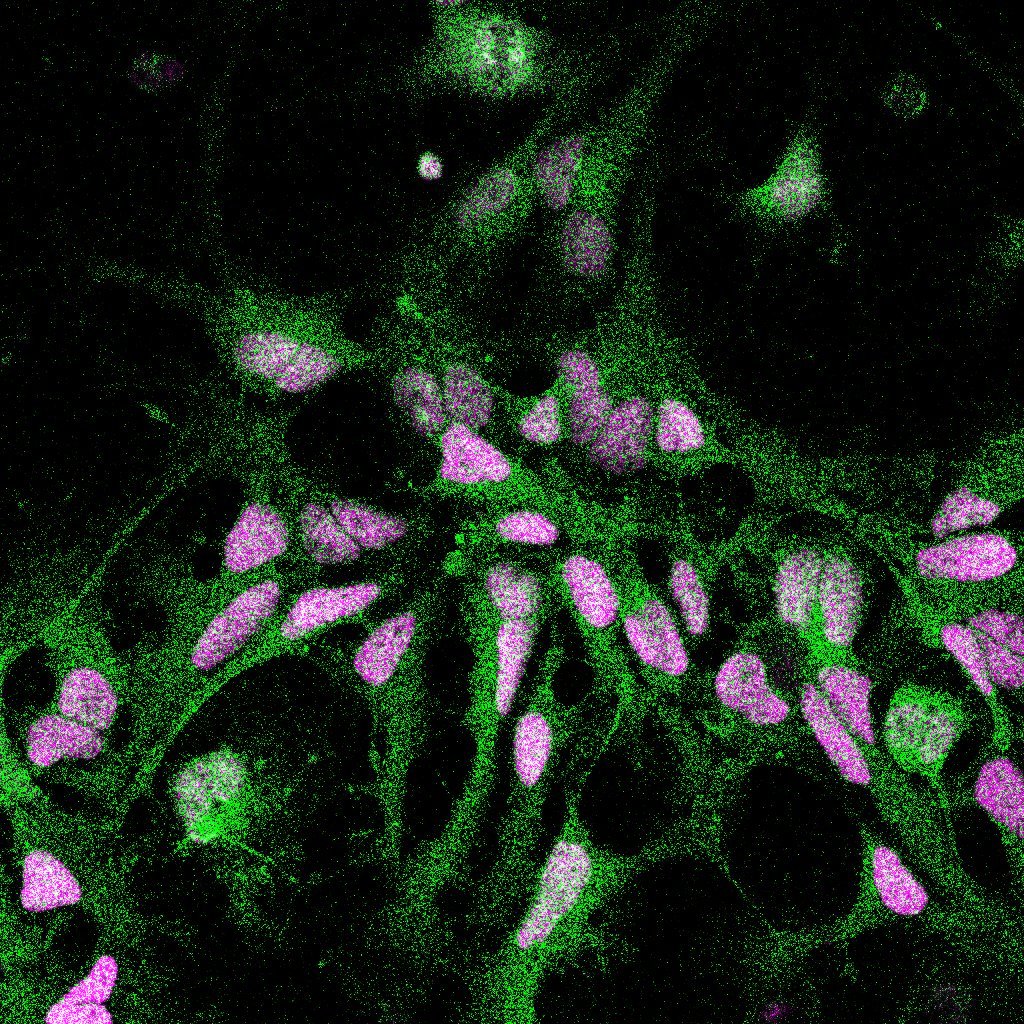

Supplement: Supplementary file 10 — Appendix Figures Source Data [file 44319_2026_768_MOESM10_ESM.zip › Appendix Figures/Appendix Figure S1/S1A/Merge.jpg]

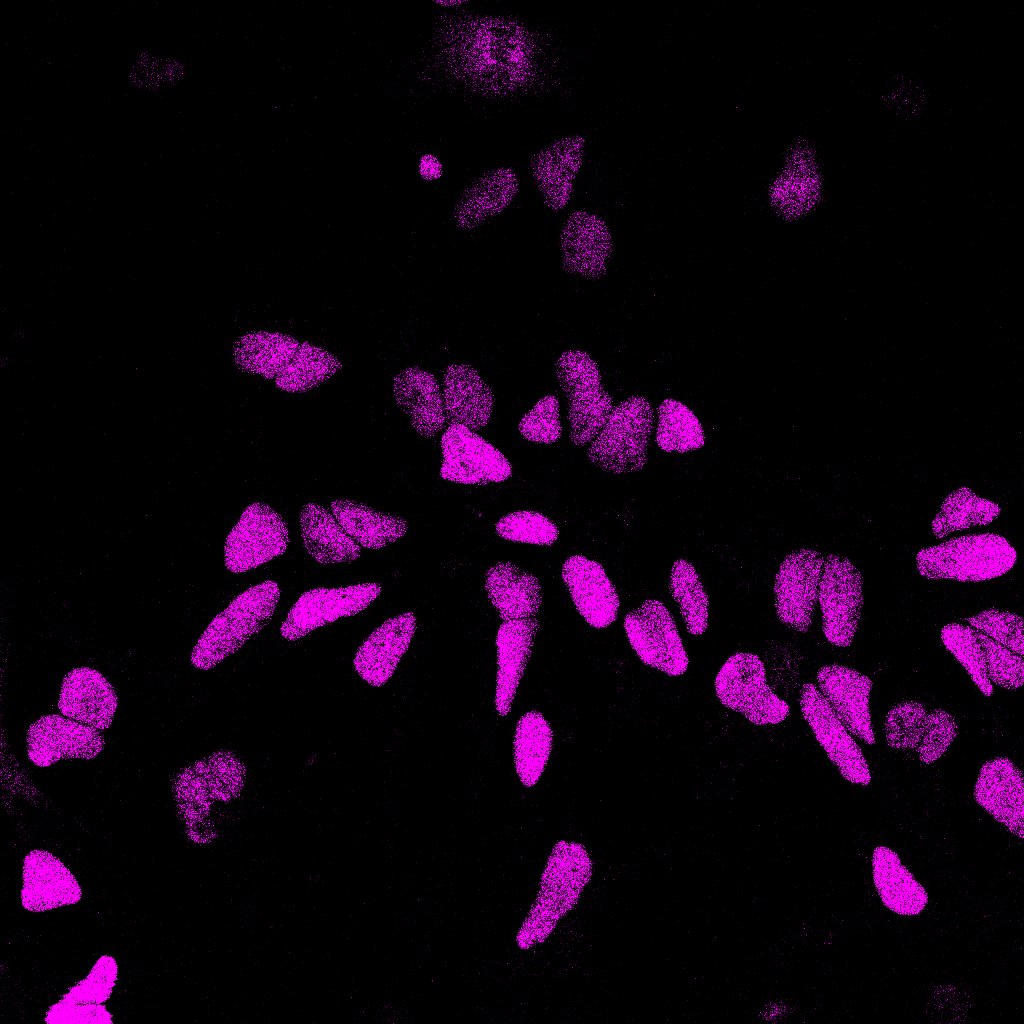

Supplement: Supplementary file 10 — Appendix Figures Source Data [file 44319_2026_768_MOESM10_ESM.zip › Appendix Figures/Appendix Figure S1/S1A/SOX2.jpg]

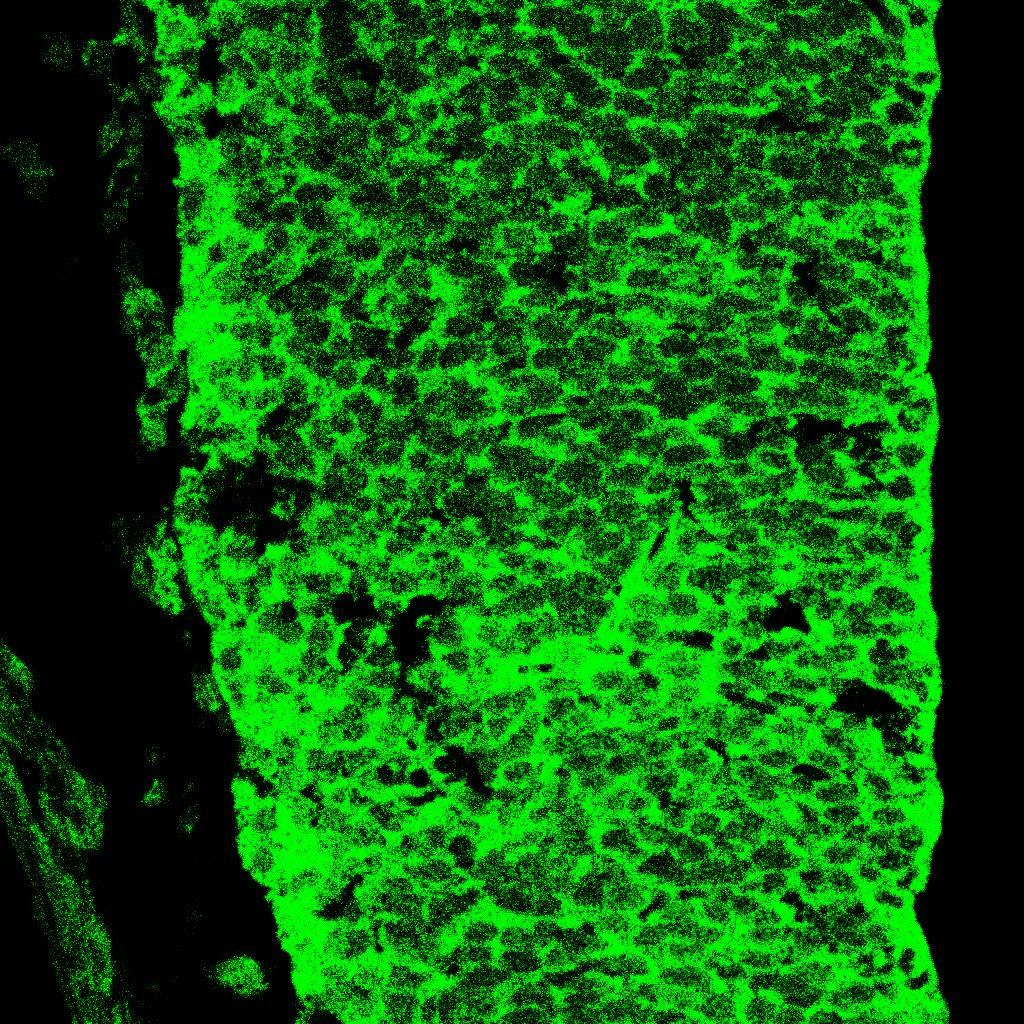

Supplement: Supplementary file 10 — Appendix Figures Source Data [file 44319_2026_768_MOESM10_ESM.zip › Appendix Figures/Appendix Figure S1/S1B/AKT.jpg]

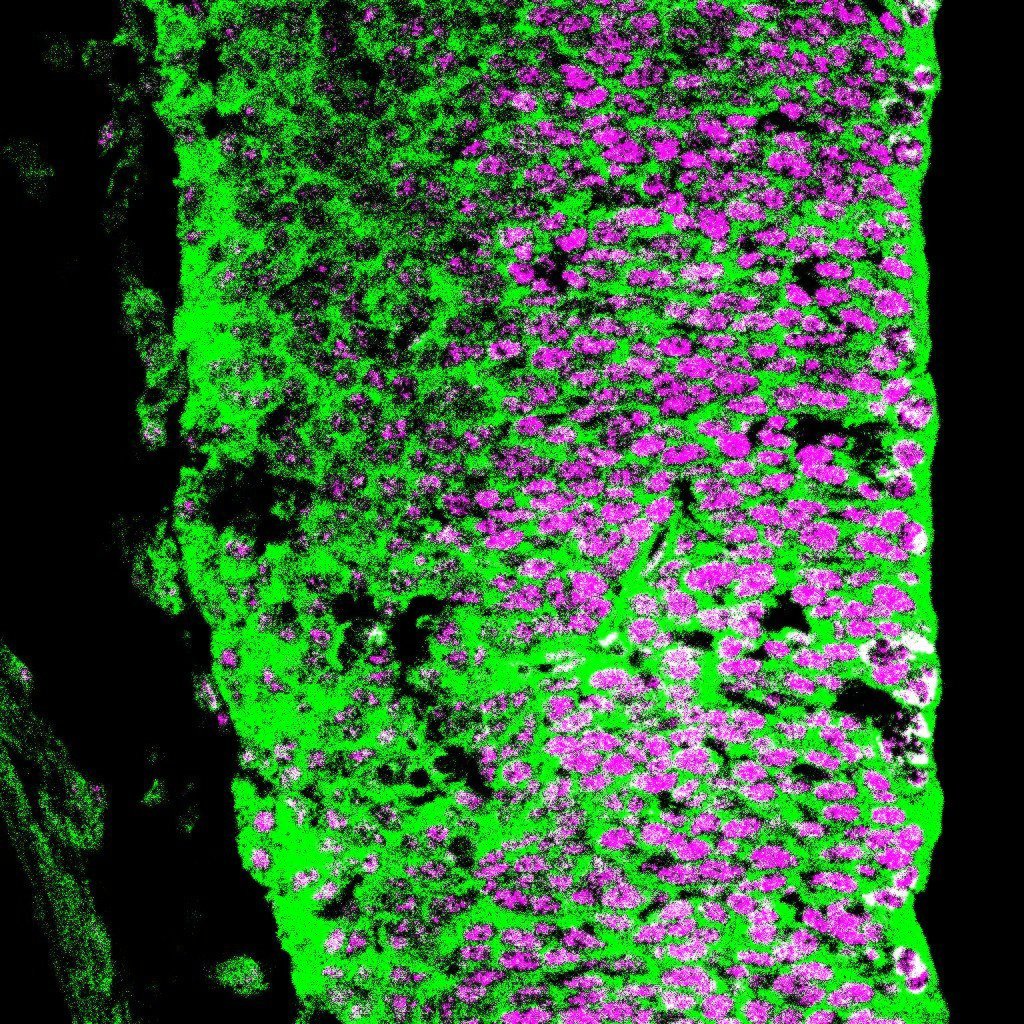

Supplement: Supplementary file 10 — Appendix Figures Source Data [file 44319_2026_768_MOESM10_ESM.zip › Appendix Figures/Appendix Figure S1/S1B/Merge.jpg]

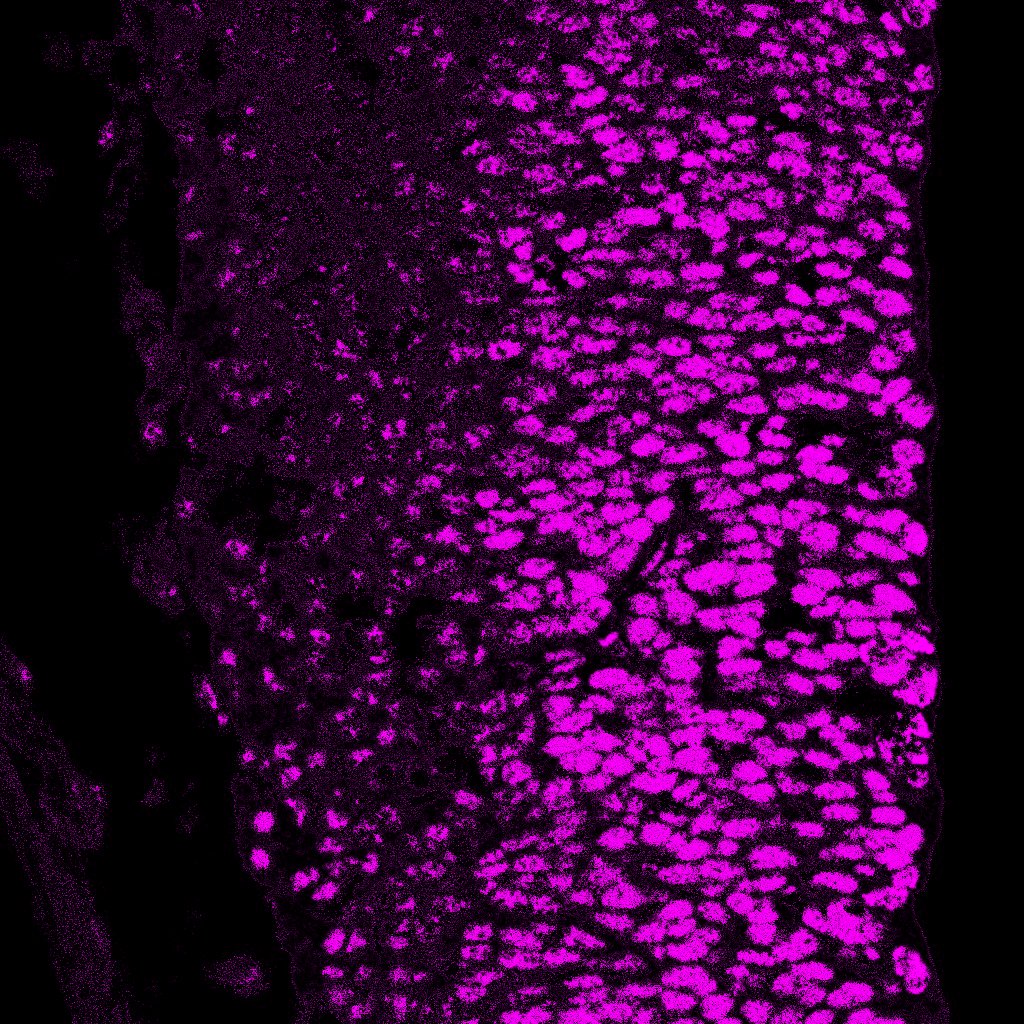

Supplement: Supplementary file 10 — Appendix Figures Source Data [file 44319_2026_768_MOESM10_ESM.zip › Appendix Figures/Appendix Figure S1/S1B/SOX2.jpg]

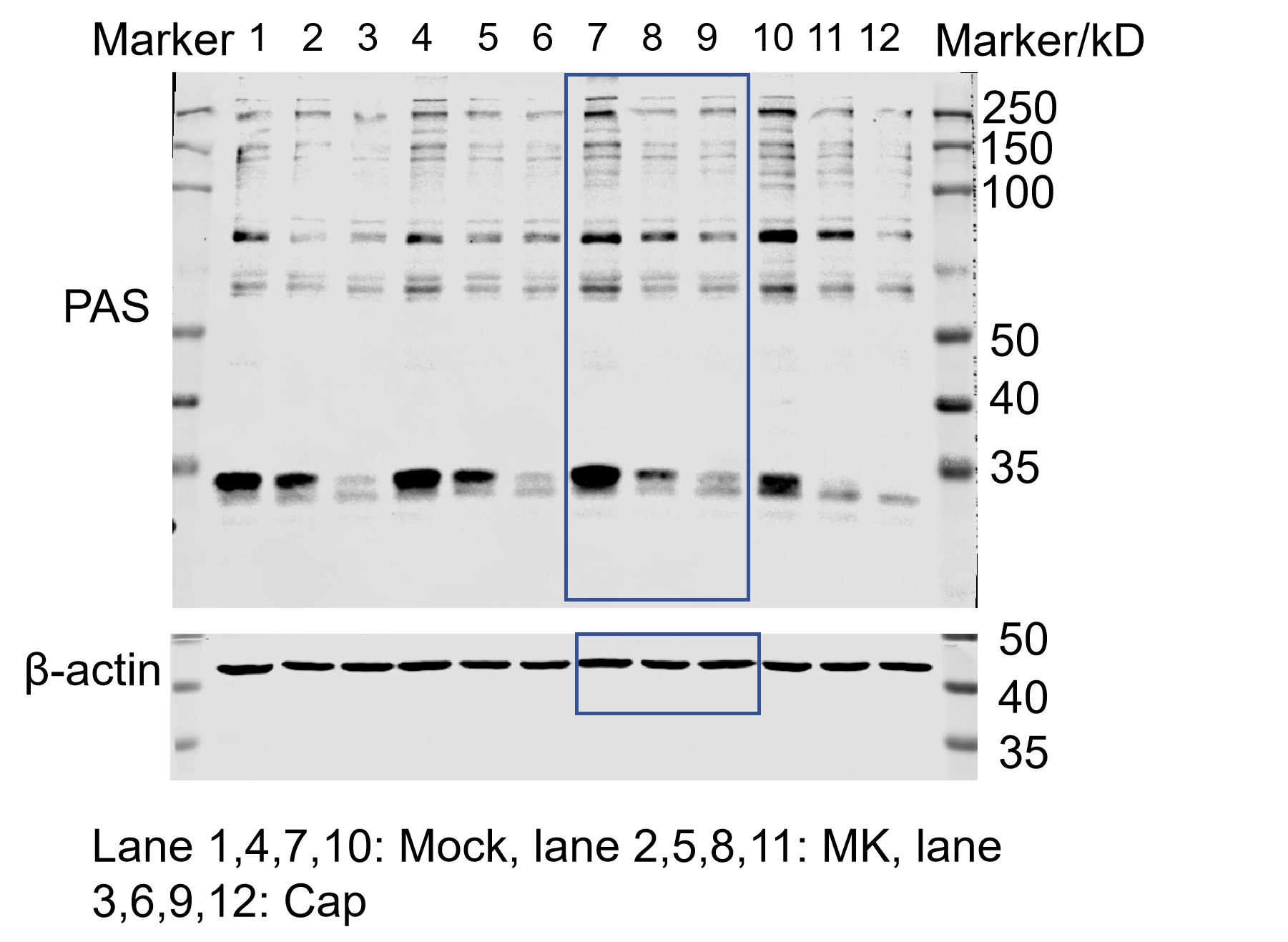

Supplement: Supplementary file 10 — Appendix Figures Source Data [file 44319_2026_768_MOESM10_ESM.zip › Appendix Figures/Appendix Figure S1/S1C/S1C.tif]

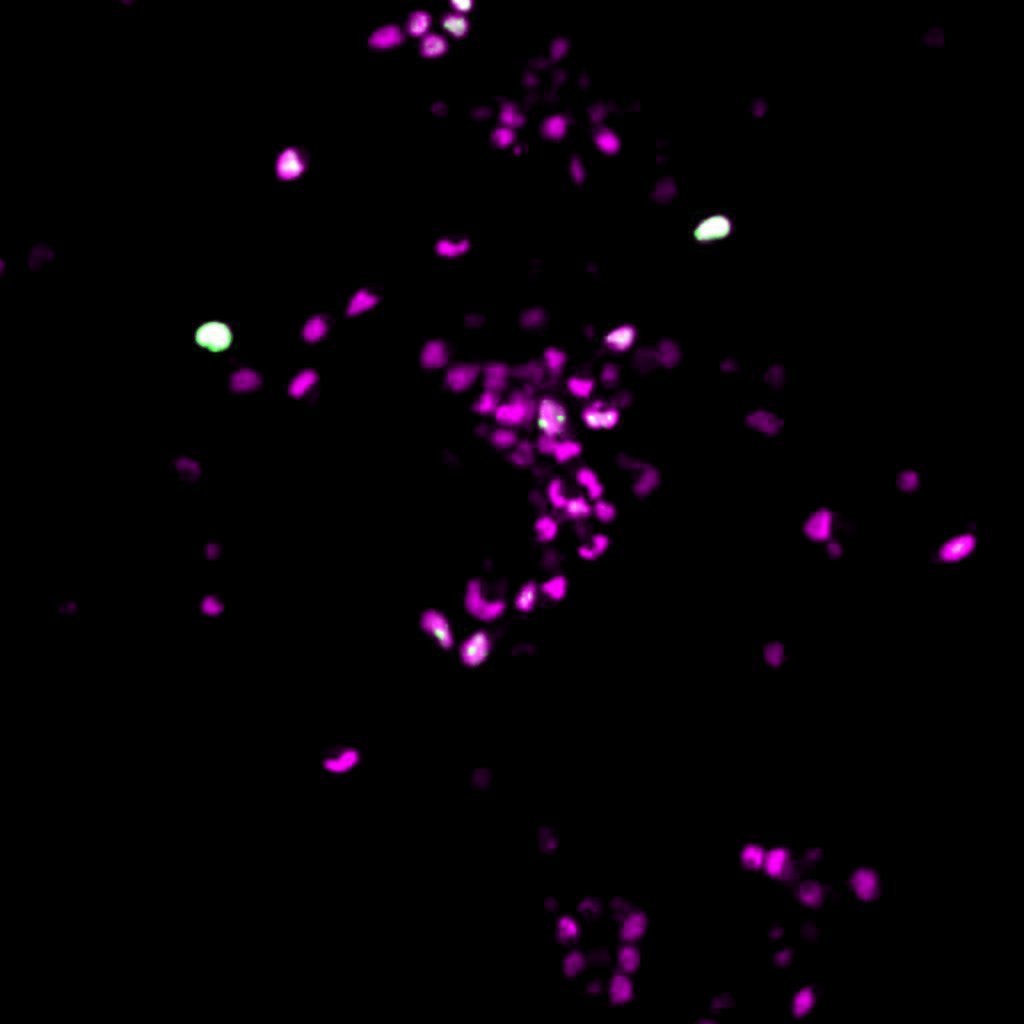

Supplement: Supplementary file 10 — Appendix Figures Source Data [file 44319_2026_768_MOESM10_ESM.zip › Appendix Figures/Appendix Figure S1/S1D/Cap.jpg]

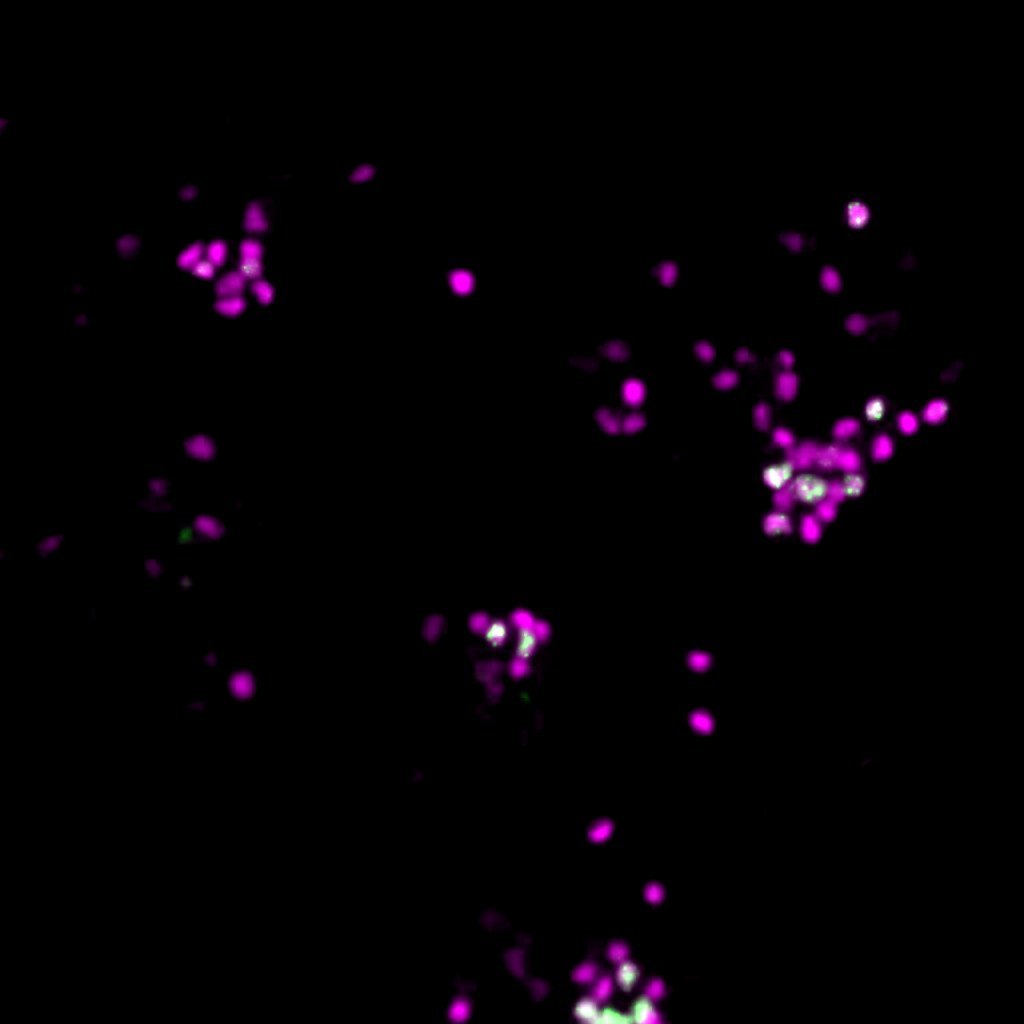

Supplement: Supplementary file 10 — Appendix Figures Source Data [file 44319_2026_768_MOESM10_ESM.zip › Appendix Figures/Appendix Figure S1/S1D/MK.jpg]

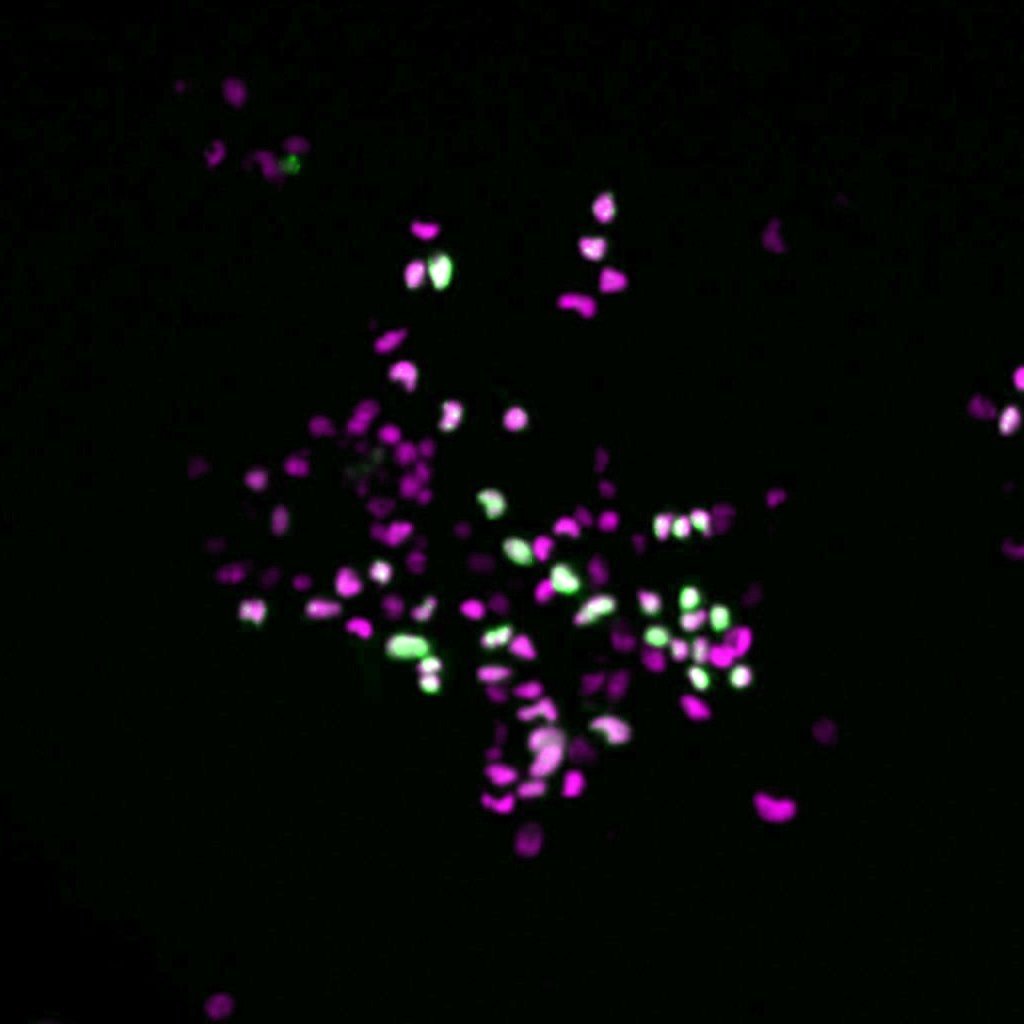

Supplement: Supplementary file 10 — Appendix Figures Source Data [file 44319_2026_768_MOESM10_ESM.zip › Appendix Figures/Appendix Figure S1/S1D/Mock.jpg]

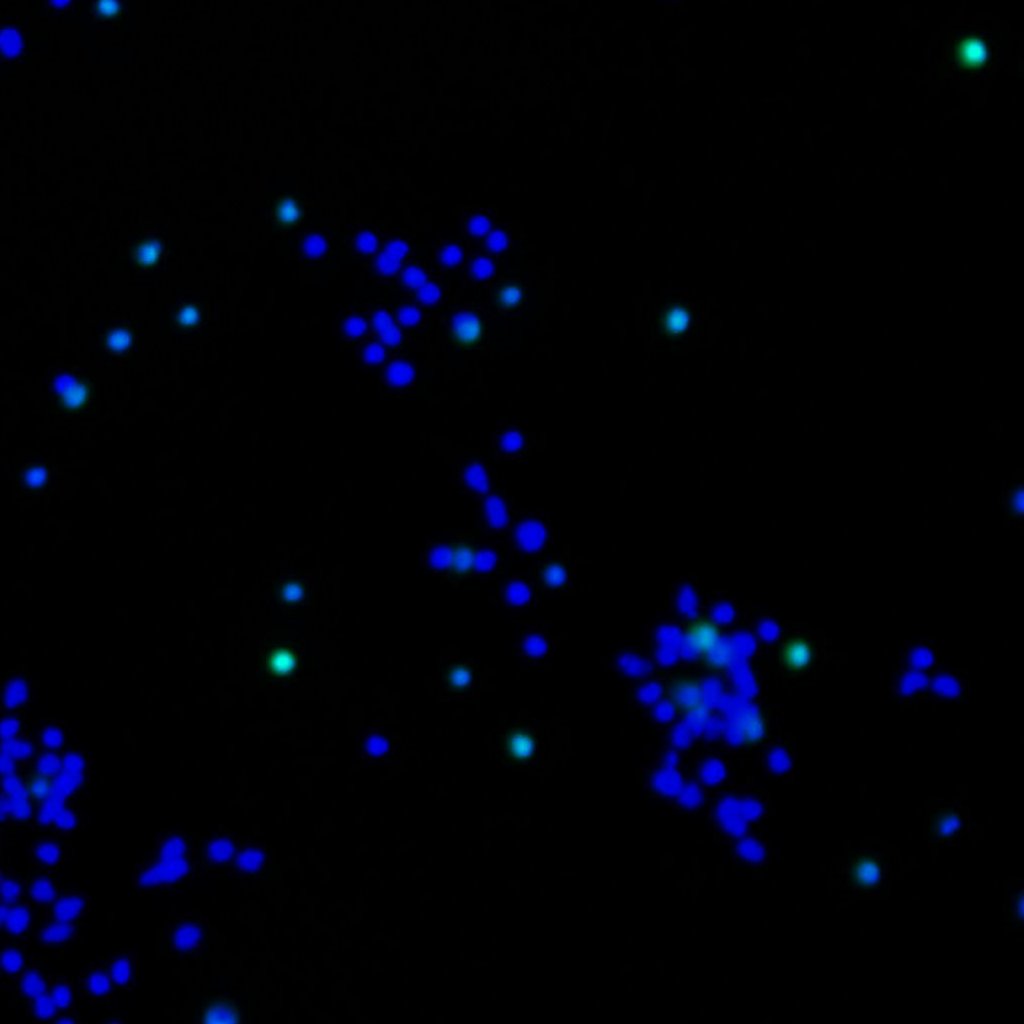

Supplement: Supplementary file 10 — Appendix Figures Source Data [file 44319_2026_768_MOESM10_ESM.zip › Appendix Figures/Appendix Figure S1/S1F/Cap.jpg]

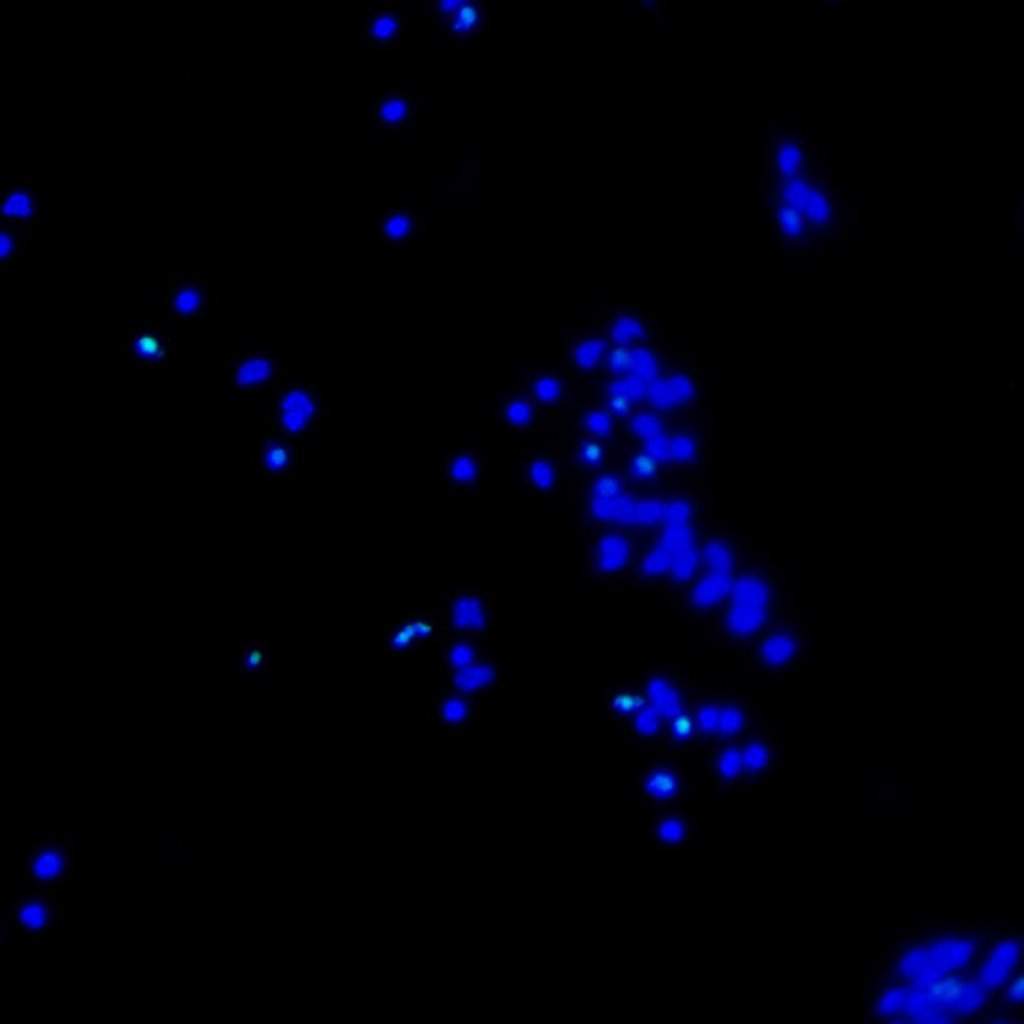

Supplement: Supplementary file 10 — Appendix Figures Source Data [file 44319_2026_768_MOESM10_ESM.zip › Appendix Figures/Appendix Figure S1/S1F/MK.jpg]

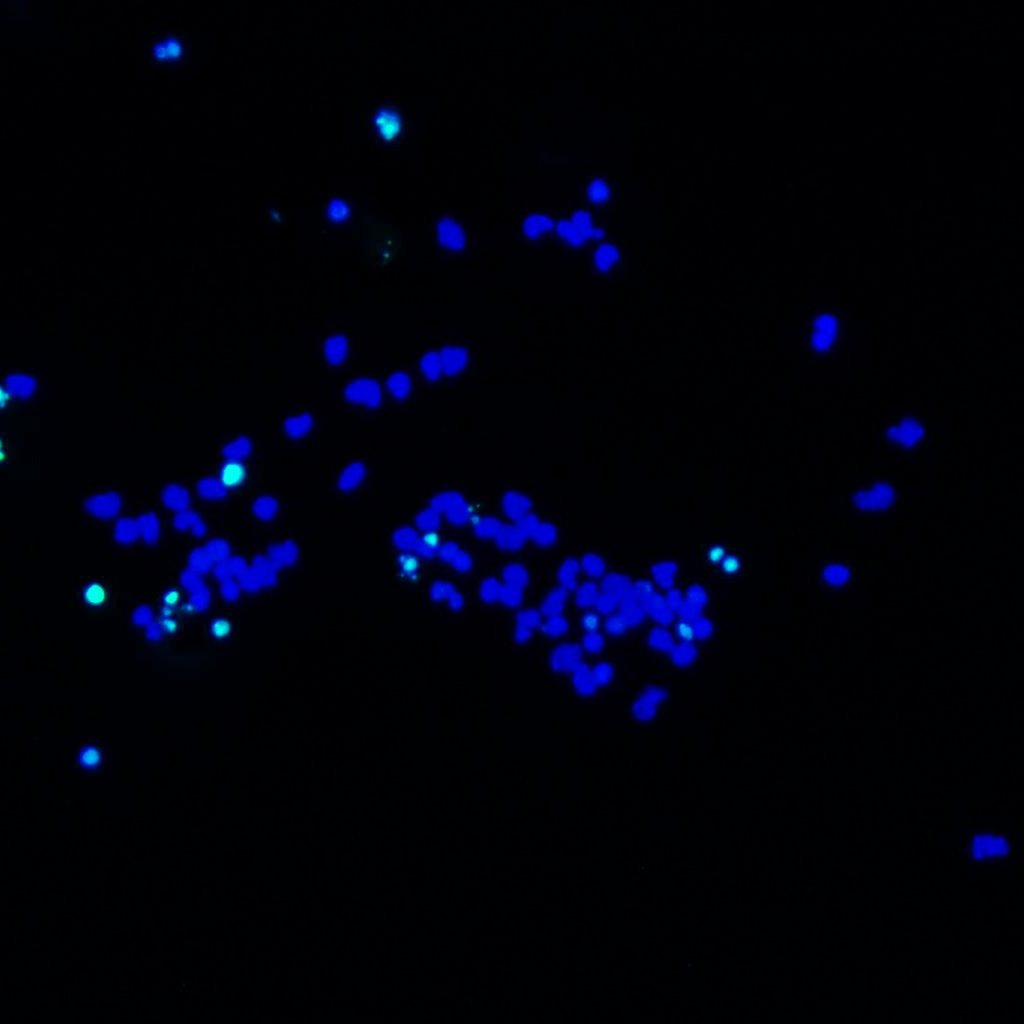

Supplement: Supplementary file 10 — Appendix Figures Source Data [file 44319_2026_768_MOESM10_ESM.zip › Appendix Figures/Appendix Figure S1/S1F/Mock.jpg]

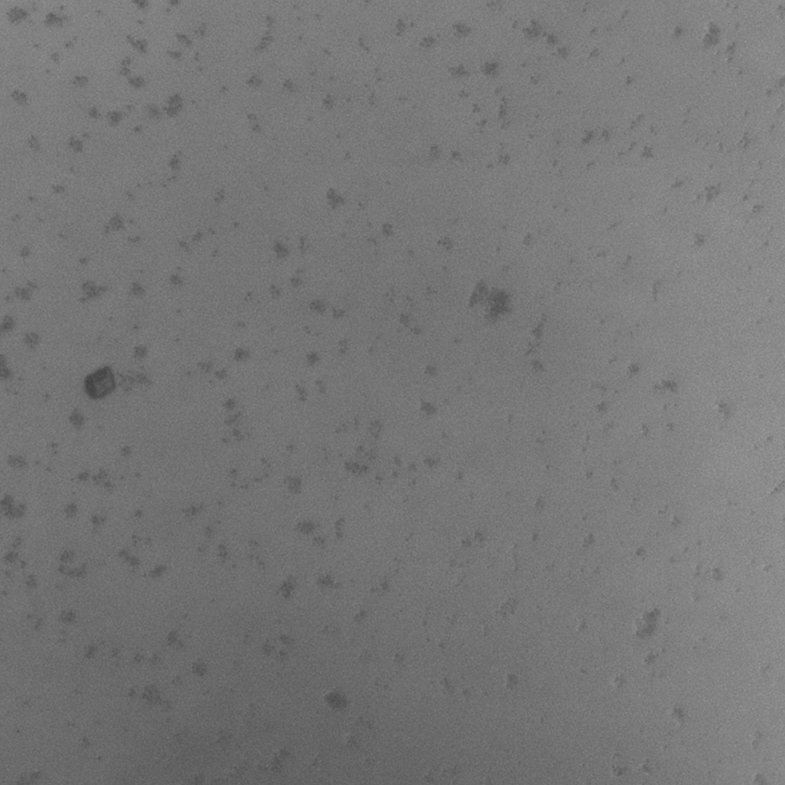

Supplement: Supplementary file 10 — Appendix Figures Source Data [file 44319_2026_768_MOESM10_ESM.zip › Appendix Figures/Appendix Figure S1/S1H/Blasticidin.jpg]
