# Supplementary figures and images for: CRL4AMBRA1 is a key mediator for AKT-dependent cell cycle control in neural progenitor cells (part 2 of 2)
Source: EMBO Rep. 2026 Apr 27;27(11):3099–119. doi: 10.1038/s44319-026-00768-7 (PMC13260899; doi:10.1038/s44319-026-00768-7)

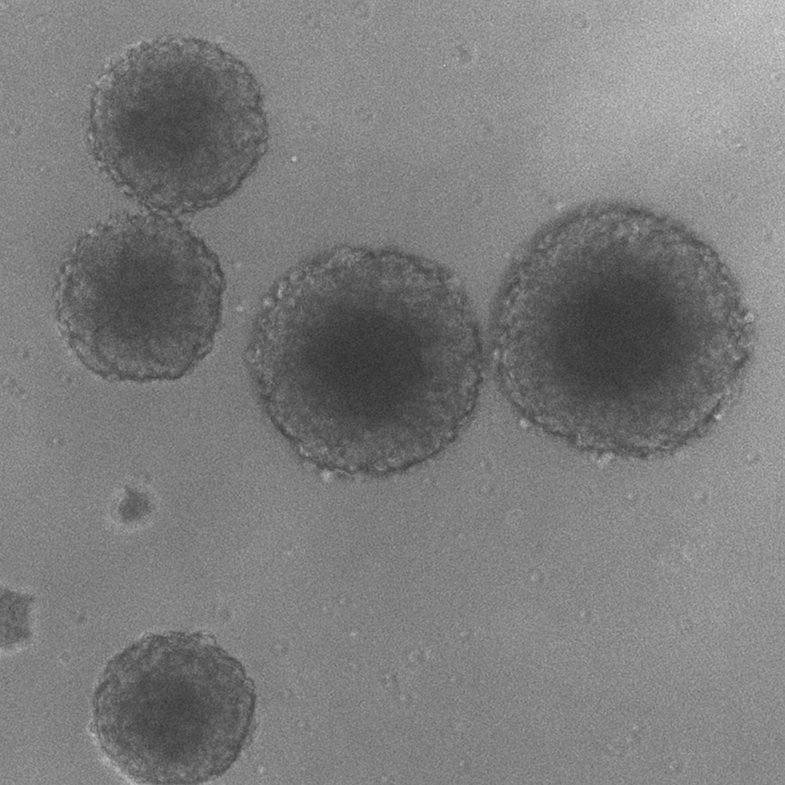

Supplement: Supplementary file 10 — Appendix Figures Source Data [file 44319_2026_768_MOESM10_ESM.zip › Appendix Figures/Appendix Figure S1/S1H/Bright field.jpg]

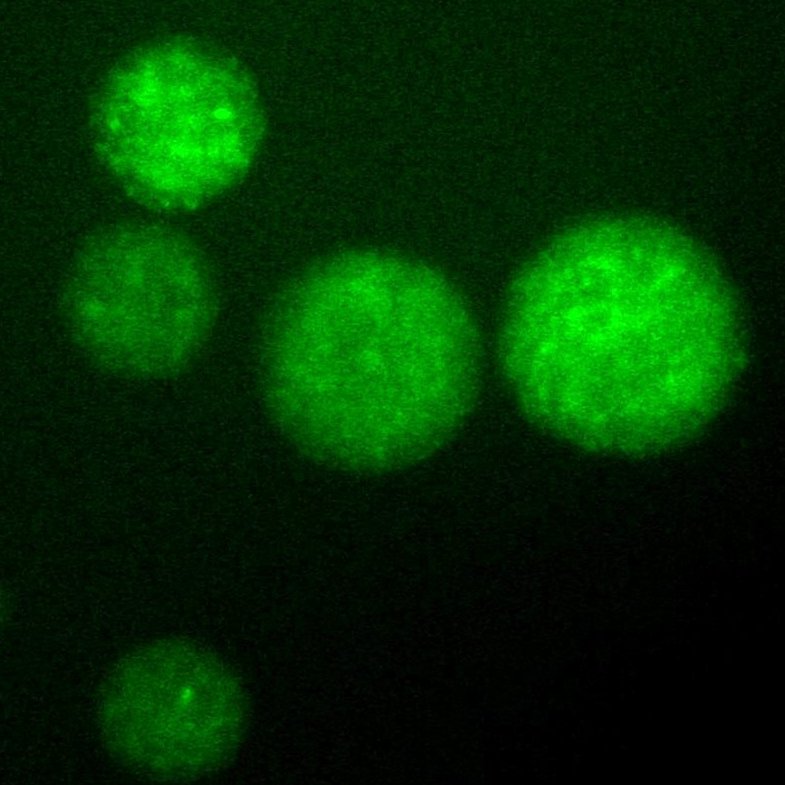

Supplement: Supplementary file 10 — Appendix Figures Source Data [file 44319_2026_768_MOESM10_ESM.zip › Appendix Figures/Appendix Figure S1/S1H/GFP.jpg]

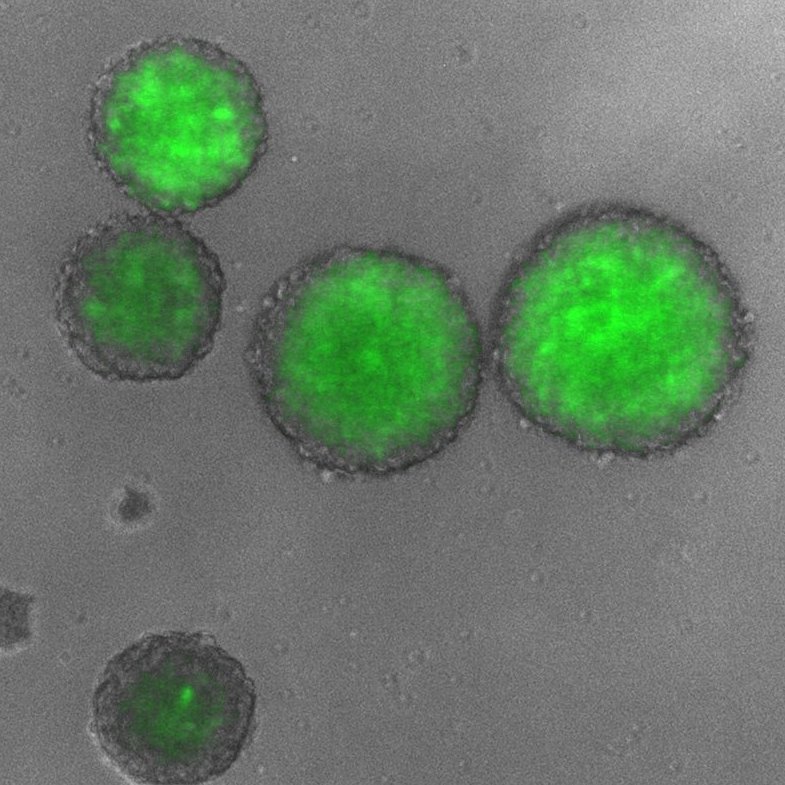

Supplement: Supplementary file 10 — Appendix Figures Source Data [file 44319_2026_768_MOESM10_ESM.zip › Appendix Figures/Appendix Figure S1/S1H/Merge.jpg]

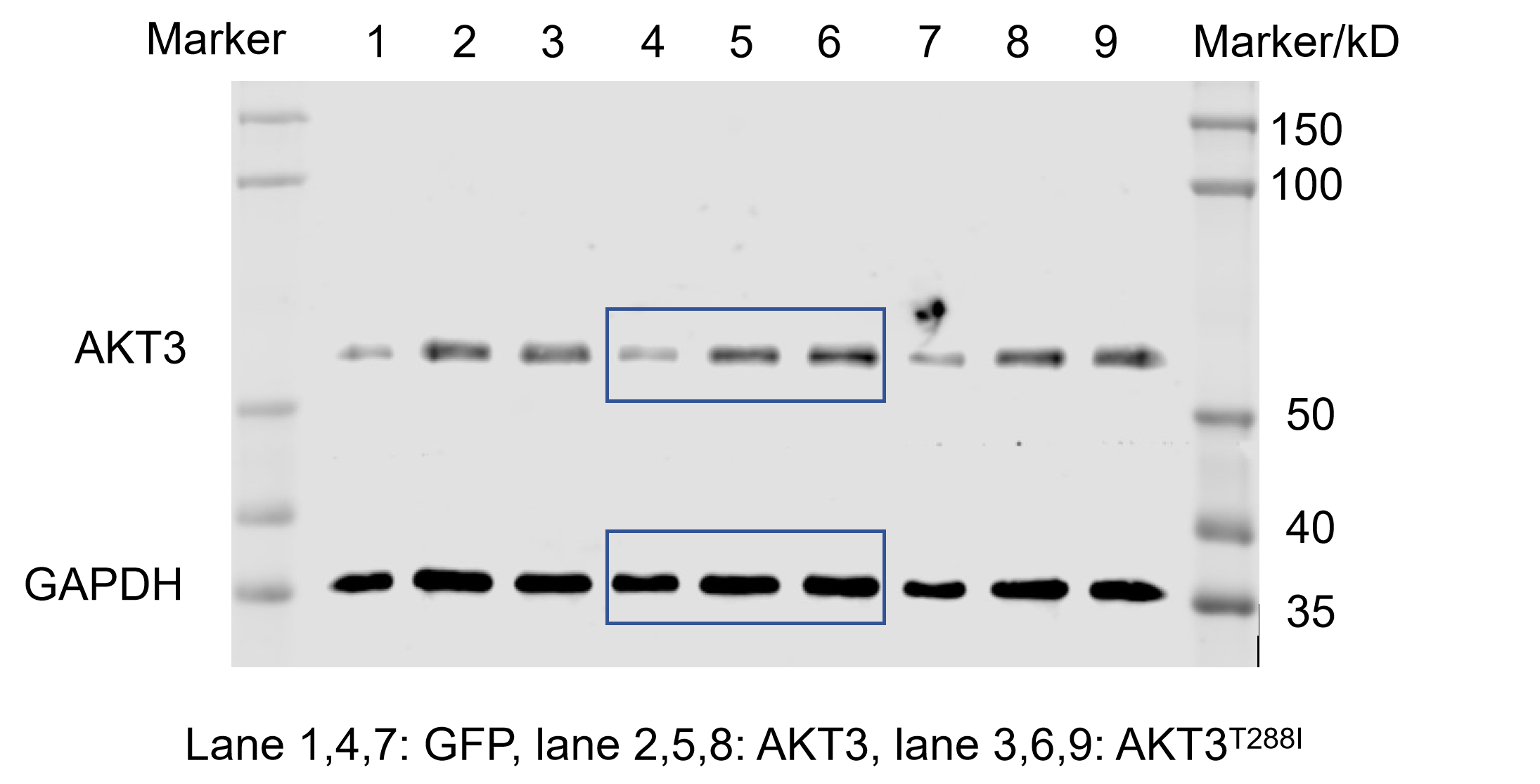

Supplement: Supplementary file 10 — Appendix Figures Source Data [file 44319_2026_768_MOESM10_ESM.zip › Appendix Figures/Appendix Figure S1/S1I/S1I.tif]

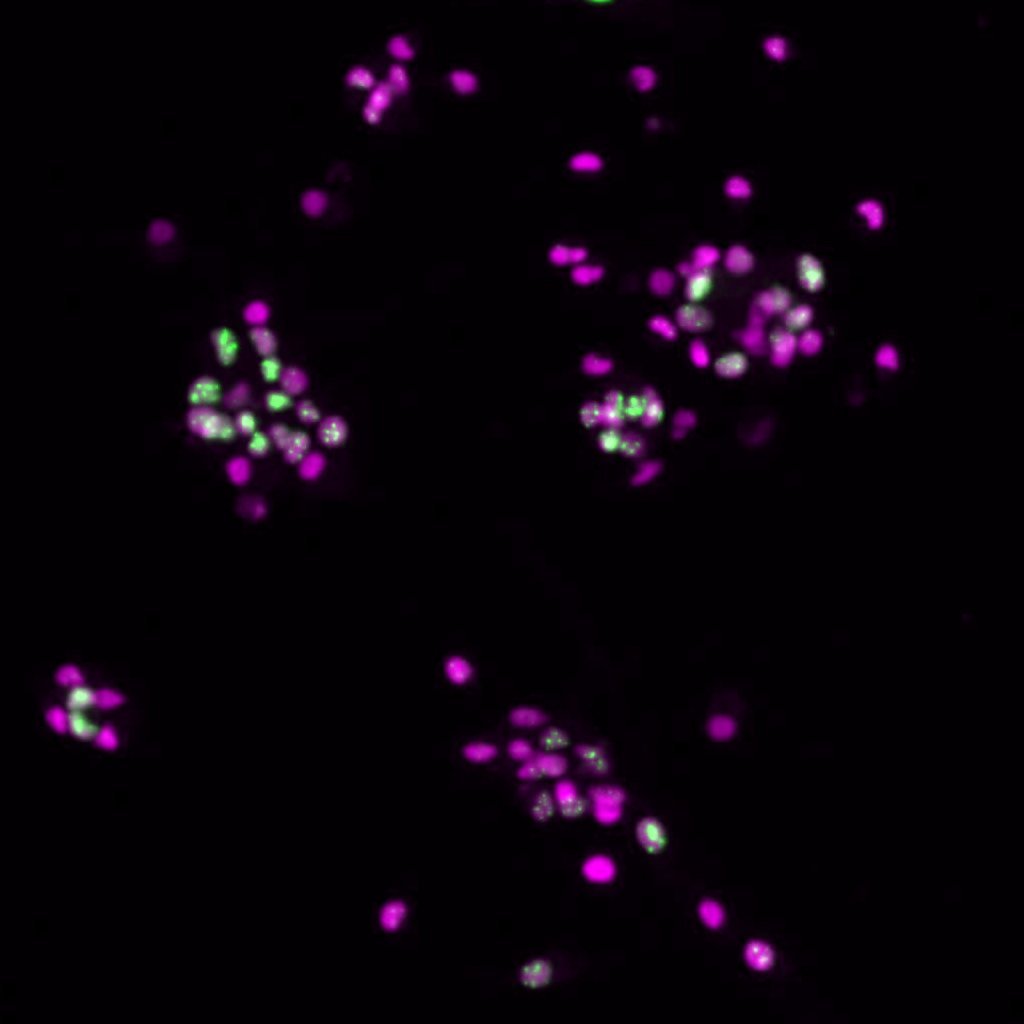

Supplement: Supplementary file 10 — Appendix Figures Source Data [file 44319_2026_768_MOESM10_ESM.zip › Appendix Figures/Appendix Figure S1/S1K/AKT3.jpg]

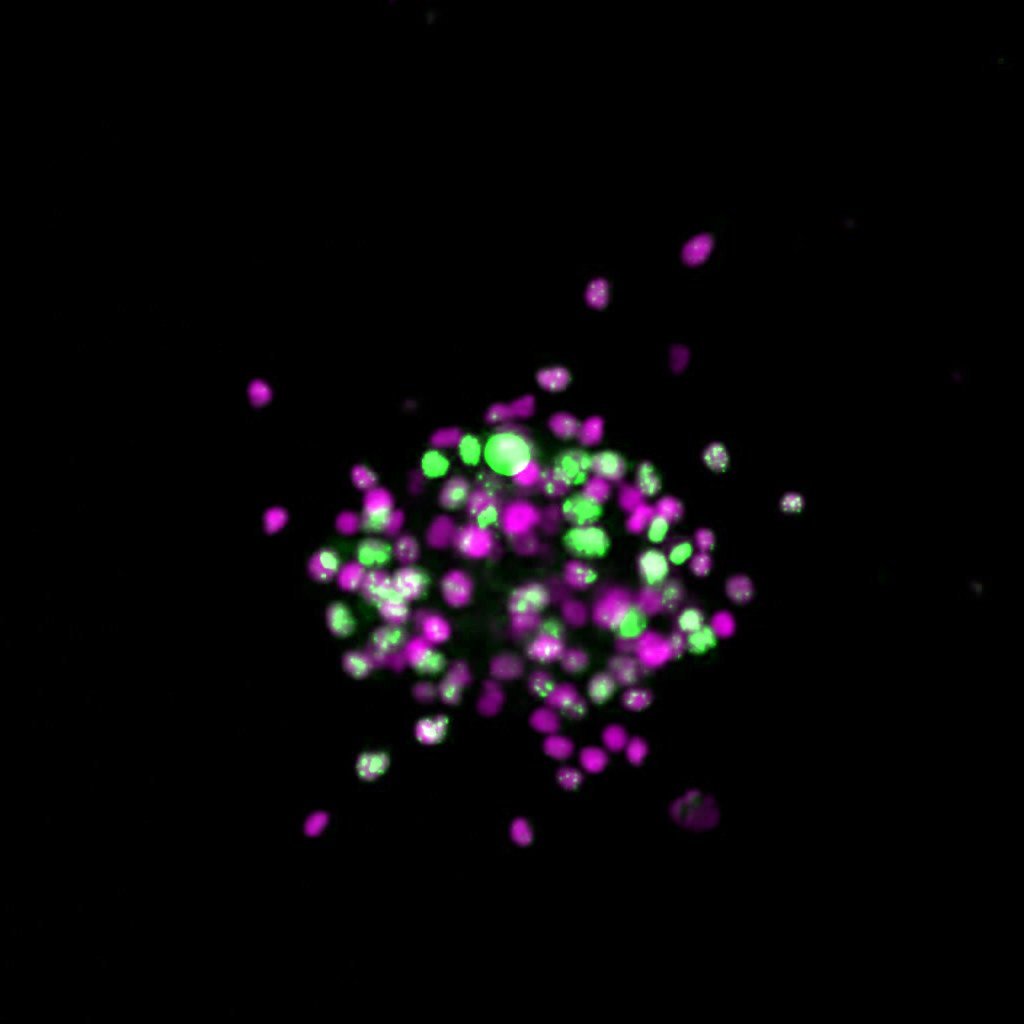

Supplement: Supplementary file 10 — Appendix Figures Source Data [file 44319_2026_768_MOESM10_ESM.zip › Appendix Figures/Appendix Figure S1/S1K/T288I.jpg]

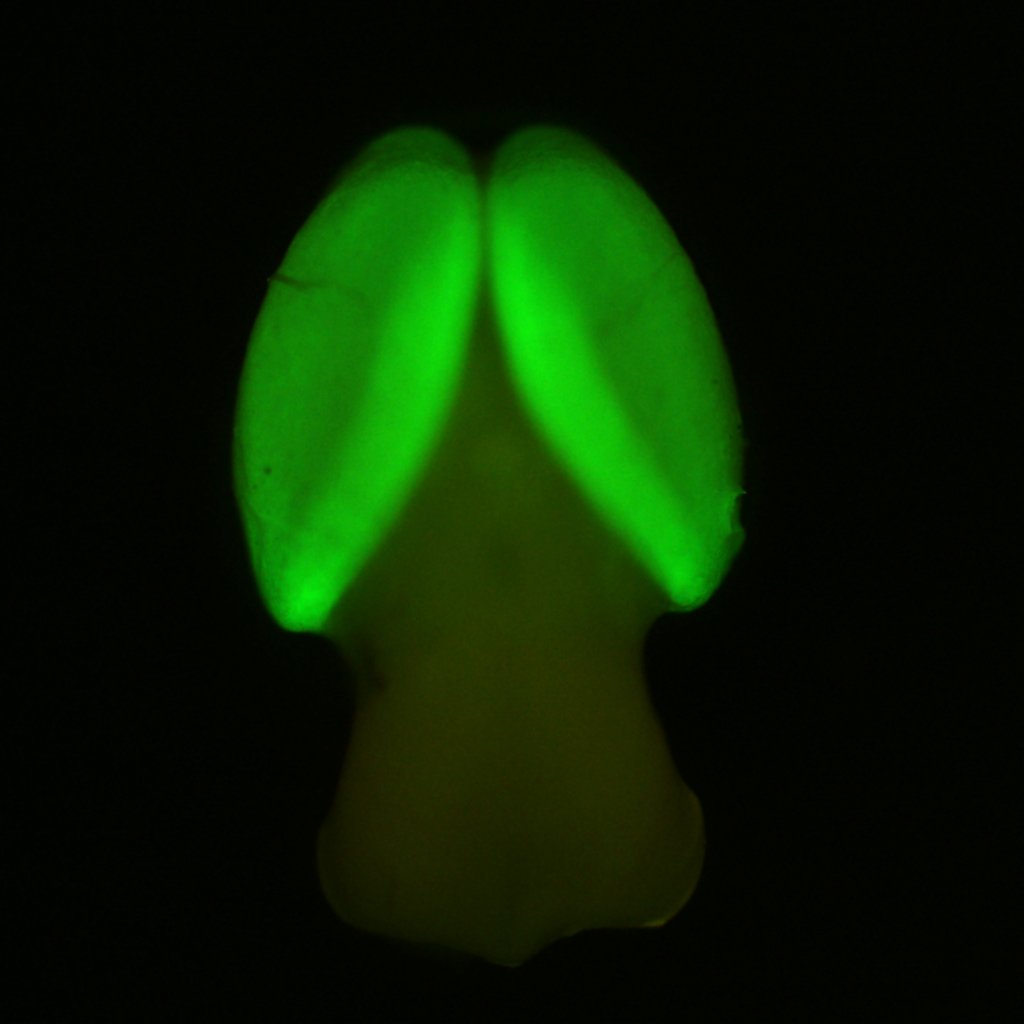

Supplement: Supplementary file 10 — Appendix Figures Source Data [file 44319_2026_768_MOESM10_ESM.zip › Appendix Figures/Appendix Figure S2/S2B/mtmg-emx1-cre.jpg]

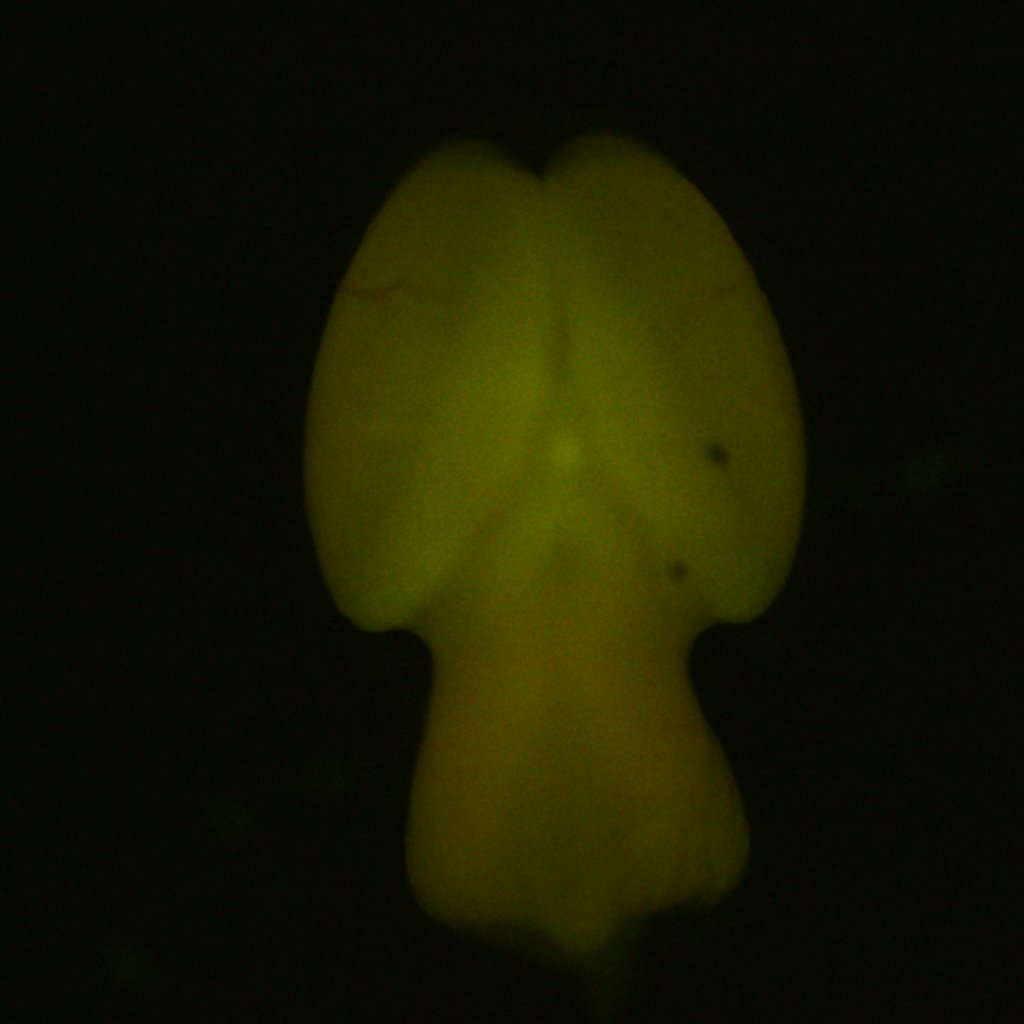

Supplement: Supplementary file 10 — Appendix Figures Source Data [file 44319_2026_768_MOESM10_ESM.zip › Appendix Figures/Appendix Figure S2/S2B/mtmg.jpg]

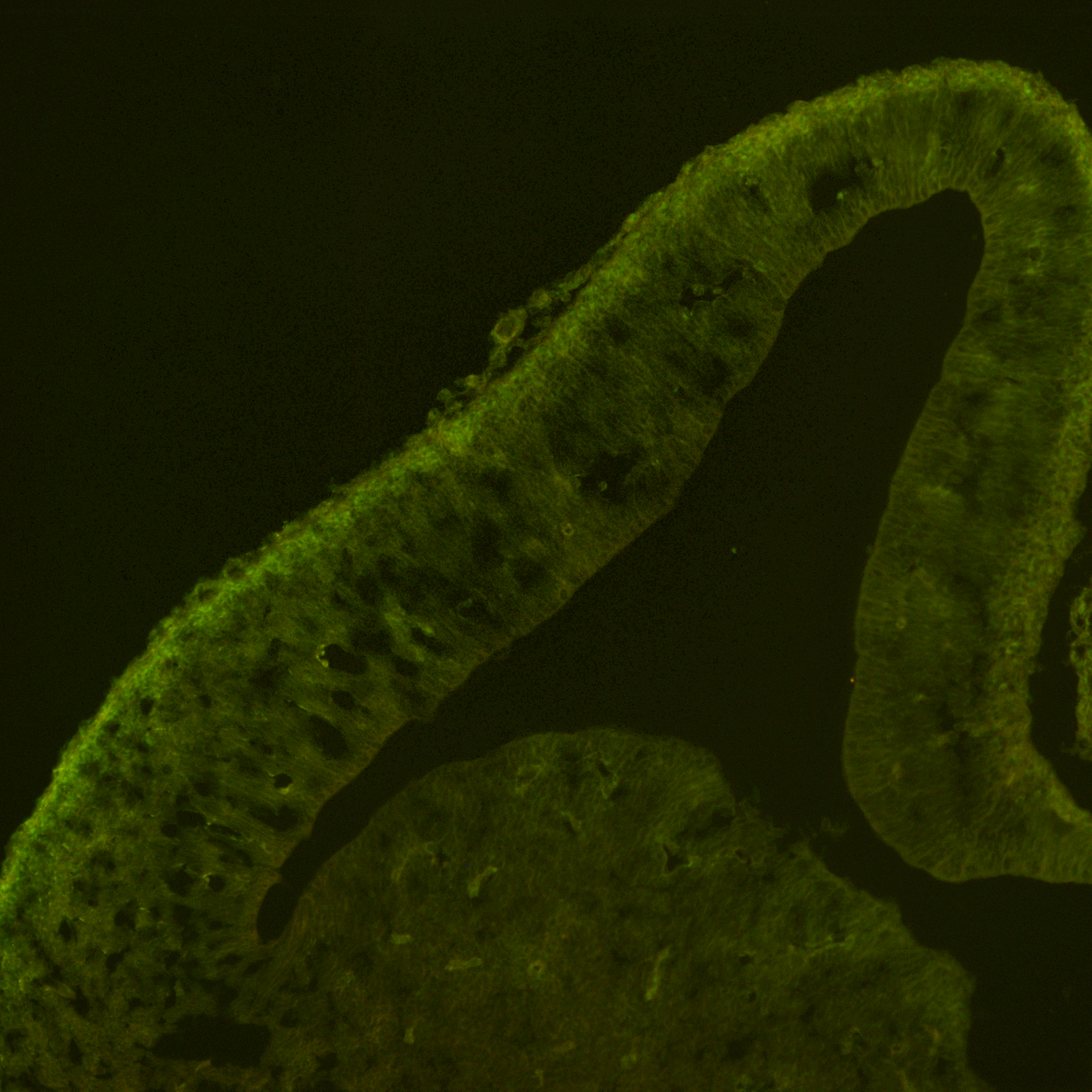

Supplement: Supplementary file 10 — Appendix Figures Source Data [file 44319_2026_768_MOESM10_ESM.zip › Appendix Figures/Appendix Figure S2/S2C/mtmg.tif]

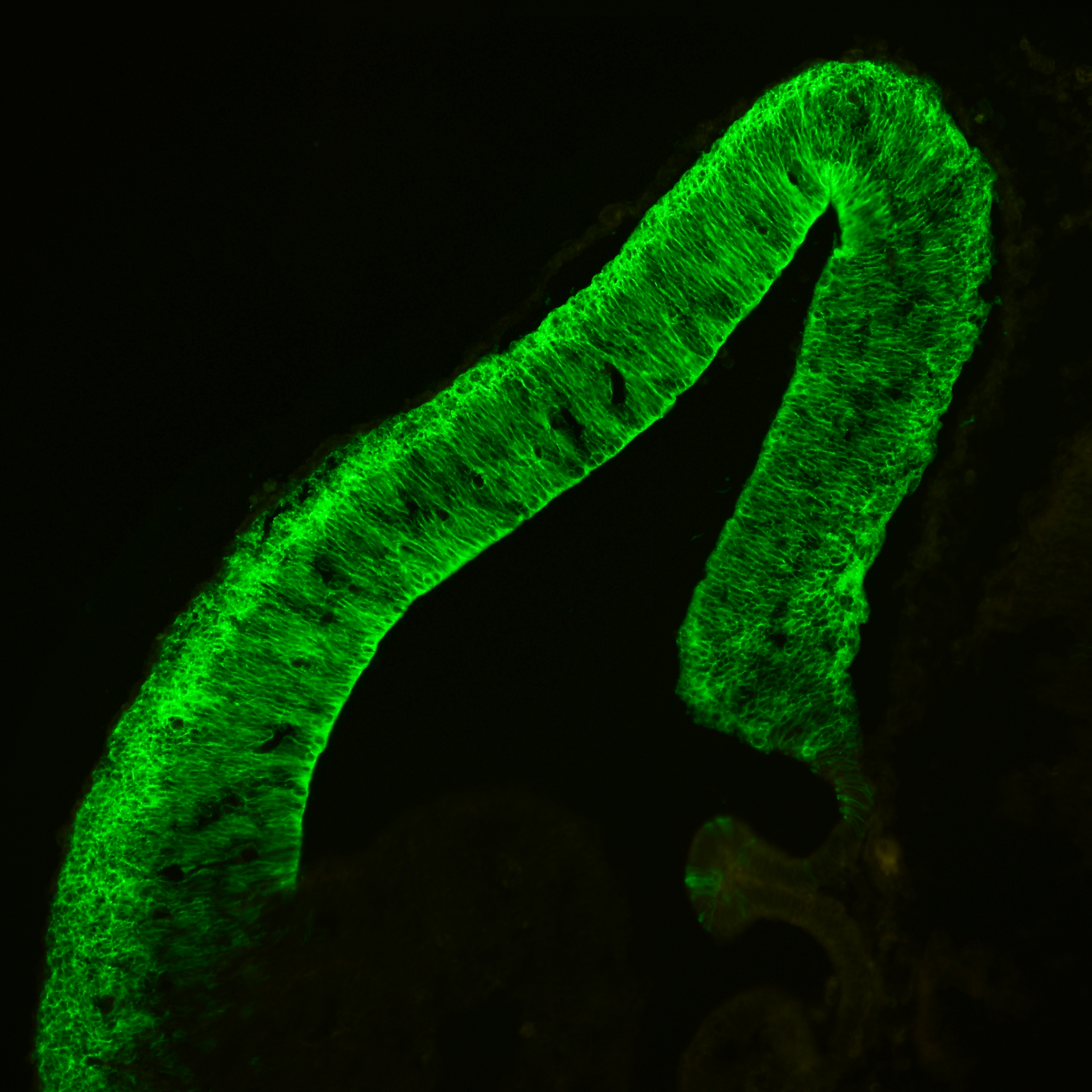

Supplement: Supplementary file 10 — Appendix Figures Source Data [file 44319_2026_768_MOESM10_ESM.zip › Appendix Figures/Appendix Figure S2/S2C/mtmg;emx1-cre.tif]

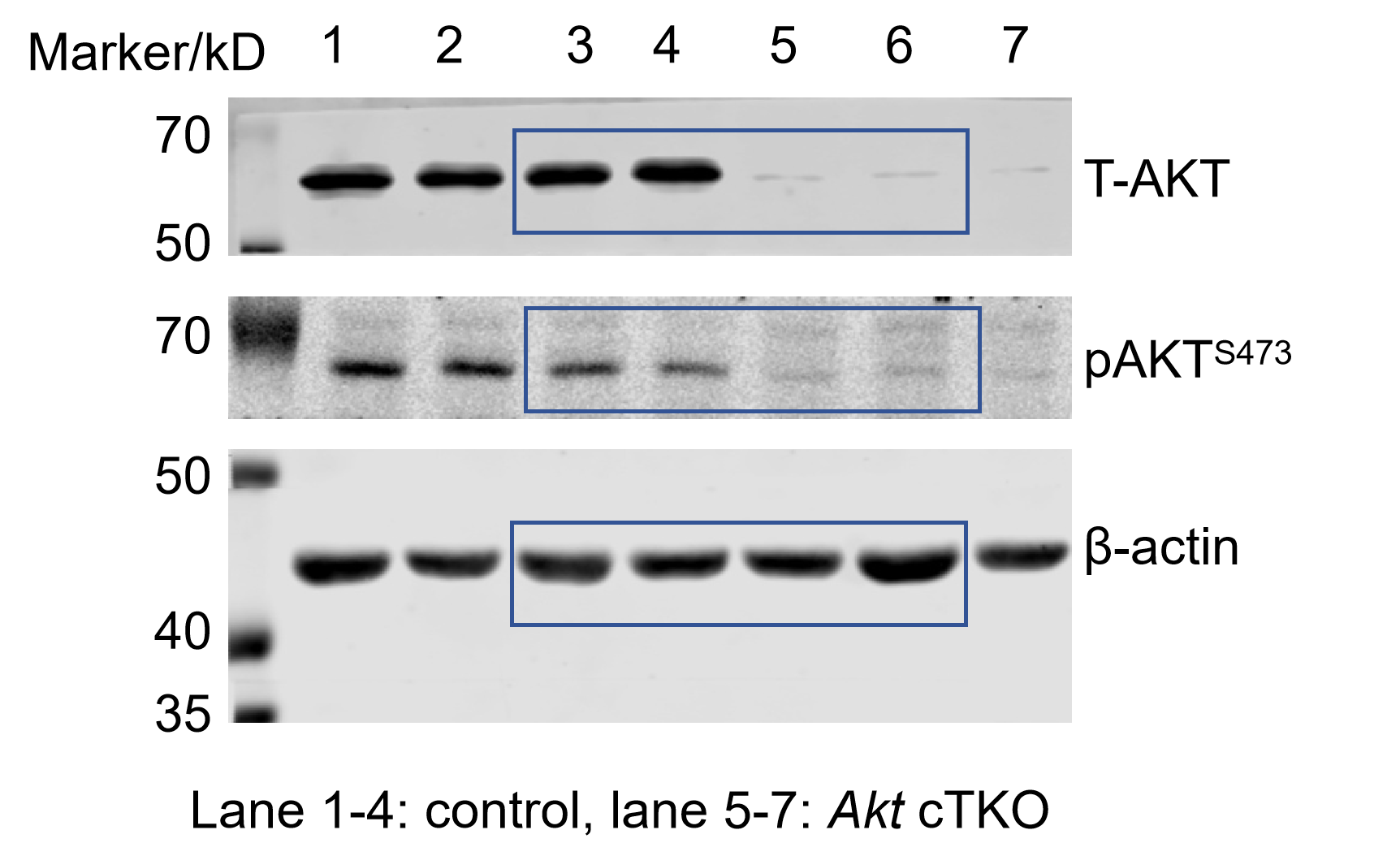

Supplement: Supplementary file 10 — Appendix Figures Source Data [file 44319_2026_768_MOESM10_ESM.zip › Appendix Figures/Appendix Figure S2/S2E/S2E.tif]

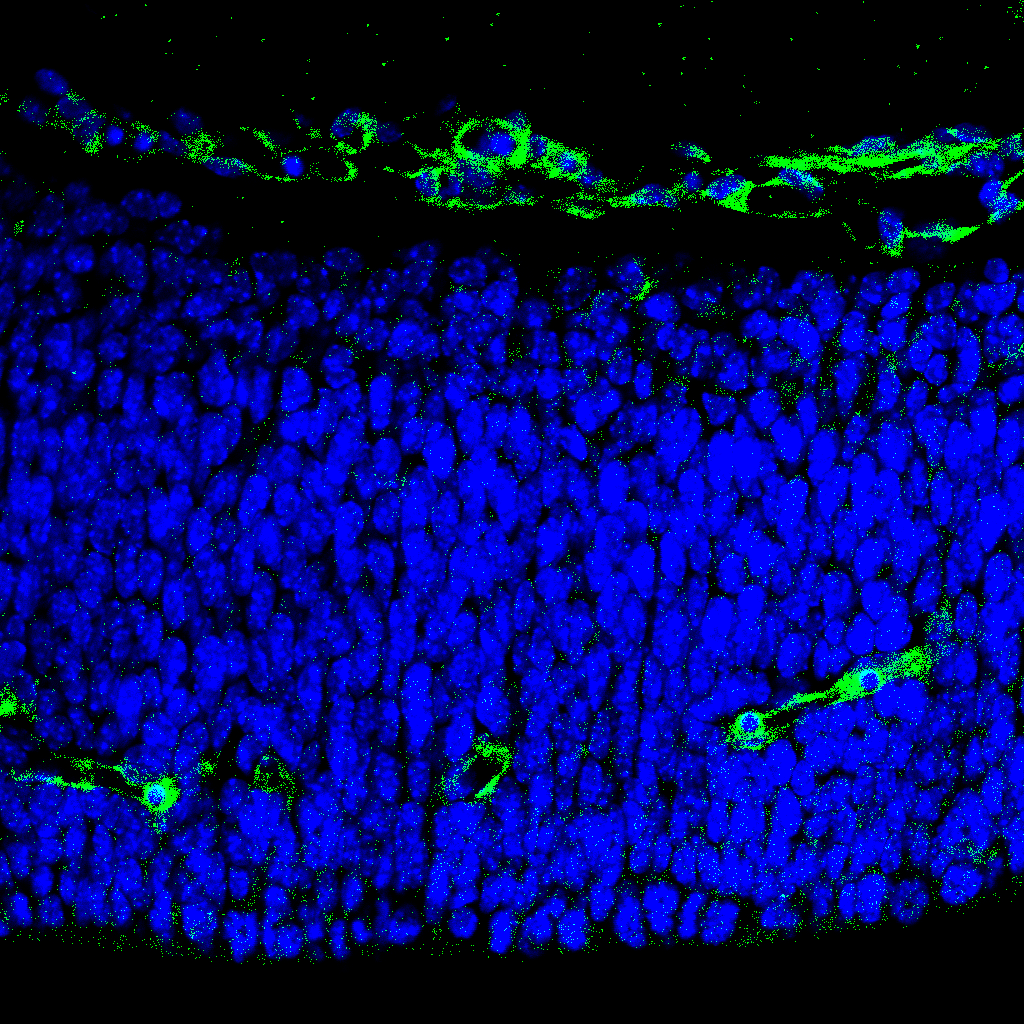

Supplement: Supplementary file 10 — Appendix Figures Source Data [file 44319_2026_768_MOESM10_ESM.zip › Appendix Figures/Appendix Figure S2/S2G/ctko.tif]

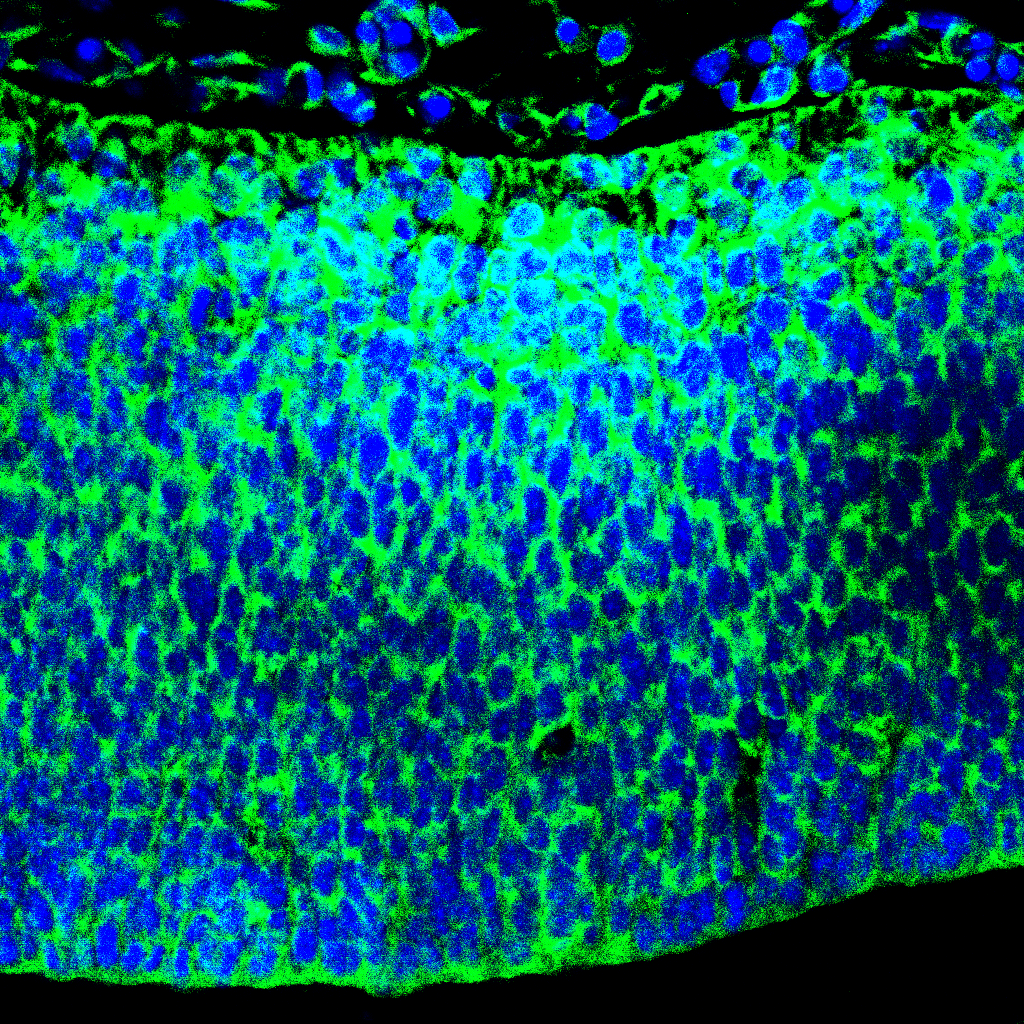

Supplement: Supplementary file 10 — Appendix Figures Source Data [file 44319_2026_768_MOESM10_ESM.zip › Appendix Figures/Appendix Figure S2/S2G/ctrl.tif]

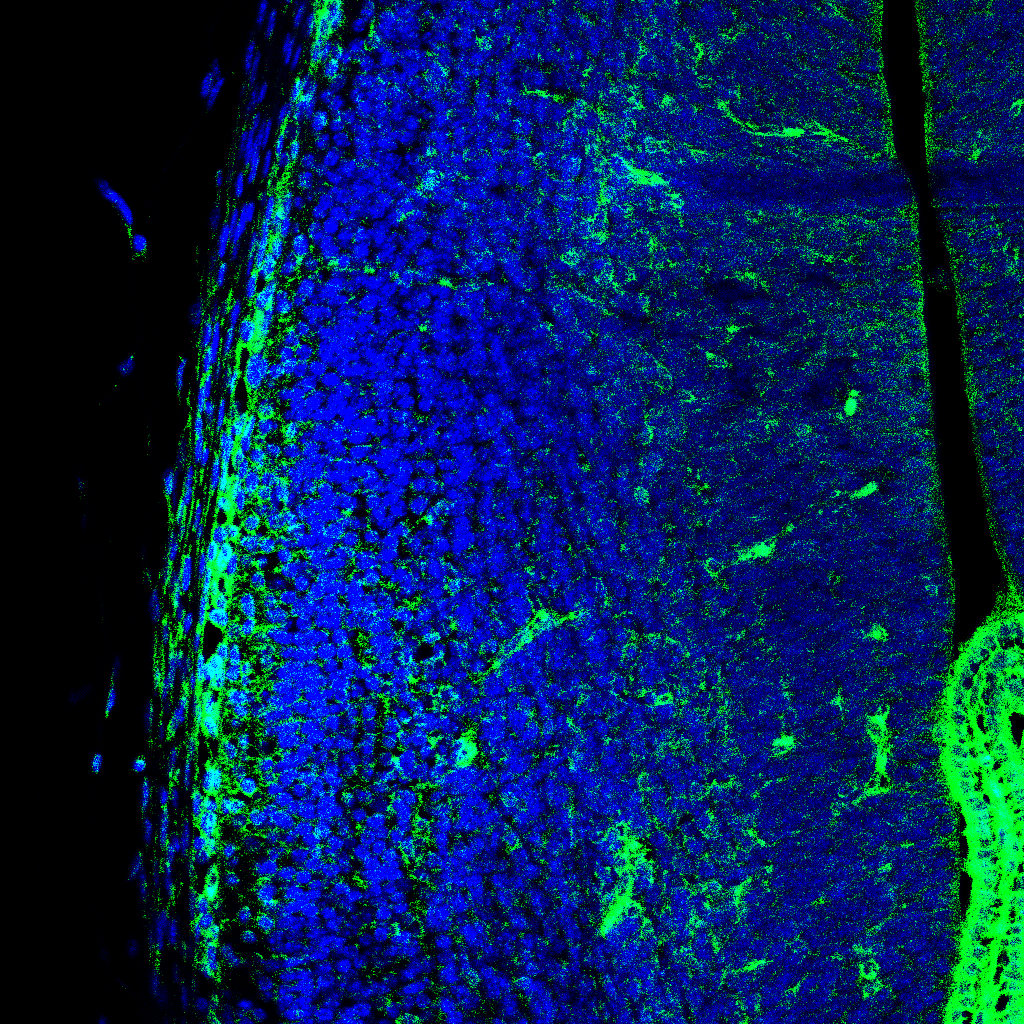

Supplement: Supplementary file 10 — Appendix Figures Source Data [file 44319_2026_768_MOESM10_ESM.zip › Appendix Figures/Appendix Figure S2/S2H/ctko.tif]

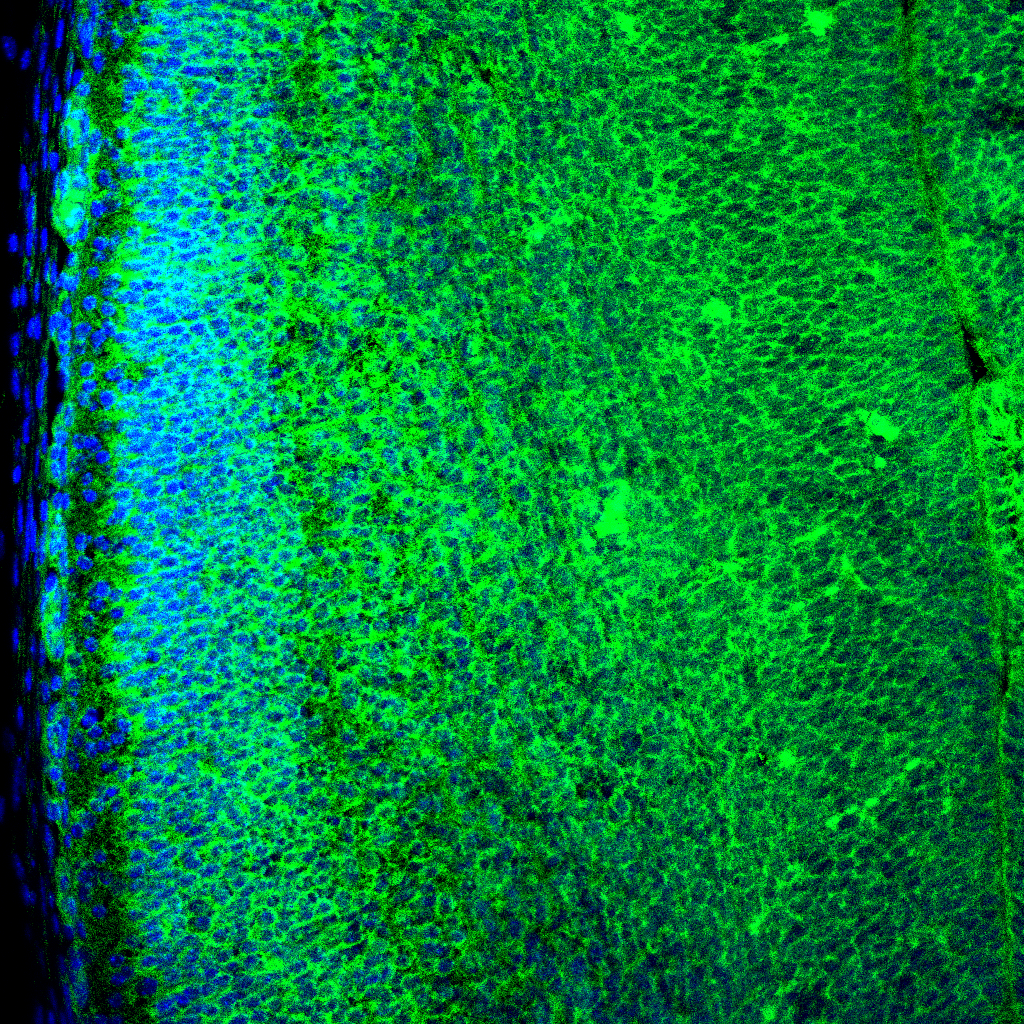

Supplement: Supplementary file 10 — Appendix Figures Source Data [file 44319_2026_768_MOESM10_ESM.zip › Appendix Figures/Appendix Figure S2/S2H/ctrl.tif]

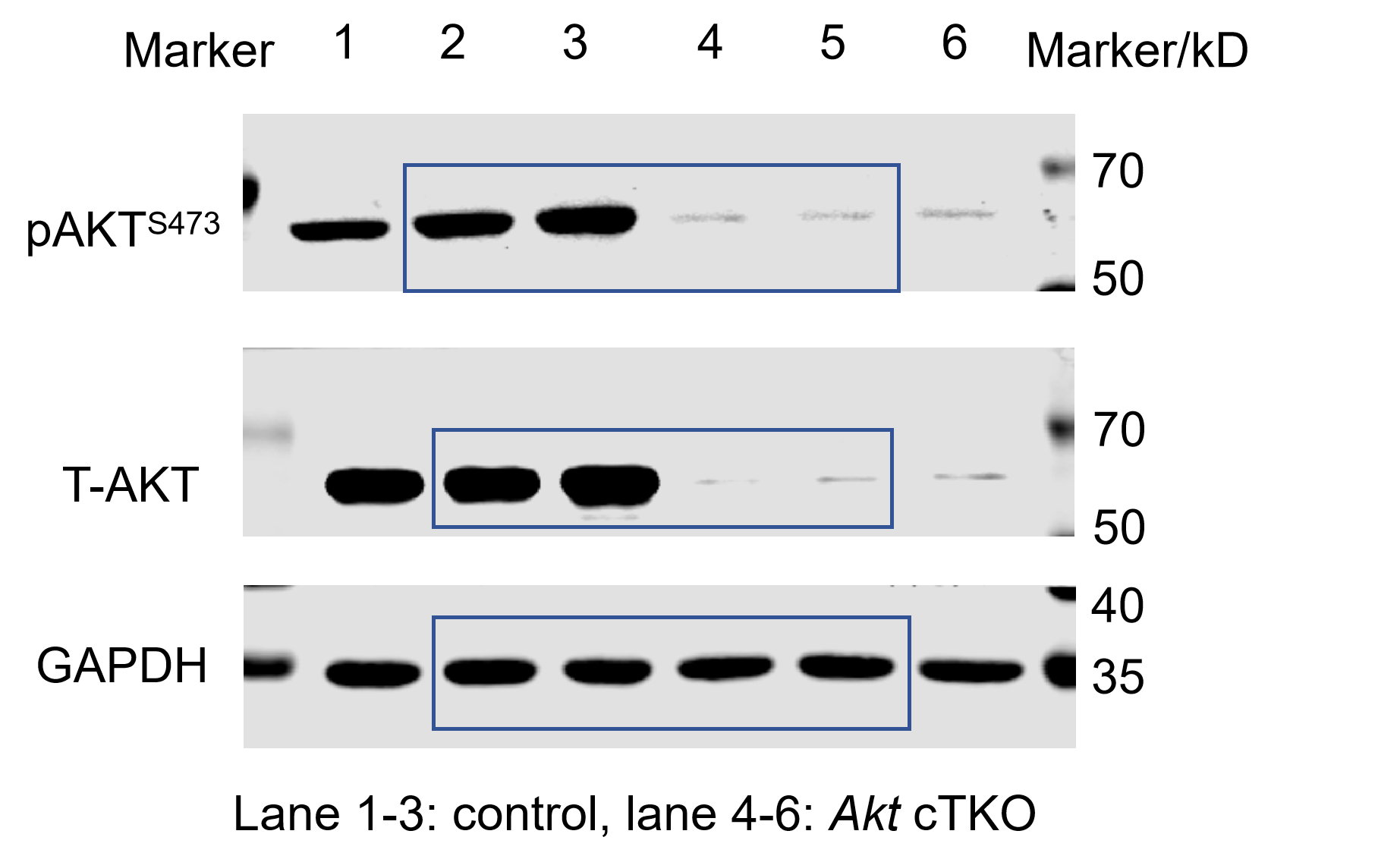

Supplement: Supplementary file 10 — Appendix Figures Source Data [file 44319_2026_768_MOESM10_ESM.zip › Appendix Figures/Appendix Figure S2/S2I/S2I.tif]

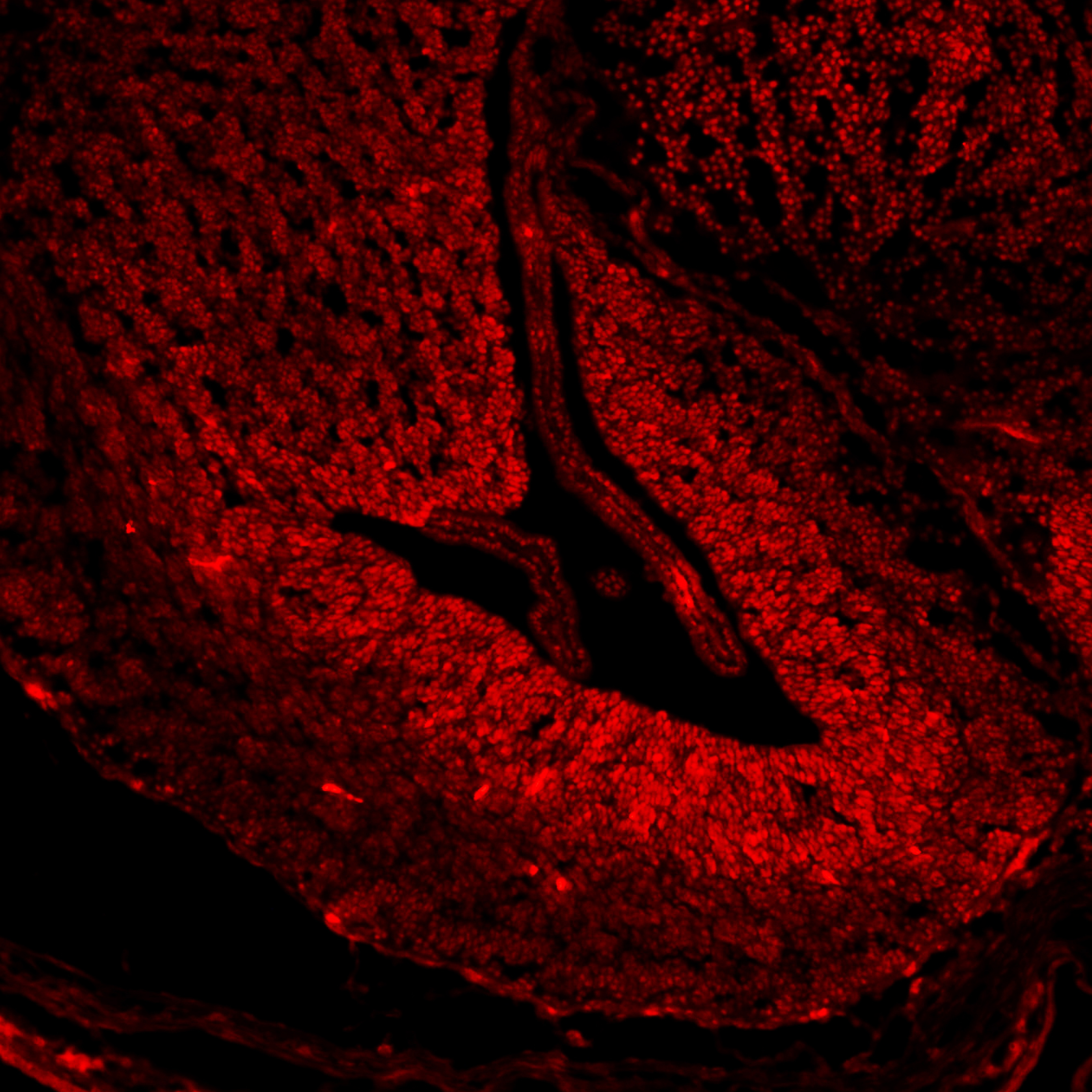

Supplement: Supplementary file 10 — Appendix Figures Source Data [file 44319_2026_768_MOESM10_ESM.zip › Appendix Figures/Appendix Figure S2/S2K/ctko.jpg]

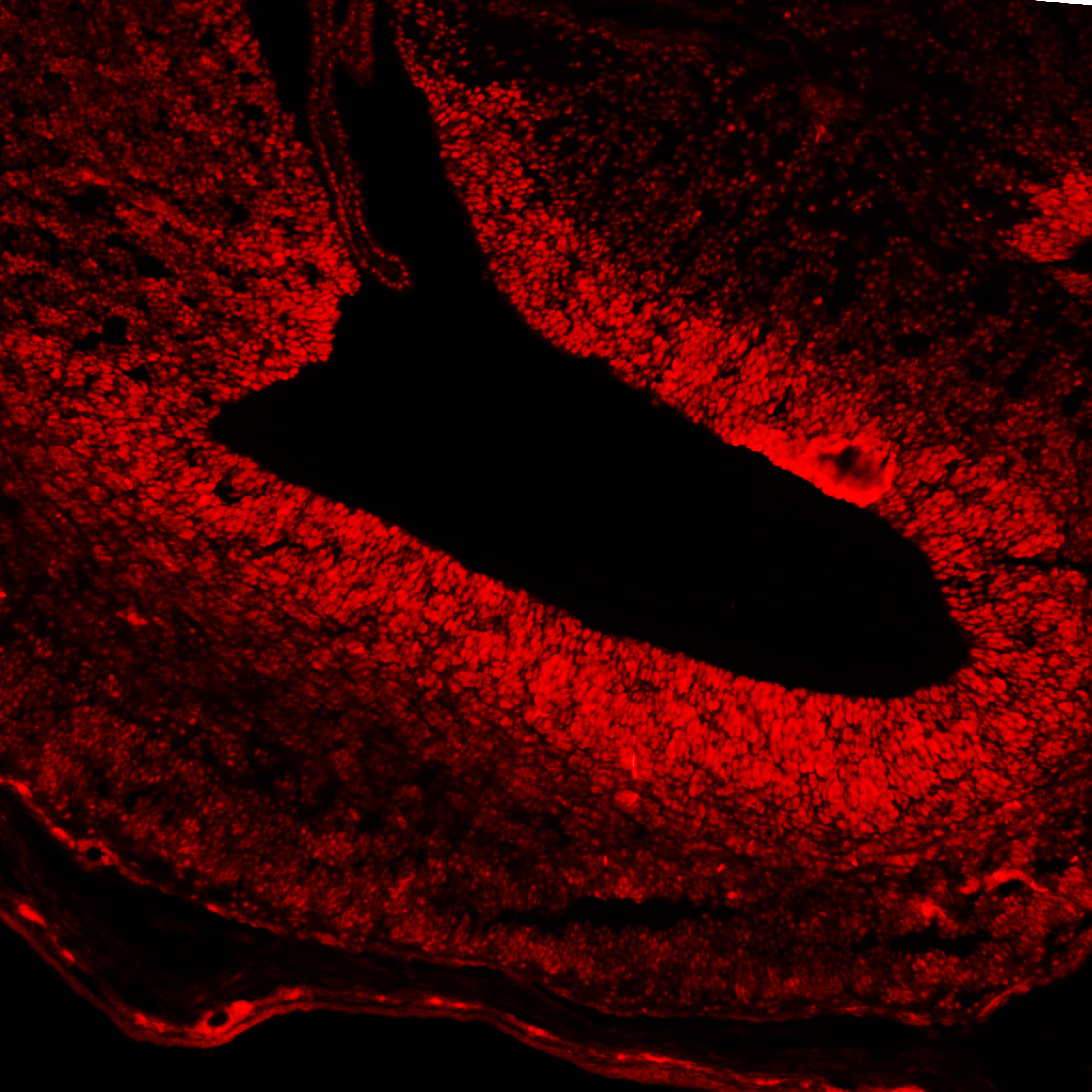

Supplement: Supplementary file 10 — Appendix Figures Source Data [file 44319_2026_768_MOESM10_ESM.zip › Appendix Figures/Appendix Figure S2/S2K/ctrl.jpg]

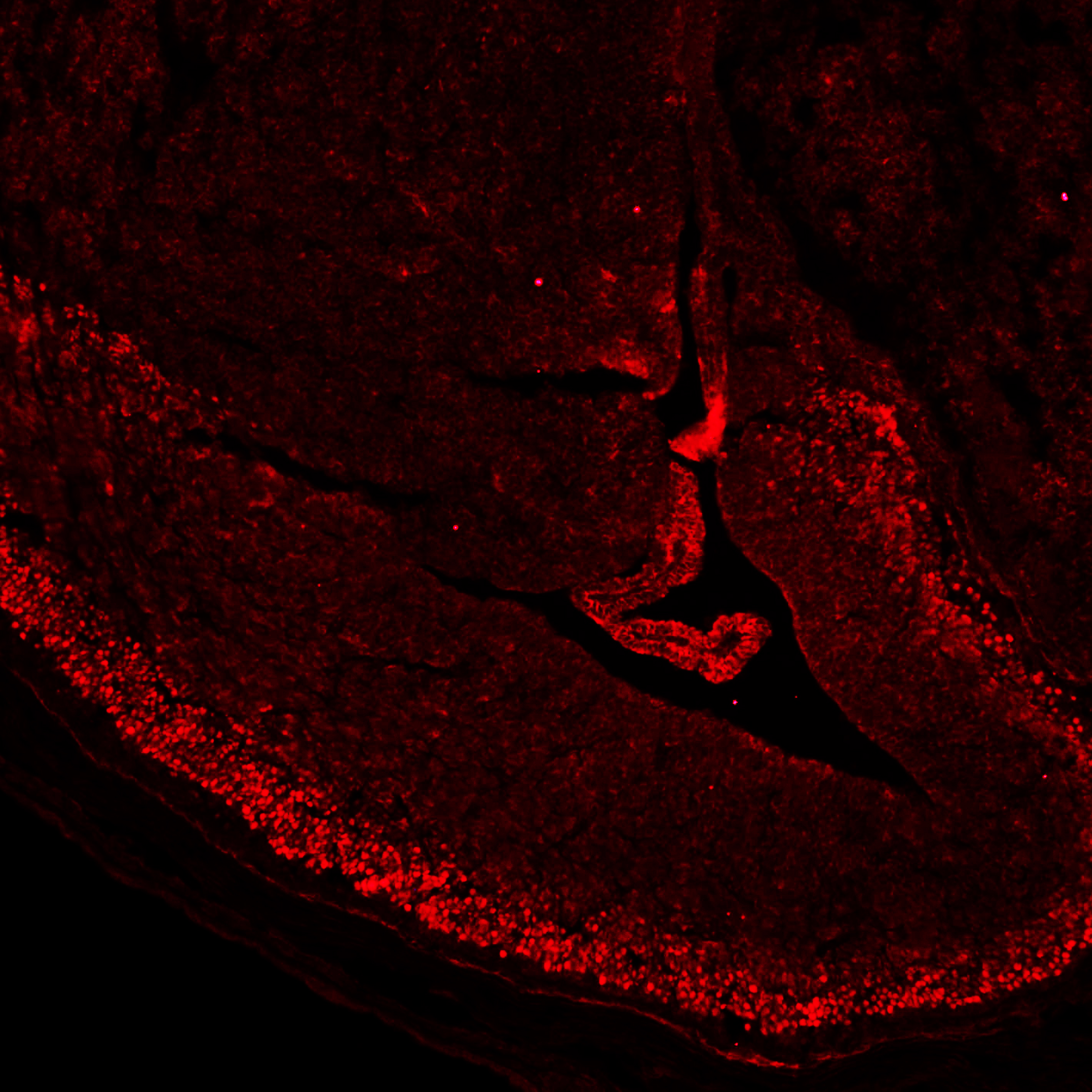

Supplement: Supplementary file 10 — Appendix Figures Source Data [file 44319_2026_768_MOESM10_ESM.zip › Appendix Figures/Appendix Figure S3/S3A/ctko.jpg]

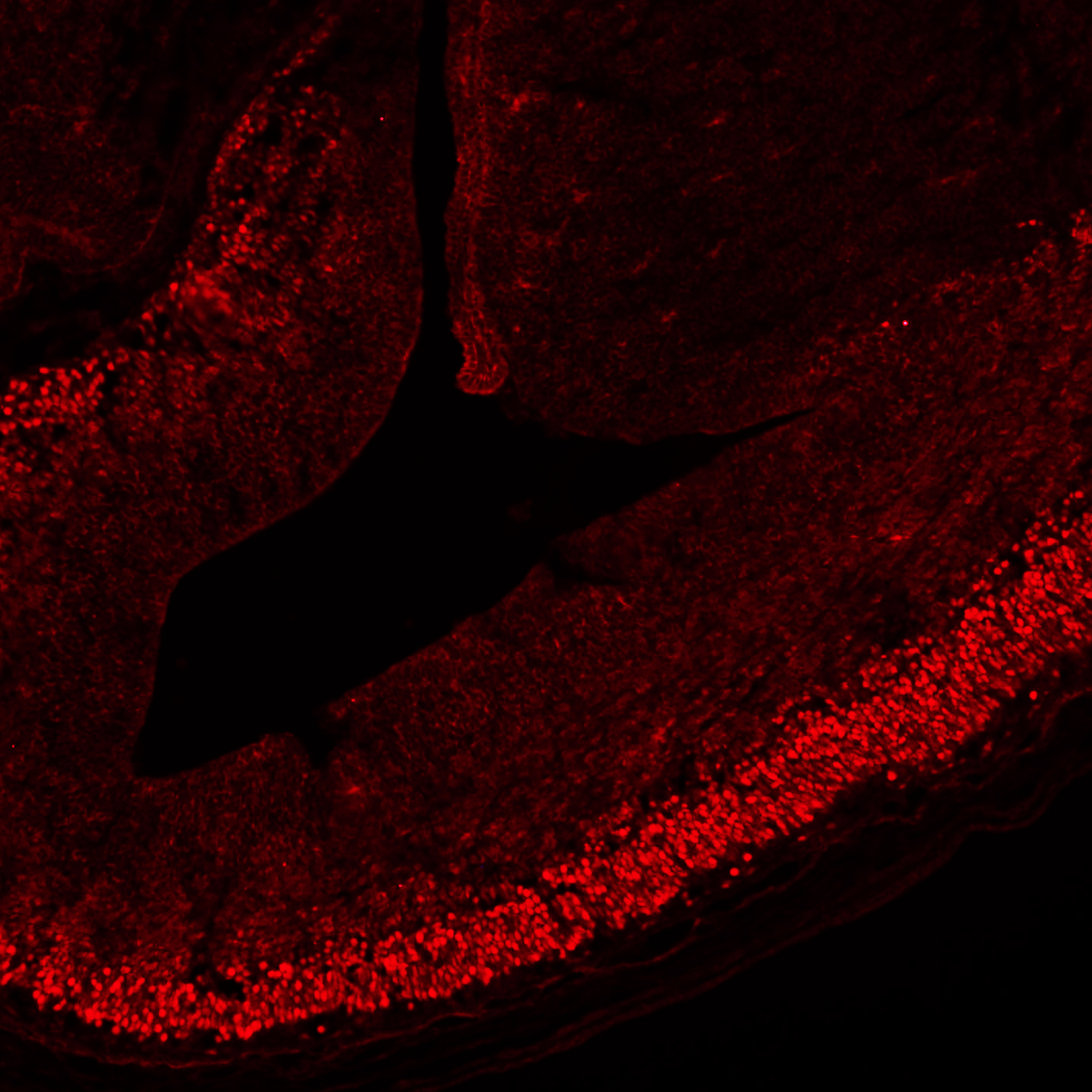

Supplement: Supplementary file 10 — Appendix Figures Source Data [file 44319_2026_768_MOESM10_ESM.zip › Appendix Figures/Appendix Figure S3/S3A/ctl.jpg]

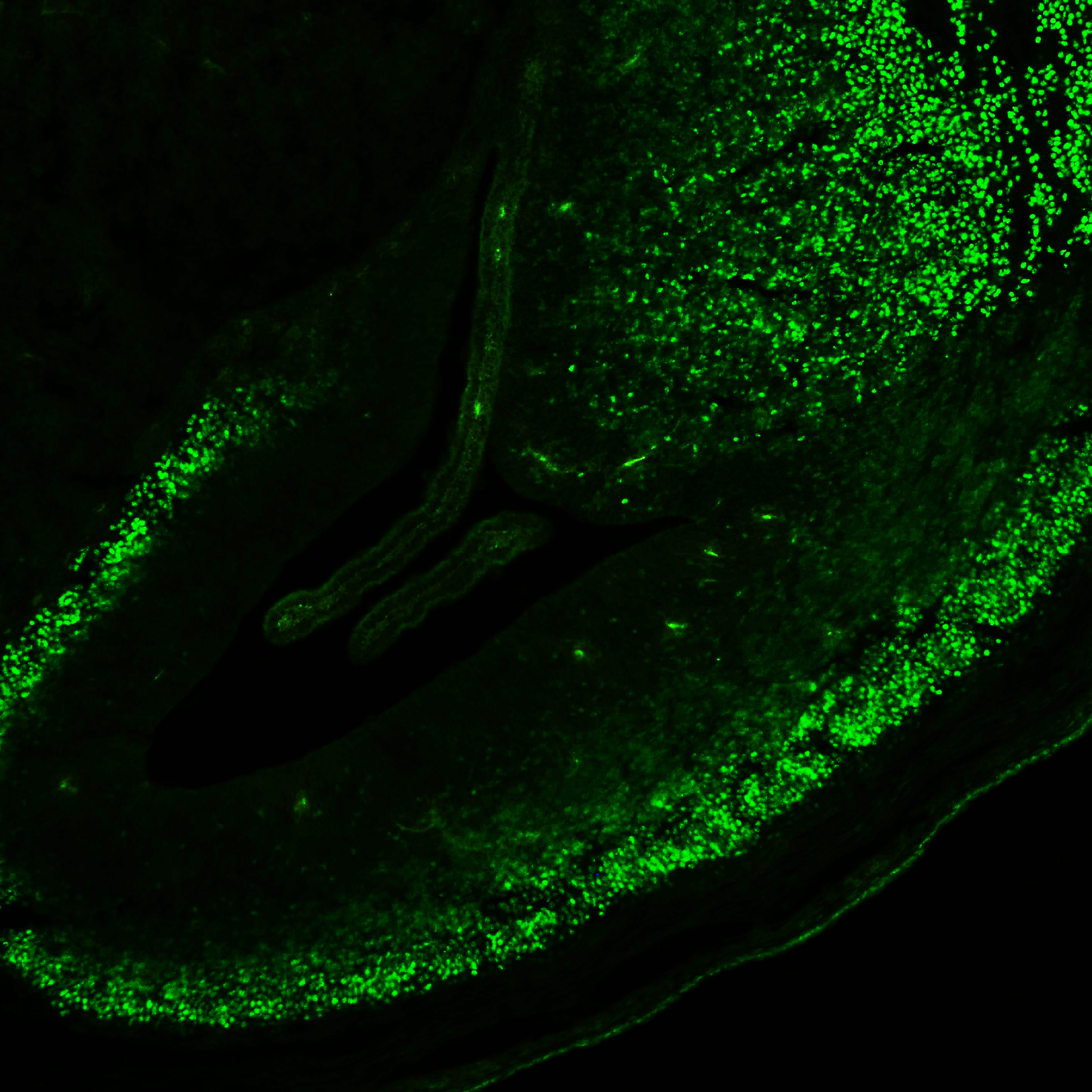

Supplement: Supplementary file 10 — Appendix Figures Source Data [file 44319_2026_768_MOESM10_ESM.zip › Appendix Figures/Appendix Figure S3/S3C/ctko.jpg]

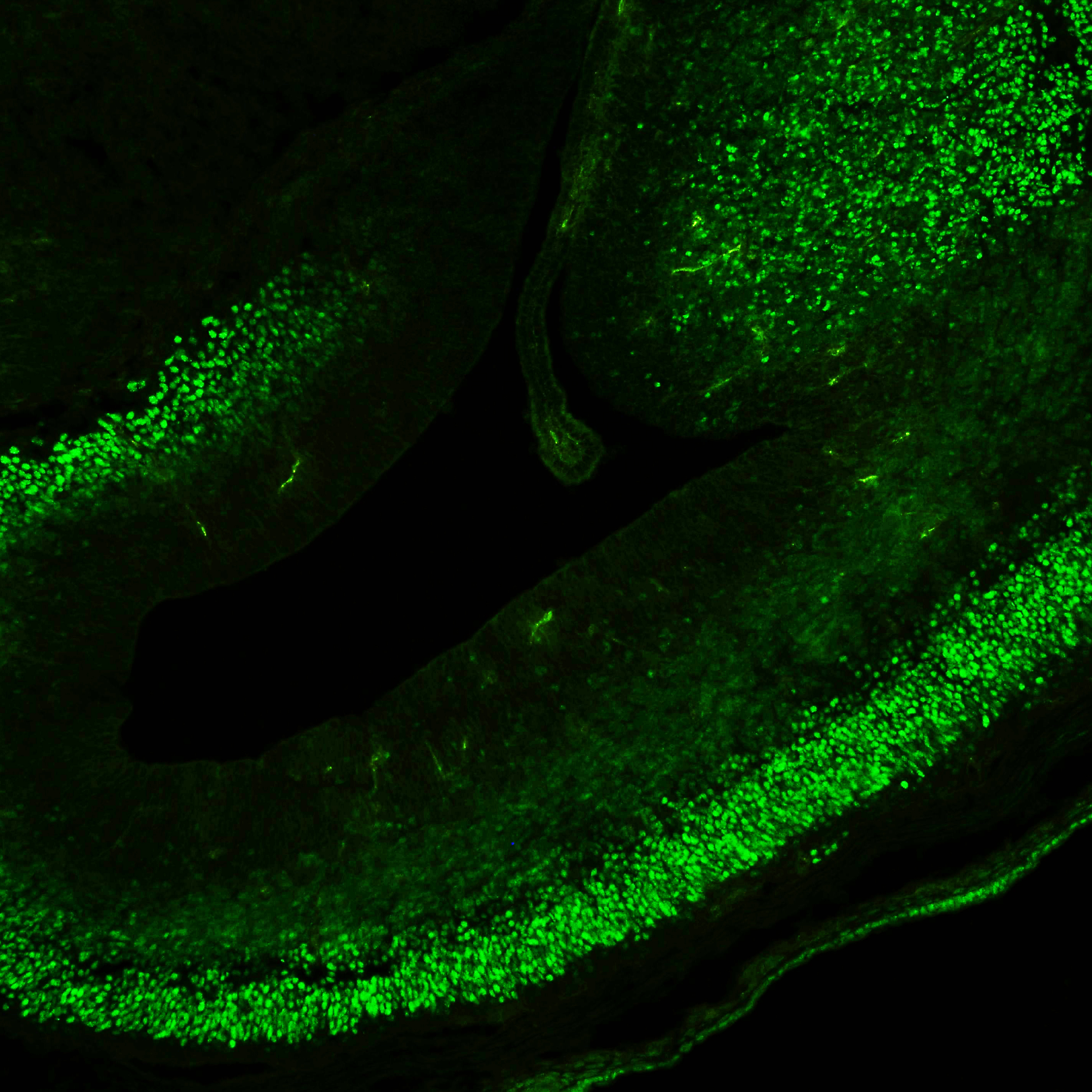

Supplement: Supplementary file 10 — Appendix Figures Source Data [file 44319_2026_768_MOESM10_ESM.zip › Appendix Figures/Appendix Figure S3/S3C/ctrl.jpg]

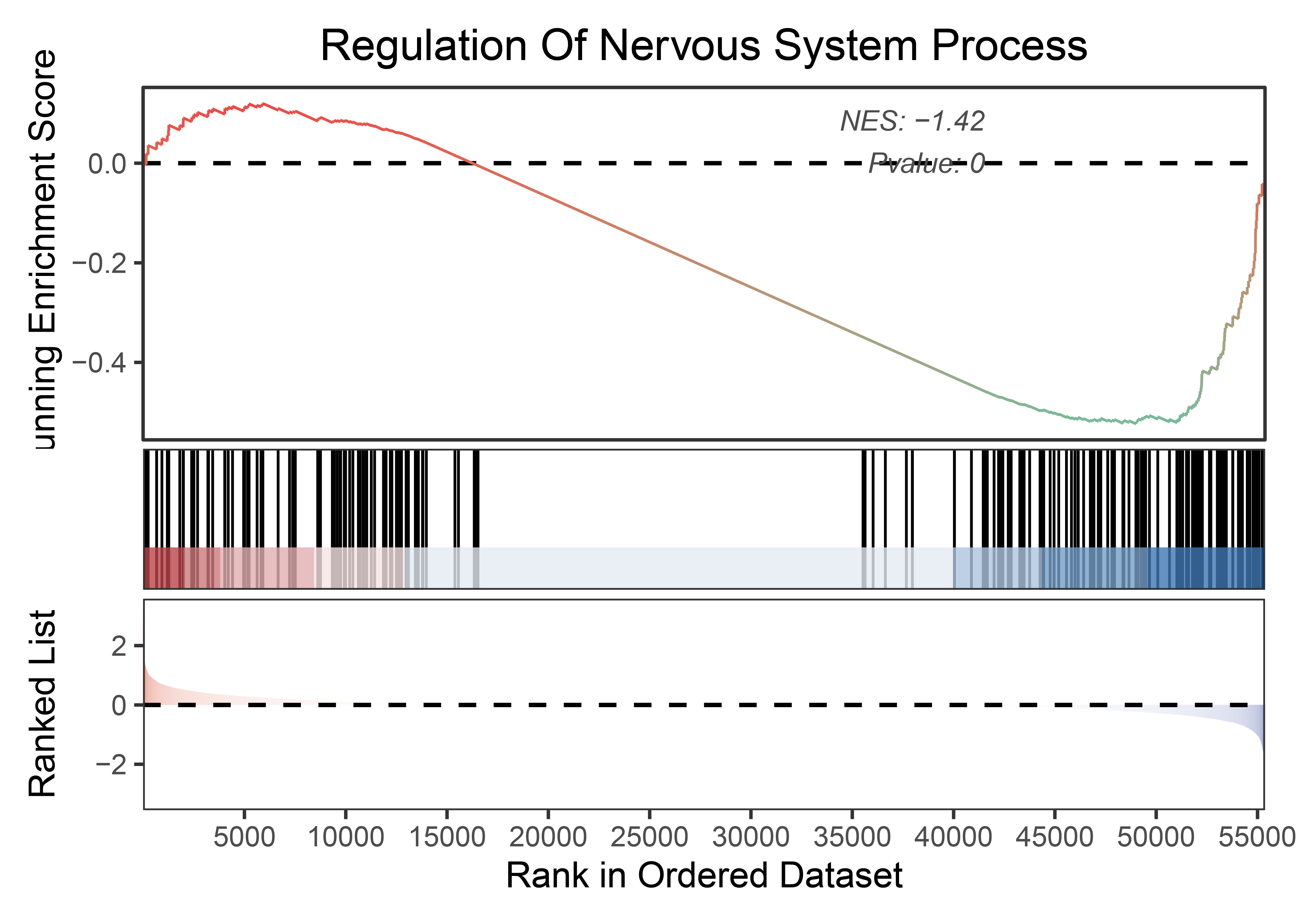

Supplement: Supplementary file 10 — Appendix Figures Source Data [file 44319_2026_768_MOESM10_ESM.zip › Appendix Figures/Appendix Figure S4/S4A/s4a.jpg]

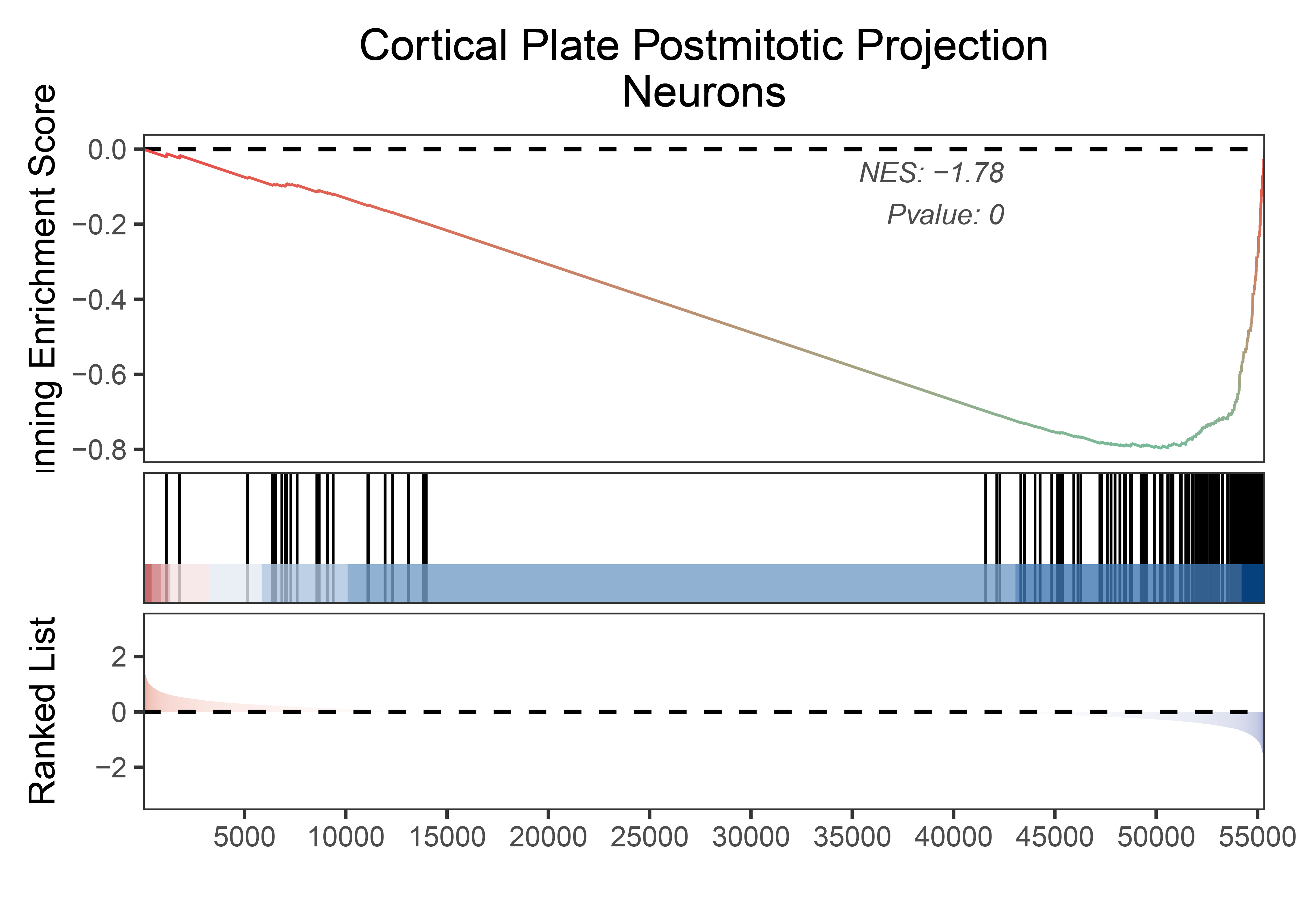

Supplement: Supplementary file 10 — Appendix Figures Source Data [file 44319_2026_768_MOESM10_ESM.zip › Appendix Figures/Appendix Figure S4/S4B/s4b.jpg]

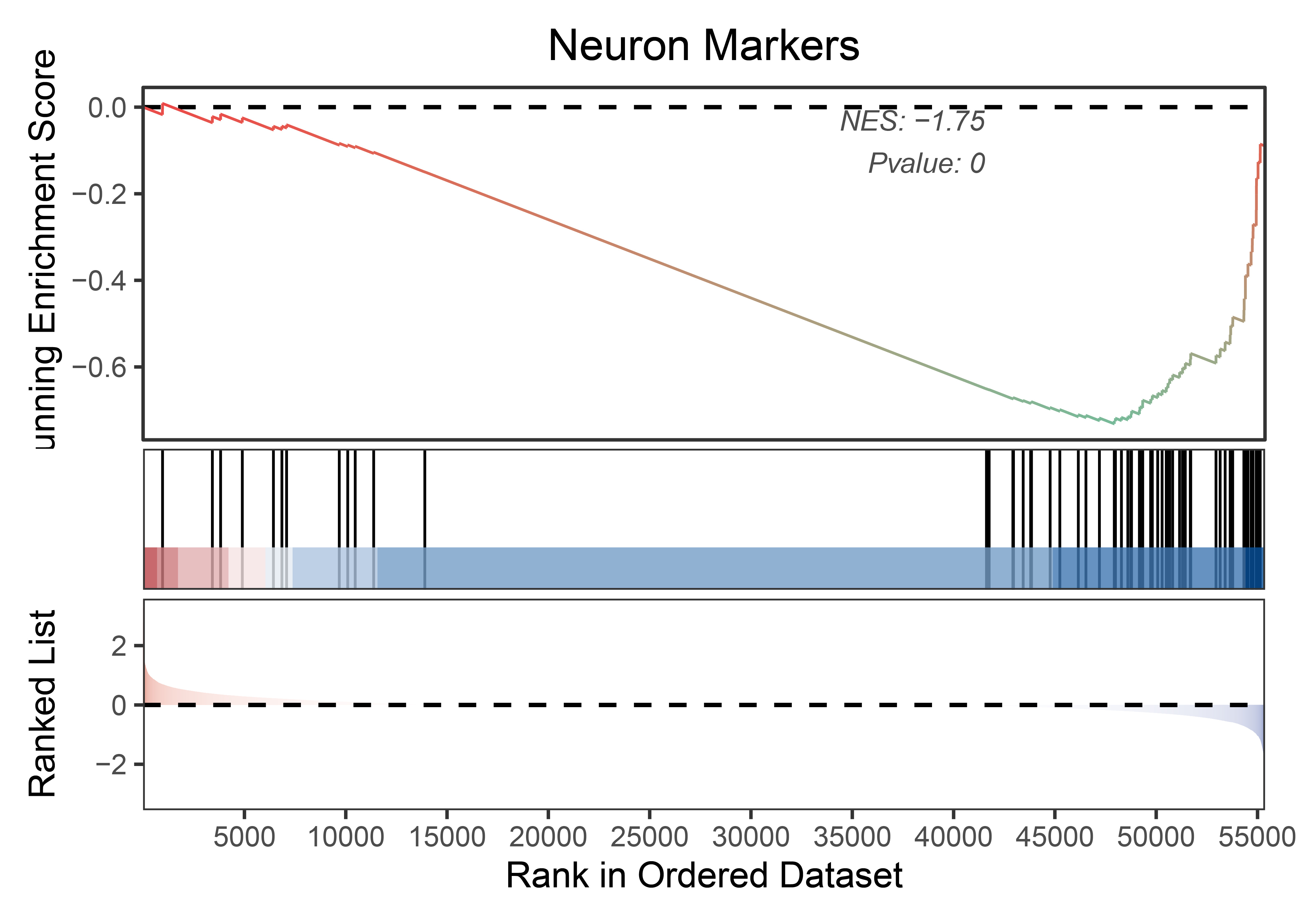

Supplement: Supplementary file 10 — Appendix Figures Source Data [file 44319_2026_768_MOESM10_ESM.zip › Appendix Figures/Appendix Figure S4/S4C/s4c.jpg]

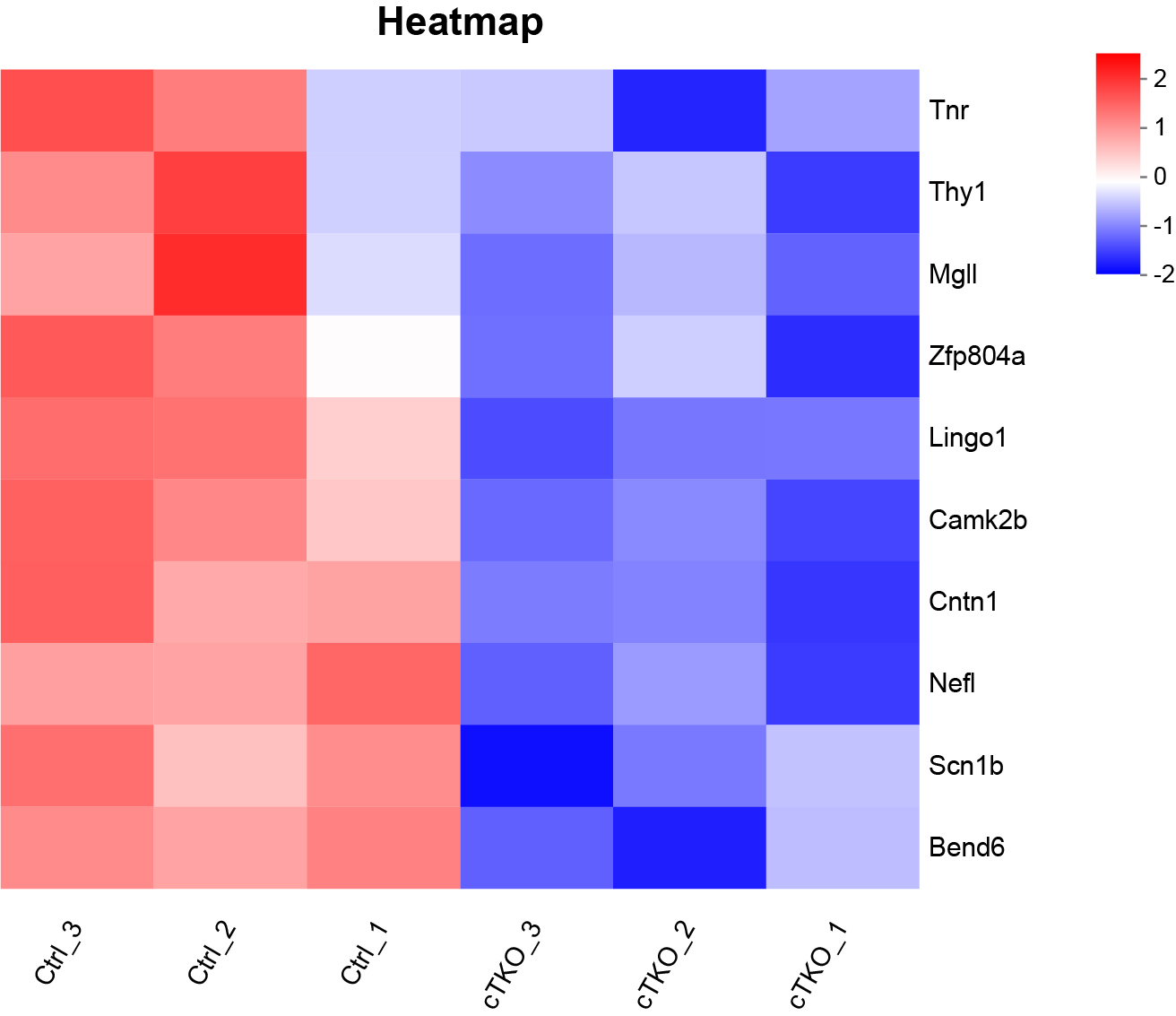

Supplement: Supplementary file 10 — Appendix Figures Source Data [file 44319_2026_768_MOESM10_ESM.zip › Appendix Figures/Appendix Figure S4/S4D/s4d.jpg]

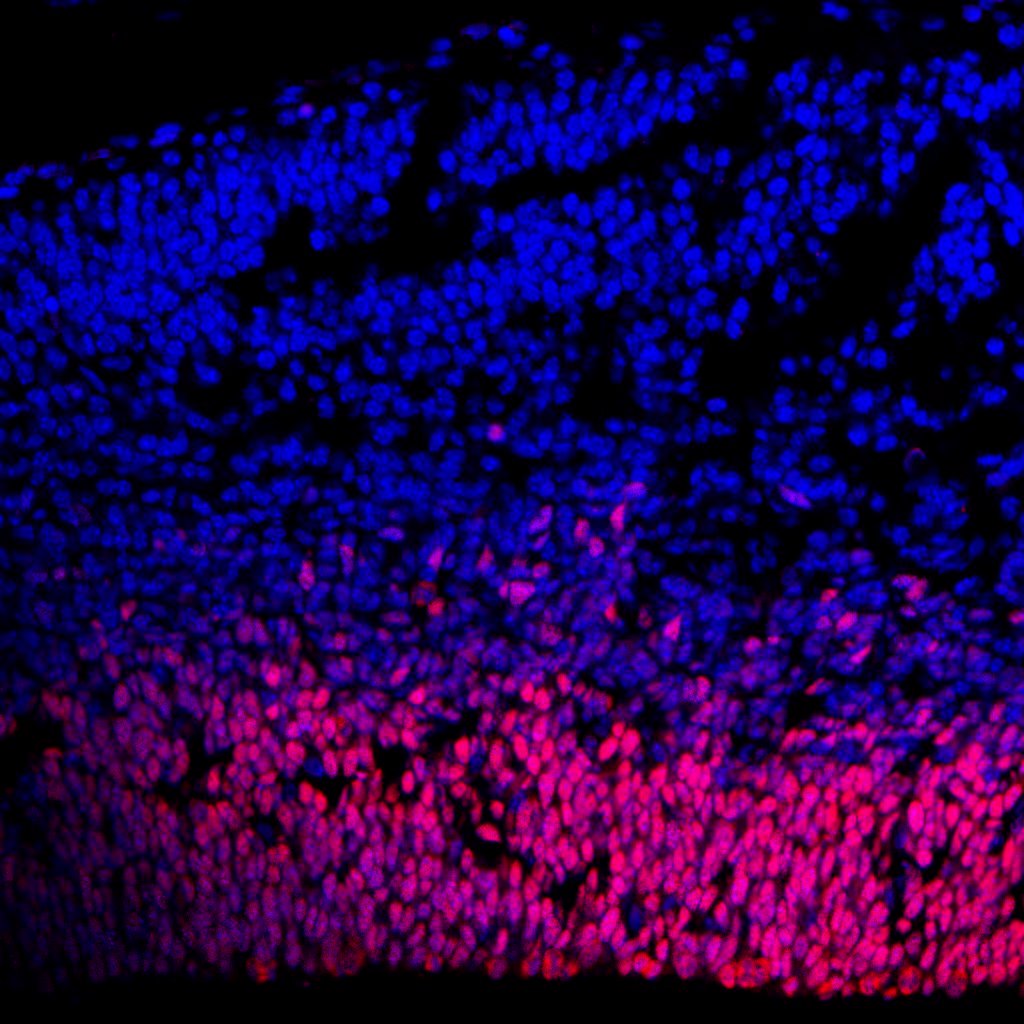

Supplement: Supplementary file 10 — Appendix Figures Source Data [file 44319_2026_768_MOESM10_ESM.zip › Appendix Figures/Appendix Figure S5/S5C/a.jpg]

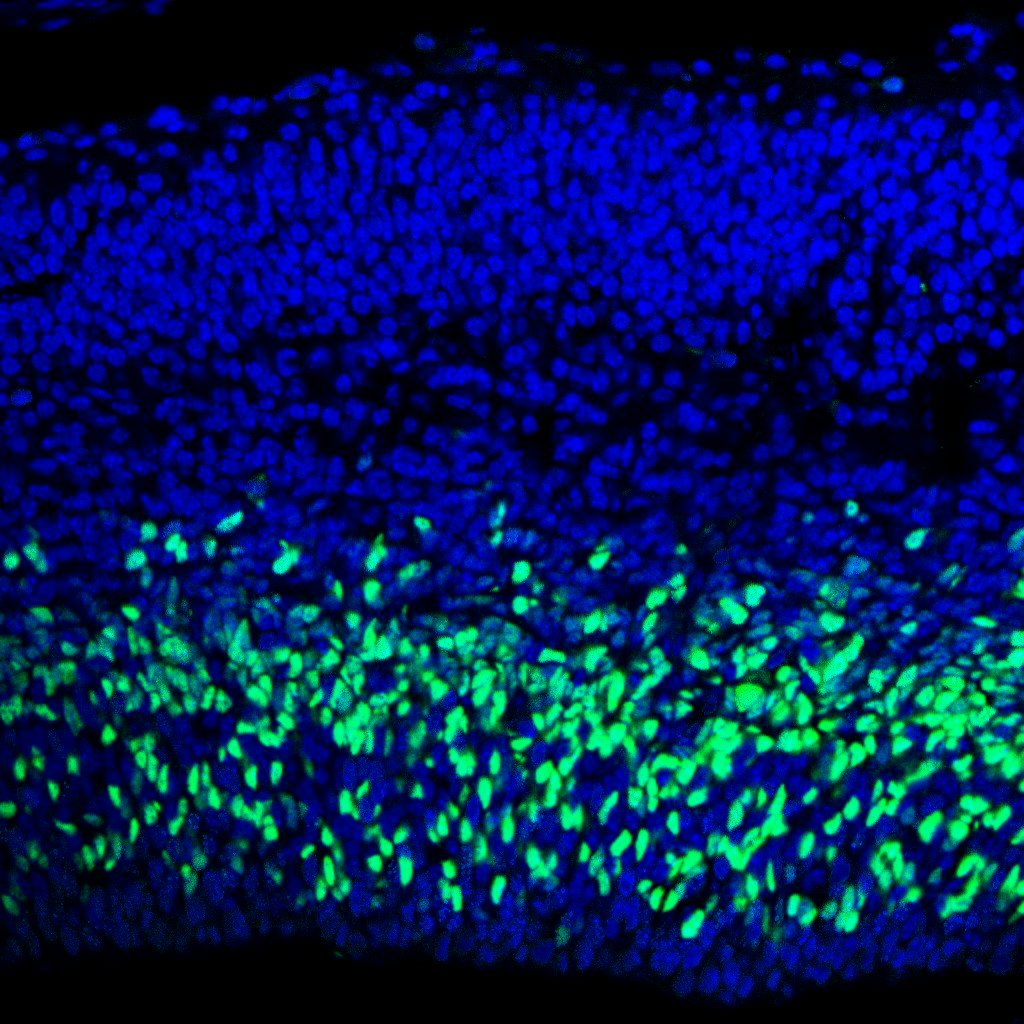

Supplement: Supplementary file 10 — Appendix Figures Source Data [file 44319_2026_768_MOESM10_ESM.zip › Appendix Figures/Appendix Figure S5/S5C/b.jpg]

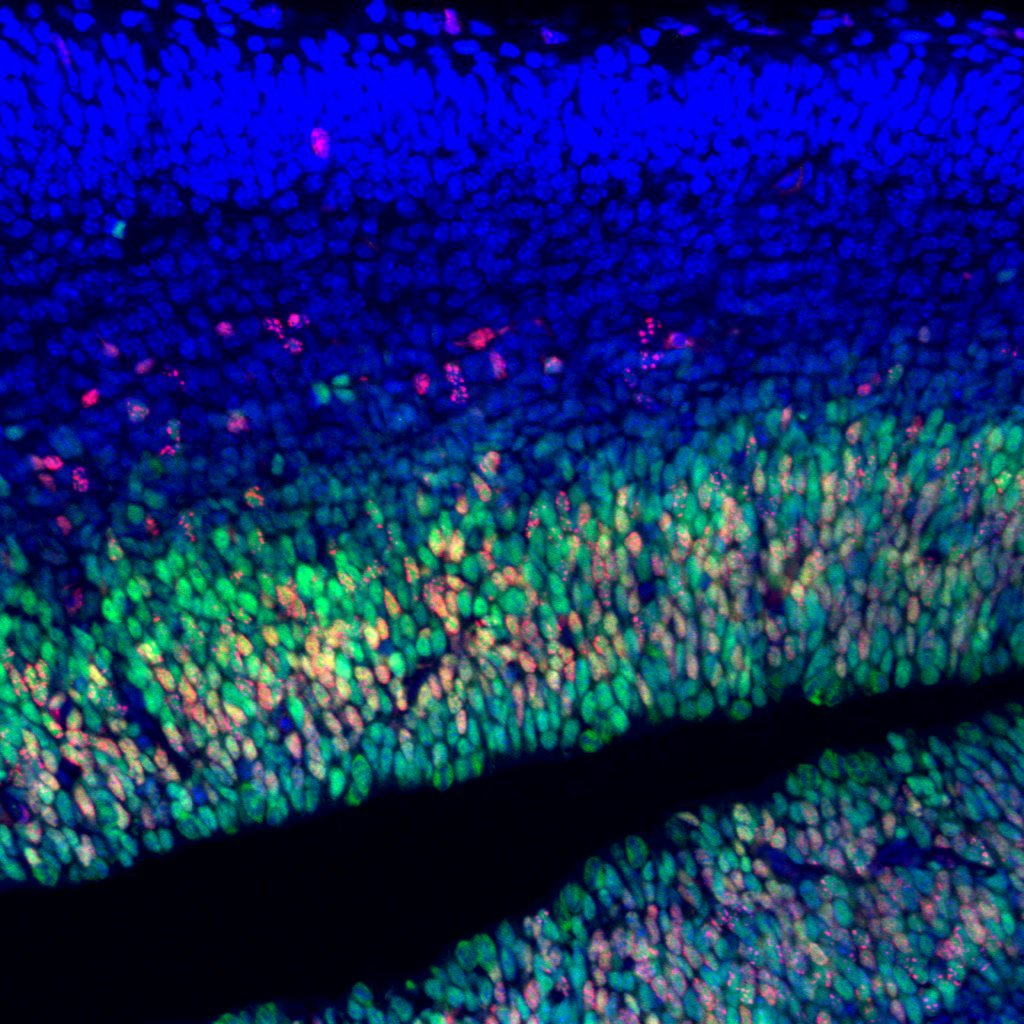

Supplement: Supplementary file 10 — Appendix Figures Source Data [file 44319_2026_768_MOESM10_ESM.zip › Appendix Figures/Appendix Figure S5/S5D/a.jpg]

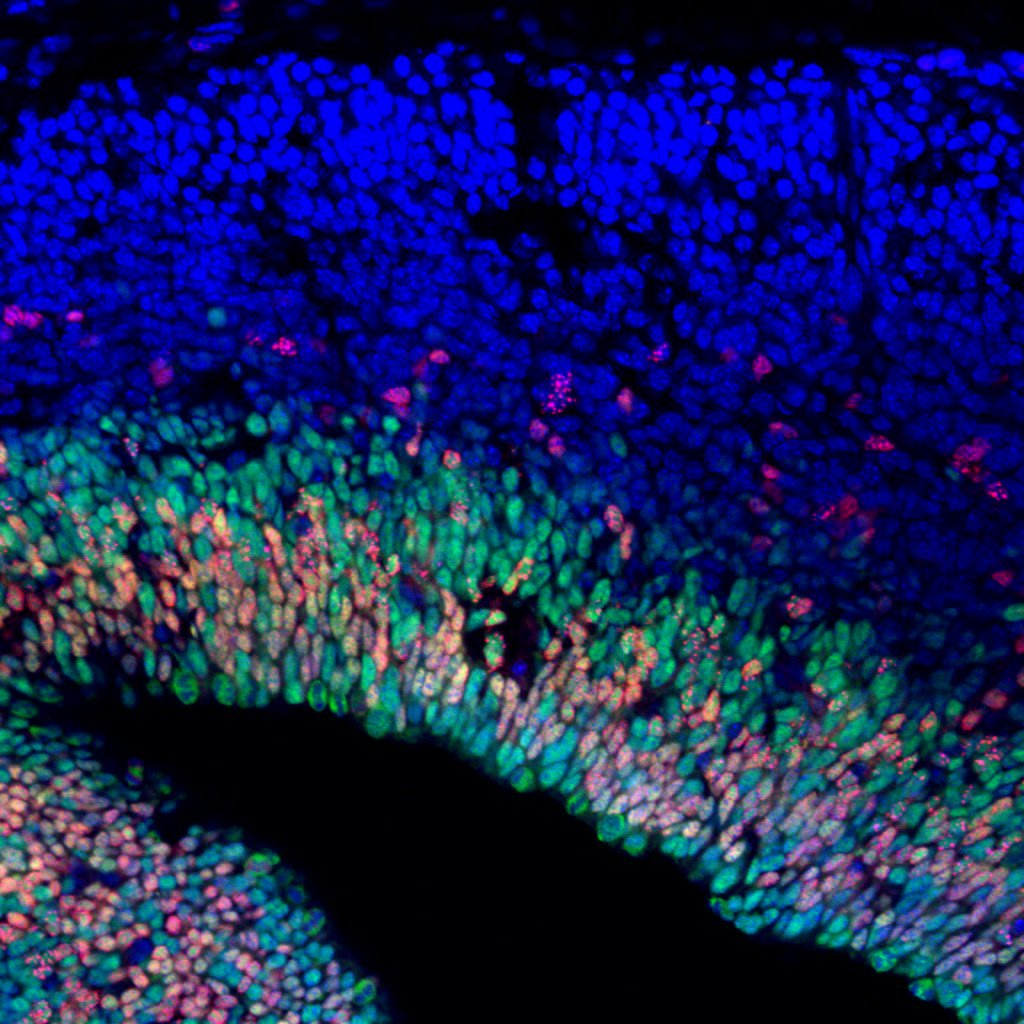

Supplement: Supplementary file 10 — Appendix Figures Source Data [file 44319_2026_768_MOESM10_ESM.zip › Appendix Figures/Appendix Figure S5/S5D/b.jpg]

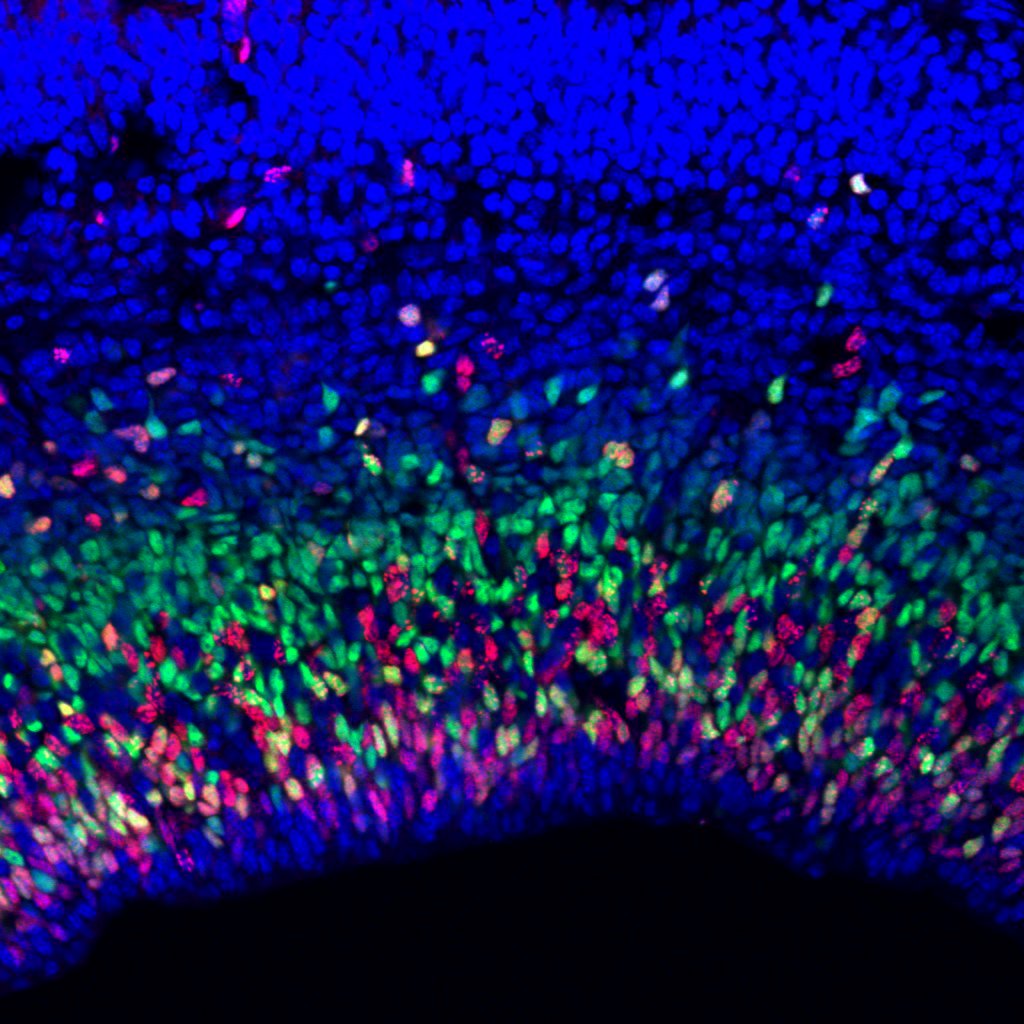

Supplement: Supplementary file 10 — Appendix Figures Source Data [file 44319_2026_768_MOESM10_ESM.zip › Appendix Figures/Appendix Figure S5/S5D/c.jpg]

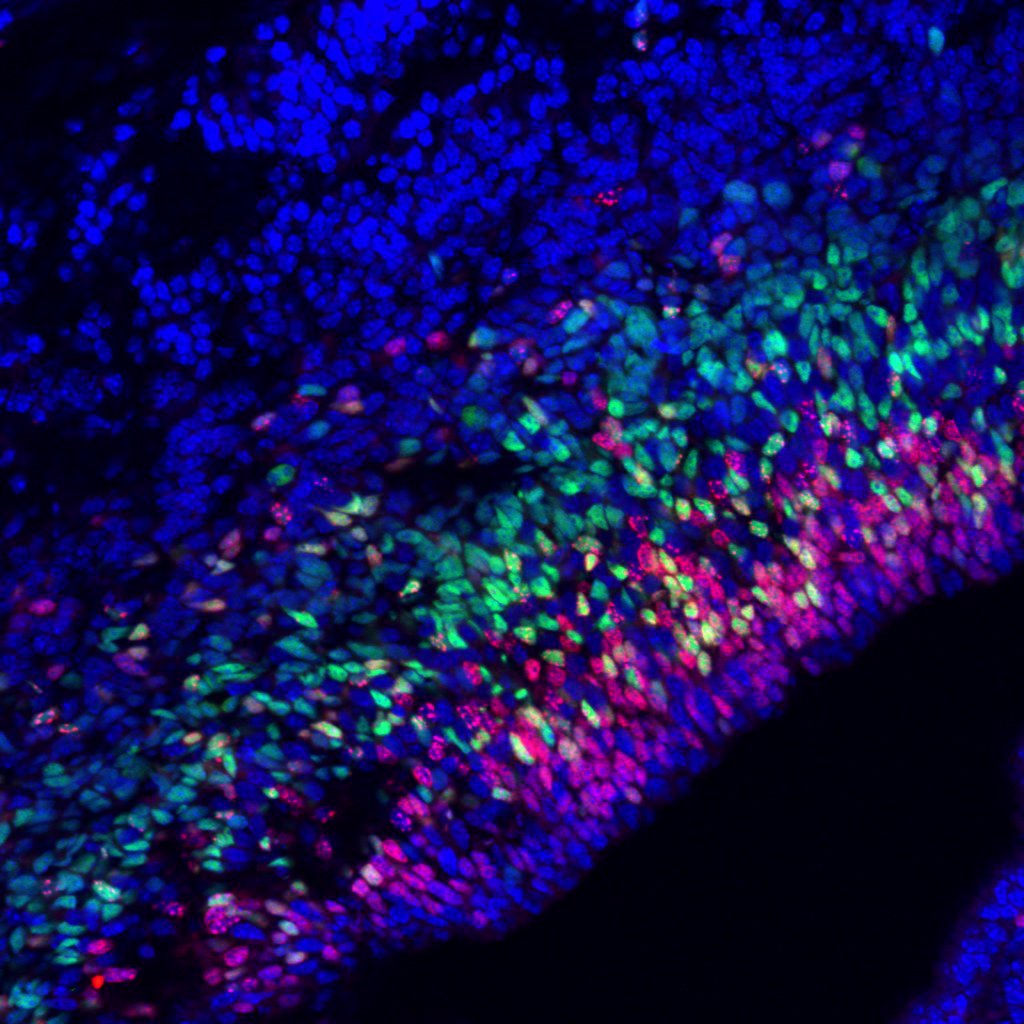

Supplement: Supplementary file 10 — Appendix Figures Source Data [file 44319_2026_768_MOESM10_ESM.zip › Appendix Figures/Appendix Figure S5/S5D/d.jpg]

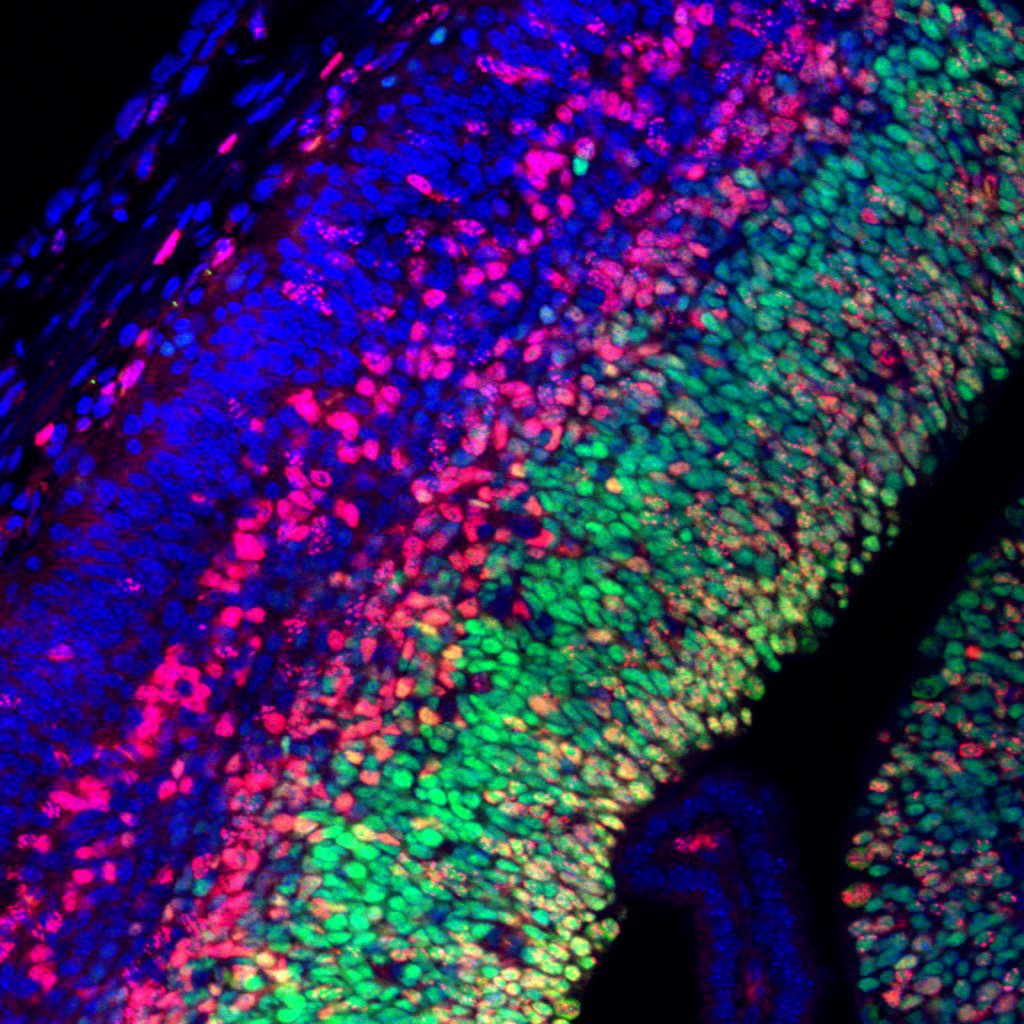

Supplement: Supplementary file 10 — Appendix Figures Source Data [file 44319_2026_768_MOESM10_ESM.zip › Appendix Figures/Appendix Figure S5/S5E/a.jpg]

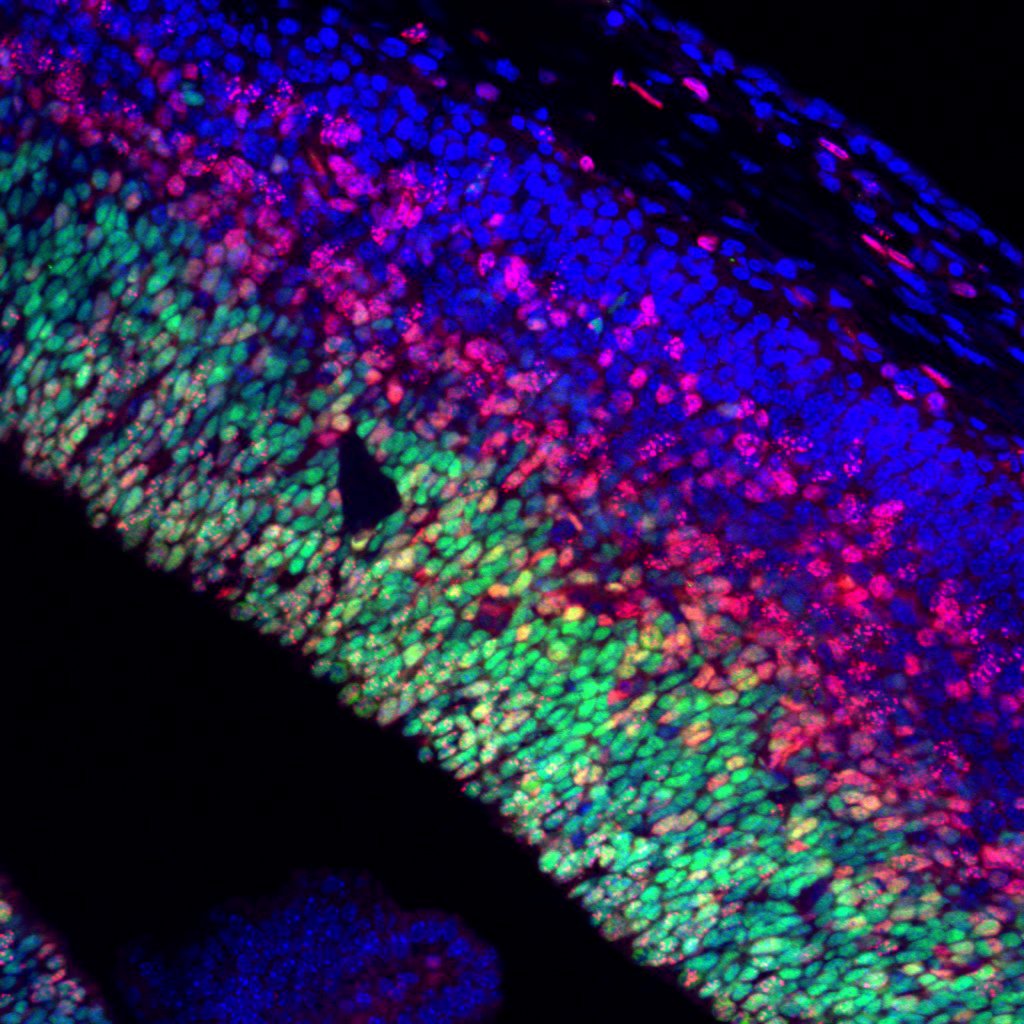

Supplement: Supplementary file 10 — Appendix Figures Source Data [file 44319_2026_768_MOESM10_ESM.zip › Appendix Figures/Appendix Figure S5/S5E/b.jpg]

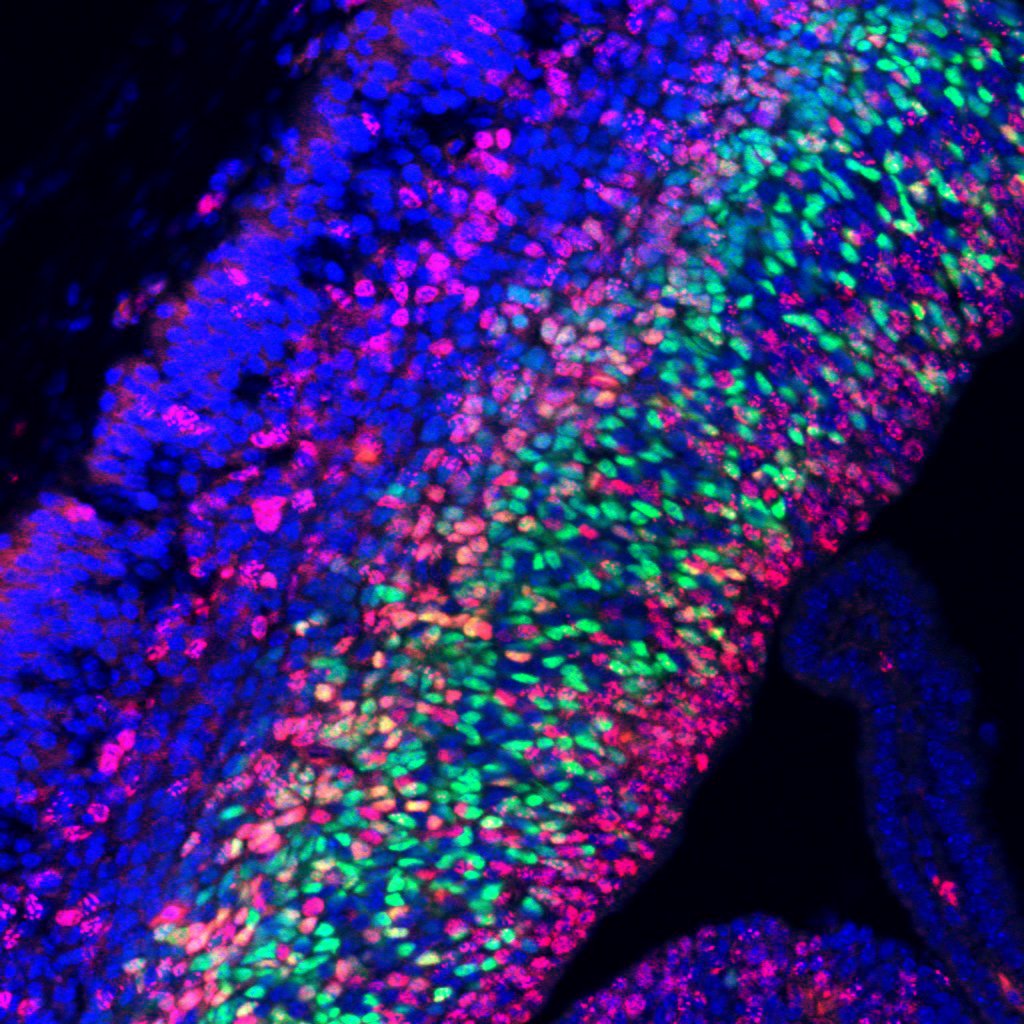

Supplement: Supplementary file 10 — Appendix Figures Source Data [file 44319_2026_768_MOESM10_ESM.zip › Appendix Figures/Appendix Figure S5/S5E/c.jpg]

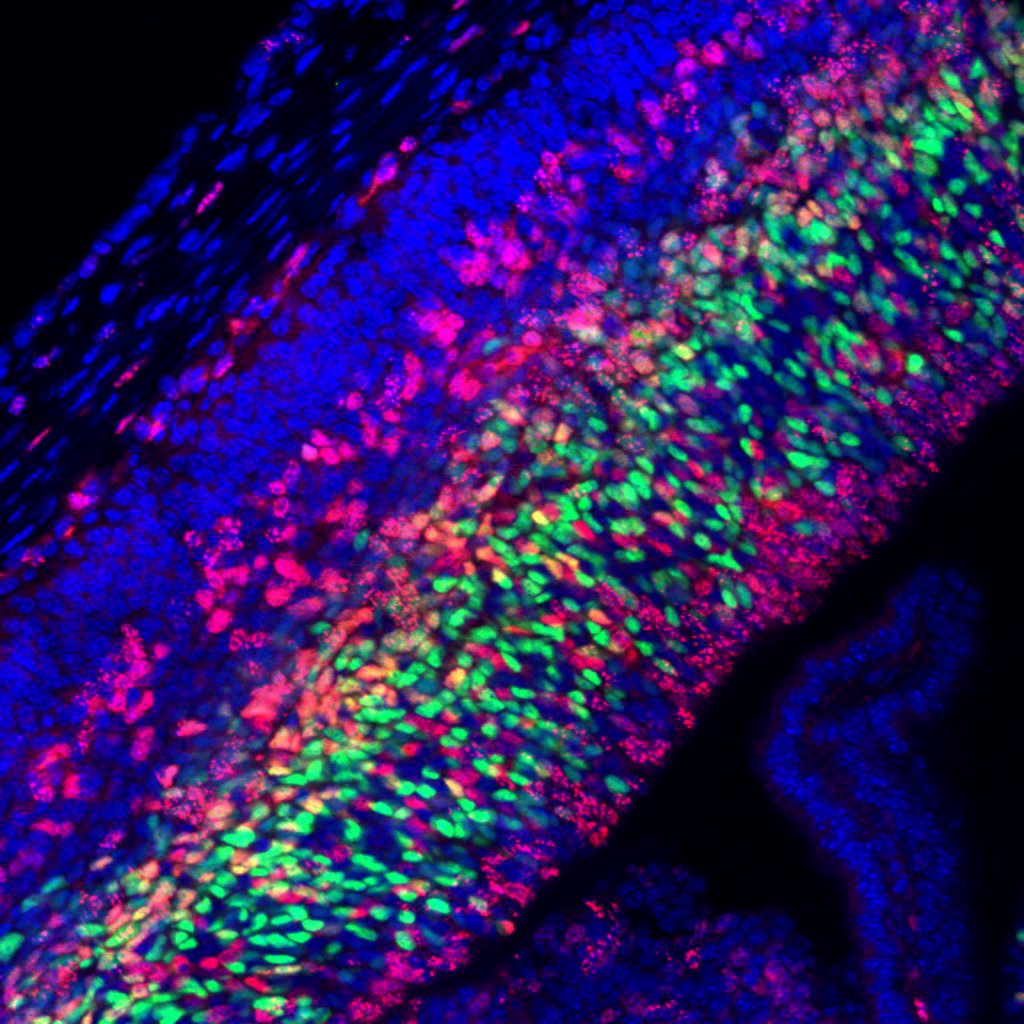

Supplement: Supplementary file 10 — Appendix Figures Source Data [file 44319_2026_768_MOESM10_ESM.zip › Appendix Figures/Appendix Figure S5/S5E/d.jpg]

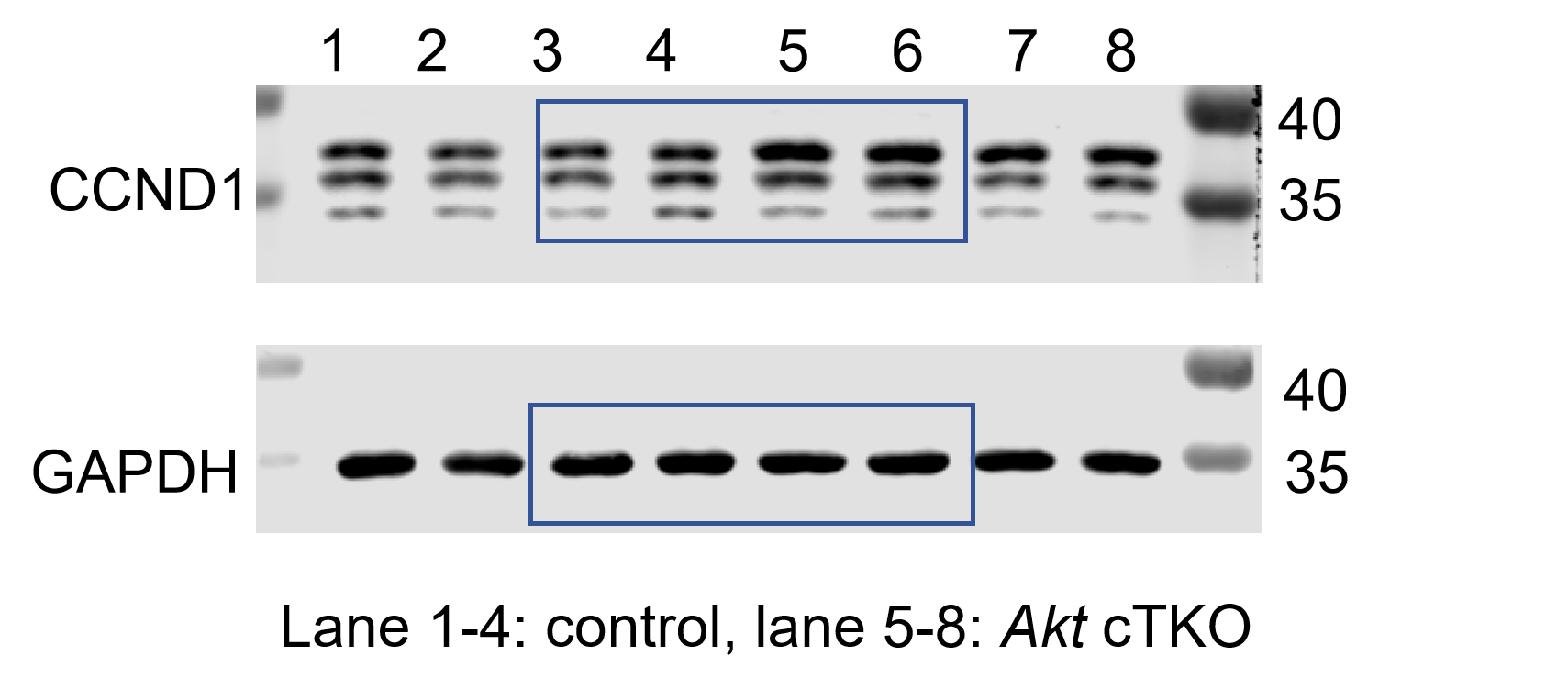

Supplement: Supplementary file 10 — Appendix Figures Source Data [file 44319_2026_768_MOESM10_ESM.zip › Appendix Figures/Appendix Figure S6/S6A/S6A.tif]

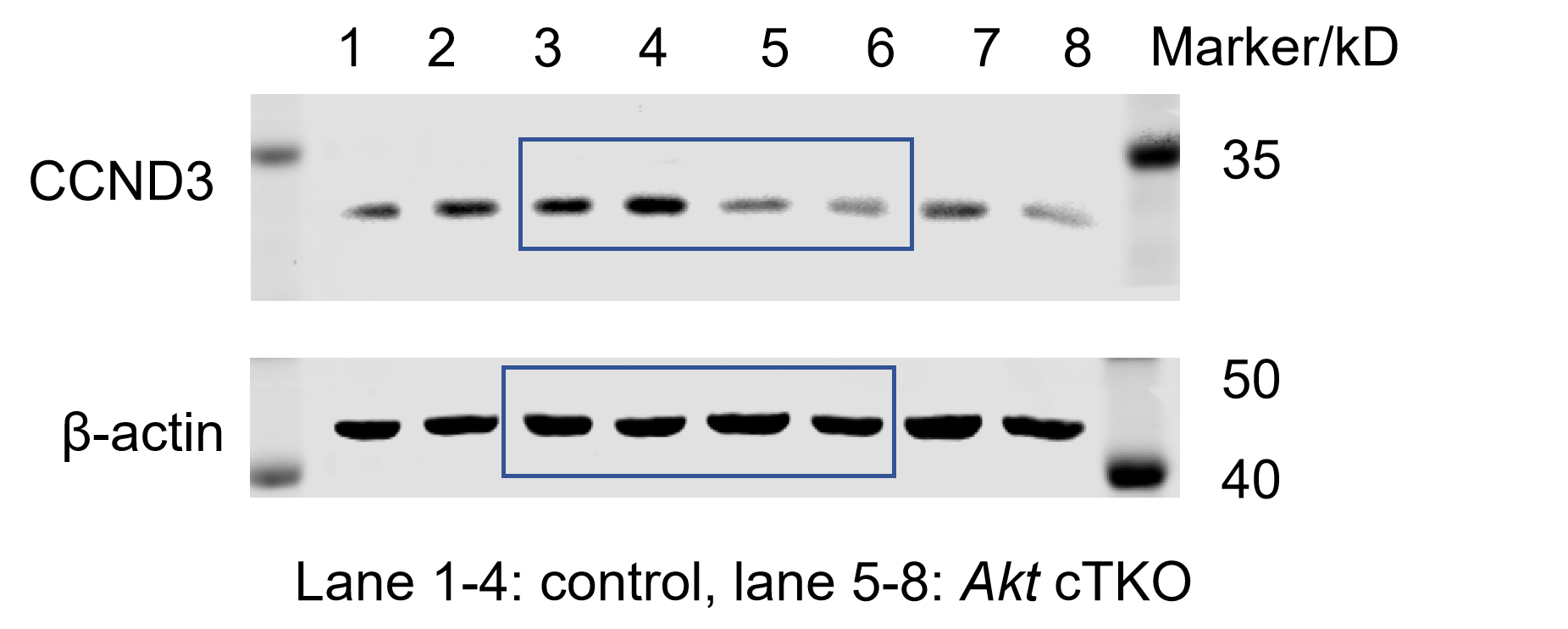

Supplement: Supplementary file 10 — Appendix Figures Source Data [file 44319_2026_768_MOESM10_ESM.zip › Appendix Figures/Appendix Figure S6/S6B/S6B.tif]

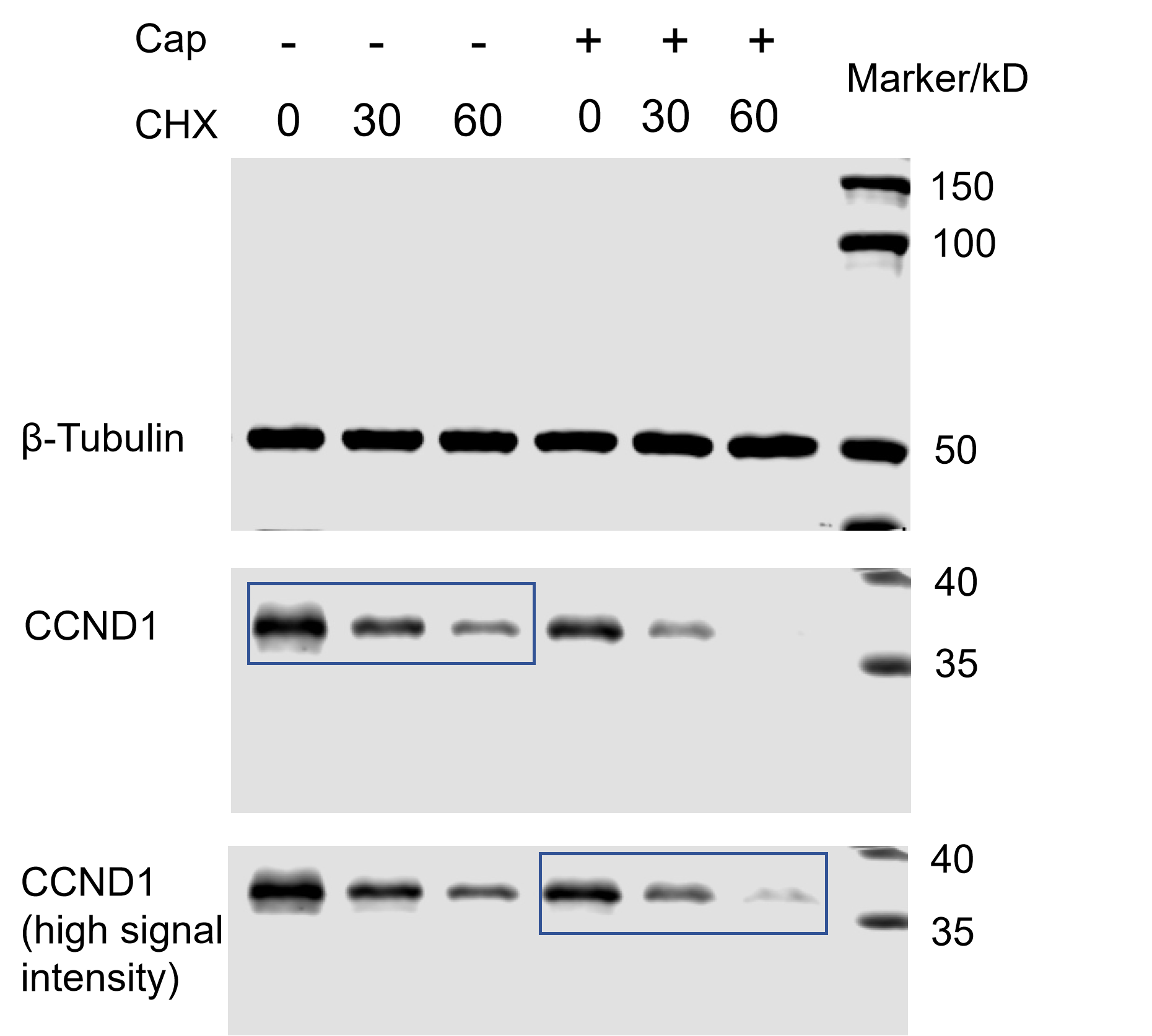

Supplement: Supplementary file 10 — Appendix Figures Source Data [file 44319_2026_768_MOESM10_ESM.zip › Appendix Figures/Appendix Figure S6/S6F/S6F.tif]

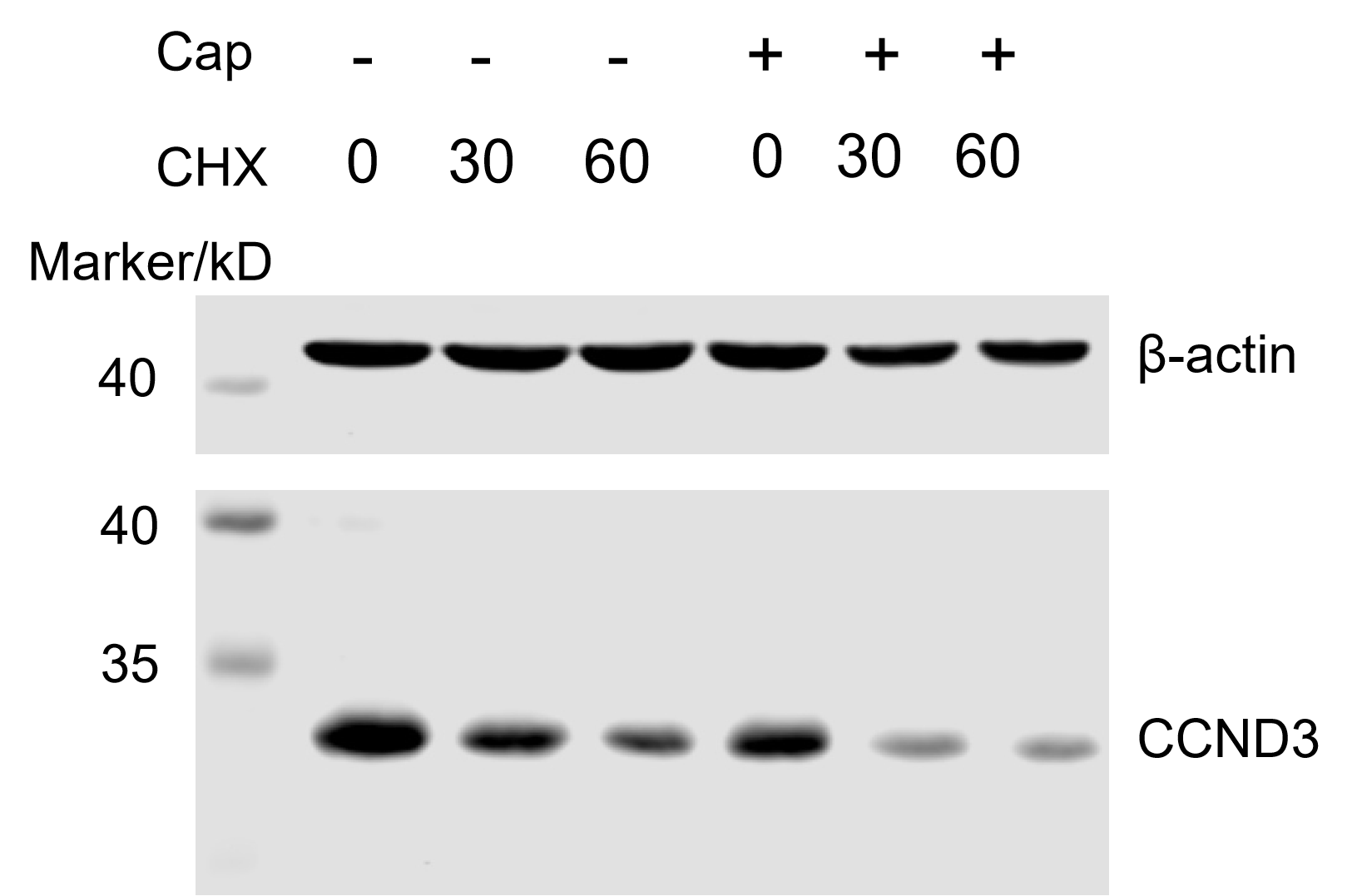

Supplement: Supplementary file 10 — Appendix Figures Source Data [file 44319_2026_768_MOESM10_ESM.zip › Appendix Figures/Appendix Figure S6/S6H/S6H.tif]

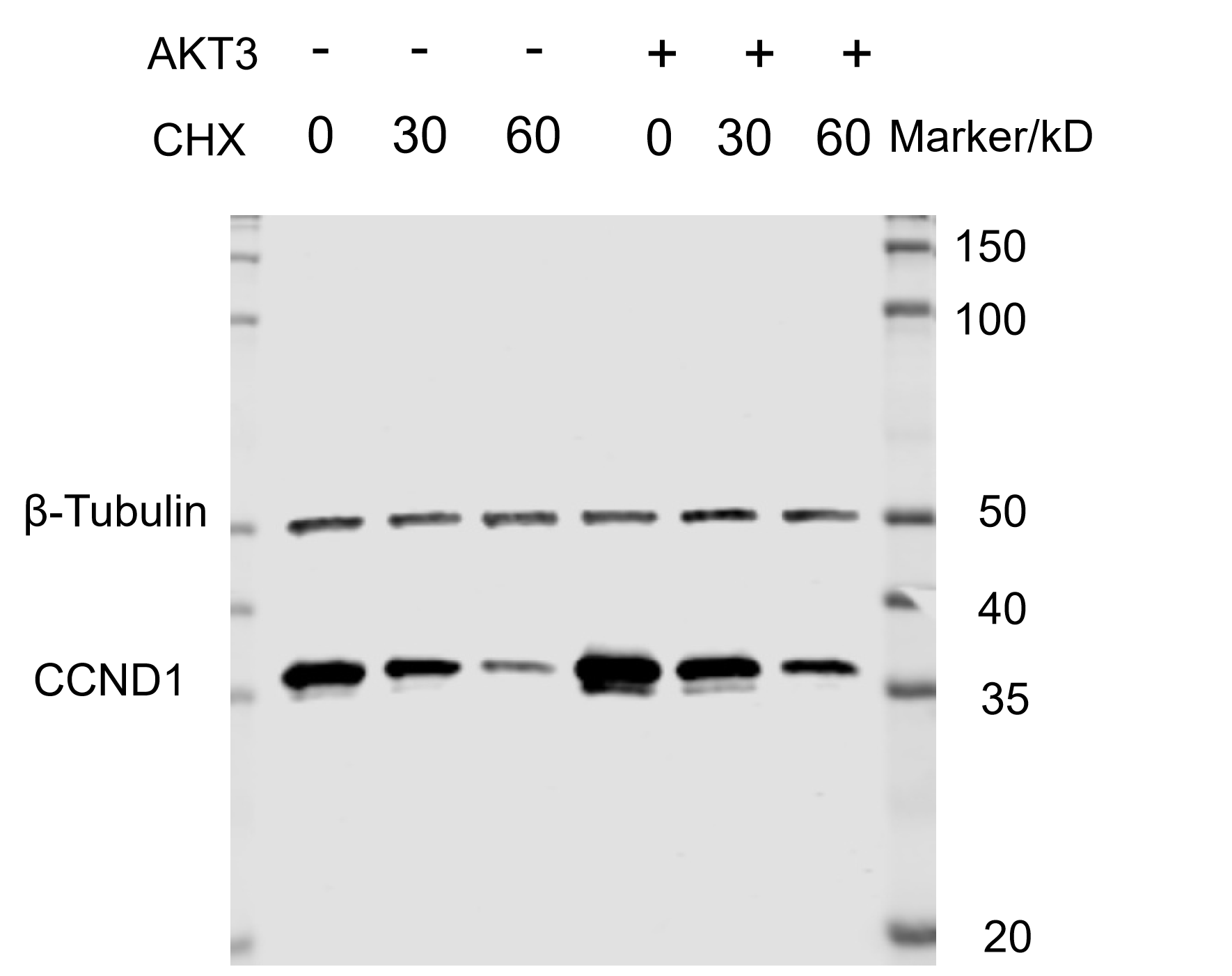

Supplement: Supplementary file 10 — Appendix Figures Source Data [file 44319_2026_768_MOESM10_ESM.zip › Appendix Figures/Appendix Figure S6/S6J/6J.tif]

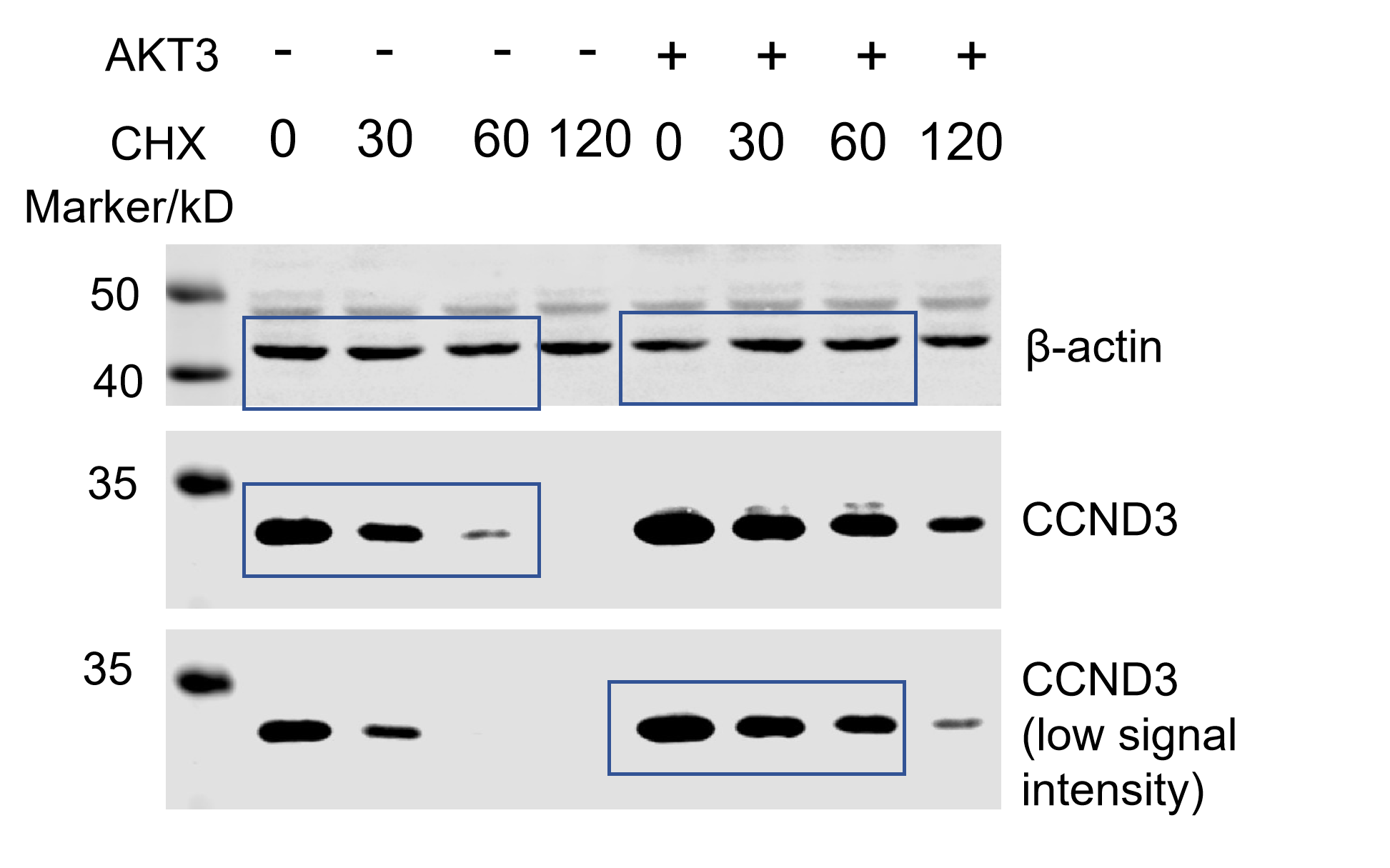

Supplement: Supplementary file 10 — Appendix Figures Source Data [file 44319_2026_768_MOESM10_ESM.zip › Appendix Figures/Appendix Figure S6/S6L/S6L.tif]

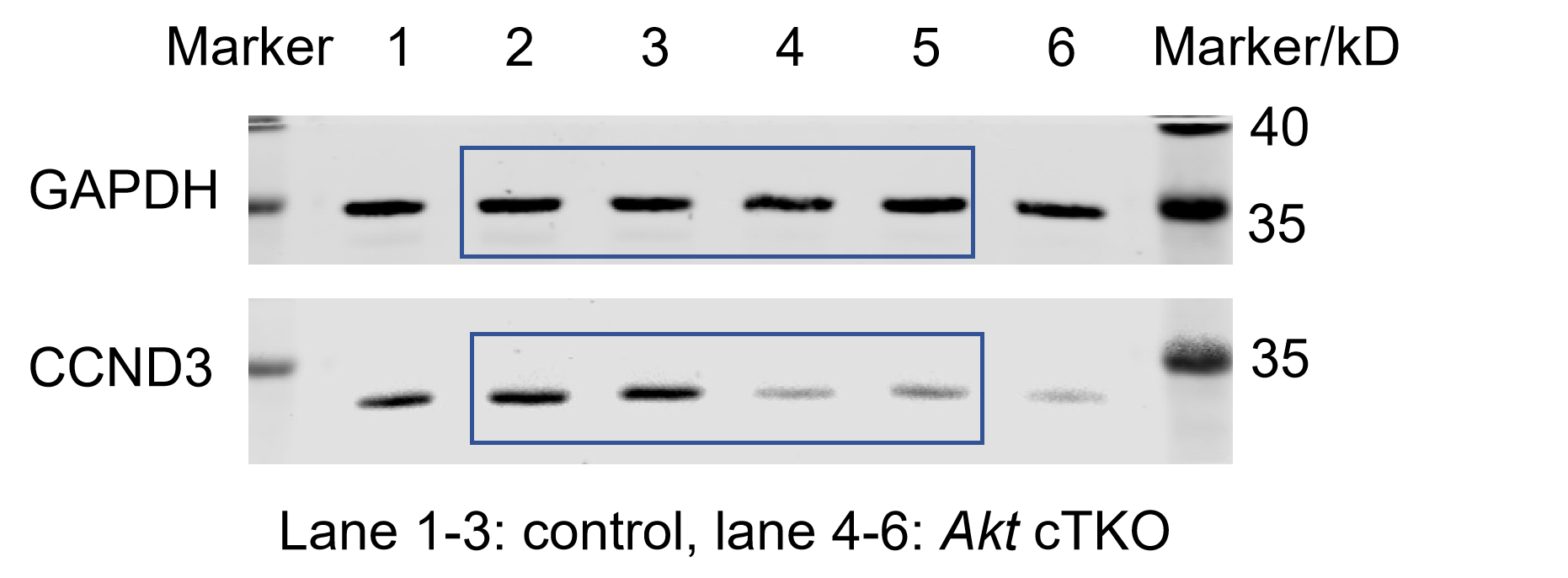

Supplement: Supplementary file 10 — Appendix Figures Source Data [file 44319_2026_768_MOESM10_ESM.zip › Appendix Figures/Appendix Figure S6/S6N/S6N.tif]

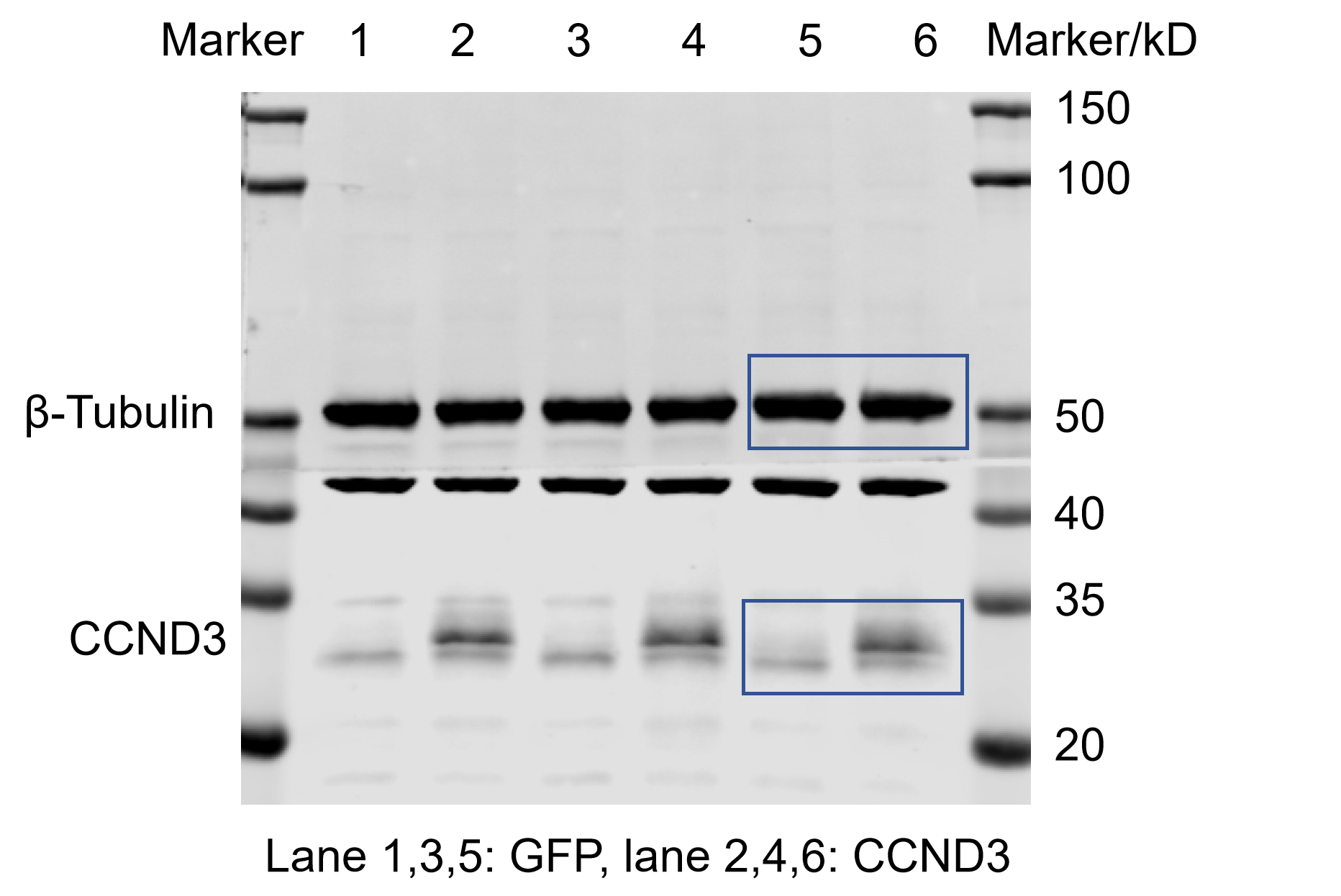

Supplement: Supplementary file 10 — Appendix Figures Source Data [file 44319_2026_768_MOESM10_ESM.zip › Appendix Figures/Appendix Figure S6/S6P/S6P.tif]

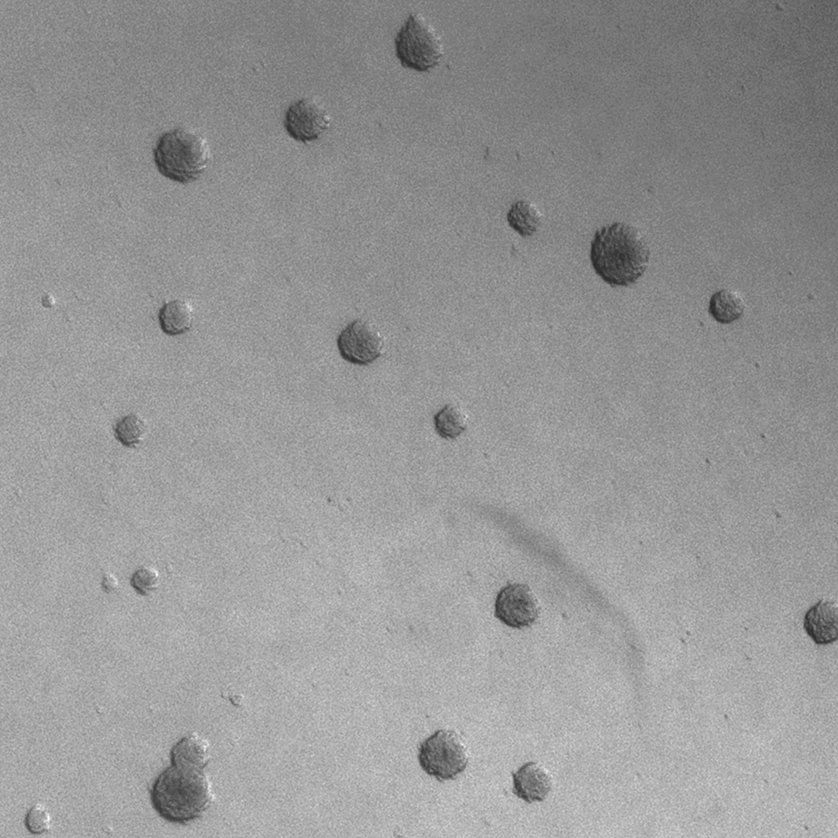

Supplement: Supplementary file 10 — Appendix Figures Source Data [file 44319_2026_768_MOESM10_ESM.zip › Appendix Figures/Appendix Figure S6/S6R/ccnd3.jpg]

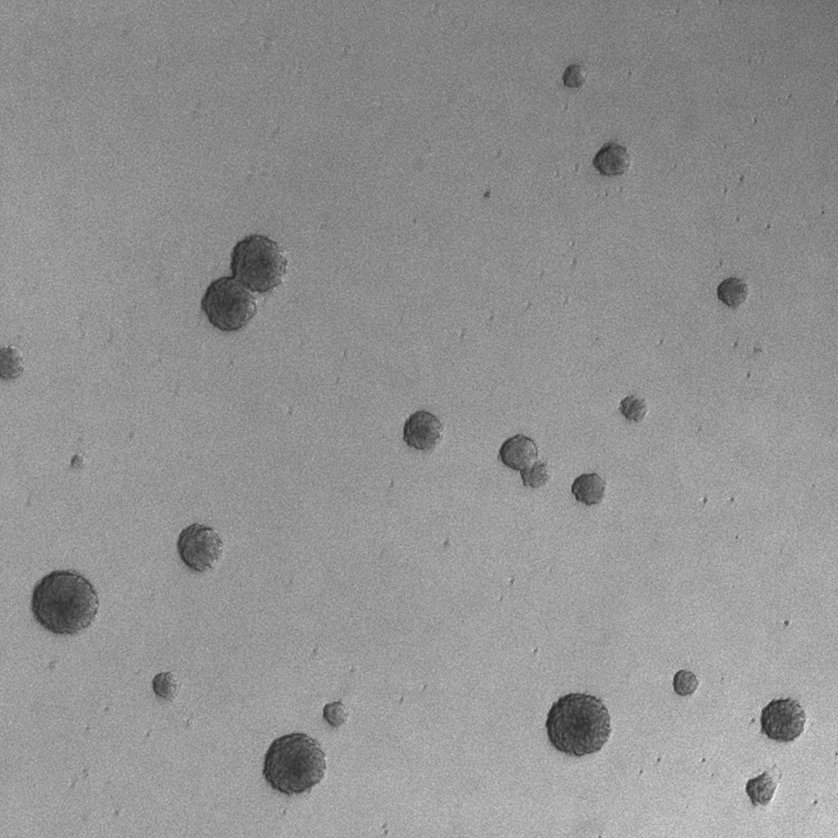

Supplement: Supplementary file 10 — Appendix Figures Source Data [file 44319_2026_768_MOESM10_ESM.zip › Appendix Figures/Appendix Figure S6/S6R/gfp.jpg]

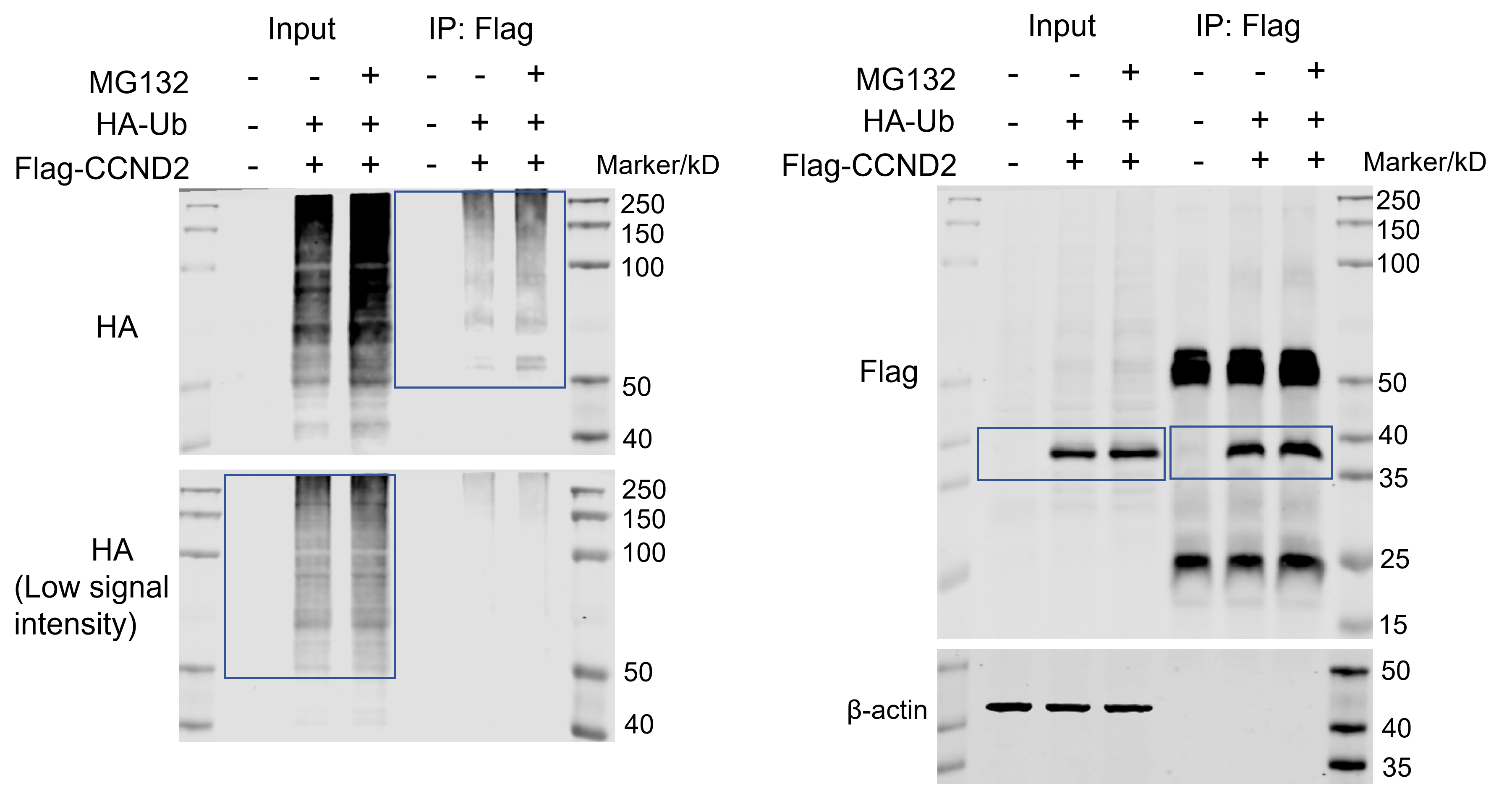

Supplement: Supplementary file 10 — Appendix Figures Source Data [file 44319_2026_768_MOESM10_ESM.zip › Appendix Figures/Appendix Figure S6/S6T/S6T.tif]

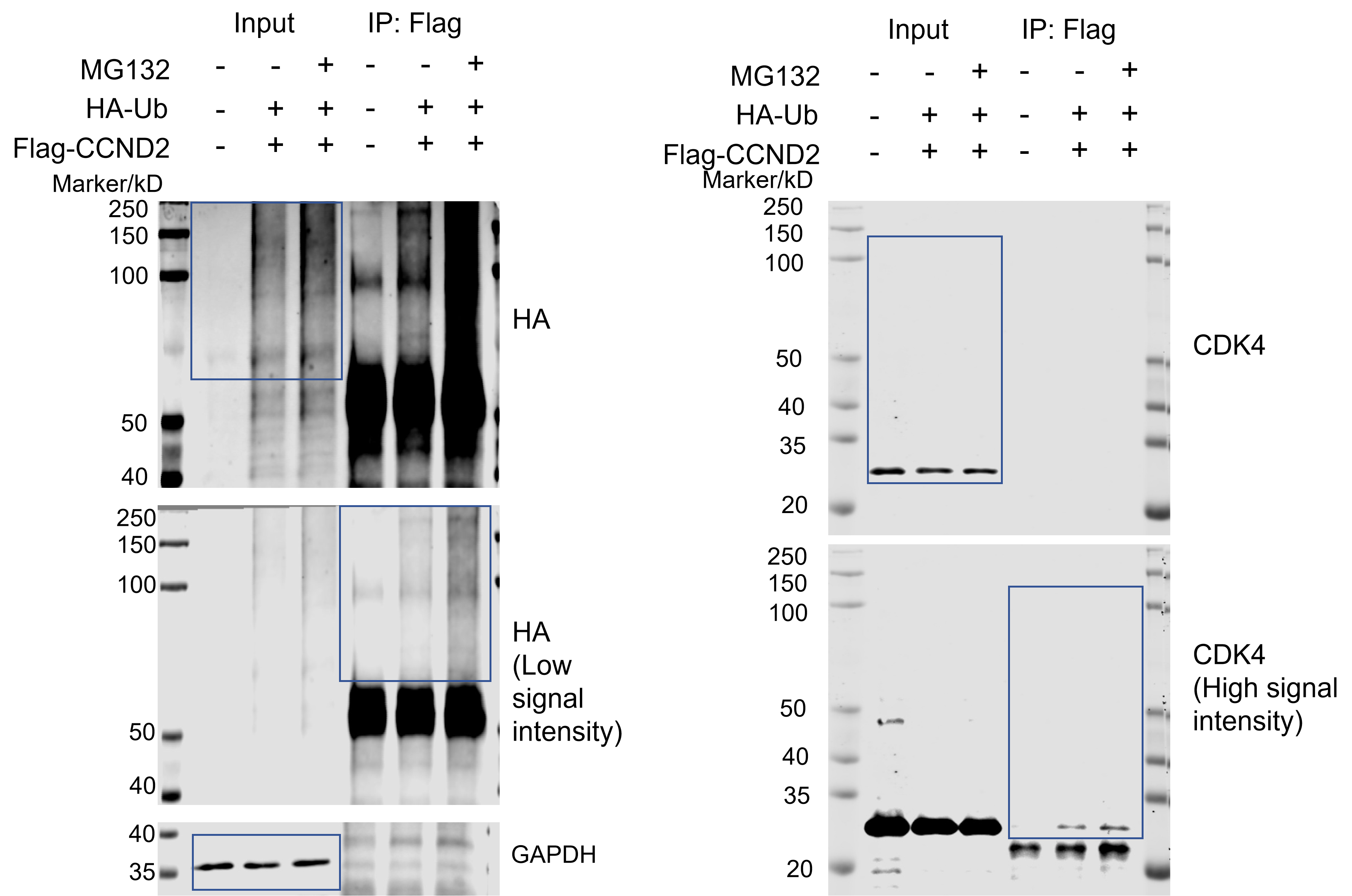

Supplement: Supplementary file 10 — Appendix Figures Source Data [file 44319_2026_768_MOESM10_ESM.zip › Appendix Figures/Appendix Figure S6/S6U/S6T.tif]

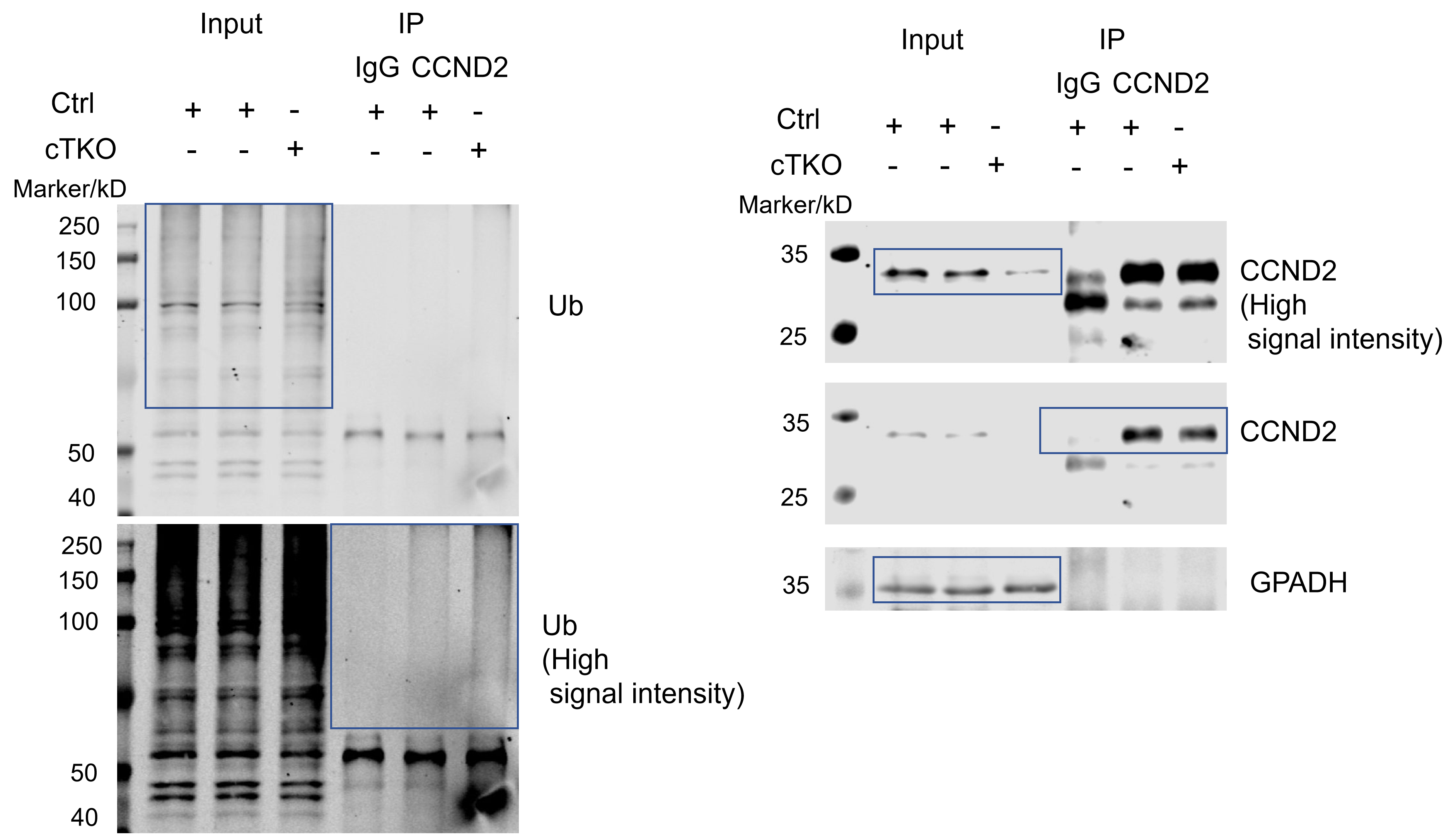

Supplement: Supplementary file 10 — Appendix Figures Source Data [file 44319_2026_768_MOESM10_ESM.zip › Appendix Figures/Appendix Figure S6/S6V/S6U.tif]

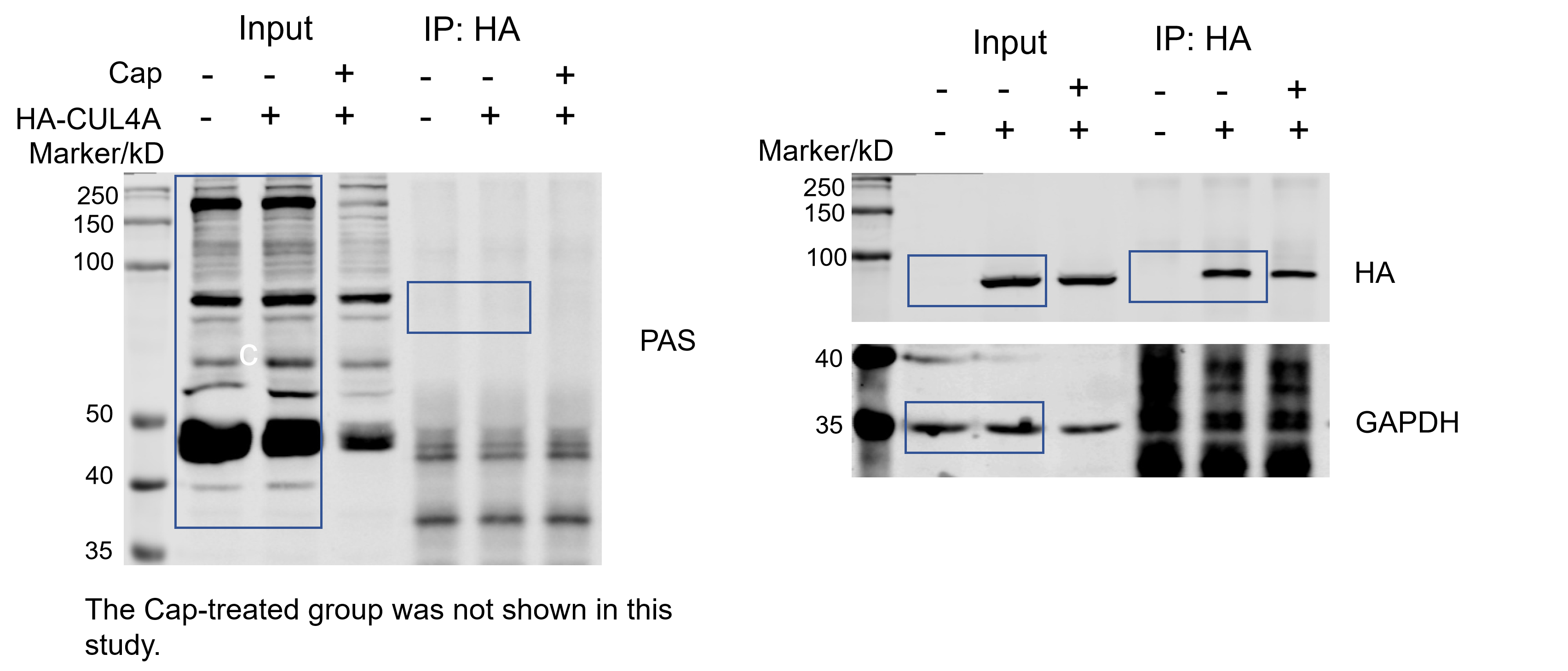

Supplement: Supplementary file 10 — Appendix Figures Source Data [file 44319_2026_768_MOESM10_ESM.zip › Appendix Figures/Appendix Figure S7/S7B/S7B.tif]

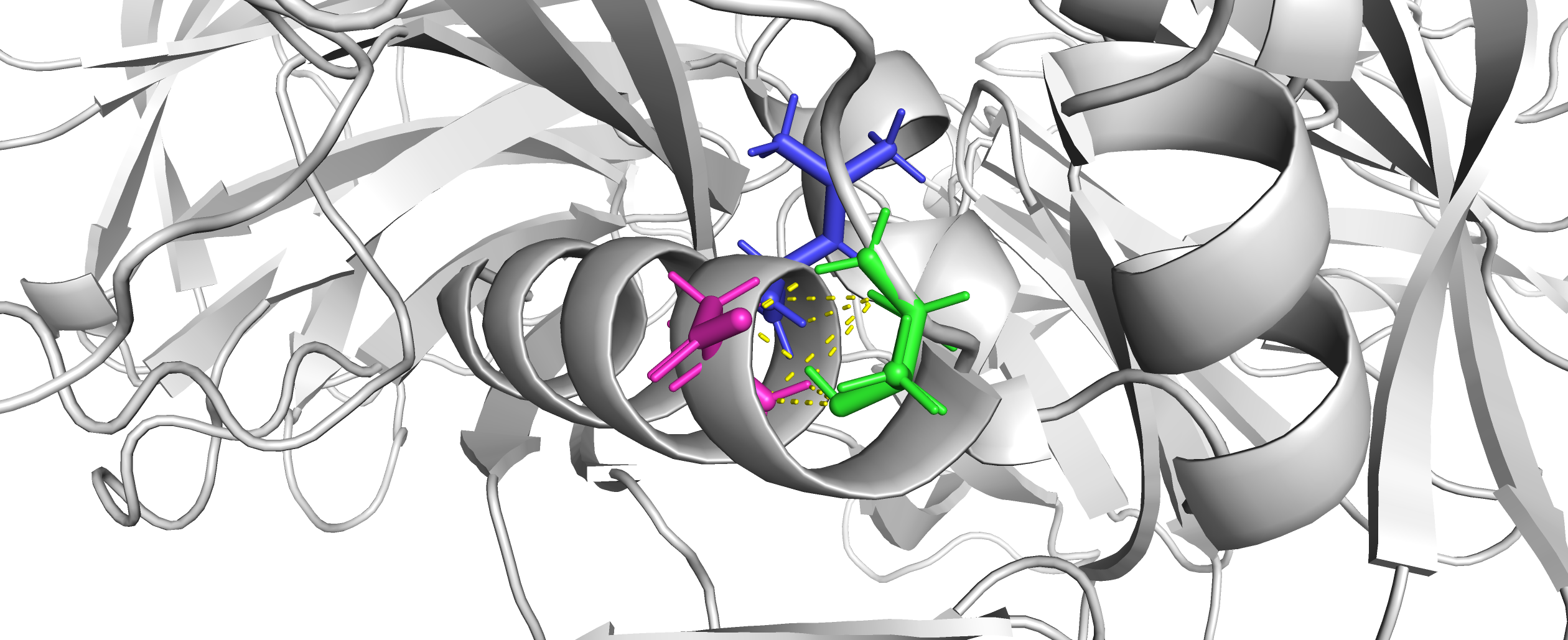

Supplement: Supplementary file 10 — Appendix Figures Source Data [file 44319_2026_768_MOESM10_ESM.zip › Appendix Figures/Appendix Figure S7/S7C/DDB1-enlarged.png]

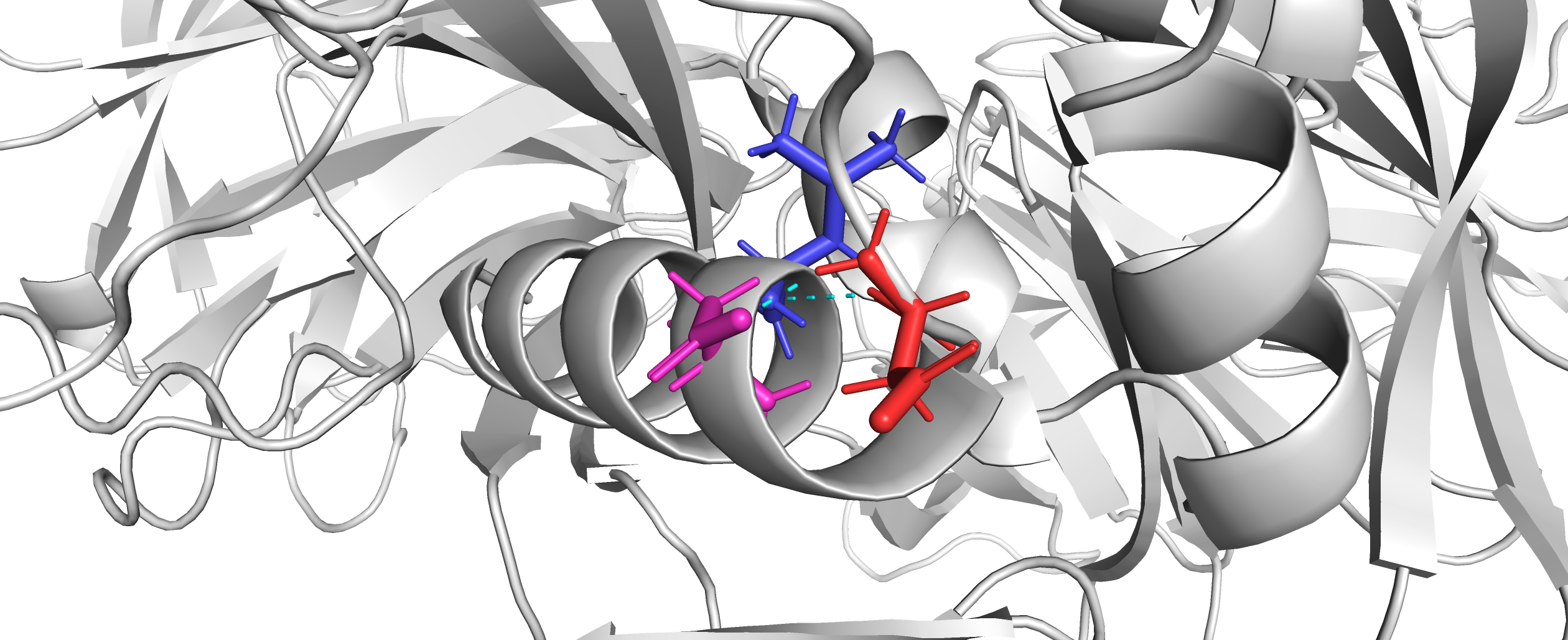

Supplement: Supplementary file 10 — Appendix Figures Source Data [file 44319_2026_768_MOESM10_ESM.zip › Appendix Figures/Appendix Figure S7/S7C/DDB1-T1125D-enlarged.png]

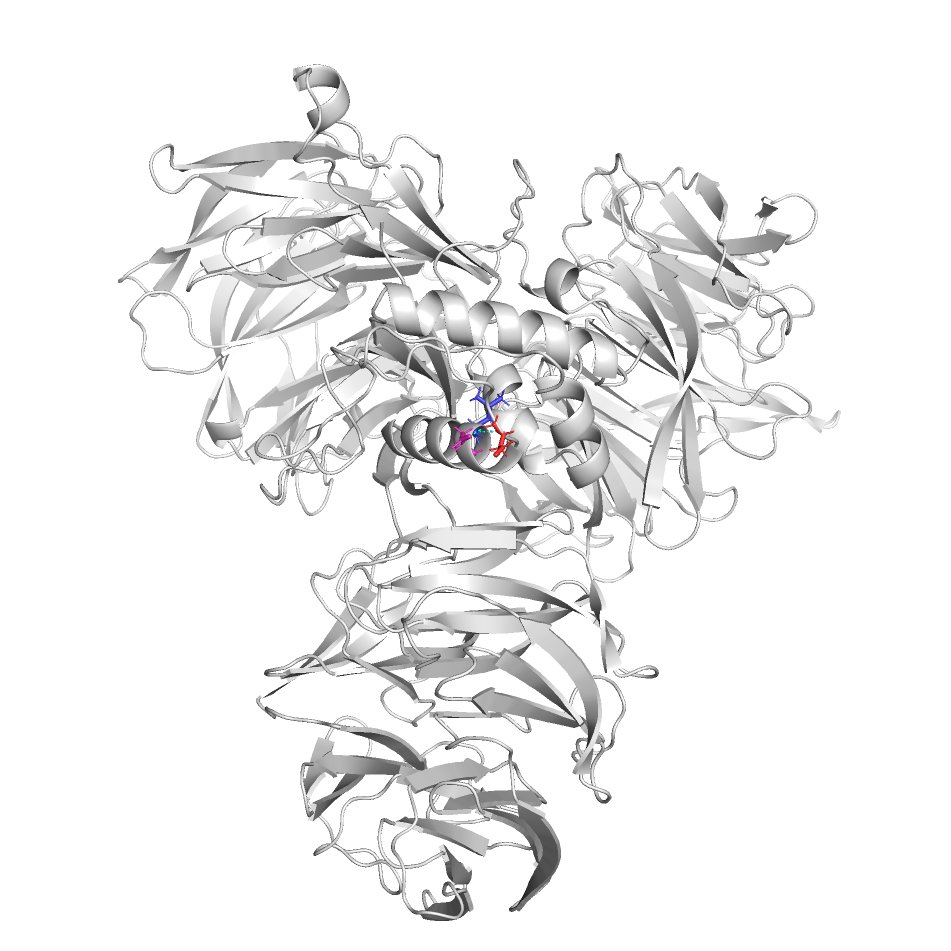

Supplement: Supplementary file 10 — Appendix Figures Source Data [file 44319_2026_768_MOESM10_ESM.zip › Appendix Figures/Appendix Figure S7/S7C/DDB1-T1125D.jpg]

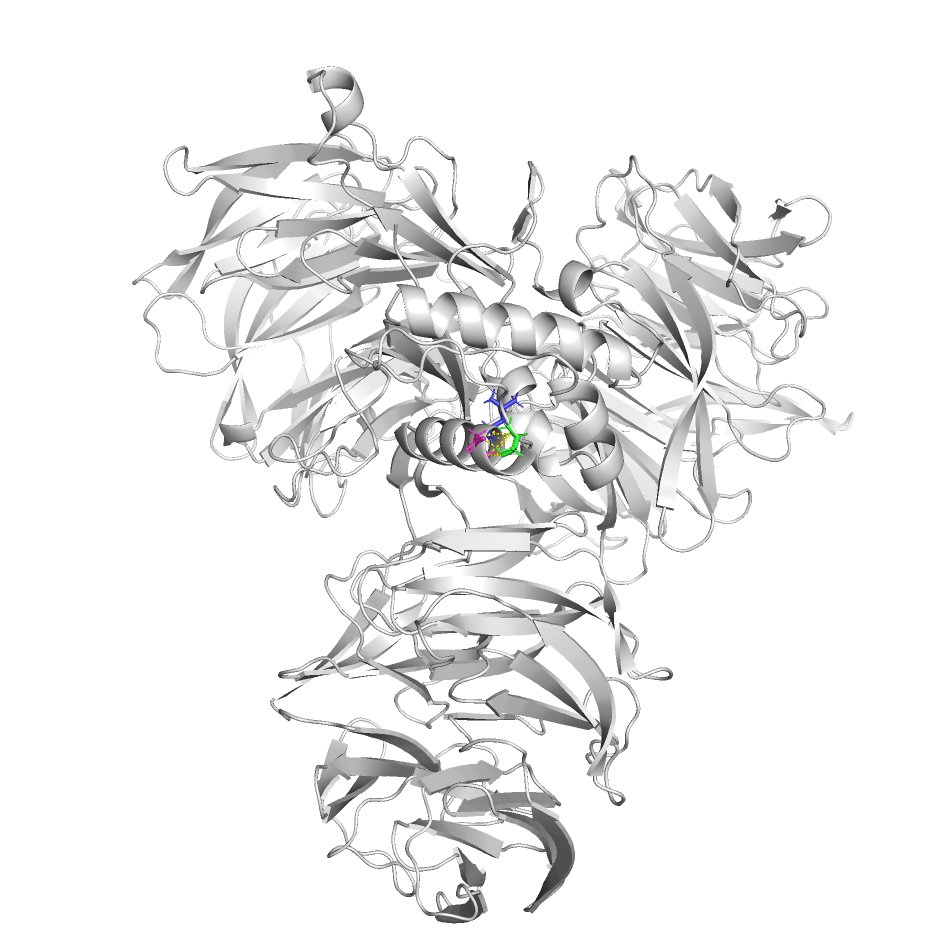

Supplement: Supplementary file 10 — Appendix Figures Source Data [file 44319_2026_768_MOESM10_ESM.zip › Appendix Figures/Appendix Figure S7/S7C/DDB1.jpg]

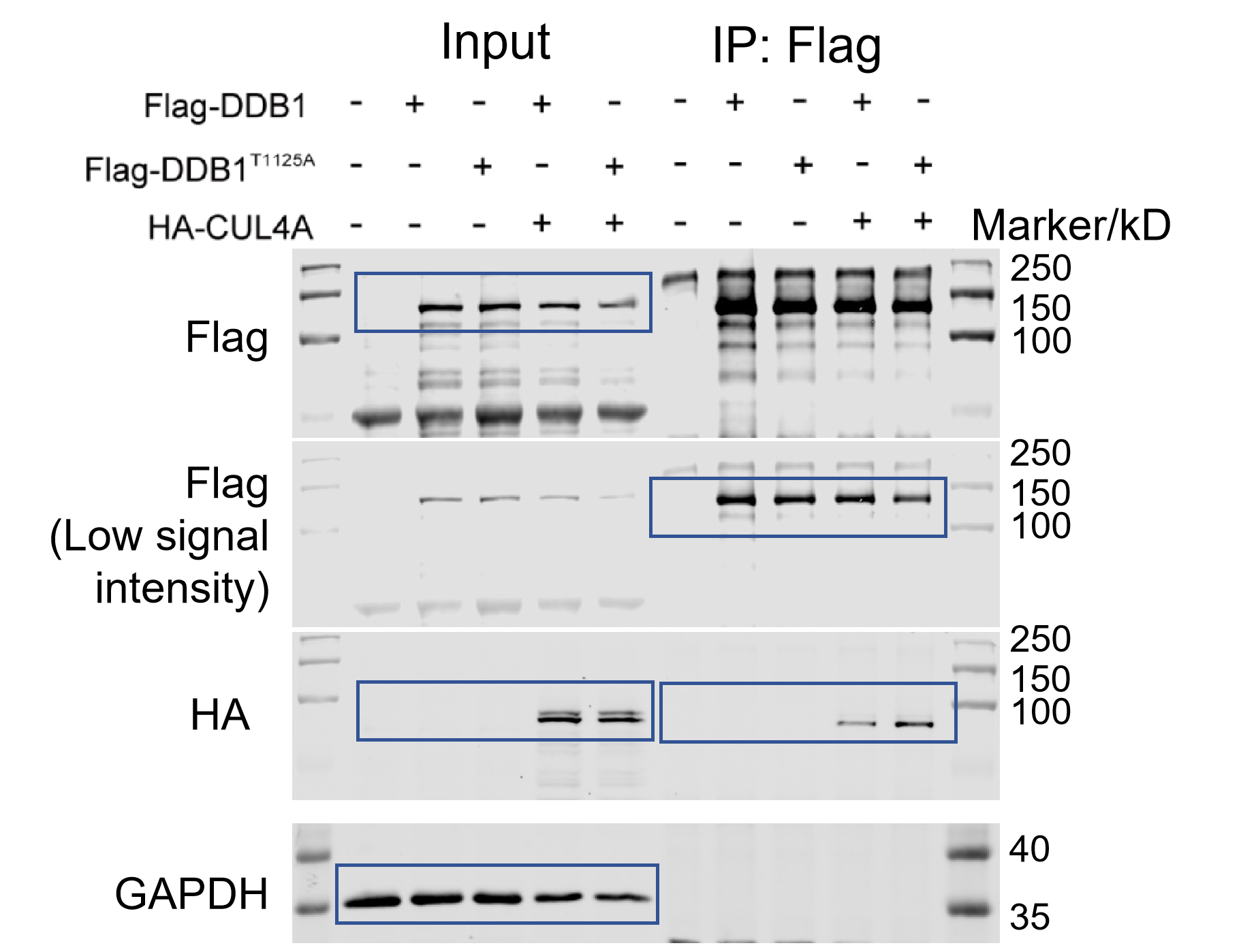

Supplement: Supplementary file 10 — Appendix Figures Source Data [file 44319_2026_768_MOESM10_ESM.zip › Appendix Figures/Appendix Figure S7/S7D/S7D.tif]

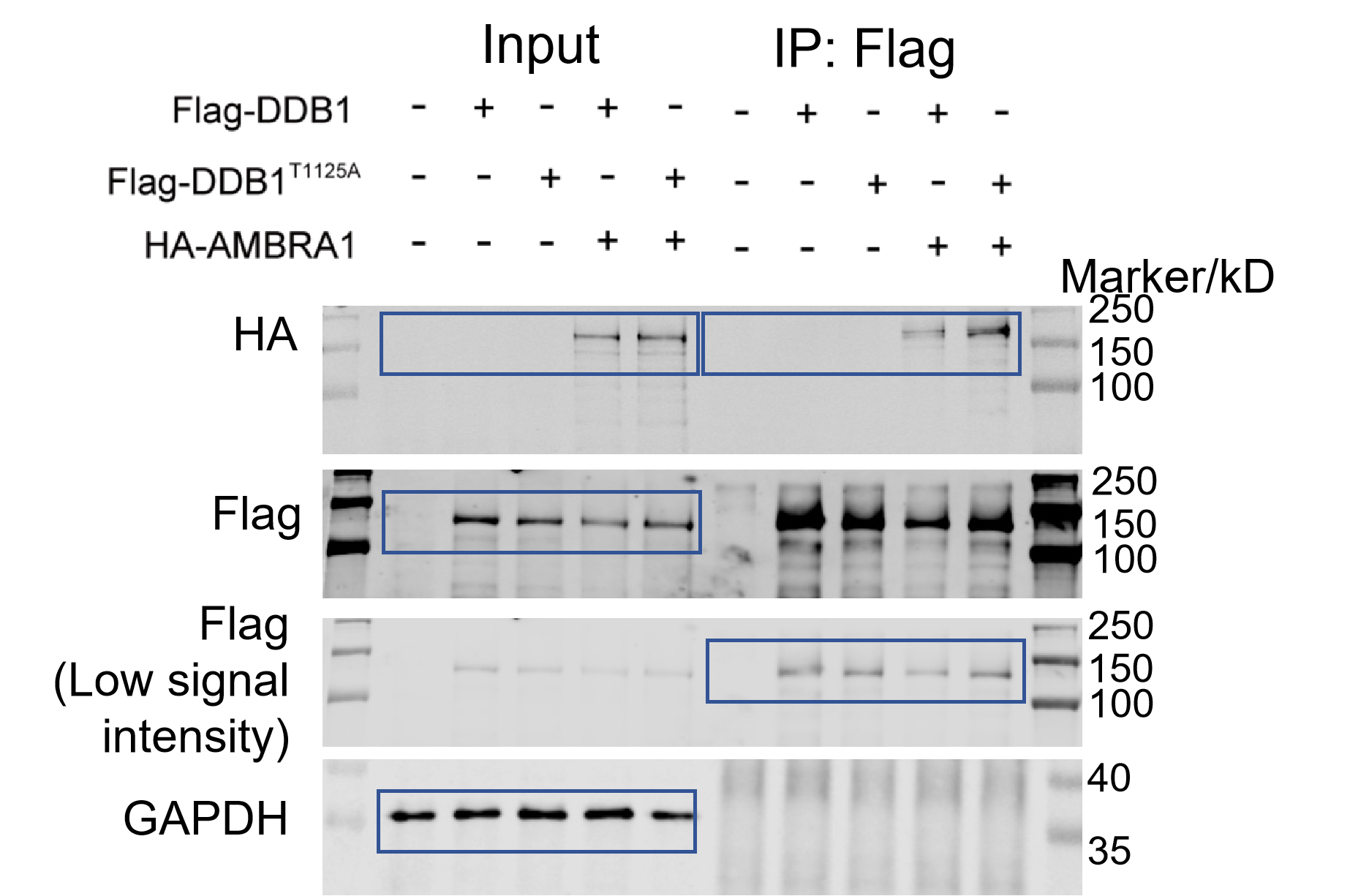

Supplement: Supplementary file 10 — Appendix Figures Source Data [file 44319_2026_768_MOESM10_ESM.zip › Appendix Figures/Appendix Figure S7/S7F/S7F.tif]

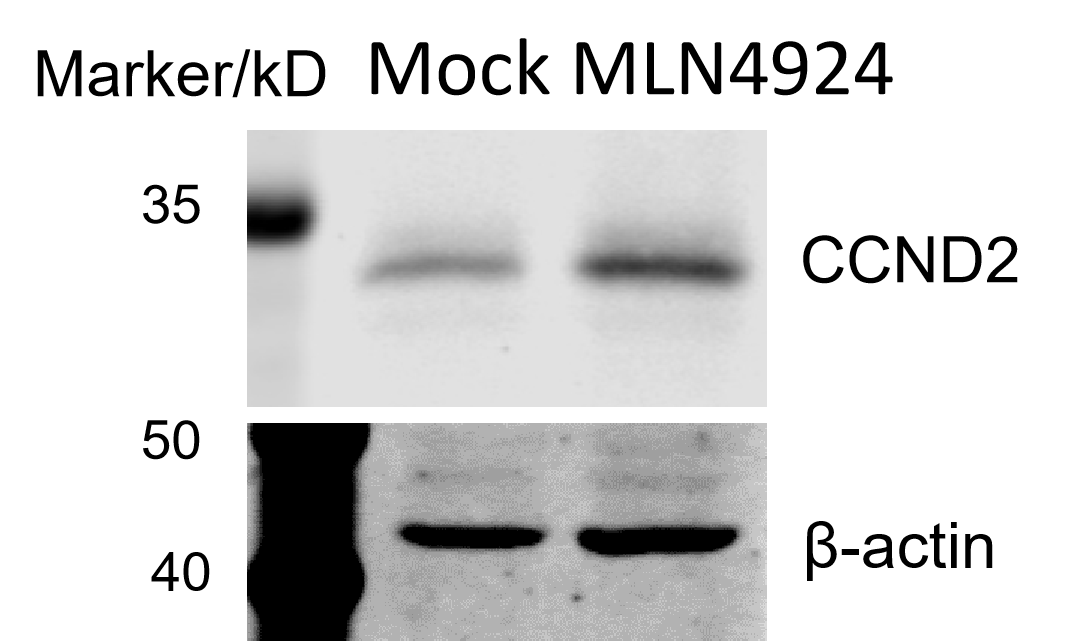

Supplement: Supplementary file 10 — Appendix Figures Source Data [file 44319_2026_768_MOESM10_ESM.zip › Appendix Figures/Appendix Figure S7/S7H/S7H.tif]

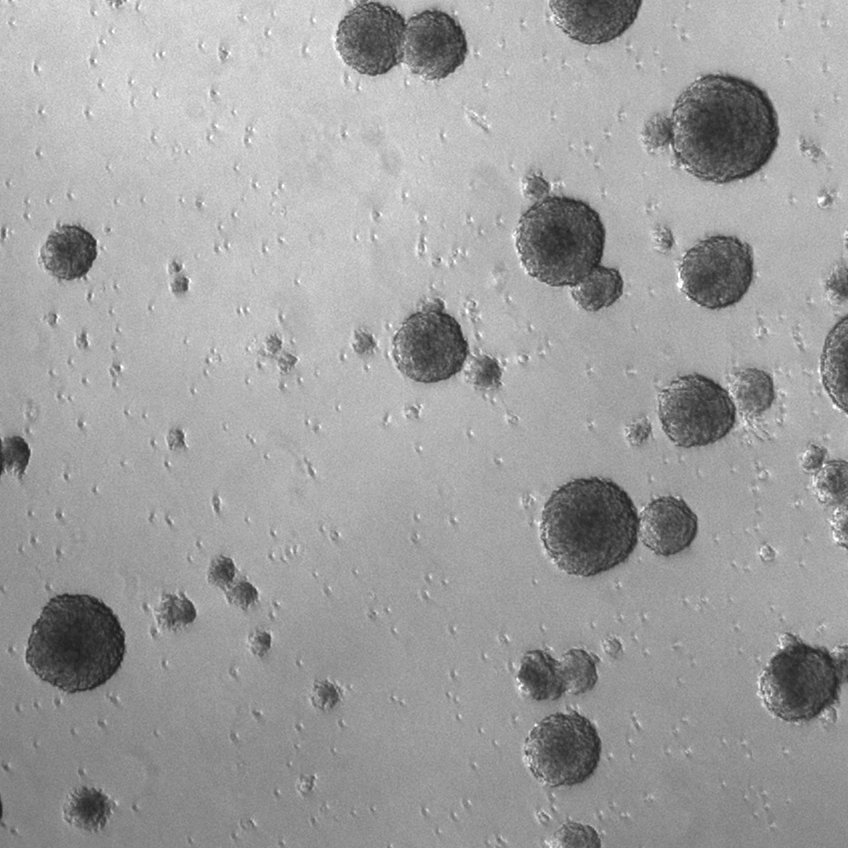

Supplement: Supplementary file 10 — Appendix Figures Source Data [file 44319_2026_768_MOESM10_ESM.zip › Appendix Figures/Appendix Figure S7/S7J/MLN4924.jpg]

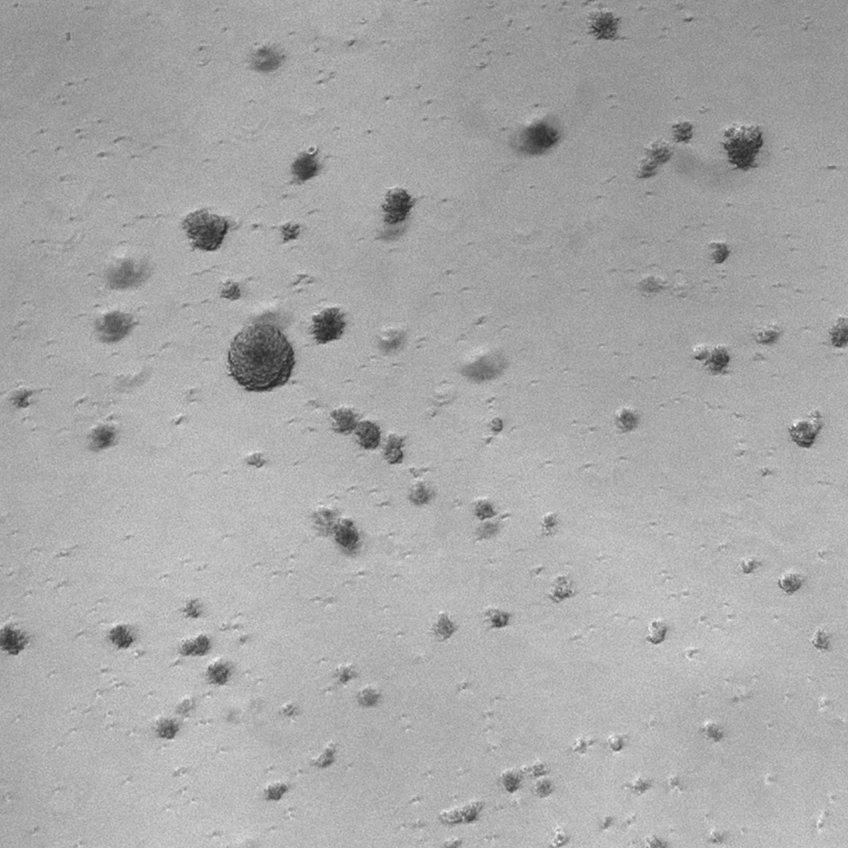

Supplement: Supplementary file 10 — Appendix Figures Source Data [file 44319_2026_768_MOESM10_ESM.zip › Appendix Figures/Appendix Figure S7/S7J/Mock.jpg]

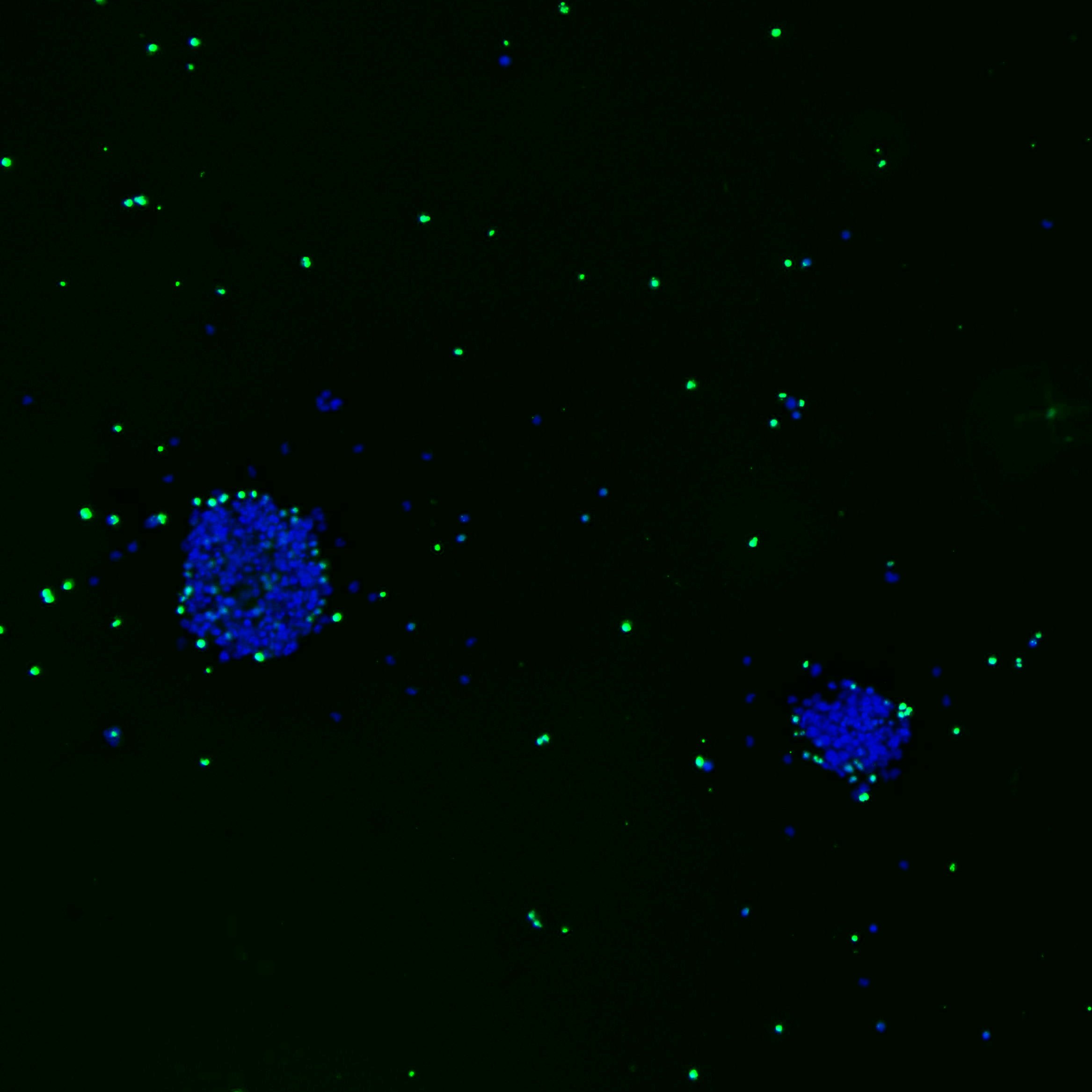

Supplement: Supplementary file 10 — Appendix Figures Source Data [file 44319_2026_768_MOESM10_ESM.zip › Appendix Figures/Appendix Figure S7/S7L/0.1.jpg]

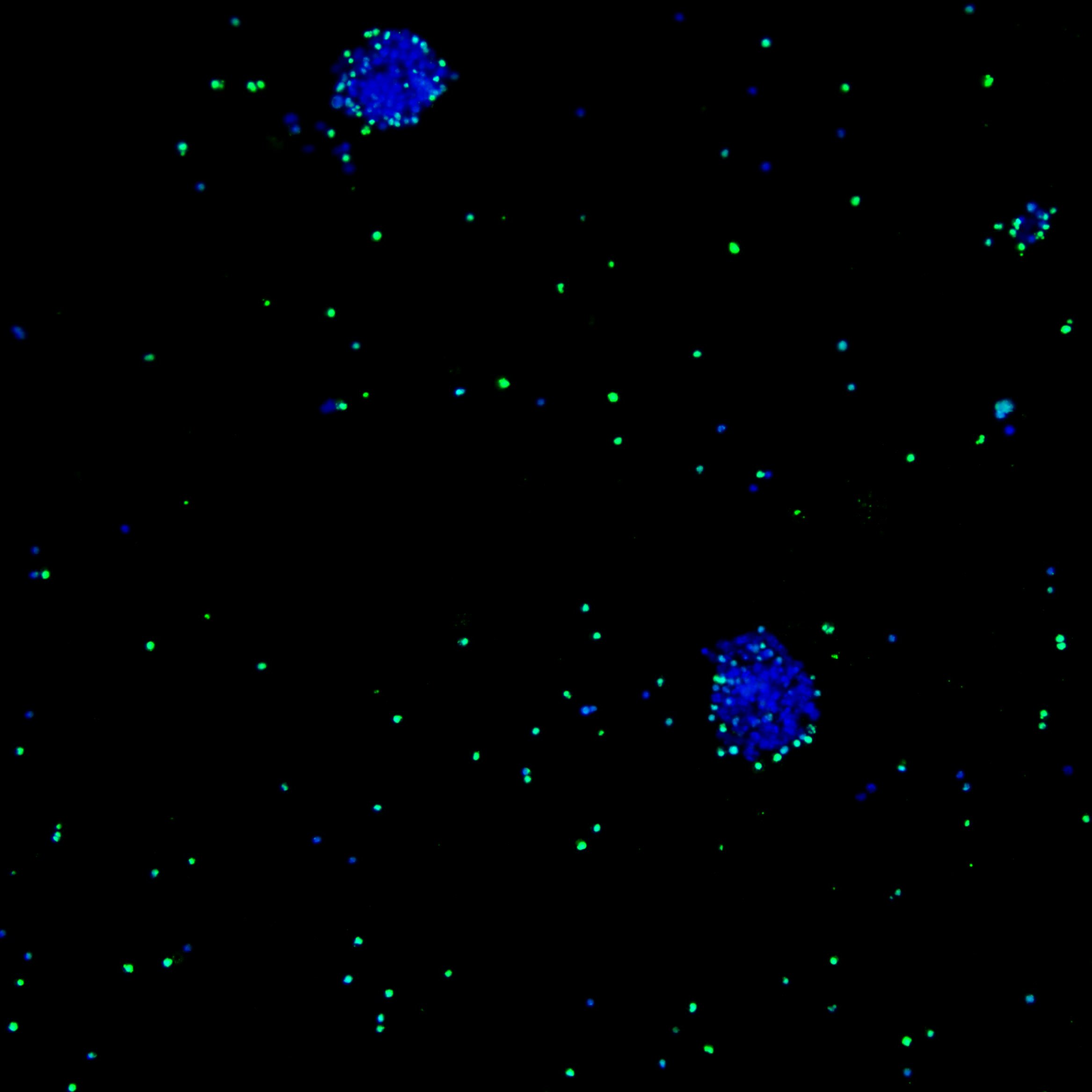

Supplement: Supplementary file 10 — Appendix Figures Source Data [file 44319_2026_768_MOESM10_ESM.zip › Appendix Figures/Appendix Figure S7/S7L/0.jpg]

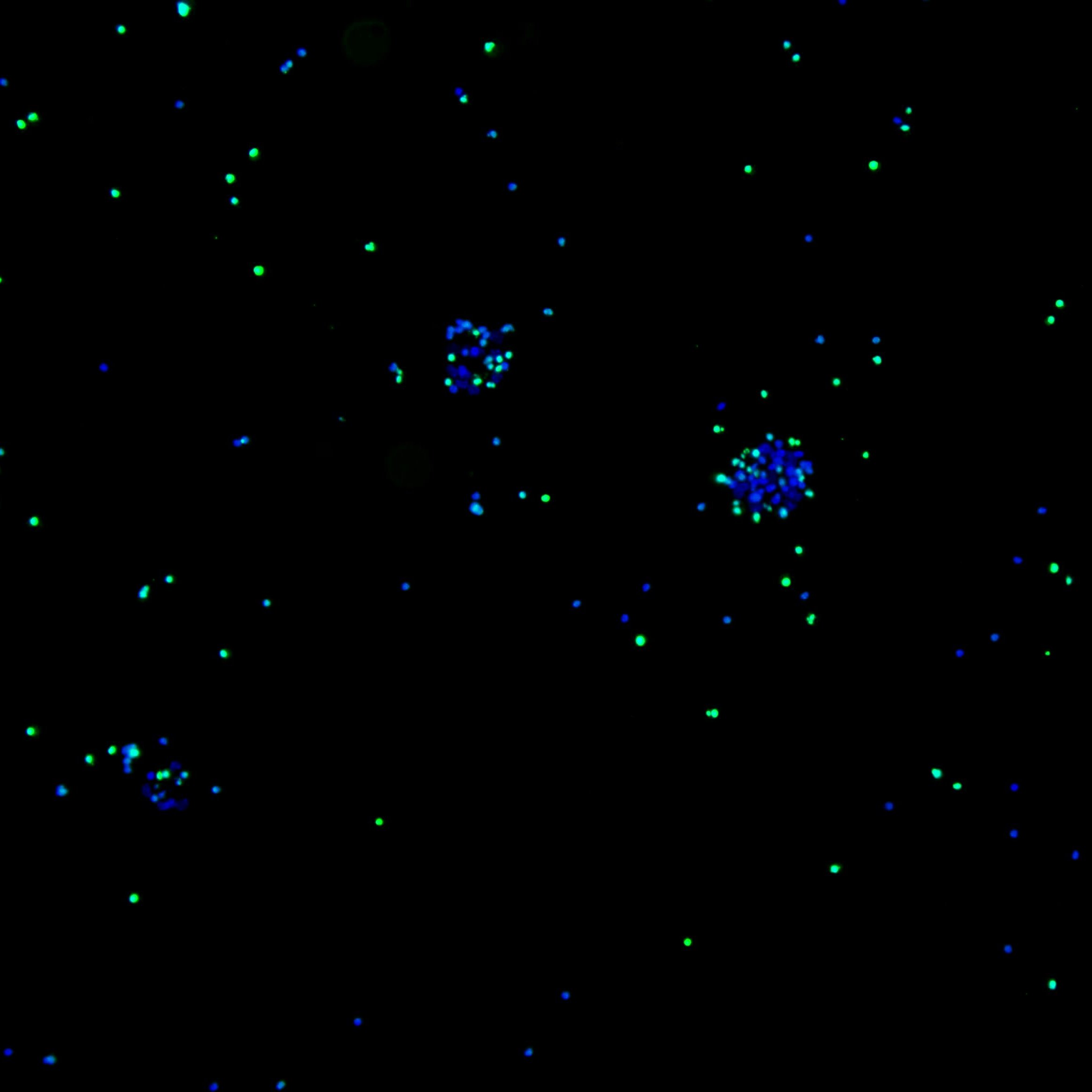

Supplement: Supplementary file 10 — Appendix Figures Source Data [file 44319_2026_768_MOESM10_ESM.zip › Appendix Figures/Appendix Figure S7/S7L/1.jpg]

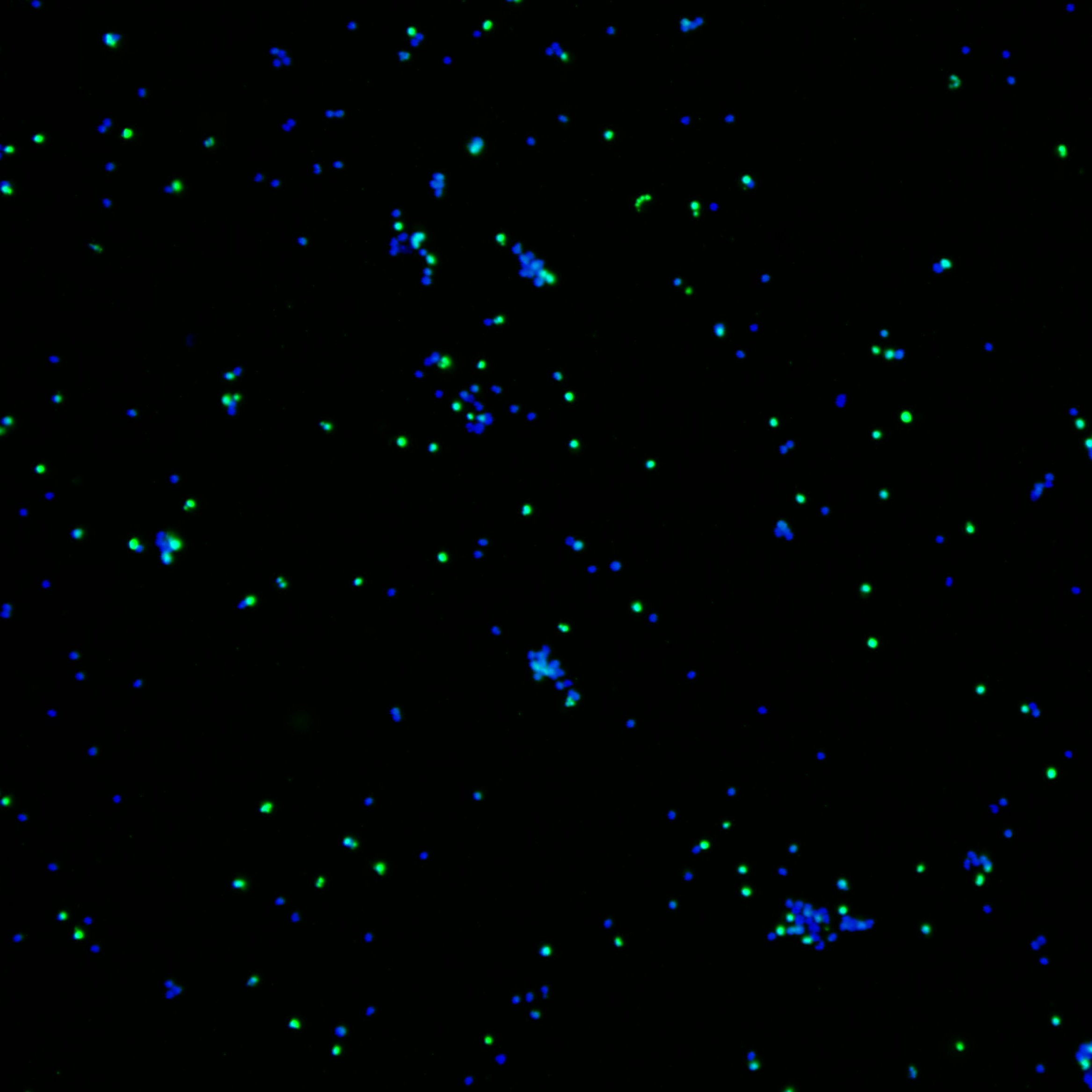

Supplement: Supplementary file 10 — Appendix Figures Source Data [file 44319_2026_768_MOESM10_ESM.zip › Appendix Figures/Appendix Figure S7/S7L/10.jpg]
