# Supplementary material for: CRL4AMBRA1 is a key mediator for AKT-dependent cell cycle control in neural progenitor cells
Source: EMBO Rep. 2026 Apr 27;27(11):3099–119. doi: 10.1038/s44319-026-00768-7 (PMC13260899; doi:10.1038/s44319-026-00768-7)
Supplement: Supplementary file 2 — Peer Review File [file 44319_2026_768_MOESM2_ESM.pdf]

EMBO reports

CRL4AMBRA1 Is a Key Mediator for AKT-dependent Cell Cycle Control in Neural Progenitor Cells

He Wang, Panmiao Liu, Runmin Wang, Hanwen Gu, Tingting Zhu, Guiquan Chen, and Jian-Jun Yang

Corresponding author(s): Guiquan Chen (chenguiquan@nju.edu.cn) , He Wang (wangheriver@zzu.edu.cn), Jian-Jun Yang (yjyangjj@zzu.edu.cn)

Review Timeline:

Submission Date:	1st Sep 25
Editorial Decision:	7th Oct 25
Revision Received:	13th Jan 26
Editorial Decision:	24th Feb 26
Revision Received:	11th Mar 26
Accepted:	26th Mar 26

Editor: Esther Schnapp

Transaction Report:

(Note: With the exception of the correction of typographical or spelling errors that could be a source of ambiguity, letters and reports are not edited. Depending on transfer agreements, referee reports obtained elsewhere may or may not be included in this compilation. Referee reports are anonymous unless the Referee chooses to sign their reports.)

Dear Dr. Chen,

Thank you for the submission of your manuscript to EMBO reports. We have now received the full set of referee reports that is pasted below.

As you will see, the referees acknowledge that the findings are interesting. However, together they also raise some concerns and have several suggestions for how the study should be improved and strengthened. I think all comments are good and should be addressed. Please let me know in case you disagree and we can discuss the exact revision requirements further, also in a video chat, if you like.

I would thus like to invite you to revise your manuscript with the understanding that the referee concerns must be fully addressed and their suggestions taken on board. Please address all referee concerns in a complete point-by-point response. Acceptance of the manuscript will depend on a positive outcome of a second round of review. It is EMBO reports policy to allow a single round of major revision only and acceptance or rejection of the manuscript will therefore depend on the completeness of your responses included in the next, final version of the manuscript.

We realize that it is difficult to revise to a specific deadline. In the interest of protecting the conceptual advance provided by the work, we recommend a revision within 3 months (7th Jan 2026). Please discuss the revision progress ahead of this time with the editor if you require more time to complete the revisions.

IMPORTANT NOTE: we perform an initial quality control of all revised manuscripts before re-review. Your manuscript will FAIL this control and the handling will be DELAYED if the following APPLIES:
1) A data availability section providing access to data deposited in public databases is missing. If you have not deposited any data, please add a sentence to the data availability section that explains that.
2) Your manuscript contains statistics and error bars based on n=2. Please use scatter blots in these cases. No statistics should be calculated if n=2.

When submitting your revised manuscript, please carefully review the instructions that follow below. Failure to include requested items will delay the evaluation of your revision.

1) a .docx formatted version of the manuscript text (including legends for main figures, EV figures and tables). Please make sure that the changes are highlighted to be clearly visible.

2) individual production quality figure files as .eps, .tif, .jpg (one file per figure). See https://wol-prod-cdn.literatumonline.com/pb-assets/embo-site/EMBOPress_Figure_Guidelines_061115-1561436025777.pdf for more info on how to prepare your figures.

3) We replaced Supplementary Information with Expanded View (EV) Figures and Tables that are collapsible/expandable online. A maximum of 5 EV Figures can be typeset. EV Figures should be cited as 'Figure EV1, Figure EV2" etc... in the text and their respective legends should be included in the main text after the legends of regular figures.

- For the figures that you do NOT wish to display as Expanded View figures, they should be bundled together with their legends in a single PDF file called *Appendix*, which should start with a short Table of Content. Appendix figures should be referred to in the main text as: "Appendix Figure S1, Appendix Figure S2" etc. See detailed instructions regarding expanded view here: <https://www.embopress.org/page/journal/14693178/authorguide#expandedview>

- Additional Tables/Datasets should be labeled and referred to as Table EV1, Dataset EV1, etc. Legends have to be provided in a separate tab in case of .xls files. Alternatively, the legend can be supplied as a separate text file (README) and zipped together with the Table/Dataset file.

4) a .docx formatted letter INCLUDING the reviewers' reports and your detailed point-by-point responses to their comments. As part of the EMBO Press transparent editorial process, the point-by-point response is part of the Review Process File (RPF), which will be published alongside your paper.

5) a complete author checklist, which you can download from our author guidelines <https://www.embopress.org/page/journal/14693178/authorguide>. Please insert information in the checklist that is also reflected in the manuscript. The completed author checklist will also be part of the RPF.

6) Please note that all corresponding authors are required to supply an ORCID ID for their name upon submission of a revised manuscript (<https://orcid.org/>). Please find instructions on how to link your ORCID ID to your account in our manuscript tracking system in our Author guidelines <https://www.embopress.org/page/journal/14693178/authorguide#authorshipguidelines>

7) Before submitting your revision, primary datasets produced in this study need to be deposited in an appropriate public database (see https://www.embopress.org/page/journal/14693178/authorguide#datadeposition). Please remember to provide a reviewer password if the datasets are not yet public. The accession numbers and database should be listed in a formal "Data Availability" section placed after Materials & Method (see also https://www.embopress.org/page/journal/14693178/authorguide#datadeposition). Please note that the Data Availability Section is restricted to new primary data that are part of this study. * Note - All links should resolve to a page where the data can be accessed. *
If your study has not produced novel datasets, please mention this fact in the Data Availability Section.

8) At EMBO Press we ask authors to provide source data for the main manuscript figures. You will receive a separate email with instructions for providing source data with your revised manuscript, including how to upload and organize the files.

9) Our journal also encourages inclusion of *data citations in the reference list* to directly cite datasets that were re-used and obtained from public databases. Data citations in the article text are distinct from normal bibliographical citations and should directly link to the database records from which the data can be accessed. In the main text, data citations are formatted as follows: "Data ref: Smith et al, 2001" or "Data ref: NCBI Sequence Read Archive PRJNA342805, 2017". In the Reference list, data citations must be labeled with "[DATASET]". A data reference must provide the database name, accession number/identifiers and a resolvable link to the landing page from which the data can be accessed at the end of the reference. Further instructions are available at https://www.embopress.org/page/journal/14693178/authorguide#referencesformat

10) Regarding data quantification (see Figure Legends: https://www.embopress.org/page/journal/14693178/authorguide#figureformat)

The following points must be specified in each figure legend:

- the name of the statistical test used to generate error bars and P values,

- the number (n) of independent experiments (please specify technical or biological replicates) underlying each data point,

- the nature of the bars and error bars (s.d., s.e.m.),

- If the data are obtained from n Program fragment delivered error ``Can't locate object method "less" via package "than" (perhaps you forgot to load "than"?) at //ejpvfs23/sites23b/embor_www/letters/embor_decision_revise_and_review.txt line 56.' 2, use scatter blots showing the individual data points.

Discussion of statistical methodology can be reported in the materials and methods section, but figure legends should contain a basic description of n, P and the test applied.

- Please also include scale bars in all microscopy images.

11) The journal requires a statement specifying whether or not authors have competing interests (defined as all potential or actual interests that could be perceived to influence the presentation or interpretation of an article). In case of competing interests, this must be specified in your disclosure statement. Further information: https://www.embopress.org/competing-interests

12) All Materials and Methods need to be described in the main text using our 'Structured Methods' format, which is required for all research articles. According to this format, the Methods section includes a separate Reagents and Tools Table file (listing key reagents, experimental models, software and relevant equipment and including their sources and relevant identifiers) and a Methods and Protocols section describing the methods using a step-by-step protocol format. The aim is to facilitate adoption of the methodologies across labs. More information on how to adhere to this format as well as a downloadable template (.docx) for the Reagents and Tools Table can be found in our author guidelines: https://www.embopress.org/page/journal/14693178/authorguide#structuredmethods.

An example of a Method paper with Structured Methods can be found here: https://www.embopress.org/doi/full/10.1038/s44320-024-00037-6#sec-4

We would also welcome the submission of cover suggestions, or motifs to be used by our Graphics Illustrator in designing a cover.

As part of the EMBO publication's Transparent Editorial Process, EMBO reports publishes online a Review Process File (RPF) to accompany accepted manuscripts. This File will be published in conjunction with your paper and will include the referee reports, your point-by-point response and all pertinent correspondence relating to the manuscript.

You are able to opt out of this by letting the editorial office know (emboreports@embo.org). If you do opt out, the Review

Process File link will point to the following statement: "No Review Process File is available with this article, as the authors have chosen not to make the review process public in this case."

I look forward to seeing a revised form of your manuscript when it is ready.

Best regards,
Esther

Esther Schnapp, PhD
Senior Editor
EMBO reports

Referee #1:

This study shows that AKT is essential for neural progenitor cell (NPC) proliferation and cortical development. Using human and mouse NPCs together with knockout models, the authors demonstrate that AKT loss prolongs G1, reduces NPC numbers, and causes microcephaly. Mechanistically, AKT phosphorylates DDB1 to inhibit CCND2 degradation by the CRL4AMBRA1 complex, thereby sustaining NPC proliferation. These findings identify an AKT-DDB1-CCND2 axis as a key regulator of cortical expansion and a potential mechanism underlying AKT-related brain malformations.
The manuscript is well written, easy to follow, and presents high-quality data in the first half, particularly in Figures 1-2 and 5. However, the mechanistic section is less convincing and leaves the paper feeling unfinished. Several major concerns remain:

1. The authors never address why cyclin D1 does not appear to fall under AKT regulation, unlike cyclins D2 and D3. This is crucial, since previous studies have shown that CRL4AMBRA1 regulates the half-life of all three D-type cyclins.
2. CCND3, despite being affected by AKT, is dropped from most downstream experiments without justification, and CCND1 is not addressed at all. This weakens the mechanistic narrative. The author should include CCND1 and CCND3 in the experiments shown in Fig. 6 and Fig. 7.
3. Ubiquitination assays (Fig. 6) were performed under non-denaturing conditions. The authors need to repeat these to ensure that they are not probing the ubiquitination of other cyclin interactors. Apart from MG132 and an AKT inhibitor as control they should also use AKT TKO controls.
4. The TUNEL assay (Fig. 3) shows unusually high variability compared to otherwise consistent datasets and should be repeated.
5. Comparisons of AKT binding to CRL4AMBRA1 complex components were performed on separate membranes, raising concerns about the robustness of these findings. Authors should load all the IP-s onto one membrane with comparable amounts of baits to asses the differential binding on these.
6. While the authors demonstrate applicability of their findings in 293T cells, they never discuss how these results might extend to cancer biology, where the regulation of D-type cyclins is highly relevant.
7. The rescue experiments with MLN4924 are questionable. This inhibitor blocks all CRL mediated degradation in the cells which is toxic after an extended period of time (24 hours). How were the author able to see rescue with something that is so broad in specificity? I'm surprised they even saw cell proliferation at all. Even if the MLN4924 was just used at 0.1 uM, treating the cells with it for days should cause apoptosis. More robust rescue experiments are needed to prove the authors mechanistic claims.
In summary, while the descriptive and correlative data are strong, the mechanistic claims lack rigor and sufficient experimental support. Addressing the role of cyclin D1, including CCND1/3 in downstream analyses, providing more robust ubiquitination and rescue experiments, and discussing the broader implications in cancer would considerably strengthen the manuscript.

Referee #2:

This manuscript demonstrates a crucial role for AKT in regulating the G1/S transition in NPCs via a novel post-translational mechanism. The authors provide in vivo and in vitro evidence, moving logically from phenotypic characterization to mechanistic insight. The rescue experiments with both pharmacological inhibition and genetic overexpression of CCND2 are particularly compelling. The findings have important implications for understanding cortical development and the etiology of certain malformations of cortical development (MCDs). The major and minor points were listed as follows:

Major points：
1.Mechanistic specificity of DDB1 phosphorylation need to be further addressed. While the data strongly suggest that AKT-mediated phosphorylation of DDB1 at T1125 disrupts its interaction with CCND2, the exact mechanism remains somewhat speculative. Does this phosphorylation induce a conformational change in DDB1? Does it affect DDB1's interaction with other components of the CRL4 complex, like CUL4A or AMBRA1?
2.Functional rescue with phospho-mimetic DDB1 is essential but lacking. The study shows that the phospho-deficient mutant (T1125A) binds more CCND2. A critical functional test would be to see if a phospho-mimetic mutant (e.g., T1125D/E) can rescue

the phenotypes in AKT-deficient NPCs.
3.The WB data (Fig. 6A, B, S6B, C) show that both CCND2 and CCND3 protein levels are reduced in Akt cTKO cortices. However, the authors focus exclusively on CCND2, justified by its established role in cortical development. The manuscript would be strengthened by addressing whether CCND3 is also a substrate of the same AKT-CRL4AMBRA1 axis.
4.Clarity on CRL4AMBRA1 complex specificity need to be addressed. The title and text refer to "CRL4AMBRA1" but the interaction data (Fig. 7A, S7A-C) show that AKT binds CUL4A and DDB1, but not CUL4B or AMBRA1. This is an important distinction, as it suggests the specific involvement of the CUL4A-containing complex.

Minor points:
1.Some co-IP results (e.g., Fig. 7A, E, P, Q) are presented as representative blots without quantification. Including quantitative data from multiple replicates (as was done in Fig. 7F) for these key experiments would bolster the statistical rigor.
2.Inhibitor specificity issue: while MK2206 and Capivasertib are well-established AKT inhibitors, a brief mention in the Methods or Results about their specific targets (allosteric vs. ATP-competitive) and the justification for using both would be helpful for readers.
3.In Fig. 1K, L: the description of the CFSE assay is slightly confusing. It states that Akt cTKO has a "greater proportion of high CFSE-intensity cells," which is correct (less dilution due to less proliferation). However, the graph in L is gated on cells with low CFSE intensity. The legend should be clarified to avoid misinterpretation.

Referee #3:

The manuscript by Wang et al provides new insights into a long-standing question on the molecular mechanisms controlling G1 phase progression in cortical development, by addressing the role of AKT in the regulation of mouse neural progenitor cell (NPCs) proliferation.
The authors performed a series of experiments on the developing mouse cortex, and used in vitro models (HEK293T cells, mouse neurospheres and human NPC), to dissect the molecular mechanisms involved in this regulation.
They first show that AKT is highly expressed in mouse NPCs, both in vivo and in vitro, and that gain-of-function mutations of AKT increase neurosphere size.
The authors then examine the effects of AKT loss in NPCs, demonstrating that mice with triple knockout (cTKO) of Akt1/2/3 in NPCs exhibit a thinner cortex, reduced cortical progenitor proliferation, and altered G1 phase progression. Similarly, pharmacological inhibition of AKT decreases neurosphere size; neurospheres isolated from cTKO mice are also smaller than controls, and display reduced proliferation.
These findings are complemented by RNAseq analysis of the cTKO cortices, which provides supportive evidence for deregulated cell-cycle progression, including G1 phase.
As Akt cTKO NPCs exhibit reduced levels of the G1-phase regulator Cyclin D2 (CCND2), the authors further dissect the link between AKT and CCND2 in vitro in HEK296T cells. They show that AKT activity stabilizes CCND2, via inhibition of the ubiquitine-proteasome pathway.
Finally, they return to mouse NPCs and perform rescue experiments in cTKO neurospheres that indicate that altered proliferation of cTKO NPCs is due, at least in part, to reduced levels of CCND2.

General comments:
Overall, the data are clearly presented and the work provides a mechanistic link between AKT signaling and CCND2 stability, to control the cell-cycle in NPCs.

However, the general conclusion heavily relies on the in vivo data and the analysis of cTKO mice. The methods implemented for the in vivo quantifications must thus be clarified/corrected. Specifically, quantifications of PAX6+, TBR2+, Sox2+, BrdU+, pH3+, and Tunnel+ cells need to be improved (detailed below).
The choice of HEK293T cells for mechanistic dissection, although experimentally convenient, raises concerns about physiological relevance and it would be interesting to show that the same regulatory mechanism (at least some aspects) is conserved in NPCs themselves.

The authors omitted to quote relevant work related to the role of G1 phase and cyclins in cell-cycle regulation of cortical progenitors (eg Pilaz et al. PNAS, 2009 and other studies). This should be corrected.

Specific points:

Consequences of AKT GOF in NPCs
The authors show that expression of the "p.T288I mutant significantly increased neurosphere size compared to WT AKT3 controls (Fig. 1, G and H, and fig. S1D), demonstrating enhanced NPC proliferation" (Lines 105-106).
-They should show the level of AKT3 in control neurospheres (neurospheres non transfected, and neurospheres transfected with AKT3) and in neurospheres expressing the mutant T288I.
-They should analyse the proliferation rate of NPCs in T288I mutant neurospheres vs AKT3 controls (eg, %Ki67+ cells).

Pharmalogical AKT inhibition

Page 4 line 99: Treatment of mouse neurospheres with two selective AKT inhibitors "caused a marked reduction in neurosphere size compared to mock-treated controls". The authors conclude that it indicates "suppressed NPC proliferation upon AKT inhibition".

- The authors need to analyse cell proliferation in neurospheres treated with the inhibitors vs controls (eg FACS analysis).
- Decreased neurosphere size could be also be due to cell death. Therefore, authors should also analyse cell death.

Consequences of AKT inhibition using the triple knock-out mice:

-The authors quantified multiple parameters (Figs 2 and 3) (including number of PAX6+, TBR2+ per section; BrdU+ cells following a BrdU pulse; pH3+ cells, and apoptotic cells by TUNNEL staining...). They conclude that AKT loss affects NPC proliferation rather than survival.
This conclusion depends critically on the method used for the quantifications.
It is not clear how the authors determined the size of the sections (the squares indicated by dotted lines?).
Reporting absolute cell counts per section is not rigorous nor accurate, because counts vary with local cell density. The authors should report the proportion of positive cells among the total (DAPI+) cells.
In Figure S3, they quantified the "area of TUJ1+ or DCX+ cells/103 µm2". What does "area of TUJ1+"? mean?
All the above mentionned quantifications need to be reanalyzed using appropriate methods, as the validity of the in vivo results-and consequently a substantial part of the conclusions-fully depends on this.

Cell cycle and INM in cTKO

"At 12 hours post-injection, significantly fewer BrdU+ cells were detected in the basal VZ (R2) of Akt cTKO cortices, suggesting delayed cell cycle progression (Fig. 5, E to G, and fig. S5, C and D)." (Page 7. Lines 183-185).
The authors should explain this result more clearly. The data suggests altered, rather than delayed, cell cycle progression.
To precisely define the R1 and R2 regions, the authors need to provide provide PAX6/TBR2/BrdU co-stainings images for both Control and cTKO.

"A 24-hour BrdU labeling experiment further revealed that fewer Akt cTKO NPCs completed second mitotic cycle compared to controls (Fig. 5, H and I, and fig. S5E)". (Page 7. Lines 185-187).
It is not clear why they deduce that "NPCs completed second mitotic cycle compared to controls". The authors need to clarify this point.

Investigating the impact of AKT KO on INM requires a more detailed analysis (ie, the position of G1, S, G2 and M cells within the thickness of the VZ).

Minor points:

-The description of the mutant mice (lines 124-130): should be moved upper (mixed with lines 108-111).
line 86-87: The title is not clear. "AKT promotes NPC proliferation: enhanced by gain-of-function mutations and inhibited by pharmacological suppression".

Figures:
Figure 1: E and F should be side by side.
Legend Fig.1 (G,H) (Page 26; Lines 827-831): "The neurospheres were infected with lentivirus carrying AKT3.." carrying should be replaced by "expressing"... "infected with AKT3 mutation" should be replaced by 'infected with a lentivirus expressing the AKT3 mutant"

The Title "The size of neurospheres" is not clear.

Figure 2C: The limits of the "area of the cortex" are not clear to us (especially at E15.5). We think that the authors should compare the thickness of the cortex rather than the area.

Figure 5C. Title axis: "Time/h" should be changed (eg "Tc(hrs)").

Figure 5E: should be better explained in the legend. Explain what do the red arrows indicate?

Dear Reviewers,

We would like to sincerely thank you for your insightful comments and constructive suggestions, which have greatly enhanced the quality of our work. We have carefully addressed each of the points you raised, and our detailed point-by-point responses are provided below.

The manuscript has been thoroughly revised in accordance with your feedback, and all modifications are highlighted in blue for your convenience.

Thank you once again for your valuable time and guidance.

Sincerely,

Guiquan Chen
(on behalf of all authors)

Point-by-point Responses to Reviewers' comments

Reviewer 1's comments and our responses:

This study shows that AKT is essential for neural progenitor cell (NPC) proliferation and cortical development. Using human and mouse NPCs together with knockout models, the authors demonstrate that AKT loss prolongs G1, reduces NPC numbers, and causes microcephaly. Mechanistically, AKT phosphorylates DDB1 to inhibit CCND2 degradation by the CRL4AMBRA1 complex, thereby sustaining NPC proliferation. These findings identify an AKT-DDB1-CCND2 axis as a key regulator of cortical expansion and a potential mechanism underlying AKT-related brain malformations.
The manuscript is well written, easy to follow, and presents high-quality data in the first half, particularly in Figures 1-2 and 5. However, the mechanistic section is less convincing and leaves the paper feeling unfinished. Several major concerns remain:
1. The authors never address why cyclin D1 does not appear to fall under AKT regulation, unlike cyclins D2 and D3. This is crucial, since previous studies have shown that CRL4AMBRA1 regulates the half-life of all three D-type cyclins.

To answer this important question, we have conducted two sets of additional experiments. First, our new protein degradation work on CCND1 showed that AKT inhibition caused enhanced CCND1 degradation (Appendix Fig. S6F,G), and that AKT overexpression led to increased stability of CCND1 (Appendix Fig. S6J,K). Overall, results in Figs.6 & S6 indicate that AKT may regulate the post-translational expression of CCND1/2/3 in a similar way. Second, our new qRT-PCR analysis revealed significantly increased *Ccnd1* mRNA levels in the cortex of *Akt* cTKO mice

compared with control littermates (Appendix Fig. S6E), suggesting that AKT may negatively regulate the transcription of *Ccnd1*. We reason that enhanced transcription of *Ccnd1* may compensate the accelerated degradation of CCND1, leading to no significant decrease in CCND1 protein levels in *Akt* cTKO mice. The above new results have been incorporated into Paragraphs 1&2 on Page 9.

2. CCND3, despite being affected by AKT, is dropped from most downstream experiments without justification, and CCND1 is not addressed at all. This weakens the mechanistic narrative. The author should include CCND1 and CCND3 in the experiments shown in Fig. 6 and Fig. 7.

Thank the reviewer for this great question. We have conducted the following additional experiments for CCND3. Our new qRT-PCR results showed that *Ccnd3* mRNA levels were not significantly changed in *Akt* cTKO mice compared with age-matched control mice (Appendix Fig. S6D). Our new protein degradation data revealed that AKT inhibition caused enhanced CCND3 degradation (Appendix Fig. S6H,I), and that AKT overexpression resulted in increased stability of CCND3 (Appendix Fig. S6L,M). To examine whether reduced CCND3 contributes to the deficient NPC proliferation induced by AKT deletion, we performed rescue experiments by overexpressing CCND3 in neurospheres cultured from *Akt* cTKO mice. Western blot analysis confirmed a significant increase in CCND3 protein levels in neurospheres with lentivirus-mediated *Ccnd3* infection (Appendix Fig. S6P,Q). In contrast, we did not observe any difference in the average size of neurospheres between the CCND3-overexpressing group and the control group (Appendix Fig. S6R,S). Therefore, unlike CCND2, CCND3 does not rescue the NPC proliferation deficit caused by AKT loss. We have answered your question about CCND1 in our responses to your Question 1.

3. Ubiquitination assays (Fig. 6) were performed under non-denaturing conditions. The authors need to repeat these to ensure that they are not probing the ubiquitination of other cyclin interactors. Apart from MG132 and an AKT inhibitor as control they should also use AKT TKO controls.

According to your suggestions above, we conducted new experiments to examine ubiquitinated CDK4, since CDK4 is an interacting partner of CCNDs. 293T cells were transfected with plasmids expressing ubiquitin and CCND2, and were treated with MG132 for 4hrs.We performed immunoprecipitation for CCND2 and did detect CDK4 in the precipitate. While we detected CDK4 and ubiquitin in the precipitate of CCND2, we did not observe a high molecular-weight band corresponding to ubiquitinated CDK4 (Appendix Fig. S6T). These results indicate that ubquitinated CDK4 is not present in the precipitate of CCND2 under the current experimental conditions. Next, we performed immunoprecipitation for CCND2using neurospheres cultured from control and *Akt* cTKO mice. We found that ubiquitin levels were higher in the precipitate of CCND2 from *Akt* cTKO neurospheres than those from control

neurospheres (Appendix Fig. S6U), in line with the observations from 293T cells.

4. The TUNEL assay (Fig. 3) shows unusually high variability compared to otherwise consistent datasets and should be repeated.

To address this question, we performed additional TUNEL assays using new cohorts of mice aged at E13.5 and E15.5. The new data and the previous ones were pooled together, revealing no significant difference in the total number of TUNEL+ cells between control and *Akt* cTKO mice at either E13.5 or E15.5.

5. Comparisons of AKT binding to CRL4AMBRA1 complex components were performed on separate membranes, raising concerns about the robustness of these findings. Authors should load all the IP-s onto one membrane with comparable amounts of baits to assess the differential binding on these.

According to your suggestion above, we re-performed Co-IP experiments. We used the same membrane to examine the interactions between AKT and components of $CRL4^{AMBRA1}$ complex. Western blot showed that only CUL4A and DDB1 were detected in the precipitate for AKT, consistent with the results obtained using separate membranes.

6. While the authors demonstrate applicability of their findings in 293T cells, they never discuss how these results might extend to cancer biology, where the regulation of D-type cyclins is highly relevant.

Thank you very much for this suggestion. We have added the following paragraph in the Discussion section in the revised manuscript.

While the present study demonstrates that genetic or pharmacological inhibition of AKT suppresses NPC proliferation, recent evidence indicates that AKT inhibitors can synergize with temozolomide to inhibit glioblastoma growth (Cui *et al*, 2023). Glioblastoma is the most common primary brain tumor, and its malignancy is driven mainly by glioblastoma stem cells (GSCs) (Lathia *et al*, 2015). Notably, GSCs and NPCs share several fundamental cellular properties, including self-renewal capacity and the ability to differentiate into various lineages (Deng *et al*, 2025; Götz & Huttner, 2005; Zhu *et al*, 2014). Although AKT inactivation markedly reduces the NPC population, a substantial number of NPCs retain proliferative capacity both *in vivo* and *in vitro*, suggesting that additional signaling pathways contribute to the maintenance of the self-renewal in AKT-deficient NPCs. Identifying these compensatory pathways in future studies may uncover novel therapeutic targets relevant to both neurodevelopmental disorders and brain tumors.

7. The rescue experiments with MLN4924 are questionable. This inhibitor blocks all CRL mediated degradation in the cells which is toxic after an extended period of time

(24 hours). How were the author able to see rescue with something that is so broad in specificity? I'm surprised they even saw cell proliferation at all. Even if the MLN4924 was just used at 0.1 uM, treating the cells with it for days should cause apoptosis. More robust rescue experiments are needed to prove the authors mechanistic claims.

To answer your question above, we conducted additional TUNEL assays using cultured neurospheres treated with MLN4924 at different concentrations, including 0.1 uM, 1 uM, and 10 uM. Cells were treated with MLN4924 for 6 days and were collected for TUNEL assay. Our cell-counting results showed that the ratio of total TUNEL+ cells to total DAPI+ cells was not significantly different between neurospheres treated with mock- and 0.1 μM MLN4924, suggesting that 0.1 μM MLN4924 did not induce significant apoptosis (Appendix Fig. S7L,M). In contrast, significantly increased ratio of total TUNEL+ cells to total DAPI+ cells was detected for neurospheres treated with 1 μM or 10 μM MLN4924 compared with those treated with mock (Appendix Fig. S7L,M). Therefore, toxicity induced by MLN4924 is dosage-dependent in cultured neurospheres.

According to your suggestion, we conducted a new rescue experiment. Since our findings in this study indicate that AKT may regulate NPC proliferation via phosphorylation of DDB1, Lentivirus expressing phospho-mimetic mutant $DDB1^{T1125D}$ was used to transfect *Akt* cTKO neurospheres. Our new results showed that the expression of $DDB1^{T1125D}$ restored the neurosphere size in *Akt* cTKO cell cultures (Fig. 7M,N), providing strong evidence supporting the conclusion that AKT-mediated phosphorylation of DDB1 at T1125 is crucial for NPC proliferation.

In summary, while the descriptive and correlative data are strong, the mechanistic claims lack rigor and sufficient experimental support. Addressing the role of cyclin D1, including CCND1/3 in downstream analyses, providing more robust ubiquitination and rescue experiments, and discussing the broader implications in cancer would considerably strengthen the manuscript.

Thank the reviewer for these thoughtful concerns. We hope that the new lines of evidence we provided in the revised manuscript are sufficient to address your questions above.

Reference

Cui X, Zhao J, Li G, Yang C, Yang S, Zhan Q, Zhou J, Wang Y, Xiao M, Hong B *et al* (2023) Blockage of EGFR/AKT and mevalonate pathways synergize the antitumor effect of temozolomide by reprogramming energy metabolism in glioblastoma. *Cancer Communications* 43: 1326-1353

Deng Y, Yuan Z, Jin X, Wang Z, Gong R, Ren S, Park JB, Shi B, Yin J (2025) Synapsin III promotes neuronal-like transdifferentiation of glioblastoma stem cells by disrupting JAG1-Notch1 interaction. *Neuro-Oncology* 27: 1686-1701

Götz M, Huttner WB (2005) The cell biology of neurogenesis. *Nature Reviews Molecular Cell*

Biology 6: 777-788
Lathia JD, Mack SC, Mulkearns-Hubert EE, Valentim CL, Rich JN (2015) Cancer stem cells in glioblastoma. *Genes Dev* 29: 1203-1217
Zhu T-Z, Li X-M, Luo L-H, Song Z-Q, Gao X, Li Z-Q, Su J-Y, Liang G-B (2014) β-elemene inhibits stemness, promotes differentiation and impairs chemoresistance to temozolomide in glioblastoma stem-like cells. *Int J Oncol* 45: 699-709

Reviewer 2's comments and our responses:

This manuscript demonstrates a crucial role for AKT in regulating the G1/S transition in NPCs via a novel post-translational mechanism. The authors provide in vivo and in vitro evidence, moving logically from phenotypic characterization to mechanistic insight. The rescue experiments with both pharmacological inhibition and genetic overexpression of CCND2 are particularly compelling. The findings have important implications for understanding cortical development and the etiology of certain malformations of cortical development (MCDs). The major and minor points were listed as follows:

Major points：

1.Mechanistic specificity of DDB1 phosphorylation need to be further addressed. While the data strongly suggest that AKT-mediated phosphorylation of DDB1 at T1125 disrupts its interaction with CCND2, the exact mechanism remains somewhat speculative. Does this phosphorylation induce a conformational change in DDB1? Does it affect DDB1's interaction with other components of the CRL4 complex, like CUL4Aor AMBRA1?

We thank the reviewer for these insightful questions regarding the mechanistic specificity of DDB1 phosphorylation. To assess whether AKT-mediated phosphorylation of DDB1 at T1125 induces a conformational change, we performed structural modeling using SWISS-MODEL to compare wild-type DDB1 (T1125) with a phospho-mimetic mutant (D1125). Structural analysis revealed that residue T1125 in wild-type DDB1 forms ten hydrogen bonds with neighboring amino acids, whereas the D1125 mutation forms only two hydrogen bonds (Appendix Fig. S7C). As T1125 is located within the C-terminal α-helix of DDB1, this marked reduction in hydrogen bonding is predicted to destabilize the local structure, providing a plausible structural basis for altered protein-protein interactions.

To determine whether phosphorylation at T1125 affects the association of DDB1 with other components of the CRL4 complex, we conducted co-immunoprecipitation experiments using a non-phosphorylatable DDB1 mutant ($DDB1^{T1125A}$). Compared with wild-type DDB1, $DDB1^{T1125A}$ exhibited increased binding to both CUL4A and AMBRA1 (Appendix Fig. S7D-G). These findings indicate that phosphorylation of DDB1 at T1125 negatively regulates its interaction with core $CRL4^{AMBRA1}$ components.

Together, these new structural and biochemical data support a model in which AKT-mediated phosphorylation of DDB1 at T1125 induces conformational changes that weaken its association with the $CRL4^{AMBRA1}$ complex, thereby modulating CCND2 stability and NPC proliferation.

2.Functional rescue with phospho-mimetic DDB1 is essential but lacking. The study shows that the phospho-deficient mutant (T1125A) binds more CCND2. A critical functional test would be to see if a phospho-mimetic mutant (e.g., T1125D/E) can rescue the phenotypes in AKT-deficient NPCs.

To answer this question, we performed an additional rescue experiment using phospho-mimetic DDB1^{T1125D}. AKT-deficient neurospheres were transfected with a lentivirus expressing GFP or DDB1^{T1125D}. Our results showed that the average diameter of *Akt* cTKO neurospheres expressing DDB1^{T1125D} was significantly larger than that of those expressing GFP (Fig. 7M,N). Therefore, phospho-mimetic DDB1 restored the proliferation of AKT-deficient NPCs, providing strong evidence supporting the conclusion that the phosphorylation of DDB1 at T1125 is critical for AKT-dependent NPC proliferation.

3.The WB data (Fig. 6A, B, S6B, C) show that both CCND2 and CCND3 protein levels are reduced in Akt cTKO cortices. However, the authors focus exclusively on CCND2, justified by its established role in cortical development. The manuscript would be strengthened by addressing whether CCND3 is also a substrate of the same AKT-CRL4AMBRA1 axis.

Thank the reviewer for this great question. We conducted the following additional experiments to address whether CCND3 is also a key mediator for AKT-dependent NPC proliferation. First, our qRT-PCR analysis revealed no significant difference in *Ccnd3* mRNA levels between control and *Akt* cTKO mice (Appendix Fig. S6D). Therefore, AKT deficiency did not affect the transcription of *Ccnd3*. Second, we performed CCND3 protein degradation assays. Whereas AKT inhibition caused increased CCND3 degradation (Appendix Fig. S6H,I), AKT overexpression led to increased stability of CCND3 (Appendix Fig. S6L,M). These results suggest that AKT may regulate post-translational stability of CCND3. Third, we conducted a rescue experiment on CCND3 using *Akt* cTKO neurospheres. Western blot revealed increased levels of CCND3 in *Akt* cTKO neurospheres transfected with a lentivirus expressing CCND3 (Appendix Fig. S6P,Q). However, the average diameter of *Akt* cTKO neurospheres expressing CCND3 was not different from that of control *Akt* cTKO neurospheres (Appendix Fig. S6R,S). Therefore, CCND3 did not restore the proliferation ability in *Akt* cTKO neurospheres. These findings suggest that CCND3 is not a key mediator for AKT-dependent NPC proliferation.

4.Clarity on CRL4AMBRA1 complex specificity need to be addressed. The title and text refer to "CRL4AMBRA1" but the interaction data (Fig. 7A, S7A-C) show that AKT binds CUL4A and DDB1, but not CUL4B or AMBRA1. This is an important distinction, as it suggests the specific involvement of the CUL4A-containing complex.

Thank the reviewer for this excellent question. Recent evidence has shown that the E3 ligase CRL4^{AMBRA1} complex is a master regulator of CCND stability. Here we

identified that AKT may interact with CUL4A and DDB1, two core components of the CRL4^{AMBRA1} complex (Fig. 7A). We have shown that DDB1 is a direct substrate of AKT (Fig. 7B-D). Furthermore, our Co-IP data revealed that DDB1^{T1125A}, a non-phosphorylatable DDB1 mutant, exhibits increased binding to CUL4A and AMBRA1 (Appendix Fig. S7D-G), leading to enhanced recognition of CCND2 (Fig. 7E,F). For clarification purpose, we propose the following working model, in which AKT phosphorylates DDB1 to inhibit the activity of the CRL4^{AMBRA1} complex, increasing the stability of CCND proteins.

Minor points:

1.Some co-IP results (e.g., Fig. 7A, E, P, Q) are presented as representative blots without quantification. Including quantitative data from multiple replicates (as was done in Fig. 7F) for these key experiments would bolster the statistical rigor.

For Fig.7A, the reason why no quantification results were presented is that, these Co-IP experiments were used for qualitative purpose. This finding indicates that AKT may interact with DDB1 and CUL4A. These experiments were repeated three times. For experiments in Fig.7E, the quantification results were indeed presented in Fig.7F. Since there were no panels P and Q in Fig. 7, we have corrected the figure labeling in the revised manuscript.

2.Inhibitor specificity issue: while MK2206 and Capivasertib are well-established AKT inhibitors, a brief mention in the Methods or Results about their specific targets (allosteric vs. ATP-competitive) and the justification for using both would be helpful for readers.

Thanks very much for this great suggestion. We have updated information for MK2206 and Capivasertib in the revised manuscript.

3.In Fig. 1K, L: the description of the CFSE assay is slightly confusing. It states that Akt cTKO has a "greater proportion of high CFSE-intensity cells," which is correct (less dilution due to less proliferation). However, the graph in L is gated on cells with low CFSE intensity. The legend should be clarified to avoid misinterpretation.

Thanks very much for this great suggestion. We have revised the text to "a lower proportion of low CFSE-intensity cells."

Reviewer 3's comments and our responses:

The manuscript by Wang et al provides new insights into a long-standing question on the molecular mechanisms controlling G1 phase progression in cortical development, by addressing the role of AKT in the regulation of mouse neural progenitor cell (NPCs) proliferation.
The authors performed a series of experiments on the developing mouse cortex, and used in vitro models (HEK293T cells, mouse neurospheres and human NPC), to dissect the molecular mechanisms involved in this regulation.
They first show that AKT is highly expressed in mouse NPCs, both in vivo and in vitro, and that gain-of-function mutations of AKT increase neurosphere size.
The authors then examine the effects of AKT loss in NPCs, demonstrating that mice with triple knockout (cTKO) of Akt1/2/3 in NPCs exhibit a thinner cortex, reduced cortical progenitor proliferation, and altered G1 phase progression. Similarly, pharmacological inhibition of AKT decreases neurosphere size; neurospheres isolated from cTKO mice are also smaller than controls, and display reduced proliferation.
These findings are complemented by RNAseq analysis of the cTKO cortices, which provides supportive evidence for deregulated cell-cycle progression, including G1 phase.
As Akt cTKO NPCs exhibit reduced levels of the G1-phase regulator Cyclin D2 (CCND2), the authors further dissect the link between AKT and CCND2 in vitro in HEK296T cells. They show that AKT activity stabilizes CCND2, via inhibition of the ubiquitine-proteasome pathway.
Finally, they return to mouse NPCs and perform rescue experiments in cTKO neurospheres that indicate that altered proliferation of cTKO NPCs is due, at least in part, to reduced levels of CCND2.

General comments:
Overall, the data are clearly presented and the work provides a mechanistic link between AKT signaling and CCND2 stability, to control the cell-cycle in NPCs.
However, the general conclusion heavily relies on the in vivo data and the analysis of cTKO mice. The methods implemented for the in vivo quantifications must thus be clarified/corrected. Specifically, quantifications of PAX6+, TBR2+, Sox2+, BrdU+, pH3+, and Tunnel+ cells need to be improved (detailed below).
The choice of HEK293T cells for mechanistic dissection, although experimentally convenient, raises concerns about physiological relevance and it would be interesting to show that the same regulatory mechanism (at least some aspects) is conserved in NPCs themselves.
The authors omitted to quote relevant work related to the role of G1 phase and cyclins in cell-cycle regulation of cortical progenitors (eg Pilaz et al. PNAS, 2009 and other studies). This should be corrected.

Thank you very much for these excellent comments and suggestions. While we have addressed your specific points in the following sections, we have cited additional

references about the role of G1 phase and cyclins in cell-cycle regulation of cortical progenitors.

Specific points:

1. Consequences of AKT GOF in NPCs

The authors show that expression of the "p.T288I mutant significantly increased neurosphere size compared to WT AKT3 controls (Fig. 1, G and H, and fig. S1D), demonstrating enhanced NPC proliferation" (Lines 105-106).

-They should show the level of AKT3 in control neurospheres (neurospheres non transfected, and neurospheres transfected with AKT3) and in neurospheres expressing the mutant T288I.

-They should analyse the proliferation rate of NPCs in T288I mutant neurospheres vs AKT3 controls (eg, %Ki67+ cells).

Thanks very much for these insightful comments. First, Western blot for AKT3 was presented in Appendix Fig. S1I, J. Cultured neurospheres were transfected with lentivirus expressing GFP, AKT3, or $AKT3^{T288I}$. We observed significantly increased levels of AKT3 in neurospheres expressing wild-type AKT3 or $AKT3^{T288I}$ compared to those expressing GFP. Second, we performed fluorescence immunostaining for SOX2 and KI67. Quantification results showed that the proportion of KI67+/SOX2+ cells to total SOX2+ cells was significantly higher in neurospheres expressing $AKT3^{T288I}$ than those expressing wild-type AKT3 (Appendix Fig. S1K,L). Therefore, AKT gain-of-function mutation caused enhanced NPC proliferation.

2.Pharmalogical AKT inhibition

Page 4 line 99: Treatment of mouse neurospheres with two selective AKT inhibitors "caused a marked reduction in neurosphere size compared to mock-treated controls". The authors conclude that it indicates "suppressed NPC proliferation upon AKT inhibition".

- The authors need to analyse cell proliferation in neurospheres treated with the inhibitors vs controls (eg FACS analysis).

- Decreased neurosphere size could be also be due to cell death. Therefore, authors should also analyse cell death.

Thank the reviewer for these insightful comments. First, we performed co-staining for KI67/SOX2 using neurospheres treated with AKT inhibitors or mock control. We did observe a significant decrease in the ratio of KI67+/SOX2+ cells to total SOX2+ cells in neurospheres treated with AKT inhibitors compared to those treated with mock (Appendix Fig. S1D,E), suggesting that AKT inhibition impairs NPC proliferation. Second, TUNEL assay showed that the proportion of TUNEL+/DAPI+ cells to total DAPI+ cells was comparable between mock- and inhibitors-treated neurospheres (Appendix Fig. S1F,G). Overall, these findings suggest that the decreased size of inhibitors-treated neurospheres is due to a proliferation defect but not apoptosis.

3.Consequences of AKT inhibition using the triple knock-out mice:

-The authors quantified multiple parameters (Figs 2 and 3) (including number of PAX6+, TBR2+ per section; BrdU+ cells following a BrdU pulse; pH3+ cells, and apoptotic cells by TUNNEL staining...). They conclude that AKT loss affects NPC proliferation rather than survival.
This conclusion depends critically on the method used for the quantifications.
It is not clear how the authors determined the size of the sections (the squares indicated by dotted lines?).
Reporting absolute cell counts per section is not rigorous nor accurate, because counts vary with local cell density. The authors should report the proportion of positive cells among the total (DAPI+) cells.
In Figure S3, they quantified the "area of TUJ1+ or DCX+ cells/103 µm2". What does "area of TUJ1+"? mean?
All the above mentionned quantifications need to be reanalyzed using appropriate methods, as the validity of the in vivo results- and consequently a substantial part of the conclusions-fully depends on this.

To clarify the quantification methods, we chose the dorsal cortex, indicated by the dotted lines in the figures, to count different types of cells. While BrdU+ and PH3+ NPCs are distributed throughout the brain, PAX6 and TBR2 are specifically expressed in NPCs in the dorsal cortex. These characteristics have helped us delineate the boundary of the dorsal cortex in brain sections for IHC experiments. In this study, we consistently selected the ventricular zone (VZ) in the dorsal cortex and the corresponding cortical area, outlined by white dotted lines, for quantitative analysis.

We agree that the cell density may vary across brain sections. To overcome this problem, we consistently focused on the dorsal cortex to count NPCs and DAPI+ cells. The latter are composed of NPCs and immature neurons in the dorsal cortex at E13.5 and E15.5. According to your suggestions, we conducted BrdU pulse-labeling experiments to examine NPC proliferation. After we performed co-immunostaining for PAX6/BrdU and TBR2/BrdU (Fig. 3E,G), we quantified the ratio of PAX6+/BrdU+ cells to total PAX6+ cells and that of TBR2+/BrdU+ cells to total TBR2+ cells. We found that these ratios were significantly reduced in *Akt* cTKO cortices compared to controls (Fig. 3F,H). These results suggest that AKT deficiency impairs the NPC proliferation.

We agree that decreased areas for TUJ1+ cells and DCX+ cells do not represent decreased population size for immature neurons. According to your comments, we have removed data for TUJ1+ cells and DCX+ cells from the revised manuscript.

4.Cell cycle and INM in cTKO
"At 12 hours post-injection, significantly fewer BrdU+ cells were detected in the basal VZ (R2) of Akt cTKO cortices, suggesting delayed cell cycle progression (Fig. 5, E to

G, and fig. S5, C and D)." (Page 7. Lines 183-185).
The authors should explain this result more clearly. The data suggests altered, rather than delayed, cell cycle progression.

These are great suggestions. Accordingly, we have changed "delayed cell cycle progression" to "altered cell cycle progression" in the revised manuscript.

To precisely define the R1 and R2 regions, the authors need to provide PAX6/TBR2/BrdU co-stainings images for both Control and cTKO.

According to your suggestion, we conducted additional BrdU pulse-labeling experiments, in which BrdU were injected into pregnant mice at E15.5 and brain tissues were collected 12 or 24 hours later. We then performed co-immunostaining for PAX6/BrdU or TBR2/BrdU using brain sections from control and *Akt* cTKO mice. For quantification purposes, the VZ was subdivided into the apical VZ (Region 1, R1), defined as the PAX6+/TBR2- domain, and the basal VZ (Region 2, R2). At the 12-hour post-BrdU injection, there was significantly increased number of BrdU+ cells in the R1 of *Akt* cTKO cortices compared to controls (Fig. 5F G, Appendix Fig. S5D). At the 24-hour post-BrdU injection, fewer BrdU+ cells in the R1were observed in *Akt* cTKO mice than in controls (Fig. 5H,I, Appendix Fig. S5E). These results indicate altered kinetics of INM and cell cycle progression upon AKT deletion.

"A 24-hour BrdU labeling experiment further revealed that fewer Akt cTKO NPCs completed second mitotic cycle compared to controls (Fig. 5, H and I, and fig. S5E)". (Page 7. Lines 185-187).
It is not clear why they deduce that "NPCs completed second mitotic cycle compared to controls". The authors need to clarify this point.

At E14.5 in mice, the total duration of cell cycle is approximately 14 hours, with S phase and G2/M phase being ~5 hours, ~2 hours, respectively (Martynoga *et al*, 2005). While BrdU labels NPCs at S phase upon injection at E14.5, these labeled NPCs will enter the next S phase about 14 hours later. It takes approximately 7 hours for these NPCs to complete S, G2, and M phases. Consequently, these NPCs complete the second mitotic cell cycle about 24 hours after the BrdU injection at E14.5.

Investigating the impact of AKT KO on INM requires a more detailed analysis (ie, the position of G1, S, G2 and M cells within the thickness of the VZ).

Thank the reviewer for this insightful comment. Although our BrdU pulse-labeling analysis helped us reveal altered INM and cell cycle progression in *Akt* cTKO cortices (Fig. 5 F-I, Appendix Fig. S5D,E), this methodology does not allow us to precisely count NPCs at each specific cell cycle phase (G1, S, G2, M). We acknowledge that the BrdU pulse-labeling approach used in this study provides an indirect measurement of INM kinetics. We currently do not have an alternative method for direct, real-time

INM analysis.

Minor points:

-The description of the mutant mice (lines 124-130): should be moved upper (mixed with lines 108-111).

According to your suggestion above, we have made these changes in the revised manuscript.

line 86-87: The title is not clear. "AKT promotes NPC proliferation: enhanced by gain-of-function mutations and inhibited by pharmacological suppression".

We changed it to “AKT is essential for NPC proliferation *in vitro*”.

Figures:
Figure 1: E and F should be side by side.
Legend Fig.1 (G,H) (Page 26; Lines 827-831): "The neurospheres were infected with lentivirus carrying AKT3.." carrying should be replaced by "expressing"... "infected with AKT3 mutation" should be replaced by 'infected with a lentivirus expressing the AKT3 mutant"
The Title "The size of neurospheres" is not clear.

Thanks very much for these great suggestions. The following changes were made in the revised manuscript. Figs. 1E and 1F are now presented side-by-side for direct comparison. The Figure 1 legends have been revised according to your suggestions. The title for this panel has been changed to "Representative images and the average diameter for cultured neurospheres expressing WT and mutant AKT3".

Figure 2C: The limits of the "area of the cortex" are not clear to us (especially at E15.5). We think that the authors should compare the thickness of the cortex rather than the area.

Thank you for this helpful suggestion. We measured cortical thickness for control and *Akt* cTKO mice at E13.5 and E15.5. The results were shown in Fig.2C.

Figure 5C. Title axis: "Time/h" should be changed (eg "Tc(hrs)").

According to your suggestion, we have changed "Time/h" to "Tc (hrs)".

Figure 5E: should be better explained in the legend. Explain what do the red arrows indicate?

According to your suggestion, we have added the indication for the red arrows in the

revised manuscript.

Reference

Martynoga B, Morrison H, Price DJ, Mason JO (2005) Foxg1 is required for specification of ventral telencephalon and region-specific regulation of dorsal telencephalic precursor proliferation and apoptosis. *Developmental Biology* 283: 113-127

Dear Dr. Chen,

Thank you for the submission of your revised manuscript. We have now received the enclosed reports from the referees and I am happy to say that all support its publication now. Referee 1 still has one more suggestion that I would like you to address and incorporate before we can proceed with the official acceptance of your manuscript.

A few editorial requests will also need to be addressed:

- Please remove the authors credits from the ms file. All credits need to be entered during online ms submission.

- Please answer all questions on statistics in the author checklist and send us a new, completed list.

- A FIGURE CALLOUT is missing for Fig. 7C, please add.

- The Materials and Methods section should include a separate Reagents and Tools Table file (listing key reagents, experimental models, software and relevant equipment and including their sources and relevant identifiers) and a Methods and Protocols section in which methods should be described using a step-by-step protocol format with bullet points. More information and a table template is available in our guide to authors online: https://link.springer.com/journal/44319/submission-guidelines

- The Source Data for the Appendix figures can be combined into one folder.

- The manuscript sections should be in the following order: Title page - Abstract & Keywords - Introduction - Results - Discussion - Methods - Data Availability - Acknowledgments - Disclosure Statement & Competing Interests - References - Figure Legends - (Main Tables with legends if applicable) - Expanded View Figure Legends.

- The resolution of Figure 1A, Figure 5A, and the Appendix file figures is too low for blot and microscopy images. This reduction in resolution is commonly caused by converting original 16-bit TIFF files to RGB format for publication. While this is not inherently problematic, it can raise concerns about image integrity for critical readers. To avoid any misunderstanding and to meet EMBO Press standards, we kindly ask that you:
* Resubmit these figures at the captured original data resolution.
* Apply the same resolution standards to the blot source data files, which are also currently below the required quality threshold. Please upload the blot source data files as high-resolution .tiff

* Figure Legends - Comments *

- Please note that the exact p values are not provided in the legends of figures 1F, J; 2C, G; 3H, 4F-H; 5I, 6E, G, S; 7H. Please provide exact values as reasonable.
- Please indicate the statistical test used for data analysis in the legends of figures 4F-H
- Please note that the dashed borders are not defined in the legend of figure 2D, E; 3A, C. This needs to be rectified.

I would like to suggest some minor changes to the abstract. Please let me know whether you agree with the following:

Neural progenitor cell (NPC) proliferation is fundamental for population expansion and brain development. G1 phase control determines the cell cycle duration of NPCs and thereby affects their proliferation efficiency. However, the molecular mechanisms governing G1 phase progression in NPCs remain unclear. Here we show that AKT gain-of-function mutations and pharmacological inhibition exert opposing effects on NPC proliferation. Consistently, Emx1-Cre-mediated deletion of Akt1/2/3 in mice impairs NPC proliferation and disrupts cortical development. We find that AKT deficiency induces G1 phase arrest and prolongs the cell cycle of NPCs. Mechanistically, we demonstrate that AKT-mediated phosphorylation inhibits the activity of the CRL4AMBRA1 E3 ubiquitin ligase to safeguard cyclin D2 (CCND2) stability. Specifically, AKT phosphorylates DDB1, the adaptor of CRL4AMBRA1, which disrupts its interaction with CCND2 and reduces its degradation. These findings reveal a post-translational mechanism impacting NPC cell cycle and cortical morphogenesis, providing insight into the etiology of malformations of cortical development.

EMBO press papers are accompanied online by A) a short (1-2 sentences) summary of the findings and their significance, B) 2-3 bullet points highlighting key results and C) a synopsis image that is exactly 550 pixels wide and 200-600 pixels high (the height is variable). The synopsis image should provide a sketch of the major findings, like a graphical abstract. Please note that text needs to be readable at the final size. Please send us this information along with the final manuscript.

I look forward to seeing a final version of your manuscript as soon as possible.

Best regards,
Esther

Esther Schnapp, PhD
Senior Editor
EMBO reports

Referee #1:

Most of my original concerns have now been addressed. In particular, the new phospho-mimetic DDB1(T1125D) rescue experiment provides a more convincing mechanistic support for the proposed AKT-DDB1-CCND2 axis and strengthens the paper.

However, I remain concerned about the ubiquitination assays. The authors still rely on non-denaturing immunoprecipitation conditions. Given that CCND2 robustly co-immunoprecipitates with CDK4, the presence of ubiquitin signal in the CCND2 IP cannot be unambiguously attributed to CCND2 itself. Any ubiquitinated CCND2-interacting protein would be carried through the IP, and this possibility is not experimentally excluded. The additional CDK4 control does not resolve this issue, as it does not address ubiquitinated species other than CDK4. A denaturing ubiquitination assay would be required to definitively demonstrate CCND2 ubiquitination.

Aside from this unresolved technical point, I find the revised manuscript substantially improved

Referee #2:

Authors have adequately provided reasonable data and arguments to clarify previous concerns. A very nice study

Referee #3:

In our opinion, the authors have adequately revised the ms to answer Reviewer's queries.

Point-by-point Responses to Editorial Requests and Referee 1' Comments

A few editorial requests will also need to be addressed:

- Please remove the authors credits from the ms file. All credits need to be entered during online ms submission.

We have removed the authors credits in the revised manuscript file.

- Please answer all questions on statistics in the author checklist and send us a new, completed list.

We answered all questions on statistics, and uploaded a new checklist.

- A FIGURE CALLOUT is missing for Fig. 7C, please add.

We have added the figure callout for Fig.7C in the revised manuscript file.

- The Materials and Methods section should include a separate Reagents and Tools Table file (listing key reagents, experimental models, software and relevant equipment and including their sources and relevant identifiers) and a Methods and Protocols section in which methods should be described using a step-by-step protocol format with bullet points. More information and a table template is available in our guide to authors online: https://link.springer.com/journal/44319/submission-guidelines

We have prepared a separate Reagents and Tools Table file, and have described methods for Neurosphere culture and Cell cycle analysis by flow cytometry using a step-by-step protocol format.

- The Source Data for the Appendix figures can be combined into one folder.

As you suggested, we have combined the Appendix figures into one folder.

- The manuscript sections should be in the following order: Title page - Abstract & Keywords - Introduction - Results - Discussion - Methods - Data Availability - Acknowledgments - Disclosure Statement & Competing Interests - References - Figure Legends - (Main Tables with legends if applicable) - Expanded View Figure Legends.

We have updated the manuscript sections following your suggestions. Our appendix figures and figure legends are provided in the appendix file. The section for Expanded View Figure Legends is not included in the main manuscript file.

- The resolution of Figure 1A, Figure 5A, and the Appendix file figures is too low for blot and microscopy images. This reduction in resolution is commonly caused by converting original 16-bit TIFF files to RGB format for publication. While this is not inherently problematic, it can raise concerns about image integrity for critical readers. To avoid any misunderstanding and to meet EMBO Press standards, we kindly ask that you:

* Resubmit these figures at the captured original data resolution.

Thank you for your feedback and for helping us ensure compliance with EMBO Press standards. In response to your concerns regarding image resolution: Figures 1A and 5A have been replaced with high-resolution versions that reflect the original

captured data. Regarding the Appendix file, the lower resolution of the figures was due to compression during the initial Word-to-PDF conversion. We have now reconverted the file using high-quality export settings to preserve image integrity.

All figures are now provided at the appropriate resolution to avoid any misunderstanding and to meet the journal's requirements.

* Apply the same resolution standards to the blot source data files, which are also currently below the required quality threshold. Please upload the blot source data files as high-resolution .tiff

The blot source data files have been updated and are now provided as high-resolution tiff files.

* Figure Legends - Comments *

- Please note that the exact p values are not provided in the legends of figures 1F, J; 2C, G; 3H, 4F-H; 5I, 6E, G, S; 7H. Please provide exact values as reasonable.
- Please indicate the statistical test used for data analysis in the legends of figures 4F-H

Thank you for your detailed review. We have revised the figure legends to include exact p-values as requested. For most figures, the exact p-values are now provided directly in the figures or their legends. Regarding Figure 4F, which presents a GSEA result, the nominal p-value was reported by the software as "0." As this represents a p-value below the resolution limit of the permutation test (specifically, less than 1/1000 = 0.001), we have indicated this as $p < 0.001$ in the figure legend, a standard practice for reporting such results (Phipson & Smyth, 2010). Additionally, the specific statistical tests used for the data analysis have now been included in the legends for Figures 4F-H.

- Please note that the dashed borders are not defined in the legend of figure 2D, E; 3A, C. This needs to be rectified.

We have added a description of the dashed borders in the figure legends.

I would like to suggest some minor changes to the abstract. Please let me know whether you agree with the following:

Neural progenitor cell (NPC) proliferation is fundamental for population expansion and brain development. G1 phase control determines the cell cycle duration of NPCs and thereby affects their proliferation efficiency. However, the molecular mechanisms governing G1 phase progression in NPCs remain unclear. Here we show that AKT gain-of-function mutations and pharmacological inhibition exert opposing effects on NPC proliferation. Consistently, Emx1-Cre-mediated deletion of Akt1/2/3 in mice impairs NPC proliferation and disrupts cortical development. We find that AKT deficiency induces G1 phase arrest and prolongs the cell cycle of NPCs. Mechanistically, we demonstrate that AKT-mediated phosphorylation inhibits the

activity of the CRL4AMBRA1 E3 ubiquitin ligase to safeguard cyclin D2 (CCND2) stability. Specifically, AKT phosphorylates DDB1, the adaptor of CRL4AMBRA1, which disrupts its interaction with CCND2 and reduces its degradation. These findings reveal a post-translational mechanism impacting NPC cell cycle and cortical morphogenesis, providing insight into the etiology of malformations of cortical development.

We agree with these minor changes and have implemented them in the manuscript. Thank you for the helpful suggestions.

EMBO press papers are accompanied online by A) a short (1-2 sentences) summary of the findings and their significance, B) 2-3 bullet points highlighting key results and C) a synopsis image that is exactly 550 pixels wide and 200-600 pixels high (the height is variable). The synopsis image should provide a sketch of the major findings, like a graphical abstract. Please note that text needs to be readable at the final size. Please send us this information along with the final manuscript.

We have prepared these materials and submitted them along with the final manuscript.

Referee #1:

Most of my original concerns have now been addressed. In particular, the new phospho-mimetic DDB1(T1125D) rescue experiment provides a more convincing mechanistic support for the proposed AKT-DDB1-CCND2 axis and strengthens the paper.

However, I remain concerned about the ubiquitination assays. The authors still rely on non-denaturing immunoprecipitation conditions. Given that CCND2 robustly co-immunoprecipitates with CDK4, the presence of ubiquitin signal in the CCND2 IP cannot be unambiguously attributed to CCND2 itself. Any ubiquitinated CCND2-interacting protein would be carried through the IP, and this possibility is not experimentally excluded. The additional CDK4 control does not resolve this issue, as it does not address ubiquitinated species other than CDK4. A denaturing ubiquitination assay would be required to definitively demonstrate CCND2 ubiquitination.

In response to the reviewer's concern regarding the interpretation of ubiquitination signals from non-denaturing immunoprecipitation (IP) conditions, specifically the possibility that ubiquitin signals detected in CCND2 IPs could arise from co-precipitated CCND2-interacting proteins such as CDK4, we conducted a ubiquitination assay under stringent denaturing conditions. To achieve this, cell lysates were prepared in buffer containing 2% SDS and boiled at 95 °C for 5 minutes to disrupt protein-protein interactions. The SDS concentration was then diluted to 0.2% with lysis buffer prior to immunoprecipitation, enabling specific isolation of CCND2 under denaturing conditions. Immunoprecipitates were subsequently analyzed by

Western blot. As shown in Appendix Fig. S6T, HA-tagged ubiquitin was detected in the CCND2 IP, and the signal was markedly enhanced upon MG132 treatment. These results provide direct evidence that CCND2 is ubiquitinated and effectively exclude the possibility that the observed signal derives from associated proteins.

Aside from this unresolved technical point, I find the revised manuscript substantially improved

Thank you for the positive evaluation. We are glad the revisions have strengthened the manuscript.

Reference

Phipson B, Smyth GK (2010) Permutation P-values Should Never Be Zero: Calculating Exact P-values When Permutations Are Randomly Drawn. *Statistical Applications in Genetics and Molecular Biology* 9

Guiquan Chen
Nanjing University
12 Xuefu Road
Nanjing, Jiangsu 210061
China

Dear Dr. Chen,

I am very pleased to accept your manuscript for publication in the next available issue of EMBO reports. Thank you for your contribution to our journal.

Your manuscript will be processed for publication by EMBO Press. It will be copy edited and you will receive page proofs prior to publication. Please note that you will be contacted by Springer Nature Author Services to complete licensing and payment information.

You may qualify for financial assistance for your publication charges - either via a Springer Nature fully open access agreement or an EMBO initiative. Check your eligibility: https://link.springer.com/journal/44319/how-to-publish-with-us

Should you be planning a Press Release on your article, please get in contact with embo_production@springernature.com as early as possible in order to coordinate publication and release dates.

If you have any questions, please do not hesitate to contact the Editorial Office. Thank you for your contribution to EMBO Reports.

Yours sincerely,

Esther Schnapp, PhD
Senior Editor
EMBO reports

>>> Please note that it is EMBO Reports policy for the transcript of the editorial process (containing referee reports and your response letter) to be published as an online supplement to each paper. If you do NOT want this, you will need to inform the Editorial Office via email immediately. More information is available here: https://link.springer.com/partners/embo-press/editorial-policies#Peer%20review

EMBO Press Author Checklist

Corresponding Author Name: He Wang, Guiquan Chen, Jian-Jun Yang	
Journal Submitted to: Embo Reports	
Manuscript Number: EMBOR-2025-62661	

USEFUL LINKS FOR COMPLETING THIS FORM
The EMBO Journal - Author Guidelines
EMBO Reports - Author Guidelines
Molecular Systems Biology - Author Guidelines
EMBO Molecular Medicine - Author Guidelines

Reporting Checklist for Life Science Articles (updated January
This checklist is adapted from Materials Design Analysis Reporting (MDAR) Checklist for Authors. MDAR establishes a minimum set of requirements in transparent reporting in the life sciences (see Statement of Task: 10.31222/osf.io/9sm4x). Please follow the journal's guidelines in preparing your
Please note that a copy of this checklist will be published alongside your article.

Abridged guidelines for figures
1. Data
The data shown in figures should satisfy the following conditions:
- the data were obtained and processed according to the field's best practice and are presented to reflect the results of the experiments in an accurate and unbiased manner.
- ideally, figure panels should include only measurements that are directly comparable to each other and obtained with the same assay.
- plots include clearly labeled error bars for independent experiments and sample sizes. Unless justified, error bars should not be shown for technical
- if n<5, the individual data points from each experiment should be plotted. Any statistical test employed should be justified.
- Source Data should be included to report the data underlying figures according to the guidelines set out in the authorship guidelines on Data

2. Captions
Each figure caption should contain the following information, for each panel where they are relevant:
- a specification of the experimental system investigated (eg cell line, species name).
- the assay(s) and method(s) used to carry out the reported observations and measurements.
- an explicit mention of the biological and chemical entity(ies) that are being measured.
- an explicit mention of the biological and chemical entity(ies) that are altered/varied/perturbed in a controlled manner.
- the exact sample size (n) for each experimental group/condition, given as a number, not a range;
- a description of the sample collection allowing the reader to understand whether the samples represent technical or biological replicates (including how many animals, litters, cultures, etc.).
- a statement of how many times the experiment shown was independently replicated in the laboratory.
- definitions of statistical methods and measures:
  - common tests, such as t-test (please specify whether paired vs. unpaired), simple χ2 tests, Wilcoxon and Mann-Whitney tests, can be unambiguously identified by name only, but more complex techniques should be described in the methods section;
  - are tests one-sided or two-sided?
  - are there adjustments for multiple comparisons?
  - exact statistical test results, e.g., P values = x but not P values < x;
  - definition of 'center values' as median or average;
  - definition of error bars as s.d. or s.e.m.

Please complete ALL of the questions below.
Select "Not Applicable" only when the requested information is not relevant for your study.

Materials

Newly Created Materials	Information included in the manuscript?	In which section is the information available? (Reagents and Tools Table, Materials and Methods, Figures, Data Availability Section)
New materials and reagents need to be available; do any restrictions apply?	Not Applicable	

Antibodies	Information included in the manuscript?	In which section is the information available? (Reagents and Tools Table, Materials and Methods, Figures, Data Availability Section)
For **antibodies** provide the following information:<br>- Commercial antibodies: RRID (if possible) or supplier name, catalogue number and or/clone number<br>- Non-commercial: RRID or citation	Yes	Reagents and Tools Table

DNA and RNA sequences	Information included in the manuscript?	In which section is the information available? (Reagents and Tools Table, Materials and Methods, Figures, Data Availability Section)
**Short novel DNA or RNA including primers, probes**: provide the sequences.	Yes	Reagents and Tools Table

Cell materials	Information included in the manuscript?	In which section is the information available? (Reagents and Tools Table, Materials and Methods, Figures, Data Availability Section)
**Cell lines:** Provide species information, strain. Provide accession number in repository **OR** supplier name, catalog number, clone number, and/**OR** RRID.	Yes	Reagents and Tools Table
**Primary cultures:** Provide species, strain, sex of origin, genetic modification status.	Yes	Methods
Report if the cell lines were recently **authenticated** (e.g., by STR profiling) and tested for mycoplasma contamination.	Not Applicable	

Experimental animals	Information included in the manuscript?	In which section is the information available? (Reagents and Tools Table, Materials and Methods, Figures, Data Availability Section)
**Laboratory animals or Model organisms:** Provide species, strain, sex, age, genetic modification status. Provide accession number in repository **OR** supplier name, catalog number, clone number, **OR** RRID.	Yes	Regents and Tool Table, Methods
**Animal observed in or captured from the field:** Provide species, sex, and age where possible.	Not Applicable	
Please detail **housing and husbandry conditions**.	Yes	Methods

Plants and microbes	Information included in the manuscript?	In which section is the information available? (Reagents and Tools Table, Materials and Methods, Figures, Data Availability Section)
**Plants:** provide species and strain, ecotype and cultivar where relevant, unique accession number if available, and source (including location for collected wild specimens).	Not Applicable	
**Microbes:** provide species and strain, unique accession number if available, and source.	Not Applicable	

Human research participants	Information included in the manuscript?	In which section is the information available? (Reagents and Tools Table, Materials and Methods, Figures, Data Availability Section)
If collected and within the bounds of privacy constraints report on age, sex and gender or ethnicity for all study participants.	Not Applicable	

Core facilities	Information included in the manuscript?	In which section is the information available? (Reagents and Tools Table, Materials and Methods, Figures, Data Availability Section)
If your work benefited from core facilities, was their service mentioned in the acknowledgments section?	Not Applicable	

Design

Study protocol	Information included in the manuscript?	In which section is the information available? (Reagents and Tools Table, Materials and Methods, Figures, Data Availability Section)
If study protocol has been **pre-registered, provide DOI in the manuscript**. For clinical trials, provide the trial registration number **OR** cite DOI.	Not Applicable	
Report the **clinical trial registration number** (at ClinicalTrials.gov or equivalent), where applicable.	Not Applicable	

Laboratory protocol	Information included in the manuscript?	In which section is the information available? (Reagents and Tools Table, Materials and Methods, Figures, Data Availability Section)
Provide DOI OR other citation details if **external detailed step-by-step protocols** are available.	Not Applicable	

Experimental study design and statistics	Information included in the manuscript?	In which section is the information available? (Reagents and Tools Table, Materials and Methods, Figures, Data Availability Section)
Include a statement about **sample size** estimate even if no statistical methods were used.	Yes	Methods
Were any steps taken to minimize the effects of subjective bias when allocating animals/samples to treatment (e.g. **randomization procedure**)? If yes, have they been described?	Yes	Methods
Include a statement about **blinding** even if no blinding was done.	Yes	Methods
Describe **inclusion/exclusion criteria** if samples or animals were excluded from the analysis. Were the criteria pre-established?<br><br>If sample or data points were omitted from analysis, report if this was due to attrition or intentional exclusion and provide justification.	Yes	Methods
For every figure, are **statistical tests** justified as appropriate? Do the data meet the assumptions of the tests (e.g., normal distribution)? Describe any methods used to assess it. Is there an estimate of variation within each group of data? Is the variance similar between the groups that are being statistically compared?	Yes	Methods, Figures

Sample definition and in-laboratory replication	Information included in the manuscript?	In which section is the information available? (Reagents and Tools Table, Materials and Methods, Figures, Data Availability Section)
In the figure legends: state number of times the experiment was **replicated** in laboratory.	Yes	Figures
In the figure legends: define whether data describe **technical or biological replicates**.	Yes	Figures

Ethics

Ethics	Information included in the manuscript?	In which section is the information available? (Reagents and Tools Table, Materials and Methods, Figures, Data Availability Section)
Studies involving **human participants**: State details of **authority granting ethics approval** (IRB or equivalent committee(s), provide reference number for approval.	Not Applicable	
Studies involving **human participants**: Include a statement confirming that **informed consent** was obtained from all subjects and that the experiments conformed to the principles set out in the WMA Declaration of Helsinki and the Department of Health and Human Services Belmont Report.	Not Applicable	
Studies involving **human participants:** For publication of **patient photos**, include a statement confirming that consent to publish was obtained.	Not Applicable	
Studies involving experimental **animals**: State details of **authority granting ethics approval** (IRB or equivalent committee(s), provide reference number for approval. Include a statement of compliance with ethical regulations.	Yes	Methods
Studies involving **specimen and field samples:** State if relevant **permits** obtained, provide details of authority approving study; if none were required, explain why.	Not Applicable	

Dual Use Research of Concern (DURC)	Information included in the manuscript?	In which section is the information available? (Reagents and Tools Table, Materials and Methods, Figures, Data Availability Section)
Could your study fall under dual use research restrictions? Please check biosecurity documents and list of **select agents and toxins** (CDC): https://www.selectagents.gov/sat/list.htm	Not Applicable	
If you used a select agent, is the security level of the lab appropriate and reported in the manuscript?	Not Applicable	
If a study is subject to dual use research of concern regulations, is the name of the **authority granting approval and reference number** for the regulatory approval provided in the manuscript?	Not Applicable	

Reporting

The MDAR framework recommends adoption of discipline-specific guidelines, established and endorsed through community initiatives. Journals have their own policy about requiring specific guidelines and recommendations to complement MDAR.

Adherence to community standards	Information included in the manuscript?	In which section is the information available? (Reagents and Tools Table, Materials and Methods, Figures, Data Availability Section)
State if relevant guidelines or checklists (e.g., **ICMJE, MIBBI, ARRIVE, PRISMA**) have been followed or provided.	Not Applicable	
For **tumor marker prognostic studies,** we recommend that you follow the **REMARK** reporting guidelines (see link list at top right). See author guidelines, under 'Reporting Guidelines'. Please confirm you have followed these guidelines.	Not Applicable	
For **phase II and III randomized controlled trials**, please refer to the **CONSORT** flow diagram (see link list at top right) and submit the CONSORT checklist (see link list at top right) with your submission. See author guidelines, under 'Reporting Guidelines'. Please confirm you have submitted this list.	Not Applicable	

Data Availability

Data availability	Information included in the manuscript?	In which section is the information available? (Reagents and Tools Table, Materials and Methods, Figures, Data Availability Section)
Have **primary datasets** been deposited according to the journal's guidelines (see 'Data Deposition' section) and the respective accession numbers provided in the Data Availability Section?	Yes	Data Availability
Were **human clinical and genomic datasets** deposited in a public access-controlled repository in accordance to ethical obligations to the patients and to the applicable consent agreement?	Not Applicable	
Are **computational models** that are central and integral to a study available without restrictions in a machine-readable form? Were the relevant accession numbers or links provided?	Not Applicable	
If publicly available data were reused, provide the respective **data citations in the reference list**.	Not Applicable	
